# Phenotypic Dissection of Bone Mineral Density Reveals Skeletal Site Specificity and Facilitates the Identification of Novel Loci in the Genetic Regulation of Bone Mass Attainment

**DOI:** 10.1371/journal.pgen.1004423

**Published:** 2014-06-19

**Authors:** John P. Kemp, Carolina Medina-Gomez, Karol Estrada, Beate St Pourcain, Denise H. M. Heppe, Nicole M. Warrington, Ling Oei, Susan M. Ring, Claudia J. Kruithof, Nicholas J. Timpson, Lisa E. Wolber, Sjur Reppe, Kaare Gautvik, Elin Grundberg, Bing Ge, Bram van der Eerden, Jeroen van de Peppel, Matthew A. Hibbs, Cheryl L. Ackert-Bicknell, Kwangbom Choi, Daniel L. Koller, Michael J. Econs, Frances M. K. Williams, Tatiana Foroud, M. Carola Zillikens, Claes Ohlsson, Albert Hofman, André G. Uitterlinden, George Davey Smith, Vincent W. V. Jaddoe, Jonathan H. Tobias, Fernando Rivadeneira, David M. Evans

**Affiliations:** 1 MRC Integrative Epidemiology Unit at the University of Bristol, Bristol, United Kingdom; 2 University of Queensland Diamantina Institute, Translational Research Institute, Brisbane, Queensland, Australia; 3 Department of Internal Medicine, Erasmus University Medical Center, Rotterdam, The Netherlands; 4 The Generation R Study Group, Erasmus University Medical Center, Rotterdam, The Netherlands; 5 Department of Epidemiology, Erasmus University Medical Center, Rotterdam, The Netherlands; 6 Netherlands Genomics Initiative (NGI)-sponsored Netherlands Consortium for Healthy Aging (NCHA), The Netherlands; 7 Analytic and Translational Genetics Unit, Massachusetts General Hospital, Boston, Massachusetts, United States of America; 8 School of Oral and Dental Sciences, University of Bristol, Bristol, United Kingdom; 9 School of Experimental Psychology, University of Bristol, Bristol, United Kingdom; 10 Department of Paediatrics, Erasmus University Medical Center, Rotterdam, The Netherlands; 11 Department of Twin Research and Genetic Epidemiology, King's College London, London, United Kingdom; 12 Department of Medical Biochemistry, Oslo University Hospital, Ullevaal, Oslo, Norway; 13 Department of Medical Biochemistry, Oslo Deacon Hospital, Oslo, Norway; 14 Department of Human Genetics, McGill University, Montréal, Canada; 15 McGill University and Genome Québec Innovation Centre, Montréal, Canada; 16 Department of Computer Science, Trinity University, San Antonio, Texas, United States of America; 17 The Jackson Laboratory, Bar Harbor, Maine, United States of America; 18 Department of Medical and Molecular Genetics, Indiana University School of Medicine, Indianapolis, Indiana, United States of America; 19 Department of Medicine, Indiana University School of Medicine, Indianapolis, Indiana, United States of America; 20 Center for Bone and Arthritis Research, Institute of Medicine, Sahlgrenska Academy, University of Gothenburg, Gothenburg, Sweden; 21 School of Clinical Sciences, University of Bristol, Bristol, United Kingdom; Dartmouth College, United States of America

## Abstract

Heritability of bone mineral density (BMD) varies across skeletal sites, reflecting different relative contributions of genetic and environmental influences. To quantify the degree to which common genetic variants tag and environmental factors influence BMD, at different sites, we estimated the genetic (r_g_) and residual (r_e_) correlations between BMD measured at the upper limbs (UL-BMD), lower limbs (LL-BMD) and skull (SK-BMD), using total-body DXA scans of ∼4,890 participants recruited by the Avon Longitudinal Study of Parents and their Children (ALSPAC). Point estimates of r_g_ indicated that appendicular sites have a greater proportion of shared genetic architecture (LL-/UL-BMD r_g_ = 0.78) between them, than with the skull (UL-/SK-BMD r_g_ = 0.58 and LL-/SK-BMD r_g_ = 0.43). Likewise, the residual correlation between BMD at appendicular sites (r_e_ = 0.55) was higher than the residual correlation between SK-BMD and BMD at appendicular sites (r_e_ = 0.20–0.24). To explore the basis for the observed differences in r_g_ and r_e_, genome-wide association meta-analyses were performed (n∼9,395), combining data from ALSPAC and the Generation R Study identifying 15 independent signals from 13 loci associated at genome-wide significant level across different skeletal regions. Results suggested that previously identified BMD-associated variants may exert site-specific effects (i.e. differ in the strength of their association and magnitude of effect across different skeletal sites). In particular, variants at *CPED1* exerted a larger influence on SK-BMD and UL-BMD when compared to LL-BMD (*P* = 2.01×10^−37^), whilst variants at *WNT16* influenced UL-BMD to a greater degree when compared to SK- and LL-BMD (*P* = 2.31×10^−14^). In addition, we report a novel association between *RIN3* (previously associated with Paget's disease) and LL-BMD (rs754388: *β* = 0.13, SE = 0.02, *P* = 1.4×10^−10^). Our results suggest that BMD at different skeletal sites is under a mixture of shared and specific genetic and environmental influences. Allowing for these differences by performing genome-wide association at different skeletal sites may help uncover new genetic influences on BMD.

## Author Summary

The heritability of bone mineral density (BMD) varies across skeletal sites, reflecting different relative contributions of genetic and environmental influences. To investigate whether the genes underlying bone acquisition act in a site-specific manner, we quantified the shared genetic influences across axial and appendicular skeletal sites by estimating the genetic and residual correlation of BMD at the upper limb, lower limb and the skull. Our results suggest that different skeletal sites as measured by total-body Dual-Energy X-Ray Absorptiometry are to a certain extent under distinct genetic and environmental influences. To further explore the basis for these differences, genome-wide association meta-analyses were performed to identify genetic loci that are preferentially associated with one or more skeletal regions. Variants at 13 loci (including *RIN3*, a novel BMD associated locus) reached genome-wide significance and several displayed evidence of differential association with BMD across the different skeletal sites in particular *CPED1 and WNT16*. Our results suggest that it may be advantageous to decompose the total-body BMD measures and perform GWAS at separate skeletal regions. By allowing for site-specific differences, new genetic variants affecting BMD and future risk of osteoporosis may be uncovered.

## Introduction

Bone mineral density (BMD) at the femoral neck and lumbar spine [as measured by dual-energy X-ray absorptiometry, (DXA)], represents the primary diagnostic marker for osteoporosis as it serves as a good predictor of bone strength and fracture risk in adults [1]. Bone strength and fracture risk are influenced by: i) bone acquisition in childhood, adolescence and young adulthood ii) the subsequent maintenance of bone mass over the life course and iii) the progressive loss of bone in later life [2], [3]. Large-scale genome-wide association studies (GWAS) using adult-BMD measured at the femoral neck (FN) and lumbar spine (LS) have successfully identified variants in 56 loci explaining 4–5% of the phenotypic variance in adult-BMD [4]–[6]. However, it is possible that the genetic variants influencing bone acquisition are different from the ones involved in bone maintenance and bone loss across the life course. Consequently, GWAS using paediatric-BMD measurements have recently been performed with the goal of identifying novel genetic variants primarily associated with bone acquisition, whilst limiting the noise introduced by bone maintenance and bone loss [7]. This approach has resulted in the successful identification of novel BMD associated variants in the *WNT16* [7] and Osterix (*SP7*) loci [8] and it is highly likely that more variants will be discovered as the sample size of these paediatric studies increases.

In growing children, changes in bone area create artefacts influencing the reproducibility, comparability and interpretation of DXA measurements. For this reason, regions of interest (ROI) containing larger bone areas [i.e. total-body, (TB)], which are less prone to these artefacts, are preferred for paediatric evaluations of bone health [9]. The skull region is generally excluded from TB-DXA scans as its relative contribution to bone mass is proportionally larger with respect to the rest of the body in children, and its inclusion has been shown to make diagnostic interpretation difficult [10]. However, from a locus discovery perspective, it may be advantageous to partition TB-DXA further into different regions, such as the upper and lower limbs and the skull. This is important if genetic heterogeneity exists in terms of loci differentially affecting BMD at different skeletal sites, or whose effect is greater at some locations than in others. Considering that environmental factors (i.e. mechanical loading) influence skeletal sites differently, analysis of skull-BMD may be particularly informative and even provide greater power to identify genetic variants. This is the case given that the skull is less influenced by mechanical loading than appendicular and other axial sites. Further, the skull is frequently affected in monogenic conditions involving the skeleton. For example, craniofacial abnormalities such as thickening of the cranium and skull base are cardinal features of van Buchems disease, Sclerosteosis and other sclerosing bone dysplasias [11], [12].

In the current study we examined whether genetic factors influence bone mass accrual in a site-specific manner, by performing regional analysis of TB-DXA scans, focussing on the total-body less head (TBLH), lower limb (LL), upper limb (UL), and skull (SK) regions. Using genome-wide complex trait analysis (GCTA) on participants from the Avon Longitudinal Study of Parents and their Children (ALSPAC), we assessed the proportion of BMD variance explained by common genetic variants, across each sub-region and additionally determined the shared genetic and residual correlation between each sub-region. Subsequently, we performed a genome-wide association (GWA) meta-analysis of BMD at each skeletal site in the ALSPAC and Generation R studies and went on to identify factors, which preferentially influence one or more skeletal regions.

## Results

### Phenotypic correlation and genome-wide complex trait analysis of BMD at different regions

Univariate GCTA analysis revealed that common genotyped variants explained a greater proportion of the variance in SK-BMD (v_g_ = 0.51, SE = 0.07, *P* = 2.0×10^−13^) than LL- (v_g_ = 0.40, SE = 0.07, *P* = 8.0×10^−9^) or UL- (v_g_ = 0.39, SE = 0.07, *P* = 2.0×10^−8^) BMD. Higher *phenotypic* correlations were observed when comparing LL- and UL-BMD than with SK-BMD (Table 1). Similarly, bivariate GCTA analysis indicated that the strongest genetic correlation was between BMD at the two appendicular sites, whereas the *genetic* correlations involving SK-BMD were more moderate. The *residual* correlation between the different sites was in general smaller than the *genetic* correlation, and was higher for BMD between the appendicular sites than for comparisons involving the skull (Table 1). Highly similar magnitudes and patterns of residual correlations were obtained for a sensitivity analysis in which BMD at all skeletal sites was corrected for age, gender, weight and height (Table S1).

**Table 1:** Bivariate GCTA estimates of the genetic and residual correlations for bone mineral density measurements at the total-body less head, lower limb, upper limb and skull for the ALSPAC cohort.

TRAIT 1	TRAIT2	SAMPLE SIZE	r_g_	SE	r_e_	SE	*P*
**SK-BMD**	**TBLH-BMD**	9732	0.52	0.088	0.29	0.086	4.1×10^−6^
	**LL-BMD**	9732	0.44	0.099	0.20	0.088	1.2×10^−3^
	**UL-BMD**	9732	0.58	0.090	0.24	0.085	9.1×10^−7^
**LL-BMD**	**UL-BMD**	9782	0.78	0.067	0.55	0.055	1.4×10^−7^

TBLH-BMD = total body less head BMD, (LL-BMD) = lower limb BMD, (UL-BMD) = upper limb BMD, (SK-BMD) = skull BMD, r_g_ = genetic correlation between trait 1 and trait 2. r_e_ = residual correlation between trait 1 and trait 2. All traits, excluding SK-BMD were adjusted for age, gender and weight. SK-BMD was adjusted for age, gender and height. *P*-refers to the *P*-value for the likelihood ratio test of whether r_g_ = 0. Phenotypic correlations (r_p)_ were as follows: SK-BMD/TBLH-BMD (r_p_ = 0.40, SE = 0.013, P<0.001), SK-BMD/LL-BMD (r_p_ = 0.31, SE = 0.013, P<0.001), SK-BMD/UL-BMD (r_p_ = 0.40, SE = 0.013, P<0.001) and LL-BMD/UL-BMD (r_p_ = 0.64, SE = 0.010, P<0.001).

### Genome wide meta-analysis of BMD across different skeletal regions in ALSPAC and Generation R

**Figure 1:**
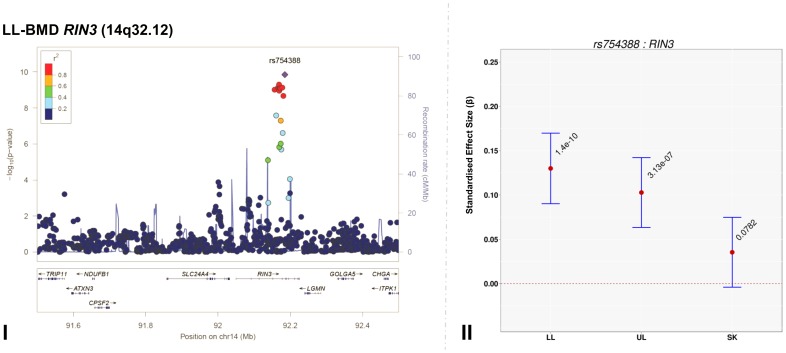
Regional association plot of the primary signal (rs754388) associate with lower limb-BMD at 14q32.12, in addition to a comparison of the effect of rs754388 on bone mineral density at three different skeletal sites. For I and II: Circles show GWA meta-analysis *P*-values and positions of SNPs found within the 14q32.12 locus. The top SNP, i.e. rs754388, is denoted by a diamond. Different colours indicate varying degrees of pair-wise linkage disequilibrium (HapMap 2 CEPH) between the top SNP and all other SNPs. For II: The per-allele effect in standard deviations (SD) (red dot) and the 95% confidence interval (error bar) of rs754388 for lower limb (LL), upper limb (UL) and skull (SK) BMD, plotted with the strength of evidence against the null hypothesis of no association.

Genome-wide association meta-analyses were performed on TBLH-, LL-, UL- and SK-BMD, using regional BMD data derived from ∼9,395 TB-DXA scans. Detailed population characteristics of the ALSPAC and Generation R cohorts are summarised in Tables S2 and S3. Summary statistics from each GWAS (after meta-analysis) indicated that negligible systematic inflation of test statistics was observed (META λ_GC_ = 1.01–1.03). In contrast, a marked deviation from the null was observed in the tail of the distribution amongst the lowest observed *P*-values of the meta-association analyses (Figure S1). SNPs in thirteen published BMD-associated loci exceeded the genome-wide significance (GWS) threshold for association (*P*≤5×10^−8^, Table 2). They included variants which mapped close to, or within: *WNT4* (1p36.12), *WNT16/FAM3C/CPED1* (7q31.31) for all skeletal sites measured, *EYA4* (6q23.2), *COLEC10/TNFRS11B* (8q24.12), *LIN7C/LGR4* (11p14.1), *PPP6R3/LRP5* (11q13.2) and *TNFRSF11A* (18q21.33) for SK-BMD, *CENPW/RSPO3* (6q22.32) for UL- and SK-BMD, *TNFSF11* (13q14.11) and *GALNT3* (2q24.3) for UL- and TBLH-BMD. In addition, variants proximal to or within *FUBP3* (9q34.11) and *KLHDC5/PTHLH* (12p11.22) were associated with TBLH- and LL-BMD. Furthermore, a novel signal (top SNP rs754388, 14q32.12), located within Ras and Rab interactor 3 (*RIN3*) achieved genome-wide significance after meta-analysis of LL-BMD (*β* = 0.13, SE = 0.02, *P* = 1.4×10^−10^, Figure 1-I, Table 2) and TBLH-BMD (*β* = 0.12, SE = 0.02, *P* = 3.0×10^−9^, Table 2 and Figure S2). The full list of all genome-wide significant SNPs and regional association plots for each locus and skeletal site are presented in Supplementary Tables S4, S5, S6, S7 and Figures S2, S3, S4, S5.

**Table 2:** Top genome-wide significant SNPs associated with bone mineral density of the total-body less head, lower limb, upper limb and skull.

						ALSPAC (n = 5,330/5,299*)				Generation R (n = 4,086)				META-ANALYSIS (n = 9,416/9,385*)					
TRAIT	RSID	LOCUS	POS	GENE	EA	EAF	*β*	SE	*P*	EAF	*β*	SE	*P*	EAF	*β*	SE	*P*	I^2^	*P*_HET_
**TBLH-BMD**	rs3765350	1p36.12	22319903	*WNT4*	A	0.78	0.106	0.023	5.75×10^−6^	0.78	0.109	0.026	2.92×10^−5^	0.78	0.107	0.017	**7.04×10^−10^**	0	9.32×10^−1^
	rs6726821	2q24.3	166286360	*GALNT3*	T	0.51	0.094	0.019	1.32×10^−6^	0.58	0.087	0.022	8.76×10^−5^	0.54	0.091	0.015	**3.95×10^−10^**	0	8.11×10^−1^
	rs7776725	7q31.31	120820357	*FAM3C* **	C	0.27	0.136	0.023	**3.65×10^−9^**	0.26	0.188	0.026	**7.67×10^−13^**	0.27	0.159	0.017	**5.67×10^−20^**	54.7	1.38×10^−1^
	rs7466269	9q34.11	132453905	*FUBP3*	A	0.64	0.094	0.020	3.72×10^−6^	0.66	0.072	0.023	2.01×10^−3^	0.65	0.084	0.015	**3.26×10^−8^**	0	4.74×10^−1^
	rs4420311	12p11.22	27875457	*KLHDC5* **	G	0.47	0.080	0.020	7.84×10^−5^	0.44	0.092	0.024	1.03×10^−4^	0.46	0.085	0.016	**4.44×10^−8^**	0	7.03×10^−1^
	rs17536328	13q14.11	42041029	*TNFSF11*	T	0.43	0.079	0.020	6.14×10^−5^	0.40	0.095	0.022	1.53×10^−5^	0.42	0.086	0.015	**7.58×10^−9^**	0	5.94×10^−1^
	rs754388	14q32.12	92185163	*RIN3*	C	0.81	0.098	0.026	1.34×10^−4^	0.83	0.149	0.031	1.41×10^−6^	0.82	0.120	0.020	**2.96×10^−9^**	36.0	2.11×10^−1^
**LL-BMD**	rs3765350	1p36.12	22319903	*WNT4*	A	0.78	0.103	0.023	1.05×10^−5^	0.78	0.090	0.026	5.74×10^−4^	0.78	0.097	0.018	**2.89×10^−8^**	0	7.12×10^−1^
	rs2908004	7q31.31	120757005	*WNT16* **	A	0.44	0.093	0.020	3.63×10^−6^	0.50	0.108	0.022	1.25×10^−6^	0.47	0.100	0.015	**3.01×10^−11^**	0	6.19×10^−1^
	rs7466269	9q34.11	132453905	*FUBP3*	A	0.64	0.097	0.020	1.85×10^−6^	0.66	0.074	0.023	1.59×10^−3^	0.65	0.087	0.015	**1.51×10^−8^**	0	4.57×10^−1^
	rs4420311	12p11.22	27875457	*KLHDC5* **	G	0.47	0.086	0.020	2.06×10^−5^	0.44	0.087	0.024	2.28×10^−4^	0.46	0.086	0.016	**3.21×10^−8^**	0	9.75×10^−1^
	rs754388	14q32.12	92185163	*RIN3*	C	0.81	0.119	0.026	3.47×10^−6^	0.83	0.145	0.031	2.51×10^−6^	0.82	0.130	0.020	**1.40×10^−10^**	0	5.26×10^−1^
**UL-BMD**	rs2235529	1p36.12	22323074	*WNT4*	C	0.84	0.099	0.027	1.98×10^−4^	0.85	0.14	0.031	5.99×10^−6^	0.85	0.117	0.021	**1.21×10^−8^**	0	3.22×10^−1^
	rs6726821	2q24.3	166286360	*GALNT3*	T	0.51	0.078	0.019	6.44×10^−5^	0.58	0.089	0.022	5.61×10^−5^	0.54	0.083	0.015	**1.13×10^−8^**	0	7.07×10^−1^
	rs1262476	6q22.32	127028689	*CENPW* **	G	0.76	0.130	0.022	**6.37×10^−9^**	0.79	0.062	0.028	2.37×10^−2^	0.77	0.104	0.018	**2.93×10^−9^**	72.3	5.76×10^−2^
	rs798943	7q31.31	120546135	*CPED1* **	G	0.61	0.187	0.020	**8.84×10^−21^**	0.62	0.205	0.023	**1.28×10^−19^**	0.61	0.195	0.015	**1.47×10^−37^**	0	5.57×10^−1^
	rs9525638	13q14.11	42026577	*TNFSF11*	C	0.43	0.094	0.020	1.63×10^−6^	0.41	0.083	0.022	1.52×10^−4^	0.42	0.089	0.015	**2.47×10^−9^**	0	7.13×10^−1^
**SK-BMD**	rs3920498	1p36.12	22365474	*WNT4*	G	0.79	0.144	0.024	**4.56×10^−9^**	0.82	0.118	0.030	8.40×10^−5^	0.80	0.134	0.019	**1.56×10^−12^**	0	5.01×10^−1^
	rs2130604	6q22.32	126862254	*CENPW* **	T	0.24	0.117	0.022	1.89×10^−7^	0.23	0.106	0.026	6.47×10^−5^	0.24	0.112	0.017	**3.33×10^−11^**	0	7.48×10^−1^
	rs3012465	6q23.2	133392629	*EYA4*	G	0.65	0.125	0.020	**7.00×10^−10^**	0.69	0.129	0.023	**3.06×10^−8^**	0.67	0.127	0.015	**8.29×10^−17^**	0	8.96×10^−1^
	rs13223036	7q31.31	120534544	*CPED1* **	T	0.63	0.170	0.020	**3.09×10^−17^**	0.65	0.167	0.023	**6.21×10^−13^**	0.64	0.169	0.015	**1.53×10^−28^**	0	9.22×10^−1^
	rs2450083	8q24.12	120132723	*COLEC10* **	T	0.48	0.105	0.020	1.66×10^−7^	0.47	0.098	0.023	2.16×10^−5^	0.47	0.102	0.015	**2.13×10^−11^**	0	8.20×10^−1^
	rs10835187	11p14.1	27462253	*LIN7C* **	C	0.45	0.145	0.020	1.05×10^−13^	0.50	0.106	0.022	1.63×10^−6^	0.47	0.127	0.015	**1.63×10^−17^**	41.1	1.93×10^−1^
	rs12272917	11q13.2	68019946	*PPP6R3* **	T	0.74	0.130	0.022	**4.01×10^−9^**	0.76	0.080	0.026	2.52×10^−3^	0.75	0.109	0.017	**1.34×10^−10^**	53.0	1.45×10^−1^
	rs884205	18q21.33	58205837	*TNFRSF11A*	C	0.72	0.092	0.023	5.38×10^−5^	0.80	0.123	0.030	3.88×10^−5^	0.75	0.104	0.018	**1.84×10^−8^**	0	4.15×10^−1^

(TBLH-BMD) = total-body less head BMD, (LL-BMD) = lower limb BMD, (UL-BMD) = upper limb BMD, (SK-BMD) = skull BMD, (GENE) = closest gene, (POS) = position in the genome based on hg18, (EAF) = effect allele frequency, (*β*) = estimates of effect size expressed as adjusted SD per copy of the effect allele (EA), (SE) = standard error of *β*, (*P*) = pvalue, (I^2^) = Cochran's Q statistic evaluating heterogeneity, (*P*_HET_) = evidence of heterogeneity and

*Sample sizes used for SK-BMD genome-wide meta-analysis.

**Please note that *PTHLH* is also located at the 12p11.22 locus containing *KLHDC5, RSPO3* is also located at the 6q.22.32 locus containing *CENPW, FAM3C and CPED1* are also located at the 7q.31.31 locus containing *WNT16*, *TNFRSF11B* is also located at the 8q.24.12 locus containing *COLEC10, LGR4* is also located at the 11p14.1 locus containing *LIN7C* and *LRP5* is also located at the 11q13.2 locus containing *PPP6R3*.

A followup of 66 independent SNPs at 58 loci, previously associated with BMD [4], [13], indicated that 31 loci showed nominal evidence of association (*P*<0.05) with TBLH-BMD, 28 with LL-BMD, 26 with UL-BMD and 26 with SK-BMD (versus an expectation of 3.3. per phenotype) (Table S8). A similar distribution of associations was also observed when a more conservative threshold considering multiple hypothesis testing was adopted that took into account the fact that 66 variants and four phenotypes had been tested (i.e. *α*<1.9×10^−4^). Using this threshold nine variants showed evidence of association with TBLH-BMD, seven with LL-BMD, six with UL-BMD and 10 with SK-BMD (versus an expectation of 0.1 per phenotype). We note that in all cases where nominal significance was reached, the direction of effect was consistent with previous studies.

To ensure that our results were robust to the possible effects of population stratification and our choice of covariates, we performed sensitivity analyses where we either restricted our analysis to white European individuals only, or adjusted for the same set of covariates across all analyses (i.e. age, gender, height, and weight). Similar effect sizes and patterns of association were observed for the top SNPs when adjusting BMD measures of all four regions for age, gender, height and weight (Model 1a, Table S9) and when limiting the GWAS meta-analysis to individuals of European ancestry (Model 1b, Table S9). In both sensitivity analyses, no additional loci reached the threshold of genome-wide association (Figure S6 and S7).

### Identification of novel BMD-associated signals

**Figure 2:**
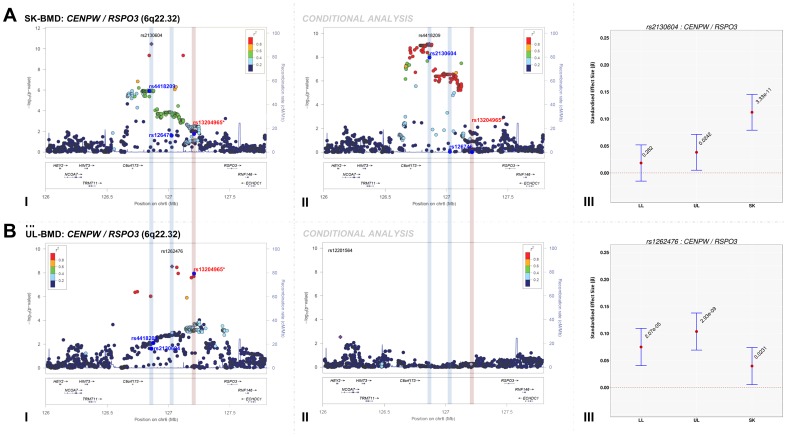
Regional association plots of the top skull- and upper limb-BMD associated SNPs at the 6q22.32 locus before and after conditioning on published SNP (rs13204965*) in addition to a comparison of the effect sizes of the top skull- (rs2130604) and upper limb-BMD (rs1262476) associated SNP (before conditional analysis) on BMD at three different skeletal sites. For I and II: Circles show GWA meta-analysis *P*-values and positions of SNPs found within each locus. Top SNPs are denoted by diamonds. Different colours indicate varying degrees of pairwise linkage disequilibrium (HapMap 2 CEPH) between the top SNP and all other SNPs. Blue vertical shaded areas indicate the position of rs2130604 (top SNP A-I) and rs1262476 (top SNP B-I) for each analysis. The red vertical shaded area represents the position of the published SNP (rs13204965*). Rsids of relevant SNPs (blue dots) have been provided. For III: The per allele effect in SD (red dot) and 95% confidence intervals (error bar) of each top SNP (before conditional analysis) for lower limb (LL), upper limb (UL) and skull (SK) BMD are plotted with their specific strength of evidence against the null hypothesis of no association. Please note: *RSPO3* is also found in the 6q.22.32 locus containing *CENPW*.

We assessed the presence of novel secondary association signals at loci that contained genome-wide associated variants. Meta-analysis of conditional association analyses resulted in the attenuation of the majority of our top association signals (Table S10, Figures S2, S3, S4, S5), indicating that these loci were not independent from signals previously reported by other BMD GWAS. However, the top signal for SK-BMD (rs2130604, *β* = 0.11, SE = 0.02, *P* = 3.3×10^−11^), mapping near *RSPO3*, but closest to C*ENPW* (previously known as *C6orf173*, 6q22.32, Figure 2A-I, Table 2) was only marginally attenuated after conditional analysis (rs2130604, *β* = 0.10, SE = 0.02, *P* = 7.1×10^−9^, Figure 2A-II, Table S10). This suggests that rs2130604 is largely independent from the previously reported signal at *RSPO3* (rs13204965, 6q22.32), which was identified in a GWAS of individuals with extremely high or low BMD at the hip [12] and later replicated in the second GEnetic Factors for OSteoporosis Consortium (GEFOS-II) BMD meta-analysis [4]. This observation is further supported by low estimates of LD (r^2^ = 0.14) between rs2130604 and rs13204965. Furthermore, the secondary signal (after conditional analysis) reached the estimated significance threshold of association after multiple testing correction (i.e. *P*≤7.2×10^−5^).

Interestingly, after conditioning rs4418209 (another SNP in the same locus) on the published BMD-associated SNP rs13204965, we observed a marked increase in its evidence of association [(*β* = 0.07, SE = 0.02, *P* = 1.1×10^−6^) *_before_* and (*β* = 0.09, SE = 0.01, *P* = 7.9×10^−10^) *_after_*], (Figure 2A-II, Table S10). The rs4418209 variant maps closest to *CENPW* (6q22.32) and is in moderate LD with the secondary independent signal (rs2130604, r^2^ = 0.43) and in low LD with the published *RSPO3* SNP (rs13204965, r^2^ = 0.12). Whilst no other SNPs reached the threshold for declaring genome-wide significance (*P*<5×10^−8^), variants from three loci still yielded suggestive evidence for association (*P*<1×10^−5^) after conditional analyses (Table S10 and Figures S2, S3, S4, S5). They included: i) *KLHDC5/PTHLH* (rs4420311, 12p11.22) associated with TBLH- (*β* = 0.08, SE = 0.016, *P* = 7.6×10^−7^) and LL-BMD (*β* = 0.08, SE = 0.016, *P* = 1.9×10^−6^), ii) *TNFSF11* (rs17536328 and rs2148072, 13q14.11) associated with TBLH- (*β* = 0.08, SE = 0.015, *P* = 5.6×10^−7^) and UL-BMD (*β* = 0.07, SE = 0.015, *P* = 2.1×10^−6^) respectively and iii) *LIN7C/LGR4* [rs10160456, 11p14.1, (*β* = 0.07, SE = 0.015, *P* = 7.8×10^−6^)] with SK-BMD. After conditional analysis, the secondary signal at *LIN7C/LGR4* (i.e. rs10160456) mapped closest to *CCDC34* and not to *LIN7C*, the gene closest to the primary signal. All these three loci might represent novel secondary signals as the residual signal reached the predicted locus specific threshold of association after multiple testing correction (Table S10). However, we cannot exclude that both associations (i.e. the primary and secondary signals) could potentially arise from their association with one or more causal variants, which could occur, on the same haplotype background. For example, one such BMD-associated rare variant has recently been identified in *LGR4* in Icelandic populations although this mutation appears specific to this population and therefore is unlikely to account for the *LIN7C/LGR4* signal we observe [14].

### Comparison of the magnitude of the effect sizes of genome-wide significant SNPs across skeletal-sites

**Figure 3:**
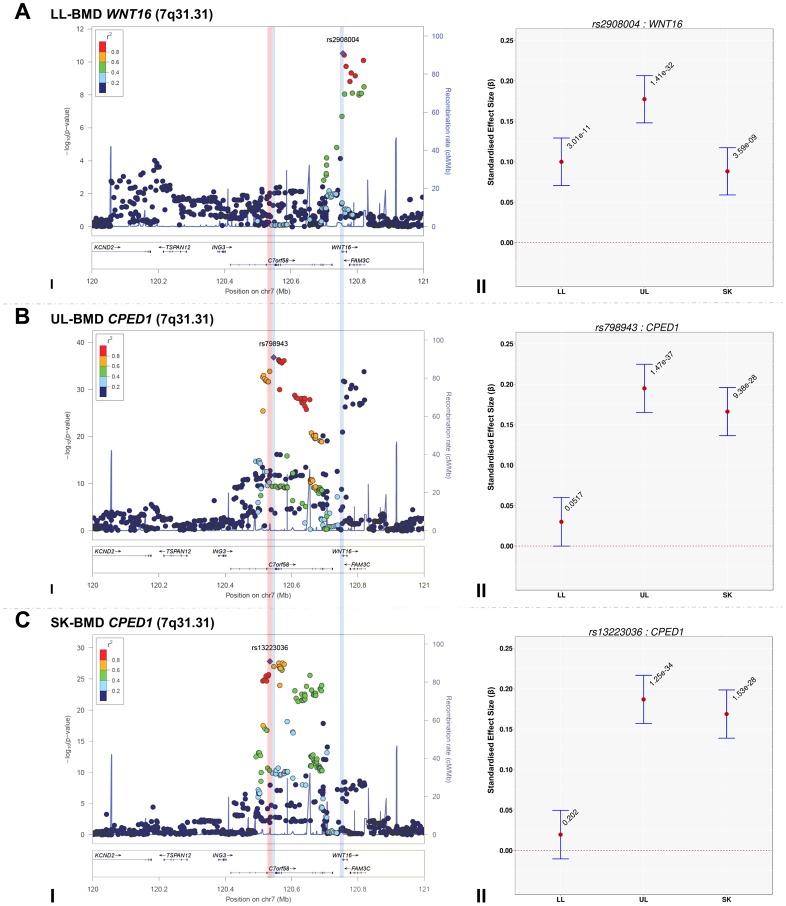
Regional association plots of the top SNPs associated with total-body less head-, lower limb-, upper limb- and skull-BMD at the 7q31.31 locus, in addition to a comparison of the effect size of the top site-specific SNP on BMD at the three different skeletal sites. For I: Circles show GWA meta-analysis *P*-values and positions of SNPs found within the 7q31.31 locus. Top SNPs are denoted by diamonds. Different colours indicate varying degrees of pair-wise linkage disequilibrium (HapMap 2 CEPH) between the top SNP and all other SNPs. Blue vertical shaded areas indicate the position of rs7776725 (top SNP A-I) and rs2908004 (top SNP B-I) and rs798943 (top SNP C-I) for each analysis. The red vertical shaded area represents the position of rs13223036 (top SNP D-I). For II: The per allele effect in SD (red dot) and the 95% confidence interval (error bar) of the top SNP for lower limb (LL), upper limb (UL) and skull (SK) are plotted with their specific strength of evidence against the null hypothesis of no association. Please note: *FAM3C* and *CPED1* are also located at the 7q.31.31 locus containing *WNT16*.

The standardized per allele effect sizes (*β*) of all the top BMD-associated SNPs were compared across three (SK-, UL-, and LL) BMD regions to determine if they preferentially influenced one or more skeletal sites (Table 3, Figure S8, S9, S10, S11). Effect sizes of the following variants: rs2130604 (*CENPW/RSPO3*, 6q22.32), rs3012465 (*EYA4*, 6q23.2), rs2450083 (*COLEC10/TNFRS11B*, 8q24.12), rs10835187 (*LIN7C/LGR4*, 11p14.1) and rs884205 (*TNFRSF11A*, 18q21.33) appeared to be largest for SK-BMD when compared to UL- and LL-BMD (Figure S8). Furthermore, differences in the magnitude of the effect were evident when comparing independent genetic variants that occurred in close proximity within a locus, as shown at the *CENPW/RSPO3* (6q22.32) and *WNT16/FAM3C/CPED1* (7q31.31) loci. Specifically, the independent signal (rs2130604, *CENPW/RSPO3*, 6q22.32) associated with SK-BMD [*β* = 0.11 (CI_95_: 0.08, 0.15) *P* = 3.3×10^−11^], was not strongly related to LL-BMD [*β* = 0.02 (CI_95_: −0.02, 0.05), *P* = 0.28], or UL-BMD [*β* = 0.04, (CI_95_: 0.01, 0.07), *P* = 0.02] (Table 3, Figure 2A-III). In contrast, a neighbouring SNP (rs1262476) primarily associated with UL-BMD appeared to influence BMD across all skeletal sites (Table 3, Figure 2B-III). Differential patterns of association between SNPs at neighbouring positions were also observed at the *WNT16* locus (Table 3, Figure 3A–C). Effect sizes were largest for UL-BMD at rs2908004 (*WNT16*, 7q31.31, Table 3, Figure 3A-II) when compared to SK- and LL-BMD. Interestingly, as compared to LL-BMD, we observed consistently larger effect sizes for rs13223036 and rs798943 (*CPED1*, previously known as *C7orf58*) for SK- and UL-BMD, (Table 3, Figure 3B-II and 3C-II).

**Table 3:** Comparison of effect sizes and the strength of association of all variants that exceeded genome-wide significance at one or more skeletal sites.

				LL-BMD				UL-BMD				S-BMD				Fisher's Product P*	
LOCUS	RSID	GENE	EA	*β*	CI-L	CI-U	*P*	*β*	CI-L	CI-U	*P*	*β*	CI-L	CI-U	*P*	Chi	*P*
1p36.12	rs3765350	*WNT4*	A	0.097	0.06	0.13	2.89×10^−8^	0.091	0.06	0.13	1.82×10^−7^	0.115	0.08	0.15	3.56×10^−11^	9.71	4.55×10^−2^
1×36.12	rs2235529	*WNT4*	C	0.106	0.07	0.15	3.27×10^−7^	0.117	0.08	0.16	1.21×10^−8^	0.143	0.10	0.18	2.99×10^−12^	8.34	7.99×10^−2^
1p36.12	rs3920498	*WNT4*	G	0.075	0.04	0.11	8.41×10^−5^	0.097	0.06	0.13	2.85×10^−7^	0.134	0.10	0.17	1.56×10^−12^	8.93	6.29×10^−2^
2q24.3	rs6726821	*GALNT3*	T	0.078	0.05	0.11	1.03×10^−7^	0.083	0.05	0.11	1.13×10^−8^	0.031	0.00	0.06	3.37×10^−2^	15.26	4.19×10^−3^
6q22.32	rs1262476	*CENPW*	G	0.075	0.04	0.11	2.07×10^−5^	0.104	0.07	0.14	2.93×10^−9^	0.040	0.01	0.07	2.31×10^−2^	27.26	1.76×10^−5^
6q22.32	rs2130604	*CENPW*	T	0.018	−0.02	0.05	2.82×10^−1^	0.038	0.01	0.07	2.42×10^−2^	0.112	0.08	0.15	3.33×10^−11^	15.09	4.51×10^−3^
6q23.2	rs3012465	*EYA4*	G	0.019	−0.01	0.05	2.09×10^−1^	0.051	0.02	0.08	7.45×10^−4^	0.127	0.10	0.16	8.29×10^−17^	35.66	3.40×10^−7^
7q31.31	rs13223036	*CPED1*	T	0.020	−0.01	0.05	2.02×10^−1^	0.187	0.16	0.22	1.25×10^−34^	0.169	0.14	0.20	1.53×10^−28^	178.00	2.01×10^−37^
7q31.31	rs798943	*CPED1*	G	0.030	0.00	0.06	5.17×10^−2^	0.195	0.17	0.23	1.47×10^−37^	0.166	0.14	0.20	9.38×10^−28^	171.73	4.44×10^−36^
7q31.31	rs2908004	*WNT16*	A	0.100	0.07	0.13	3.01×10^−11^	0.177	0.15	0.21	1.41×10^−32^	0.088	0.06	0.12	3.59×10^−9^	69.96	2.31×10^−14^
8q24.12	rs2450083	*COLEC10*	T	0.015	−0.02	0.05	3.38×10^−1^	0.037	0.01	0.07	1.42×10^−2^	0.102	0.07	0.13	2.13×10^−11^	26.62	2.37×10^−5^
9q34.11	rs7466269	*FUBP3*	A	0.087	0.06	0.12	1.51×10^−8^	0.077	0.05	0.11	3.67×10^−7^	0.052	0.02	0.08	6.83×10−4	2.40	6.63×10^−1^
11p14.1	rs10835187	*LIN7C*	C	0.045	0.02	0.07	3.10×10^−3^	0.041	0.01	0.07	5.53×10^−3^	0.127	0.10	0.16	1.63×10^−17^	38.35	9.47×10^−8^
11q13.2	rs12272917	*PPP6R3*	T	0.065	0.03	0.10	1.38×10^−4^	0.067	0.03	0.10	7.78×10^−5^	0.109	0.08	0.14	1.34×10^−10^	13.50	9.06×10^−3^
12p11.22	rs4420311	*KLHDC5*	G	0.086	0.06	0.12	3.21×10^−8^	0.066	0.04	0.10	2.25×10^−5^	0.037	0.01	0.07	1.58×10^−2^	9.78	4.43×10^−2^
13q14.11	rs9525638	*TNFSF11*	C	0.064	0.04	0.09	2.09×10^−5^	0.089	0.06	0.12	2.47×10^−9^	0.057	0.03	0.09	1.43×10^−4^	9.62	4.73×10^−2^
14q32.12	rs754388	*RIN3*	C	0.130	0.09	0.17	1.40×10^−10^	0.103	0.06	0.14	3.13×10^−7^	0.035	0.00	0.08	7.82×10^−2^	11.10	2.55×10^−2^
18q21.33	rs884205	*TNFRSF11A*	C	0.003	−0.03	0.04	8.58×10^−1^	0.023	−0.01	0.06	2.11×10^−1^	0.104	0.07	0.14	1.84×10^−8^	25.87	3.36×10^−5^

(LL-BMD) = lower limb BMD, (UL-BMD) = upper limb BMD, (SK-BMD) = skull BMD, (GENE) = closest gene, (POS) = position in the genome based on hg18, (EA) = effect allele, (*β*) = estimates of effect size expressed as adjusted SD per copy of the effect allele (EA), (CI-L) = lower limit of the 95% confidence interval for *β*, (CI-U) = upper limit of the 95% confidence interval for *β*, (*P*) = *P*-value.

*Please note that *PTHLH* is also found in the 12p.11.22 locus containing *KLHDC5, RSPO3* is also found in the 6q.22.32 locus containing *CENPW, TNFRSF11B* is also located at the 8q.24.12 locus containing *COLEC10, LGR4* is also located at the 11p.14.1 locus containing *LIN7C* and *LRP5* is also located at the 11q13.2 locus containing *PPP6R3*. *FAM3C* and *CPED1* are also located at the 7q.31.31 locus containing *WNT16*.

To formally determine whether the standardized regression coefficients of each of the above-mentioned variants truly differed across the skeletal sites, we fitted a multivariate normal likelihood model to the raw data in ALSPAC and Generation R (see Methods), and then meta-analysed the results using Fisher's method. Using a conservative threshold (i.e. *α = 5×10^−8^*), we observed robust evidence indicating that i.e. rs13223036 and rs798943, located at *CPED1* exerted strong effects on UL and SK-BMD, when compared to LL-BMD [*P* = 2.01×10^−37^ and *P* = 4.44×10^−36^ (Table3)], whereas the variant rs2908004 (*WNT16*) was strongly related to UL-BMD in comparison to BMD at the other sites (*P* = 2.31×10^−14^). Several variants at other loci were also suggestive of some degree of skeletal site specificity including *EYA4* and *LIN7C*, although they did not formally meet the criteria for statistical significance (Table 3, Figures S8, S9, S10, S11).

### Association of novel variants with hip and spine BMD

To elucidate if any of the novel primary and/or secondary signals, identified during the course of this study, were nominally associated with BMD in adults, we performed a lookup of these variants in the publicly available results of the GEFOS-II meta-analysis of hip and spine BMD (Table S11) [4]. The novel *RIN3* variant (rs754388) was not associated with femoral neck (*P_FN_* = 0.87) and lumbar spine BMD (*P_LS_* = 0.42). The G allele of the *EYA4* variant (rs3012465), associated with increased SK-BMD (*β* = 0.13, SE = 0.02, *P* = 8.3×10^−17^), but surprisingly showed nominal association with decreased BMD at both the hip (*P* = 7.1×10^−3^) and spine (*P* = 0.04). A followup of this variant in a recent published GWAS of 4061 premenopausal women aged 20 to 45 revealed no evidence of association with FN-BMD (*P* = 0.73) [15].

A lookup of the secondary independent SNPs revealed no evidence of a relationship between the TBLH- and LL-BMD-associated *KLHDC5/PTHLH* variant (rs4420311) and associations with hip or spine BMD (*P_FN_* = 0.33 and *P_LS_* = 0.45) in GEFOS-II. Similarly no evidence of association was detected for the SK-BMD-associated variant at *CENPW/RSPO3* (rs2130604: *P_FN_* = 0.98 and *P_LS_* = 0.40). Interestingly, the T allele of the *CENPW/RSPO3* variant (rs4418209), which was associated with increased SK-BMD (*β* = 0.07, SE = 0.02, *P* = 1.1×10^−6^), appeared to be nominally associated with decreased hip BMD (*P* = 5.0×10^−3^) but not spine BMD (*P* = 0.34). Further inspection revealed that the T allele of rs4418209 was nominally associated with decreased BMD at the TBLH (*β* = −0.03, SE = 0.02, *P* = 1.7×10^−2^), LL (*β* = −0.04, SE = 0.02, *P* = 6.5×10^−3^) and UL (*β* = −0.04, SE = 0.02, *P* = 8.2×10^−3^). The T allele of rs17536328 located within *TNFSF11*, associated with increased TBLH-BMD, showed nominal evidence of association with increased hip (*P* = 0.04) but not spine BMD (*P* = 0.87). In contrast, the G allele of an independent *TNFSF11* variant (rs2148072) associated with increased UL-BMD was associated with decreased spine BMD (*P* = 0.05). In addition, the C allele *LIN7C/LGR4* variant (rs10160456) associated with increased SK-BMD showed weak evidence of association with increased hip (*P* = 0.06) and spine (*P* = 0.03) BMD.

### Bioinformatic analysis of *RIN3*

We fine mapped the *RIN3* region by imputing common and rare variants using a reference panel from the 1000 Genomes Project and identified a missense variant (rs117068593) that was in strong linkage disequilibrium (r^2^ = 0.96) with the top LL-and TBLH-BMD associated *RIN3* variant (rs754388). The C allele of rs117068593 (EAF = 0.82) was associated with increased BMD of the lower limbs (*β* = 0.13, SE = 0.020, *P* = 5.97×10^−11^) and total-body less head (*β* = 0.12, SE = 0.020, *P* = 1.87×10^−9^). A search of the SIFT database [16] revealed that the missense variant could negatively affect RIN3 functioning. This prediction was further supported by a search of the Regulome database [17], which suggested that the missense variant alters the binding of the following transcription factors: EBF1, EGR1, SP1, NFKB1 and POLR2A, in lymphoblastic cell lines.

### *RIN3* expression profiling

Evaluation of cis-expression quantitative trait loci (eQTLs) from primary human osteoblasts using array-based gene expression suggested that variants located within 1MB of *RIN3* (i.e. including variants tagging *SLC24A4, LGMN, GOLGA5, CHGA* and *ITPK1*) were nominally associated with *ITPK1* expression (*P* = 0.04). This observation failed to meet the level of significance after correction for multiple testing. Examination of the temporal pattern of gene expression across osteoblastogenesis, using mouse calvarial derived cells, starting with the pre-osteoblast stage, through to mature osteoblasts revealed that *Rin3, Golga5* and *Lgmn*, and *Iptk1* were expressed in this cell type (Figure S12). In contrast, *Slc24a4* and *Chga* were not expressed at all in the pre- or mature osteoblast, as determined by RNAseq. A further investigation of the expression profiles of the aforementioned genes in human mesenchymal stem cells [(hMSCs), differentiated into adipocytes and osteoblasts] and peripheral blood monocytes [(PBMCs) differentiated into osteoclasts] indicated the following: *SLC24A4* was not expressed in any of these cell lines when differentiated, *GOLGA5* had an intermediate expression level in both differentiating hMSCs and PBMCs and *LGMN* was immediately upregulated upon differentiation into adipocytes (8 fold), osteoblasts (5 fold) and osteoclasts [(5 fold), Figure S13 and S14]. Moreover, we found that the expression of *RIN3* was 2-fold downregulated during the proliferative phase of differentiating PBMCs into osteoclasts (Figure S13). Finally a comparison of expression profiles across the *RIN3* region of illiac bone biopsies derived from 39 osteoporotic and 27 healthy postmenopausal donors revealed one transcript (i.e. 220439_at, originating from *RIN3*), that demonstrated reduced expression in the osteoporotic group relative to the control group [*P* = 2.7×10^−3^, (Table S13)].

## Discussion

This study assessed whether regional analysis of skeletal sites from TB-DXA could be used to estimate the extent to which genetic and environmental factors influence bone mass accrual of differentially loaded skeletal sites (skull, lower limbs, and upper limbs). Point estimates indicated that common SNPs on a commercially available genotyping array, explained a larger proportion of the overall variance of SK-BMD, when compared to BMD measured at the appendicular sites (i.e. lower and upper limbs). These differences potentially reflect differential exposure of each skeletal site to varying environmental stimuli that influence BMD. Specifically the skull, as opposed to appendicular sites, is less influenced by environmental factors, particularly those acting through mechanical loading. To explore this result further, we estimated the residual correlation (i.e. the proportion of environmental and other sources of variation not tagged by SNPs on the Illumina platform) across the different skeletal sites and found that whilst the environmental (and other residual) factors influencing the appendicular sites were moderately similar to each other, they appeared to be appreciably different from the factors influencing SK-BMD. Taken together, lower v_g_ estimates, coupled with a high residual correlation between the two appendicular sites, may reflect the greater exposure of these sites to loading and muscular stimulation, when compared to the skull.

Likewise, estimates of the genetic correlations indicated that the appendicular limbs shared a more similar genetic architecture when compared to the skull, possibly reflecting the composition of bone at each skeletal site and the biological processes that govern their growth and maintenance. For example, appendicular sites consist of broadly equivalent proportions of cortical and trabecular bone. The skull on the other hand is mainly comprised of flat bones, which consist primarily of cortical bone [18]. The developmental processes also differ between long and flat bones, with dermal bones such as the skull vault arising exclusively through intramembranous bone formation, in contrast to long bones, which form through endochondral bone formation involving intermediary formation of cartilage [19].

To further explore the basis for the above-mentioned differences in underlying genetic architecture, GWA meta-analyses of sub-regional TB-DXA data were performed. These analyses helped identifying genetic signals that were associated with one or more skeletal region(s). When comparing the evidence of association for all SNPs (identified in this effort) across each skeletal site, our GWA meta-analyses echoed the findings of our GCTA results, supporting the notion that although the underlying genetic architecture influencing BMD appears to be largely similar, it does vary according to skeletal site. The majority of the top SNPs were nominally associated (*P*≤0.05) with BMD across all skeletal sites (i.e. SNPs at *WNT4*, *WNT16, FAM3C*, *GALNT3*, *FUBP3*, *KLHDC5/PTHLH*, *TNSF11*, *LIN7C/LGR4* and *PPP6R3/LRP5*). In contrast, variants near or within *CPED1*, *COLEC10/TNFRS11B* and *EYA4* were strongly associated with UL- and SK-BMD, but not LL-BMD. A further variant was identified within *TNFRSF11A* that appeared to be solely related to SK-BMD. Most notably we observed a novel association between rs754388 (located within *RIN3*) and LL-/UL-BMD, but not SK-BMD. To the best of our knowledge this is the first GWAS to report an association between *RIN3* and BMD. It seems likely that this association reflects a true relationship with BMD as the same *RIN3* signal (as determined by conditional analysis) has previously been associated with an increased risk of Paget's Disease [i.e. rs10498635-C OR: 1.44, 95%-CI (1.29–1.60) *P* = 3.×10^−11^] [20].

In an attempt to further understand how the genetic variation surrounding *RIN3* may influence BMD, we fine mapped *RIN3* and identified a missense variant (rs117068593) that was in high LD with our LL-BMD associated SNP. Data mining of SIFT and ENCODE databases suggested a functional role of the missense variant that putatively affects binding of several transcription factors in lymphoblastic cell lines. We further evaluated expression quantitative trait locus (eQTL) data from primary human osteoblasts using SNP data from HapMap (i.e. not including rs117068593) and found no substantial evidence that our LL-BMD associated SNPs located at 14q32.12 regulated the expression of *RIN3* or any of the genes located nearby. However, differential patterns gene expression were detected when comparing *RIN3* expression profiles of osteoporotic and healthy individuals. Further, we also observed differential expression during osteoclast differentiation that was not present in osteoblast and adipocyte differentiation processes. Collectively, the aforementioned observations appear to be in line with previous findings that suggest that RIN3 could influence osteoclast activity, especially when considering the prior association of *RIN3* with Paget's Disease, a disease driven by osteoclast dysfunction and molecular studies that indicate that RIN3 is involved in vesicular trafficking, a process critical for bone resorption [20], [21]. Further study is however needed to elucidate the precise role of role of RIN3 in bone metabolism.

To further understand the preferential associations of some variants with different skeletal sites, we compared the standardized effect sizes of all the genome-wide significant BMD-associated variants, across each skeletal site using a formal multivariate normal likelihood model. Variants at the *CPED1* locus were strongly associated with BMD at the skull and upper limb sites, but not with LL-BMD. Similarly variants at *WNT16* were more strongly related to UL-BMD, than to BMD at the other sites. Several other SNPs showed evidence for site specificity including variants at the *EYA4* and *LIN7C* loci that were very strongly related to SK-BMD, although these variants did not surpass our conservative criterion for declaring significant heterogeneity, corroboration is needed from independent studies.

Conceivably, differences in the pattern of results across SNPs may have arisen from an artefact of the measurement (i.e. where sub-regional-specific associations reflect how accurately BMD is measured at each skeletal site). However, if the latter were the case, one would expect to observe a consistent pattern of results across all loci (i.e. the strength of association should be greatest at those sites measured more accurately). From our results, this is clearly not the case as evidence of association is sometimes greatest for the skull, whilst for other SNPs evidence is greatest for lower and/or upper limbs. In terms of biological explanations, larger effect sizes of genetic variants that influence SK-BMD possibly reflect their preferential involvement in cortical as opposed to trabecular bone metabolism and/or the involvement of intramembranous ossification vs. endochondral ossification [13]. Certain genetic factors also appeared to influence UL-BMD more strongly than LL-BMD, or vice versa. Since the composition and developmental origin of these two sites is broadly similar, presumably, other explanations are responsible. It is reasonable to think that genetic factors, which we identified, could be acting to alter responses to stimuli that are themselves site-specific. For example, adipose tissue has previously been reported to influence cortical bone of the tibia in preference to the radius [22].

Quantitative SK-BMD measurements have traditionally been ignored by genetic and epidemiological studies as they are thought to be prone to errors such as dental augmentation. Despite these concerns, a study conducted in premenopausal woman found a high correlation between the upper half of the skull (i.e. cranial vault) and total skull-BMD (r^2^ = 0.991, n = 91, Age range 19–30 years), with a mean difference of −0.004 g/cm2, suggesting that these two measurements of bone mass are similar [23]. We found that paediatric SK-BMD measures are well suited to GWAS, as indicated by the very low *P*-values obtained at some of the known BMD associated loci (10^−17^ to 10^−28^) despite our relatively small sample size. This observation may reflect the fact that SK-BMD is considerably less subject to environmental influences, such as those acting through mechanical loading. In addition, genetic variants associated with SK-BMD identified in this study may primarily reflect molecular pathways involved in bone mass accrual and growth, in contrast to variants identified from previous adult scans which may be more strongly related to mechanisms involved in bone maintenance and/or loss.

Almost all the loci we have identified in this study (i.e. with the exception of SNPs in *RIN3* and *EYA4*) have been associated with BMD at either the hip or the lumbar spine previously. Variants mapping to *RIN3* have been implicated in Paget's disease but this is the first time the locus is associated with BMD, and interestingly, the alleles associated with increased BMD are associated with increased risk for the condition. This shows that performing GWAS of BMD at sites other than at the femoral neck (FN-BMD) or lumbar spine (LS-BMD) can be used to identify loci that exert pleiotropic effects on bone. Potential advantages of examining these additional sites from a locus discovery perspective are that (i) genetic variants may exert stronger effects at these sites than at FN-BMD/LS-BMD, and/or (ii) the genetic effects may be more apparent at these sites because the effect of environmental noise is minimized. For example, the *P*-values for skull BMD at several loci (e.g. variants around *CPED1*, *EYA4* and *LIN7C*) are many orders of magnitude stronger than the corresponding *P*-values for TBLH-BMD (see Table S8). Likewise variants in *LIN7C* were first discovered using a GWAS meta-analysis of lumbar spine that was over five times the size of the present study, and even then only just exceeded the threshold for genome-wide significance [4], whereas in our study a variant at this locus has *P*<1×10^−16^ with SK-BMD. Hence, GWAS of BMD at sites such as the skull could be used to efficiently detect clinically relevant loci that might be more difficult to discover in GWAS of the femoral neck and/or lumbar spine.

To further illustrate the value of SK-BMD, we draw attention to rs3012465, a variant proximal to the eyes absent (*EYA4*) gene and associated with increased SK-BMD. We show that the signal is analogous to that previously associated with increased volumetric cortical BMD of the tibia (i.e. C allele of rs271170: *β* = 0.11, *P* = 2.7×10^−12^), based on a GWAS in ALSPAC and other young adult cohorts [13], suggesting that both findings reflect the relationship of the *EYA4* locus with cortical bone. However, a look-up in a separate cortical bone site (i.e. the femoral neck of the hip), from a GWAS in older adults, revealed that the BMD-increasing allele at the *EYA4* locus was in fact associated with lower BMD for both rs3012465 and rs271170 [4]. Taken together, these findings may reflect an age dependent effect of *EYA4* whereby *EYA4* contributes to bone accrual in early life, yet maybe influences bone loss in older adults. To test this hypothesis, we followed up these *EYA4* variants in a recent GWAS meta-analysis of FN-BMD in 4061 pre-menopausal women aged 20–45 (as described in Koller and colleagues [15]) and failed to find any evidence of association with FN-BMD (*P* = 0.73). These results suggest that the discrepancy in results between GEFOS and the present study is unlikely to be solely due to age, but rather is likely to represent a real difference between skeletal sites.

In summary, our strategy of analysing regional paediatric DXA measures of TB-BMD represents a novel approach to dissecting the genetic architecture influencing bone mass accrual and growth at different skeletal sites. Specifically, variants at 13 loci reached genome-wide significance with BMD and several displayed different degrees of association according to skeletal site. Furthermore, we report a novel association between a variant within *RIN3* and LL-BMD and note its previous association with risk of Paget's disease. We additionally provide suggestive evidence of allelic heterogeneity at the *CENPW/RSPO3*, *KLHDC5/PTHLH* and *LIN7C/LGR4* loci. In conclusion our results provide evidence that different skeletal sites as measured by TB-DXA are to a certain extent under distinct environmental and genetic influences. Allowing for these differences may help to uncover new genetic influences on BMD, particularly those examined in children as involved in bone growth and accrual.

## Materials and Methods

### Subjects

#### ALSPAC

ALSPAC is a longitudinal population-based birth cohort that recruited pregnant women residing in the former county of Avon, UK, with an expected delivery date between 1^st^ April 1991 and 31^st^ December 1992. This cohort has been described in detail on the website (http://www.alspac.bris.ac.uk) and elsewhere [24]. DXA, height and weight measurements were performed on children who attended the 9 year old focus group clinic [mean age of participant 9 (±0.32 years)]. Ethical approval was obtained from the ALSPAC Law and Ethics committee and relevant local ethics committees, and all parents provided written informed consent.

#### Generation R Study

The Generation R Study is a prospective cohort study enrolling 9,778 pregnant women living in Rotterdam with a delivery date from April 2002 until January 2006. Details of study design and data collection have been described elsewhere [25]. DXA, height and weight measurements were performed on children who visited the research centre whilst being accompanied by their mothers at a mean age of 6 (±0.5 years). All research aims and specific measurements taken during the course of the Generation R Study have been approved by the Medical Ethical Committee of the Erasmus Medical Center, Rotterdam. All parents provided written informed consent.

### Phenotypes

#### ALSPAC

TB-DXA scans were performed on all participants, using a Lunar Prodigy scanner (Lunar Radiation Corp, Madison, WI) with paediatric scanning software (GE Healthcare Bio-Sciences Corp., Piscataway, NJ). DXA measures of BMD were derived for the following regions of interest: TBLH-, UL-, LL- and S. All DXA scans were subsequently reviewed by a trained researcher, and re-analysed as necessary, to ensure that borders between adjacent ROI's were placed correctly by the automated software. The coefficient of variation for TBLH-BMD measures was 0.8%, based on the analysis of 122 children who had two scans performed on the same day. Height was measured to the nearest 0.1 cm using a Harpenden stadiometer (Holtain Ltd., Crymych, UK) and weight was measured to the nearest 50 g using Tanita weighing scales (Tanita UK Ltd, Uxbridge).

#### Generation R Study

TB-BMD was measured in all participants using a GE-Lunar iDXA scanner. Well-trained research assistants obtained the DXA scans using the same device and software (enCORE) following standard manufacturer protocols. The same regions of interest as described for ALSPAC were derived from TB-DXA scan. To ensure that the lines between adjacent ROI's were placed correctly by the automated software, scans were evaluated twice, directly after the scanning and at a later time point by a second well-trained research assistant. The coefficient of variation for total TBLH-BMD measures was 0.23%, based on duplicate scans of children that were performed on the same day.

### Genotyping and imputation

#### ALSPAC

A total of 9,912 subjects were genotyped using the Illumina HumanHap550 quad genome-wide SNP genotyping platform (Illumina Inc., San Diego, CA, USA) by 23andMe subcontracting the Wellcome Trust Sanger Institute, Cambridge, UK and the Laboratory Corporation of America (LabCorp Holdings., Burlington, NC, USA). PLINK software (v1.07) was used to carry out quality control measures [26]. Individuals were excluded from further analysis on the basis of having incorrect gender assignments, minimal or excessive heterozygosity (<0.320 and >0.345 for the Sanger data and <0.310 and >0.330 for the LabCorp data), disproportionate levels of individual missingness (>3%), evidence of cryptic relatedness (>10% IBD) and being of non-European ancestry (as detected by a multidimensional scaling analysis seeded with HapMap 2 individuals). EIGENSTRAT analysis revealed no additional obvious population stratification and genome-wide analyses with other phenotypes indicate a low lambda) [27]. SNPs with a minor allele frequency of <1% and call rate of <95% were removed. Furthermore, only SNPs that passed an exact test of Hardy–Weinberg equilibrium (P>5×10^−7^) were considered for analysis. After quality control, 8,365 unrelated individuals who were genotyped at 500,527 SNPs were available for analysis. Known autosomal variants were imputed with Markov Chain Haplotyping software (MACH 1.0.16) [28], [29], using CEPH individuals from phase II of the HapMap project (hg18) as a reference set (release 22) [30]. The BMD associated *RIN3* locus was further imputed using the complete reference panel from the third phase of the 1000 Genomes Project (i.e. March 2012) [31].

#### Generation R Study

Genotyping was performed using the Illumina HumanHap 610 QUAD microarray using standard manufacturer protocols. Stringent quality control of the genotype and imputation process was performed in this study as previously described [7]. Samples with gender discrepancy, excess of heterozygosity, low genotype quality and sample replicates were excluded from the analysis. A reference panel for imputation, consisting of CEPH, YRI and CHB/JPT haplotypes was constructed using data from phase 2 of the HapMap project (hg18, release 22) [30]. A two-step imputation process was performed using MACH for haplotype phasing and Minimac for imputation [28], [29]. A similar 1000 Genomes imputation strategy (as described above for ALSPAC) was used to fine map the *RIN3* locus.

### Statistical methods

#### Choice of covariates

BMD as measured by DXA is strongly influenced by weight, in part because weight is related to skeletal size. BMD as assessed by DXA, does not correct for the thickness (depth) of bone, therefore true (volumetric) bone mineral density is often underestimated in smaller individuals and overestimated in larger subjects. Weight is also thought to affect BMD by other pathways such as increased skeletal loading, and possibly by other metabolic influences. We reasoned that SK-BMD is likely to be relatively unaffected by these other pathways, and so whereas TBLH-, UL- and LL-BMD measures were adjusted for weight, SK-BMD was adjusted for height.

#### Genome-wide complex trait analysis

Univariate restricted maximum likelihood (REML) genome-wide complex trait analyses (GCTA) [32] were performed on ≥4,866 ALSPAC subjects to estimate the proportion of additive genetic variance in BMD at each site, explained by directly genotyped variants that had a minor allele frequency ≥1%. Bivariate REML GCTA analysis [33] was further used to estimate the pair-wise genetic and residual correlations between BMD at each skeletal site. A cryptic relatedness cut-off of 0.025 was applied in order to ensure that distantly related individuals (i.e. n∼444) were removed prior to the analysis, thereby reducing the potential for bias (Figure S15). GCTA analysis was not performed in the Generation R Study given its multi-ethnic composition. Pearson Product Moment Correlation was used to estimate the linear relationship between standardised residuals of BMD after adjusting for age, gender, and weight or height using the STATA statistical package [34].

#### Genome-wide association meta-analysis of BMD in ALSPAC and Generation R

To identify genetic loci influencing variation in TBLH-, LL-, UL- and SK-BMD, we performed GWAS meta-analyses combining 5,330 children (5,299 for SK-BMD) from the ALSPAC cohort and 4,086 children from the Generation R Study, who had DXA BMD measurements and imputed GWAS data. Cohort specific GWAS analyses were conducted in ALSPAC and Generation R using standardised residuals derived from BMD measures after adjustment for age, gender and weight for all skeletal sites except the skull, where weight was substituted for height. The first 20 ancestry informative principal components were additionally incorporated into the Generation R model to control for population stratification, due to the multi-ethnic nature of this cohort as described previously [7]. Genome-wide association analyses were performed using MACH2QTL [29] as implemented in GRIMP [35], using linear regression models based on an expected allelic dosage for SNPs, adjusting for the above mentioned covariates where necessary. We combined association data for ∼2.5 million imputed autosomal SNPs into an inverse variance fixed-effects meta-analysis, using METAL and controlled for genomic inflation in each cohort [36]. P-values less than 5×10^−8^ were considered genome-wide significant. Heterogeneity was evaluated using Cochran's Q statistic and was quantified by the I^2^ metric. Regional association plots from our genome-wide association scans were generated using Locuszoom (v1.1) [37], using linkage-disequilibrium (LD) information estimated from the HapMap 2 (hg18) CEPH reference dataset [38]. All pair-wise LD estimates were obtained using SNAP software in conjunction with the HapMap2Phase II (hg18) CEPH reference dataset [39]. All remaining plots were generated in R [40] using the ggplot2 software package [41].

#### Sensitivity analysis

In order to test the robustness of our results to the choice of covariates at each site, we performed a sensitivity analysis adjusting each region-specific BMD measure for: age, gender, height and weight (i.e. Model 1a) and performing GCTA and GWAS meta-analysis using the residuals. In order to confirm that our results were not being driven by underlying population substructure, we performed further GWAS meta-analyses using the same residuals derived for Model 1a, except that the analyses were restricted to individuals of European ancestry (i.e. Model 1b).

#### Conditional meta-analysis

To identify novel secondary BMD association signals (i.e. independent from those published), at loci which reached genome-wide significance for each BMD meta-analysis, we carried out conditional meta-analyses by conditioning on all previously published BMD-associated variants that mapped to between 1–2 Mb of the top locus specific SNP [depending on the extend to LD, (Table S12)]. In the case of rs7851693 (*FUBP3*), not present in the Generation R dataset, conditional analysis was performed using a proxy SNP (i.e. rs7030440), which was in high LD (r^2^>0.96) with the missing BMD associated variant. For *RIN3*, there were no BMD associated loci previously published, thus for that locus we conditioned on the top SNP. After conditional analysis, locus specific significance correction thresholds (for multiple testing of SNPs which are in linkage disequilibrium with each other) were calculated using the Single Nucleotide Polymorphism Spectral Decomposition (SNPSpD) software package [42]. Locus specific regions used for SNPSpD were defined as the region proximal (1–2 Mb) to each locus specific signal. The presence of independent secondary association signals was confirmed in situations where the residual signal (after conditional meta-analysis) reached the locus specific threshold corrected for multiple testing.

#### Comparison of the magnitude of the effect sizes of genome-wide significant SNPs across skeletal-sites

As we were interested in whether the genetic variants exerted greater influence on BMD at a particular site, it would be misleading to directly compare standardized regression coefficients from the meta-analysis by e.g. simple Z test, since the summary statistics were derived from correlated measures on the same individuals and are therefore not independent. In addition, because we are only testing variants that have already met the criterion for genome-wide significance (and were therefore selected on the basis of having an extreme regression coefficient at a particular site), it is not appropriate to compare the regression coefficients from different sites using an uncorrected type I error level of *α* = 0.05. To address both these considerations, we fitted a multivariate normal model to the standardized BMD scores at each site by maximum likelihood using the software package Mx [43]. We fitted a model where the standardized regression coefficients for each site (SK-BMD, LL-BMD, UL-BMD) were constrained to be equal, and then another model in which the regression coefficient most different from the other two was allowed to vary). Twice the difference in log-likelihood between these models is distributed as a chi-square statistic with one degree of freedom. We analysed each cohort separately using this method and then combined the results using Fisher's product of *P*-values evaluating statistical significance against a conservative threshold of *α* = 5×10^−8^ (i.e. as if we had performed the comparison genome-wide, not just post-hoc on the significant sites).

### Functional analysis of *RIN3*

In an attempt to identify a potential functional or regulatory mechanism underlying the association between *RIN3* and BMD, a range of bio-informatic and functional analyses were performed. These included: fine mapping the *RIN3* locus, data mining Regulome [17] and SIFT [16] databases and performing eQTL analysis on primary human osteoblasts. The expression profiles of *RIN3* and neighboring genes: *SLC2484, LGMN, GOLGA5, CHGA* and *ITPK1* were also investigated in bone biopsies of healthy and osteoporotic women, in addition to murine and human cell lines that were differentiated into osteoblasts and/or osteoclasts. Methods specific to each analysis are described below.

#### Human primary osteoblasts

Expression profiling of untreated primary human osteoblasts, obtained from 113 (51 female and 62 male) unrelated Swedish donors, was performed using the Illumina HumRef-8 BeadChips in accordance with the manufacturer's instructions. Up to 3 biological replicates were analysed per sample. Genotyping for genotype-expression association was performed using the Illumina HapMap 550 k Duo chip. Individuals with low genotyping rate and SNPs showing significant deviation from Hardy-Weinberg equilibrium (*P*<0.05) were excluded. Similarly, low frequency (MAF<0.05) SNPs and SNPs with high rates of missing data were excluded. Genotypes from samples that passed quality control (n = 103) were imputed for all SNPs (n = 478,805) oriented to the positive strand from phased autosomal chromosomes of HapMap Phase 2 CEPH panel (release 22, build 36) using MACH 1.0. A RSQR cut-off of <0.3 was used to remove poorly imputed markers. Association of imputed genotypes using estimated genotype probabilities with nearby expression traits (defined as ±1 Mb window flanking *RIN3*) was performed using a linear regression model implemented in the MACH2QTL software with sex and age as covariates. Detailed methods pertaining to the data generation and analysis are described elsewhere [44], [45].

#### Human illiac bone biopsies

Gene expression profiles were generated from iliac bone biopsies donated by healthy control (n = 27), osteopenic (n = 18) and osteoporotic (n = 39) postmenopausal Norwegian women. The affection status of each individual was determined by BMD measurements of the total hip or lumbar spine (L1–L4 vertebrae). Individuals with a T-score less than −2.5 and with at least one low trauma fracture were deemed osteoporotic, whilst individuals with a T-score >−1 were deemed healthy. Expression profiling was performed using an Affymetrix HG U133 2.0 plus array. The Affymetrix Cel files were imported into Partek Genomics Suite (Partek Inc., St Louis, MO, USA), and normalized using the RMA (Robust Multichip Average) algorithm. Gene expression patterns were further adjusted, as reported by Jemtland and colleagues [46], for batch effects and differing synthesis times. The gene expression profiles of all transcripts located ±250 kb of the top LL-BMD associated *RIN3* SNP (i.e. rs754388) were compared between the osteoperotic and control group. Note: the intermediate osteopenic group was excluded from this analysis.

#### Murine pre-osteoblasts

All procedures and use of mice for the neonatal osteoblast expression studies were approved by the Jackson Laboratory Animal Care and Use Committee (ACUC), in accordance with NIH guidelines for the care and use of laboratory animals. Pre-osteoblast-like cells were isolated from neonatal calvaria from C57BL/6J mice expressing cyan florescent protein (CFP) under the control of the Col3.6 promoter (pOBCol3.6CFP), using standard techniques [47]. Cells were cultured for 4 days in growth media [DMEM containing 10% fetal bovine serum (FBS) and 1× penicillin/streptomycin], and thereafter removed from culture and subjected to fluorescence-activated cell sorting (FACS) based on the presence/absence of CFP expression. Cells expressing CFP, and therefore considered pre-osteoblasts, were plated at a density of 1×10^4^ cells per cm^2^, differentiated into osteoblasts using standard methods (αMEM containing 50 µg/ml Ascorbic Acid, 4 mM *β*-glycerol phosphate, 10% FBS and 1× penicillin/streptomycin). RNA was collected at 9 time points post differentiation. mRNA profiles for triplicate samples for each time point were generated by Next Generation High throughput RNA sequencing (RNAseq), using an Illumina HiSeq 2000. The alignments for abundance estimation of transcripts was conducted using Bowtie version 0.12.9 [48] using the NCBIm37 transcriptome as the reference genome. Expression level per gene was calculated using RSEM version 1.2.0 using the following parameters: –fragment-length-mean 280 and –fragment-length-sd 50 [49] and expression level for each sample was normalized relative to the per sample upper quartile [50]. This data has been submitted to the gene expression omnibus (Accession Number: GSE54461).

#### Human mesenchymal stem cells and peripheral blood mononuclear cells

Human bone marrow derived mesenchymal stem cells [(hMSC), Lonza Group Ltd., Basel, Switzerland] were seeded in 12-well plates (5×10^3^ cells per cm^2^) and differentiated into osteoblasts (using *α*-Mem pH 7.5, 10% heat inactivated foetal calf serum (FCS), 100 nM Dexamethasone and 10 mM *β*-glycerophosphate) or adipocytes (using *α*-MEM pH 7.5, 10% heat inactivated FCS, 100 nM dexamethasone, 500 µM IBMX and 60 µM Indomethacin). Total RNA was isolated (triplicates) using Trizol (Life Technologies, Carlsbad, CA, USA) twice a week during differentiation until the 25 day of culture. Human peripheral blood mononuclear cells (PBMCs) were retrieved from buffy coats using Ficoll and seeded in 12-well plates (1.3×10^5^ cells per cm^2^). Monocytes were allowed to attach for 4 hours and non-adherent cells were removed by careful washing. In the next three days, cells were grown in *α*-Mem pH 7.5, containing 15% heat-inactivated serum and 25 ng/ml macrophage colony stimulating factor [(M-CSF), R&D Systems Inc., Minneapolis, MN, USA] to stimulate proliferation of the monocytes. After 3 days, media was replaced with *α*-Mem pH 7.5 containing 15% heat inactivated serum, 25 ng/ml M-CSF and 30 ng/ml RANKL (Peprotech Inc., Rocky Hill, NJ, USA) to initiate osteoclastogenesis. Total-RNA was isolated twice a week using Trizol until the 21^st^ day of culture. Amplification of total-RNA, Illumina microarray hybridization, data extraction and normalization were performed as previously described [51].

## Supporting Information

Figure S1 Genome-wide association meta-analysis of age-, gender-, height- or weight-adjusted BMD measured at four different skeletal sites. Manhattan and Q-Q plots derived from the genome-wide association meta-analysis of BMD measures of the total-body less head (TBLH), lower limb (LL), upper limb (UL) and skull (SK). The names of the closest genes relative to the each locus specific top SNP are indicated in blue. Q-Q plots show the inflation of the test statistics (λMETA) of each genome-wide association meta-analysis. *Please note that *PTHLH* is also located at the 12p11.22 locus containing *KLHDC5, RSPO3* is also located at the 6q.22.32 locus containing *CENPW, FAM3C and CPED1* are also located at the 7q.31.31 locus containing *WNT16*, *TNFRSF11B* is also located at the 8q.24.12 locus containing *COLEC10, LGR4* is also located at the 11p14.1 locus containing *LIN7C* and *LRP5* is also located at the 11q13.2 locus containing *PPP6R3*.
(TIF)

Figure S2 Regional association plots for all loci which reached genome-wide significance for TBLH-BMD before and after conditioning on known BMD associated SNPs. Circles show GWA meta-analysis *P*-values and positions of SNPs found within each locus. The top SNP are denoted by diamonds. Different colours indicate varying degrees of pair wise linkage disequilibrium estimates between the top SNP and all other SNPs. *Please note that *PTHLH* is also located at the 12p11.22 locus containing *KLHDC5, RSPO3* is also located at the 6q.22.32 locus containing *CENPW, FAM3C and CPED1* are also located at the 7q.31.31 locus containing *WNT16*, *TNFRSF11B* is also located at the 8q.24.12 locus containing *COLEC10, LGR4* is also located at the 11p14.1 locus containing *LIN7C* and *LRP5* is also located at the 11q13.2 locus containing *PPP6R3*.
(TIF)

Figure S3 Regional association plots for all loci which reached genome-wide significance for LL-BMD before and after conditioning on known BMD associated SNPs. Circles show GWA meta-analysis *P*-values and positions of SNPs found within each locus. The top SNP are denoted by diamonds. Different colours indicate varying degrees of pair wise linkage disequilibrium estimates between the top SNP and all other SNPs. *Please note that *PTHLH* is also located at the 12p11.22 locus containing *KLHDC5, RSPO3* is also located at the 6q.22.32 locus containing *CENPW, FAM3C and CPED1* are also located at the 7q.31.31 locus containing *WNT16*, *TNFRSF11B* is also located at the 8q.24.12 locus containing *COLEC10, LGR4* is also located at the 11p14.1 locus containing *LIN7C* and *LRP5* is also located at the 11q13.2 locus containing *PPP6R3*.
(TIF)

Figure S4 Regional association plots for all loci which reached genome-wide significance for UL-BMD before and after conditioning on known BMD associated SNPs. Circles show GWA meta-analysis *P*-values and positions of SNPs found within each locus. The top SNP are denoted by diamonds. Different colours indicate varying degrees of pair wise linkage disequilibrium estimates between the top SNP and all other SNPs. *Please note that *PTHLH* is also located at the 12p11.22 locus containing *KLHDC5, RSPO3* is also located at the 6q.22.32 locus containing *CENPW, FAM3C and CPED1* are also located at the 7q.31.31 locus containing *WNT16*, *TNFRSF11B* is also located at the 8q.24.12 locus containing *COLEC10, LGR4* is also located at the 11p14.1 locus containing *LIN7C* and *LRP5* is also located at the 11q13.2 locus containing *PPP6R3*.
(TIF)

Figure S5 Regional association plots for all loci which reached genome-wide significance for SK-BMD before and after conditioning on known BMD associated SNPs. Circles show GWA meta-analysis *P*-values and positions of SNPs found within each locus. The top SNP are denoted by diamonds. Different colours indicate varying degrees of pair wise linkage disequilibrium estimates between the top SNP and all other SNPs.*Please note that *PTHLH* is also located at the 12p11.22 locus containing *KLHDC5, RSPO3* is also located at the 6q.22.32 locus containing *CENPW, FAM3C and CPED1* are also located at the 7q.31.31 locus containing *WNT16*, *TNFRSF11B* is also located at the 8q.24.12 locus containing *COLEC10, LGR4* is also located at the 11p14.1 locus containing *LIN7C* and *LRP5* is also located at the 11q13.2 locus containing *PPP6R3*.
(TIF)

Figure S6 Genome-wide association meta-analysis of age-, gender-, height- and weight-adjusted BMD measured at four different skeletal sites. Manhattan and Q-Q plots derived from the genome-wide association meta-analysis of BMD measures of the total-body less head (TBLH), lower limb (LL), upper limb (UL) and skull (SK). The names of the closest genes relative to the each locus specific top SNP are indicated in blue. Q-Q plots show the inflation of the test statistics (λMETA) of each genome-wide association meta-analysis. *Please note that *PTHLH* is also located at the 12p11.22 locus containing *KLHDC5, RSPO3* is also located at the 6q.22.32 locus containing *CENPW, FAM3C and CPED1* are also located at the 7q.31.31 locus containing *WNT16*, *TNFRSF11B* is also located at the 8q.24.12 locus containing *COLEC10, LGR4* is also located at the 11p14.1 locus containing *LIN7C* and *LRP5* is also located at the 11q13.2 locus containing *PPP6R3*.
(TIF)

Figure S7 Genome-wide association meta-analysis of age-, gender-, height- and weight-adjusted BMD measured at four different skeletal sites in individuals of European ancestry. Manhattan and Q-Q plots derived from the genome-wide association meta-analysis of BMD measures of the total-body less head (TBLH), lower limb (LL), upper limb (UL) and skull (SK). The names of the closest genes relative to the each locus specific top SNP are indicated in blue. Q-Q plots show the inflation of the test statistics (λMETA) of each genome-wide association meta-analysis. *Please note that *PTHLH* is also located at the 12p11.22 locus containing *KLHDC5, RSPO3* is also located at the 6q.22.32 locus containing *CENPW, FAM3C and CPED1* are also located at the 7q.31.31 locus containing *WNT16*, *TNFRSF11B* is also located at the 8q.24.12 locus containing *COLEC10, LGR4* is also located at the 11p14.1 locus containing *LIN7C* and *LRP5* is also located at the 11q13.2 locus containing *PPP6R3*.
(TIF)

Figure S8 Comparison of effect sizes of the top SK-BMD associated variants across each skeletal site. The per allele effect in SD (red dot) and 95% confidence interval (error bar) of the top SNP associated with BMD measurements of the lower limb (LL), upper limb (UL) and skull (SK) are plotted with their specific strength of association. *Please note that *PTHLH* is also located at the 12p11.22 locus containing *KLHDC5, RSPO3* is also located at the 6q.22.32 locus containing *CENPW, FAM3C and CPED1* are also located at the 7q.31.31 locus containing *WNT16*, *TNFRSF11B* is also located at the 8q.24.12 locus containing *COLEC10, LGR4* is also located at the 11p14.1 locus containing *LIN7C* and *LRP5* is also located at the 11q13.2 locus containing *PPP6R3*.
(TIF)

Figure S9 Comparison of effect sizes of the top UL-BMD associated variants across each skeletal site. The per allele effect in SD (red dot) and 95% confidence interval (error bar) of the top SNP associated with BMD measurements of the lower limb (LL), upper limb (UL) and skull (SK) are plotted with their specific strength of association. *Please note that *PTHLH* is also located at the 12p11.22 locus containing *KLHDC5, RSPO3* is also located at the 6q.22.32 locus containing *CENPW, FAM3C and CPED1* are also located at the 7q.31.31 locus containing *WNT16*, *TNFRSF11B* is also located at the 8q.24.12 locus containing *COLEC10, LGR4* is also located at the 11p14.1 locus containing *LIN7C* and *LRP5* is also located at the 11q13.2 locus containing *PPP6R3*.
(TIF)

Figure S10 Comparison of effect sizes of the top LL-BMD associated variants across each skeletal site. The per allele effect in SD (red dot) and 95% confidence interval (error bar) of the top SNP associated with BMD measurements of the lower limb (LL), upper limb (UL) and skull (SK) are plotted with their specific strength of association. *Please note that *PTHLH* is also located at the 12p11.22 locus containing *KLHDC5, RSPO3* is also located at the 6q.22.32 locus containing *CENPW, FAM3C and CPED1* are also located at the 7q.31.31 locus containing *WNT16*, *TNFRSF11B* is also located at the 8q.24.12 locus containing *COLEC10, LGR4* is also located at the 11p14.1 locus containing *LIN7C* and *LRP5* is also located at the 11q13.2 locus containing *PPP6R3*.
(TIF)

Figure S11 Comparison of effect sizes of the top TBLH-BMD associated variants across each skeletal site. The per allele effect in SD (red dot) and 95% confidence interval (error bar) of the top SNP associated with BMD measurements of the lower limb (LL), upper limb (UL) and skull (SK) are plotted with their specific strength of association. *Please note that *PTHLH* is also located at the 12p11.22 locus containing *KLHDC5, RSPO3* is also located at the 6q.22.32 locus containing *CENPW, FAM3C and CPED1* are also located at the 7q.31.31 locus containing *WNT16*, *TNFRSF11B* is also located at the 8q.24.12 locus containing *COLEC10, LGR4* is also located at the 11p14.1 locus containing *LIN7C* and *LRP5* is also located at the 11q13.2 locus containing *PPP6R3*.
(TIF)

Figure S12 Gene expression profiles of *Rin3, Golga5 Lgmn, and Itpk1* measured throughout the osteoblast maturation process in cells extracted from mouse calvariae, as measured by RNAseq. Samples for expression purposes were collected every other day for 18 days, starting 2 days after the cells were first exposed to an osteoblast differentiation cocktail. Relative transcript abundance is expressed as the number of query transcripts per million unique transcripts (transcripts per million), after normalizing to the upper quartile. A local weighted scatterplot smoothing curve was plotted to help with visualizing the expression pattern. Note: *Slc24a4* and *Chgm* were not expressed in this cell type and have not been included in the figure.
(TIF)

Figure S13 Gene expression profile of *RIN3, LGMN, GOLGA5 and ITPK1* measured in osteoclast differentiating human PBMCs. Relative transcript abundance is expressed as Log2 normalized intensities. Each value is an average of 3 independent measurements and a standard deviation.
(TIF)

Figure S14 Gene expression profile of *RIN3, LGMN, GOLGA5 and ITPK1* measured in adipogenic and osteogenic differentiating hMSCs. Relative transcript abundance is expressed as Log2 normalized intensities. *RIN3* expression levels in differentiating hMSC are absent because the intensities were at background level. Each value is an average of 3 independent measurements and a standard deviation.
(TIF)

Figure S15 Flow diagram and overview of the analysis strategy used in this study. For ALSPAC, a total of 9,912 subjects were genotyped by Wellcome Trust Sanger Institute, Cambridge and the Laboratory Corporation of America. Individuals were excluded from further analysis using several quality control (QC) criteria (See methods). After merging and further QC, 8,365 unrelated subjects [identity by decent (IBD) <10% and of European ancestry] were available for GWAS analysis. Total-body DXA scans were performed on 7725 subjects that attended the Focus 9 clinic. Of these a total of 6540 passed DXA QC. For total body (TB), lower limb- (LL) and upper limb (UL) GWAS analysis 5,330 subjects had high quality bone mineral density (BMD) and genetic data, whereas 5,229 subjects were available for skull (S). For GCTA analysis, we employed a strict threshold of genome-wide identity by state >2.5% and resulting in the exclusion of additional individuals on the basis of cryptic relatedness. 4,891 (TB-, LL- and UL-BMD) and 4,866 (S-BMD) subjects were available for GCTA analysis. For Generation R study a total of 5,908 subjects were genotyped by the Erasmus Medical Centre. Following QC 5,756 individuals had high quality genotyping data. Total-body DXA scans were performed on 6,509 subjects, of these a total of 6,490 passed DXA QC. High quality BMD and genetic data was available for 4,086 subjects. Of these 2,177 subjects were of Dutch-European decent. Two GWAS meta-analysis strategies were performed for each site. The first strategy involved all the subjects in the ALSPAC and the Generation-R studies. The second approach involved all the ALSPAC subjects, but was restricted to Generation R subjects who were of European descent. The number of subjects (n) involved in each step of the analysis is indicated. ^Ŧ^ = Number of subjects that had S-BMD measurements that passed QC. ^Φ^ = Number of Generation R subjects that were of Dutch-European descent.
(TIF)

Table S1 Bivariate GCTA estimates of the genetic and residual correlations of age-, gender-, height- and weight-corrected bone mineral density measurements of the total-body less head, lower limb, upper limb and skull. (TBLH) = total-body less head, (LL-BMD) = lower limb BMD, (UL-BMD) = upper limb BMD, (SK-BMD) = skull BMD, r_g_ = genetic correlation between trait 1 and trait 2. r_e_ = residual correlation between trait 1 and trait 2. All traits were adjusted for age, gender and height and weight. *P*-refers to the *P*-value for the likelihood ratio test of whether r_g_ = 0. Phenotypic correlations (r_p)_ were as follows: SK-BMD/TBLH-BMD (r_p_ = 0.44, SE = 0.012, P<0.001), SK-BMD/LL-BMD (r_p_ = 0.34, SE = 0.013, P<0.001), SK-BMD/UL-BMD (r_p_ = 0.41, SE = 0.013, P<0.001) and LL-BMD/UL-BMD (r_p_ = 0.64, SE = 0.010, P<0.001).
(DOCX)

Table S2 Characteristics of BMD measures and other anthropometrical traits for participants of the ALSPAC and GEN-R cohorts. (TBLH-BMD) = total-body less head BMD; (LL-BMD) = lower limb BMD; (UL-BMD) = upper limb BMD; (SK-BMD) = skull BMD; UNIT = unit of measurement; n = number of subjects; SD = standard deviation of the mean value; (C-MEAN) = mean of trait measured for males and females; (F-MEAN) = mean of trait measured in females; (M-MEAN) = mean of trait measured in males. ^*^Only 5299 subjects had skull BMD measurements.
(DOCX)

Table S3 Overall and population specific characteristics of BMD measures and other anthropometric traits in Generation R. (TBLH-BMD) = total-body less head BMD; (LL-BMD) = lower limb BMD; (UL-BMD) = upper limb BMD; (SK-BMD) = skull BMD; n = number of individuals; MEAN = mean value of each trait; SD = standard deviation of the mean each trait; UNIT = unit of measurement. Please note that these classifications are based on self-reported ethnicity.
(DOCX)

Table S4 Genome-wide associated TBLH-BMD variants. (CHR) = chromosome number; (POS) = position in the genome based on hg18; (EAF) = effect allele frequency; (*β*) = estimates of effect size expressed as adjusted SD per copy of the effect allele (EA); (SE) = standard error of *β*; (*P*) = *P*-value; (I^2^) = Cochran's Q statistic evaluating heterogeneity and (*P*_HET_) = evidence of heterogeneity. The SNP that showed the strongest evidence of association at each locus is displayed in bold font.
(DOCX)

Table S5 Genome-wide associated LL-BMD variants. (CHR) = chromosome number; (POS) = position in the genome based on hg18; (EAF) = effect allele frequency; (β) = estimates of effect size expressed as adjusted SD per copy of the effect allele (EA); (SE) = standard error of β; (P) = P-value; (I2) = Cochran's Q statistic evaluating heterogeneity and (P_HET_) = evidence of heterogeneity. The SNP that showed the strongest evidence of association at each locus is displayed in bold font.
(DOCX)

Table S6 Genome-wide associated UL-BMD variants. (CHR) = chromosome number; (POS) = position in the genome based on hg18; (EAF) = effect allele frequency; (*β*) = estimates of effect size expressed as adjusted SD per copy of the effect allele (EA); (SE) = standard error of *β*; (*P*) = *P*-value; (I^2^) = Cochran's Q statistic evaluating heterogeneity and (*P*_HET_) = evidence of heterogeneity. The SNP that showed the strongest evidence of association at each locus is displayed in bold font.
(DOCX)

Table S7 Genome-wide associated SK-BMD variants. (CHR) = chromosome number; (POS) = position in the genome based on hg18; (EAF) = effect allele frequency; (*β*) = estimates of effect size expressed as adjusted SD per copy of the effect allele (EA); (SE) = standard error of *β*; (*P*) = *P*-value; (I^2^) = Cochran's Q statistic evaluating heterogeneity and (*P*_HET_) = evidence of heterogeneity. The SNP that showed the strongest evidence of association at each locus is displayed in bold font.
(DOCX)

Table S8 Comparison of published BMD SNPs with results from the total-body less head, lower limb, upper limb and skull BMD GWAS. (LS-BMD) = lumbar spine BMD; (FN-BMD) = femoral neck BMD; (F-BMD) = forearm BMD; (TBLH-BMD) = total-body less head BMD; (LL-BMD) = lower limb BMD; (UL-BMD) = upper limb BMD; (SK-BMD) = skull BMD; (POSITION) = location in the genome based on hg18; (GENE) = closest gene; (PMID) = accession number of the publication in Pubmed from which the summary statistics were obtained; (EA) = effect allele; (EAF) = effect allele frequency; (β) = estimates of effect size expressed as adjusted SD per copy of the effect allele (EA); (SE) = standard error of *β* and (*P*) = pvalue; We failed to obtain estimates for: rs9287237 (1q43, *FMN2*); rs7017914 (8q13.3, *XKR9*); rs7851693 (9q34.11, *FUBP3*) and rs5934507 (Xp22.31, *FAM9B*) as they were not imputed in the GEN-R dataset. Note for rs9287237^*^ (*FMN2*) and rs271170^*^ (*LOC285735/EYA4*) the summary statistics were obtained from a study performed by Patenoster *et. al.* 2013 and represent the effect sizes and evidence of association for these SNPs with volumetric trabecular (LS-BMD column) and cortical BMD (FN-BMD column).
(DOCX)

Table S9 Sensitivity analysis comparing genome-wide significant SNPs associated with bone mineral density measured at four skeletal sites. (TBLH-BMD) = total-body less head BMD, (LL-BMD) = lower limb BMD, (UL-BMD) = upper limb BMD, (SK-BMD) = skull BMD. (MODEL 0) = GWAS meta-analysis performed on age-, gender-, weight- or height-adjusted BMD, (MODEL 1a) = GWAS meta-analysis performed on age-, gender-, weight- and height-adjusted BMD, (MODEL 1b) = GWAS meta-analysis performed on age-, gender-, weight- and height-adjusted BMD measurements in individuals of European ancestry. (GENE) = closest gene, (POS) = position in the genome based on hg18, (EAF) = effect allele frequency, (*β*) = estimates of effect size expressed as adjusted SD per copy of the effect allele (EA), (SE) = standard error of *β*, (*P*) = pvalue, (I^2^) = Cochran's Q statistic evaluating heterogeneity, (*P*_HET_) = evidence of heterogeneity and ^*^Sample sizes used for SK-BMD genome-wide meta-analysis. **Please note that *PTHLH* is also located at the 12p11.22 locus containing *KLHDC5, RSPO3* is also located at the 6q.22.32 locus containing *CENPW, FAM3C and CPED1* are also located at the 7q.31.31 locus containing *WNT16*, *TNFRSF11B* is also located at the 8q.24.12 locus containing *COLEC10, LGR4* is also located at the 11p14.1 locus containing *LIN7C* and *LRP5* is also located at the 11q13.2 locus containing *PPP6R3*.
(DOCX)

Table S10 Top SNPs associated with bone mineral density of the total-body less head, lower limb, upper limb and skull after conditional meta-analysis. (TBLH-BMD) = total-body less head BMD, (LL-BMD) = lower limb BMD, (UL-BMD) = upper limb BMD, (SK-BMD) = skull BMD, (GENE) = closest gene, (POS) = position in the genome based on hg18, (EAF) = effect allele frequency, (*β*) = estimates of effect size expressed as adjusted SD per copy of the effect allele (EA), (SE) = standard error of *β*, (*P*) = *P*-value, (I^2^) = Cochran's Q statistic evaluating heterogeneity and (*P*_HET_) = evidence of heterogeneity. ^*^Sample sizes used for SK-BMD genome-wide meta-analysis. Locus specific multiple testing correction thresholds as calculated by SNPSpD for SNPs in LD are as follows: 1p36.12 (*P*≤7.5×10^−5^), 2q24.3 (*P*≤4.7×10^−5^), 6q22.32 (*P*≤7.2×10^−5^), 6q23.2 (*P*≤8.9×10^−5^), 7q31.31 (*P*≤1.2×10^−4^), 8q24.12 (*P*≤4.7×10^−5^), 9q34.11 (*P*≤9.4×10^−5^), 11p14.1 (*P*≤1.1×10^−4^), 11q13.2 (*P*≤6.3×10^−5^), 12p11.22 (*P*≤4.1×10^−5^), 13q14.11 (*P*≤3.7×10^−5^), 14q32.12 (*P*≤4.5×10^−5^) and 18q21.33 (*P*≤4.1×10^−5^). ^**^Please note that *PTHLH* is also located at the 12p11.22 locus containing KLHDC5 and *RSPO3* is also located at the 6q.22.32 locus containing *CENPW. FAM3C and CPED1* are also located at the 7q.31.31 locus containing *WNT16*.
(DOCX)

Table S11 Lookup of selected primary and secondary BMD SNPs in the publically released GEFOS GWAS of hip and spine BMD, in addition to a comparison of the summary statistics across each skeletal site before conditional analysis. (TBLH-BMD) = total-body less head BMD; (LL-BMD) = lower limb BMD; (UL-BMD) = upper limb BMD; (SK-BMD) = skull BMD; (LS-BMD) = lumbar spine BMD; (FN-BMD) = femoral neck BMD; (GENE) = closest gene; (EA) = effect allele; (*β*) = estimates of effect size expressed as adjusted SD per copy of the effect allele (EA); (SE) = standard error of *β* and (*P*) = pvalue. Note – all the summary statistics refer to those obtained prior to conditional analysis and in the case of femoral neck or lumbar spine, the results were obtained from the publically available data release from the GEFOS consortium.
(DOCX)

Table S12 Published SNPs used for conditional meta-analyses. (LS-BMD) = lumbar spine BMD; (FN-BMD) = femoral neck BMD; (F-BMD) = forearm BMD; (TBLH-BMD) = total-body less head BMD; (SK-BMD) = skull BMD; BMD; (TRABEC-BMD) = volumetric trabecular BMD of the tibia; (CORT-BMD) = volumetric cortical BMD of the tibia. (POSITION) = location in the genome based on hg18; (GENE) = closest gene; (PMID) = accession number of the publication in Pubmed from which the summary statistics were obtained; (*β*) = estimates of effect size expressed as adjusted SD per copy of the effect allele; (SE) = standard error of *β* and (*P*) = *P*-value. *Please note that *PTHLH* is also located at the 12p11.22 locus containing *KLHDC5, RSPO3* is also located at the 6q.22.32 locus containing *CENPW, FAM3C and CPED1* are also located at the 7q.31.31 locus containing *WNT16*, *TNFRSF11B* is also located at the 8q.24.12 locus containing *COLEC10, LGR4* is also located at the 11p14.1 locus containing *LIN7C* and *LRP5* is also located at the 11q13.2 locus containing *PPP6R3*. **The Generation R cohort did not impute the published *FUBP3* SNP (rs7851693) and therefore we chose to condition on rs7030440, a SNP which was in high LD (HapMap phase 2 release 22, CEU: r^2^ = 0.96) with the published *FUBP3* associated BMD variant. ***No previous BMD SNPs found in *14q32.12* have been published.
(DOCX)

Table S13 Comparison of transcript levels between healthy and osteoporotic women. Transcript log2 signal levels expressed from genes ±250 Kb of rs754388 were compared between postmenopausal osteoporotic women with fracture and healthy controls using students T-test. Transcripts with maximal log2 signal values below 4 were excluded. (SD) = Standard deviation and (*P*) = *P*-value.
(DOCX)
