# Supplementary figures and images for: Phenotypic Dissection of Bone Mineral Density Reveals Skeletal Site Specificity and Facilitates the Identification of Novel Loci in the Genetic Regulation of Bone Mass Attainment
Source: PLoS Genet. 2014 Jun 19;10(6):e1004423. doi: 10.1371/journal.pgen.1004423 (PMC4063697; doi:10.1371/journal.pgen.1004423)

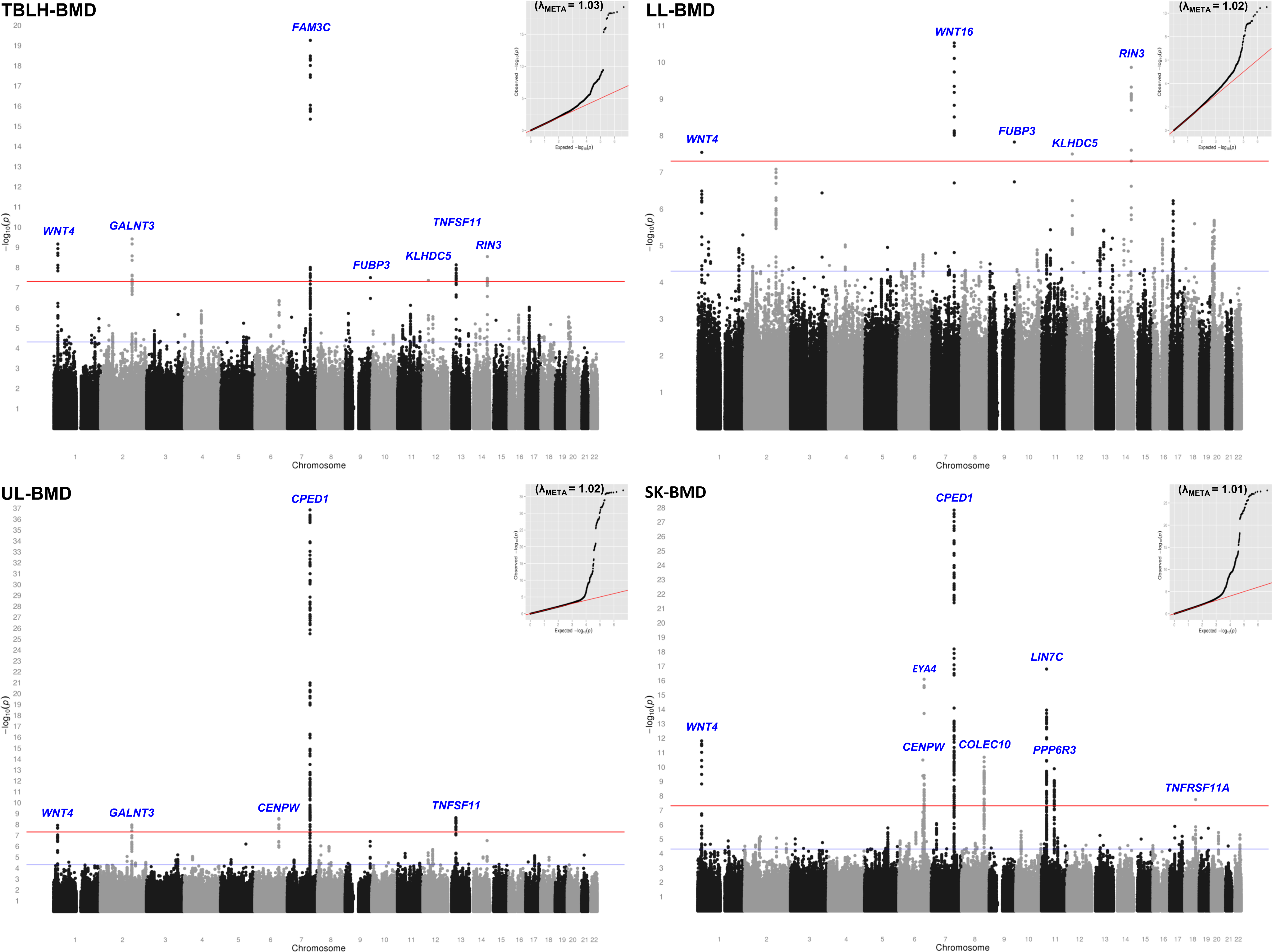

Supplement: Figure S1 — Genome-wide association meta-analysis of age-, gender-, height- or weight-adjusted BMD measured at four different skeletal sites. Manhattan and Q-Q plots derived from the genome-wide association meta-analysis of BMD measures of the total-body less head (TBLH), lower limb (LL), upper limb (UL) and skull (SK). The names of the closest genes relative to the each locus specific top SNP are indicated in blue. Q-Q plots show the inflation of the test statistics (λMETA) of each genome-wide association meta-analysis. *Please note that PTHLH is also located at the 12p11.22 locus containing KLHDC5, RSPO3 is also located at the 6q.22.32 locus containing CENPW, FAM3C and CPED1 are also located at the 7q.31.31 locus containing WNT16, TNFRSF11B is also located at the 8q.24.12 locus containing COLEC10, LGR4 is also located at the 11p14.1 locus containing LIN7C and LRP5 is also located at the 11q13.2 locus containing PPP6R3. (TIF) [file pgen.1004423.s001.tif]

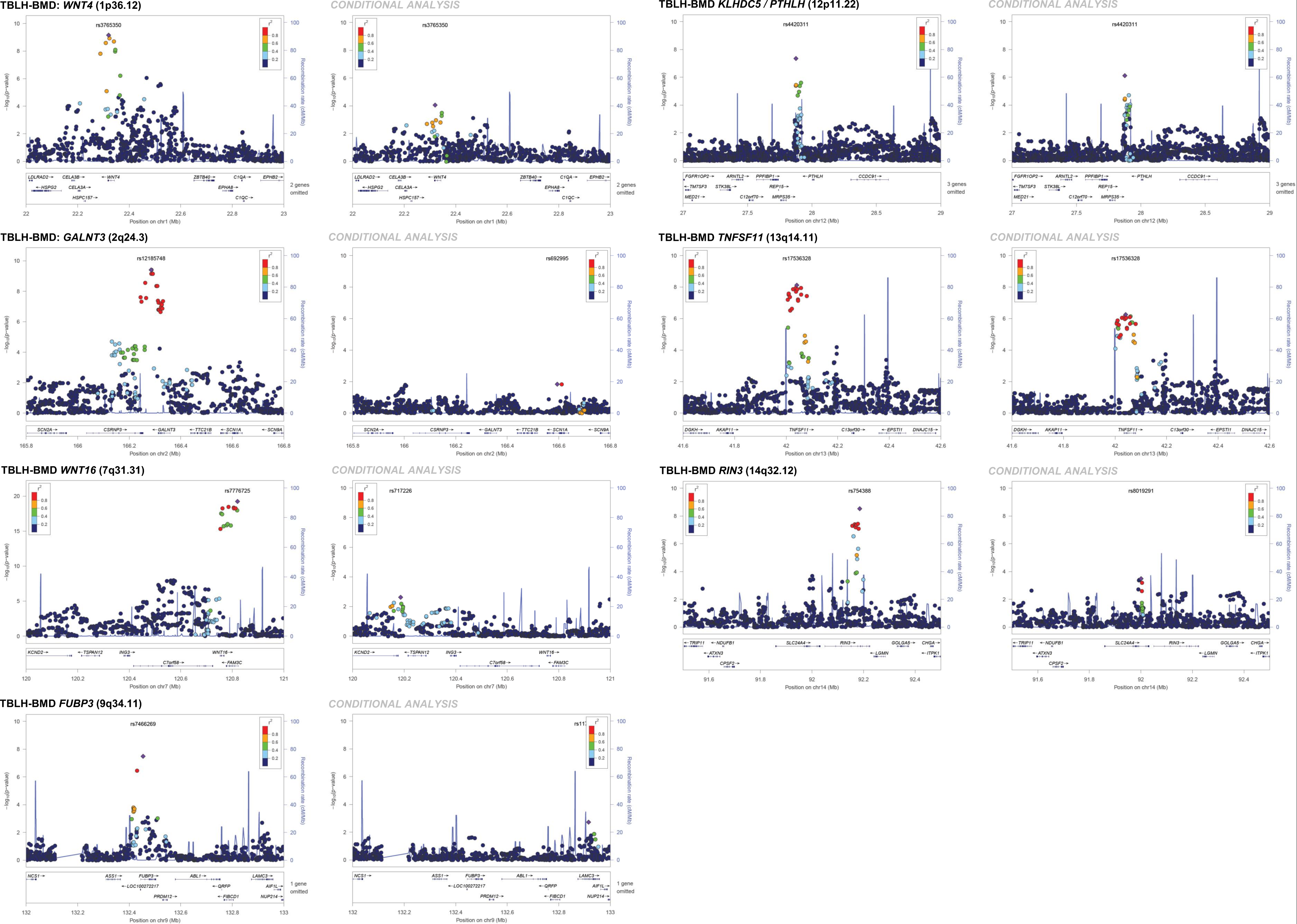

Supplement: Figure S2 — Regional association plots for all loci which reached genome-wide significance for TBLH-BMD before and after conditioning on known BMD associated SNPs. Circles show GWA meta-analysis P-values and positions of SNPs found within each locus. The top SNP are denoted by diamonds. Different colours indicate varying degrees of pair wise linkage disequilibrium estimates between the top SNP and all other SNPs. *Please note that PTHLH is also located at the 12p11.22 locus containing KLHDC5, RSPO3 is also located at the 6q.22.32 locus containing CENPW, FAM3C and CPED1 are also located at the 7q.31.31 locus containing WNT16, TNFRSF11B is also located at the 8q.24.12 locus containing COLEC10, LGR4 is also located at the 11p14.1 locus containing LIN7C and LRP5 is also located at the 11q13.2 locus containing PPP6R3. (TIF) [file pgen.1004423.s002.tif]

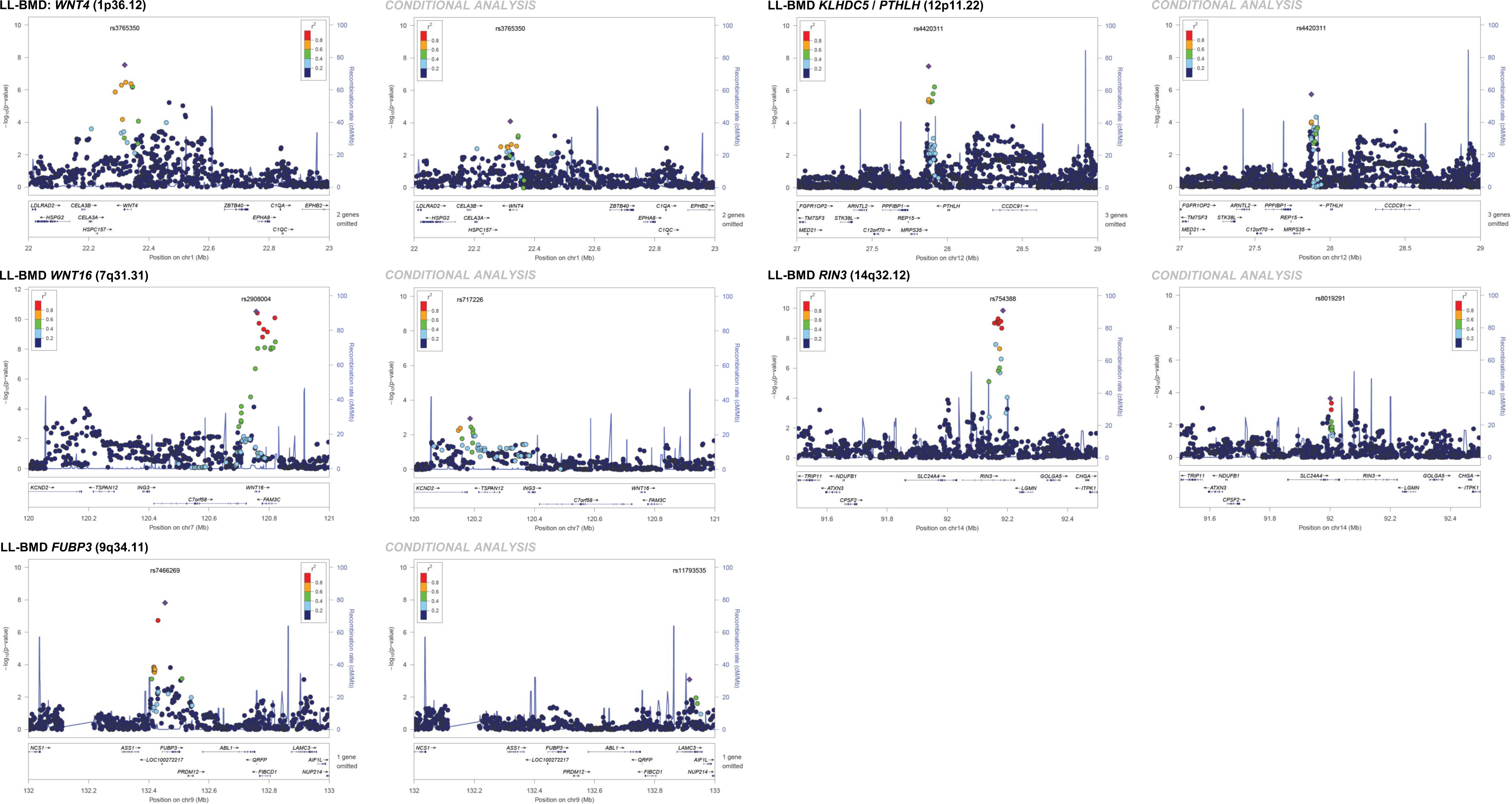

Supplement: Figure S3 — Regional association plots for all loci which reached genome-wide significance for LL-BMD before and after conditioning on known BMD associated SNPs. Circles show GWA meta-analysis P-values and positions of SNPs found within each locus. The top SNP are denoted by diamonds. Different colours indicate varying degrees of pair wise linkage disequilibrium estimates between the top SNP and all other SNPs. *Please note that PTHLH is also located at the 12p11.22 locus containing KLHDC5, RSPO3 is also located at the 6q.22.32 locus containing CENPW, FAM3C and CPED1 are also located at the 7q.31.31 locus containing WNT16, TNFRSF11B is also located at the 8q.24.12 locus containing COLEC10, LGR4 is also located at the 11p14.1 locus containing LIN7C and LRP5 is also located at the 11q13.2 locus containing PPP6R3. (TIF) [file pgen.1004423.s003.tif]

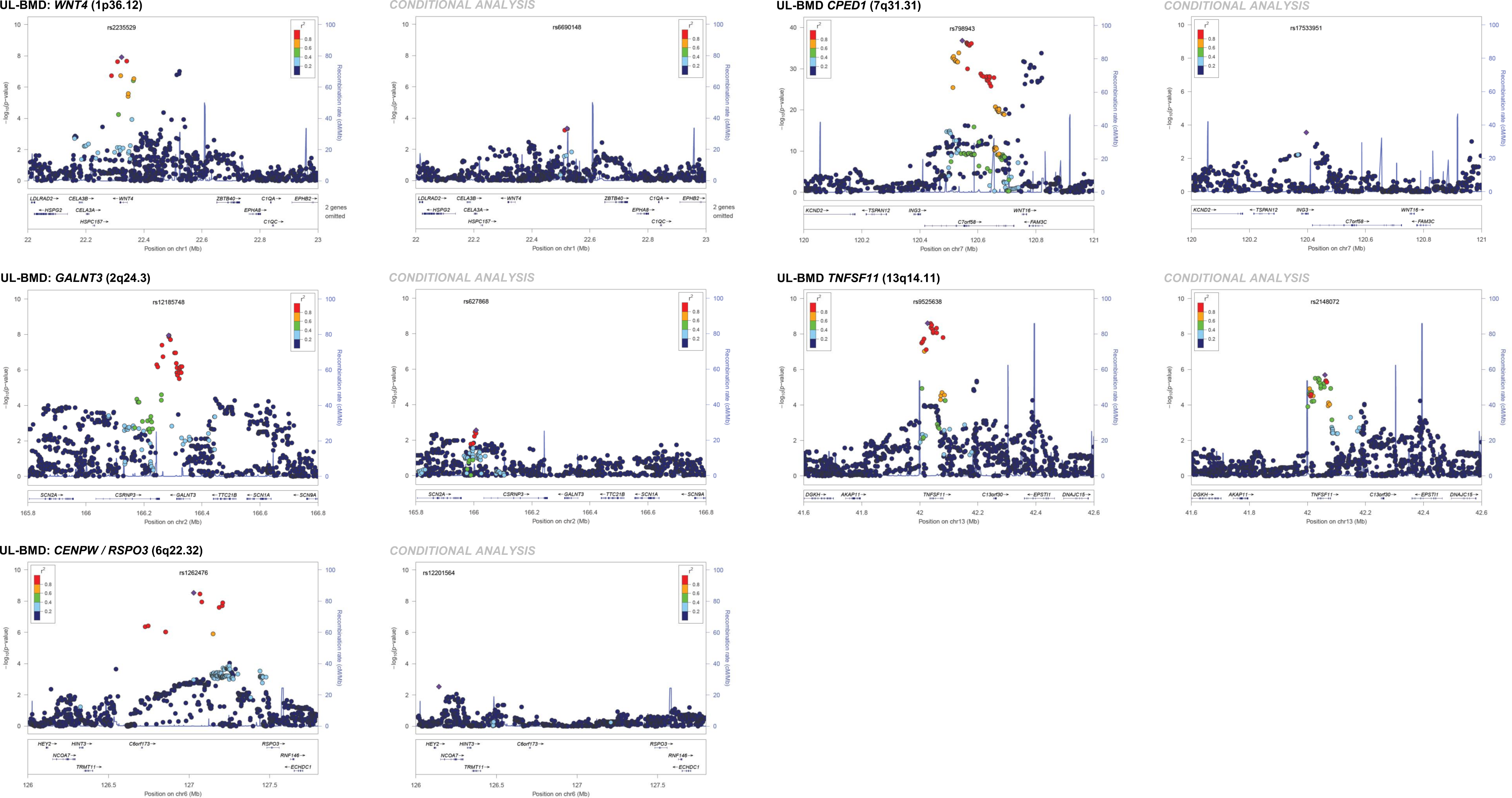

Supplement: Figure S4 — Regional association plots for all loci which reached genome-wide significance for UL-BMD before and after conditioning on known BMD associated SNPs. Circles show GWA meta-analysis P-values and positions of SNPs found within each locus. The top SNP are denoted by diamonds. Different colours indicate varying degrees of pair wise linkage disequilibrium estimates between the top SNP and all other SNPs. *Please note that PTHLH is also located at the 12p11.22 locus containing KLHDC5, RSPO3 is also located at the 6q.22.32 locus containing CENPW, FAM3C and CPED1 are also located at the 7q.31.31 locus containing WNT16, TNFRSF11B is also located at the 8q.24.12 locus containing COLEC10, LGR4 is also located at the 11p14.1 locus containing LIN7C and LRP5 is also located at the 11q13.2 locus containing PPP6R3. (TIF) [file pgen.1004423.s004.tif]

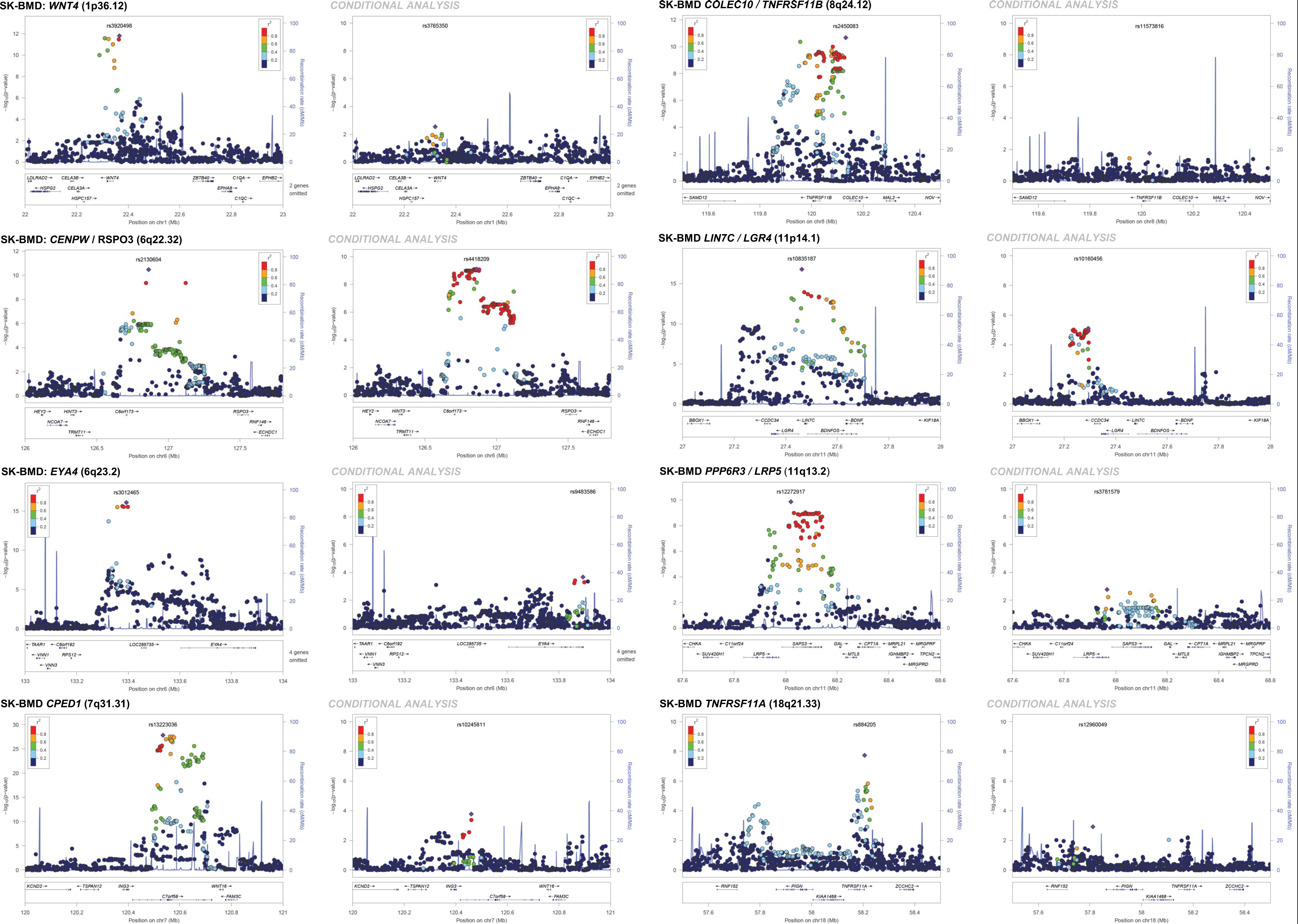

Supplement: Figure S5 — Regional association plots for all loci which reached genome-wide significance for SK-BMD before and after conditioning on known BMD associated SNPs. Circles show GWA meta-analysis P-values and positions of SNPs found within each locus. The top SNP are denoted by diamonds. Different colours indicate varying degrees of pair wise linkage disequilibrium estimates between the top SNP and all other SNPs.*Please note that PTHLH is also located at the 12p11.22 locus containing KLHDC5, RSPO3 is also located at the 6q.22.32 locus containing CENPW, FAM3C and CPED1 are also located at the 7q.31.31 locus containing WNT16, TNFRSF11B is also located at the 8q.24.12 locus containing COLEC10, LGR4 is also located at the 11p14.1 locus containing LIN7C and LRP5 is also located at the 11q13.2 locus containing PPP6R3. (TIF) [file pgen.1004423.s005.tif]

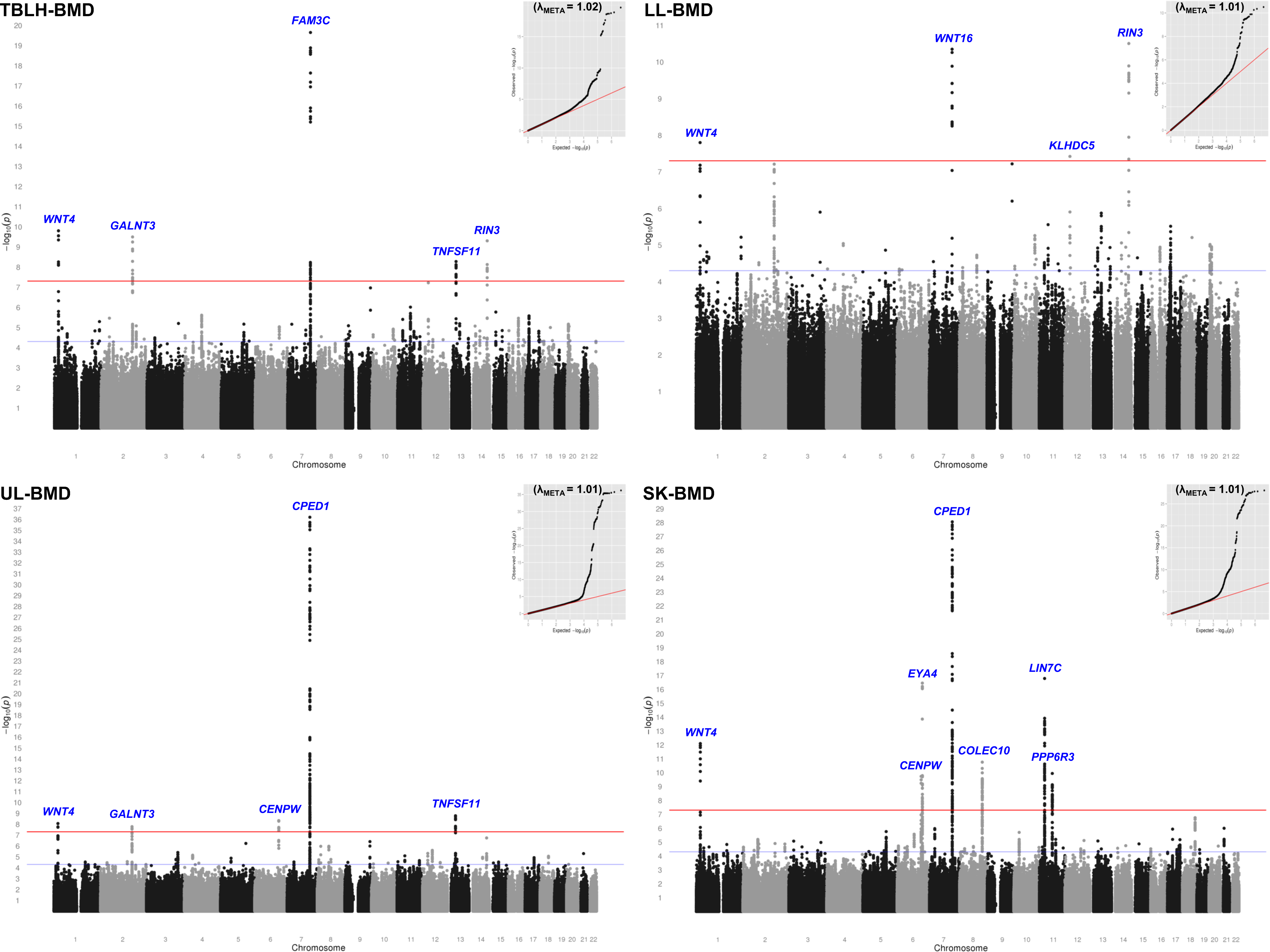

Supplement: Figure S6 — Genome-wide association meta-analysis of age-, gender-, height- and weight-adjusted BMD measured at four different skeletal sites. Manhattan and Q-Q plots derived from the genome-wide association meta-analysis of BMD measures of the total-body less head (TBLH), lower limb (LL), upper limb (UL) and skull (SK). The names of the closest genes relative to the each locus specific top SNP are indicated in blue. Q-Q plots show the inflation of the test statistics (λMETA) of each genome-wide association meta-analysis. *Please note that PTHLH is also located at the 12p11.22 locus containing KLHDC5, RSPO3 is also located at the 6q.22.32 locus containing CENPW, FAM3C and CPED1 are also located at the 7q.31.31 locus containing WNT16, TNFRSF11B is also located at the 8q.24.12 locus containing COLEC10, LGR4 is also located at the 11p14.1 locus containing LIN7C and LRP5 is also located at the 11q13.2 locus containing PPP6R3. (TIF) [file pgen.1004423.s006.tif]

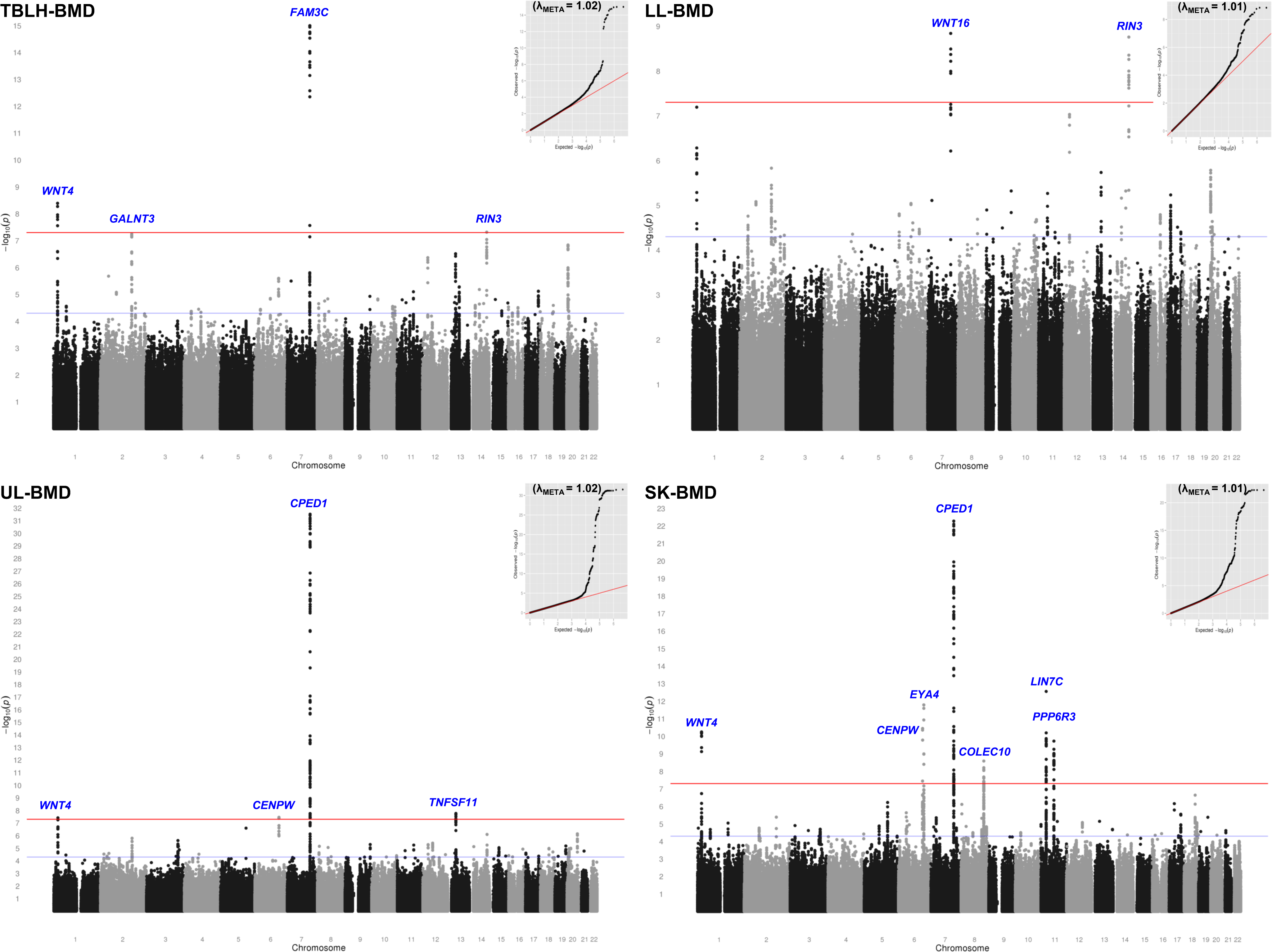

Supplement: Figure S7 — Genome-wide association meta-analysis of age-, gender-, height- and weight-adjusted BMD measured at four different skeletal sites in individuals of European ancestry. Manhattan and Q-Q plots derived from the genome-wide association meta-analysis of BMD measures of the total-body less head (TBLH), lower limb (LL), upper limb (UL) and skull (SK). The names of the closest genes relative to the each locus specific top SNP are indicated in blue. Q-Q plots show the inflation of the test statistics (λMETA) of each genome-wide association meta-analysis. *Please note that PTHLH is also located at the 12p11.22 locus containing KLHDC5, RSPO3 is also located at the 6q.22.32 locus containing CENPW, FAM3C and CPED1 are also located at the 7q.31.31 locus containing WNT16, TNFRSF11B is also located at the 8q.24.12 locus containing COLEC10, LGR4 is also located at the 11p14.1 locus containing LIN7C and LRP5 is also located at the 11q13.2 locus containing PPP6R3. (TIF) [file pgen.1004423.s007.tif]

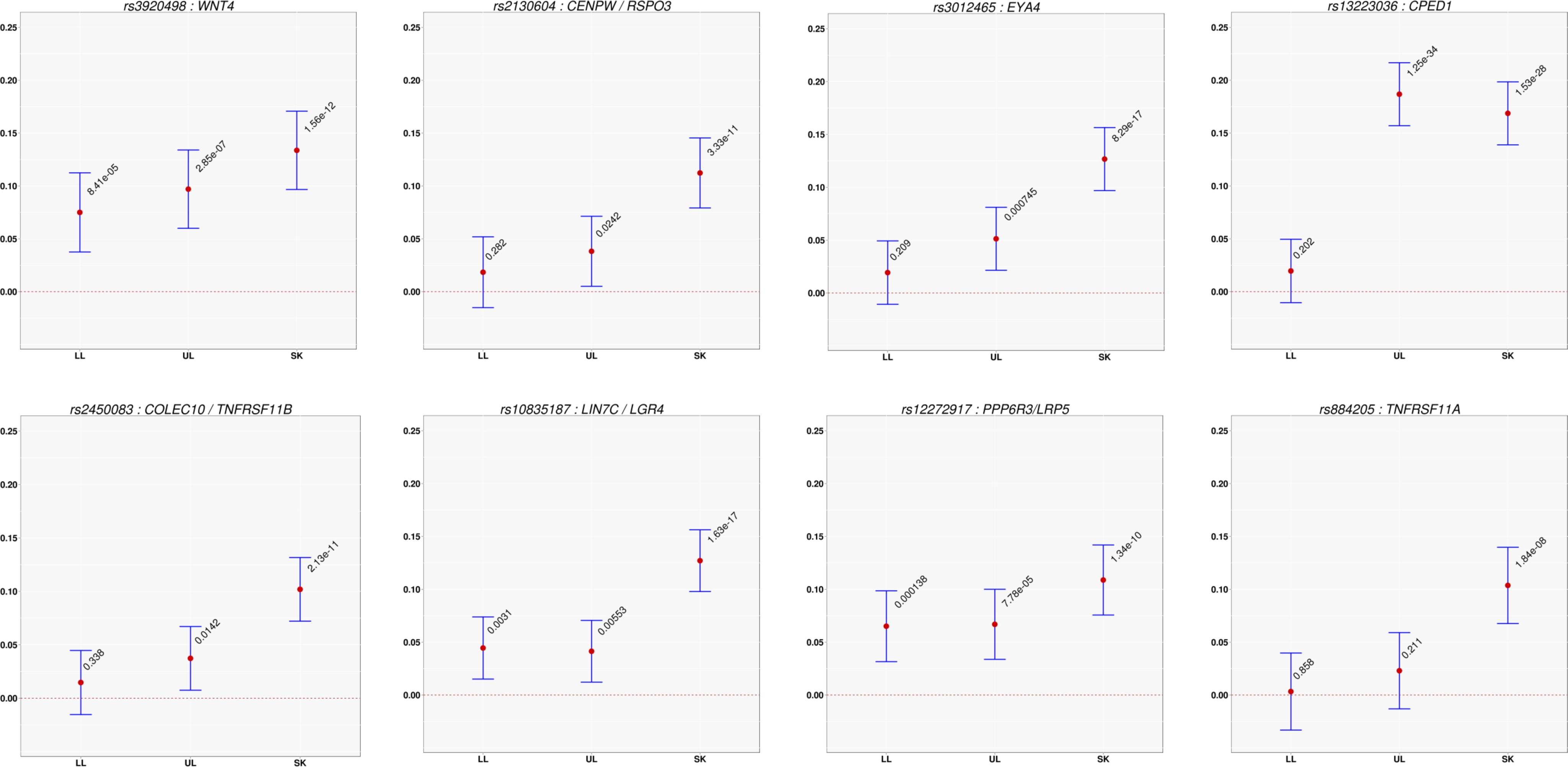

Supplement: Figure S8 — Comparison of effect sizes of the top SK-BMD associated variants across each skeletal site. The per allele effect in SD (red dot) and 95% confidence interval (error bar) of the top SNP associated with BMD measurements of the lower limb (LL), upper limb (UL) and skull (SK) are plotted with their specific strength of association. *Please note that PTHLH is also located at the 12p11.22 locus containing KLHDC5, RSPO3 is also located at the 6q.22.32 locus containing CENPW, FAM3C and CPED1 are also located at the 7q.31.31 locus containing WNT16, TNFRSF11B is also located at the 8q.24.12 locus containing COLEC10, LGR4 is also located at the 11p14.1 locus containing LIN7C and LRP5 is also located at the 11q13.2 locus containing PPP6R3. (TIF) [file pgen.1004423.s008.tif]

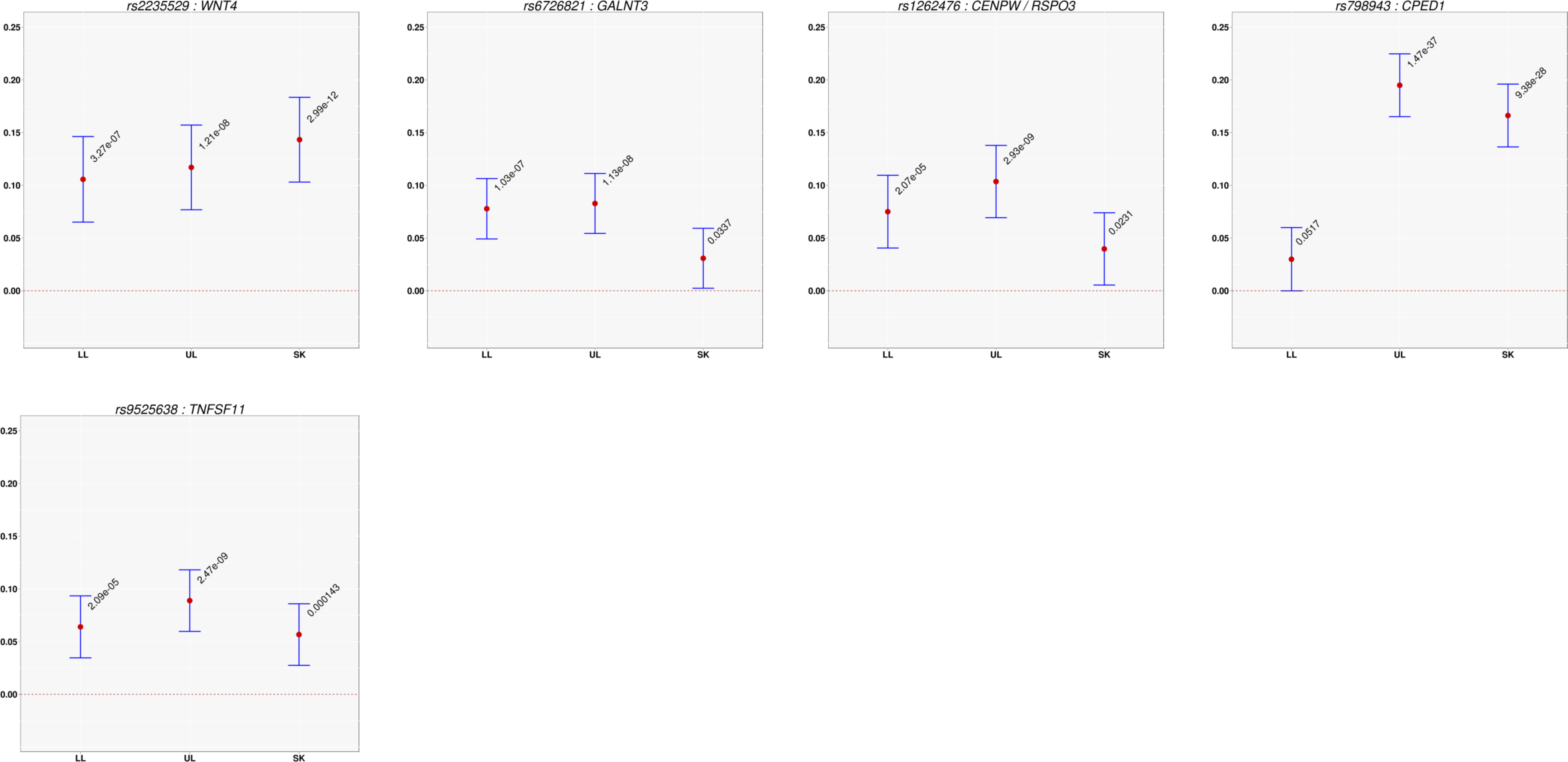

Supplement: Figure S9 — Comparison of effect sizes of the top UL-BMD associated variants across each skeletal site. The per allele effect in SD (red dot) and 95% confidence interval (error bar) of the top SNP associated with BMD measurements of the lower limb (LL), upper limb (UL) and skull (SK) are plotted with their specific strength of association. *Please note that PTHLH is also located at the 12p11.22 locus containing KLHDC5, RSPO3 is also located at the 6q.22.32 locus containing CENPW, FAM3C and CPED1 are also located at the 7q.31.31 locus containing WNT16, TNFRSF11B is also located at the 8q.24.12 locus containing COLEC10, LGR4 is also located at the 11p14.1 locus containing LIN7C and LRP5 is also located at the 11q13.2 locus containing PPP6R3. (TIF) [file pgen.1004423.s009.tif]

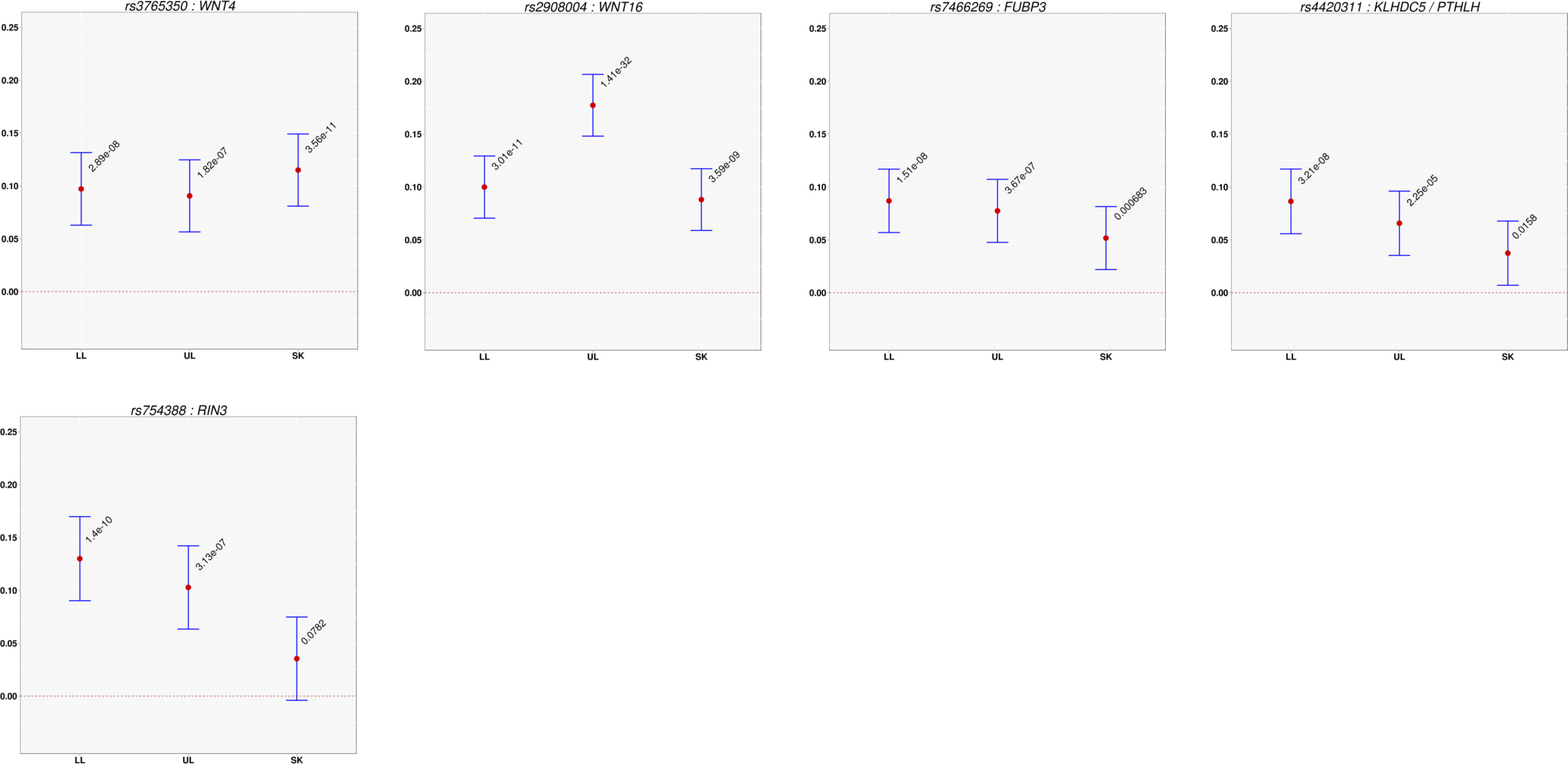

Supplement: Figure S10 — Comparison of effect sizes of the top LL-BMD associated variants across each skeletal site. The per allele effect in SD (red dot) and 95% confidence interval (error bar) of the top SNP associated with BMD measurements of the lower limb (LL), upper limb (UL) and skull (SK) are plotted with their specific strength of association. *Please note that PTHLH is also located at the 12p11.22 locus containing KLHDC5, RSPO3 is also located at the 6q.22.32 locus containing CENPW, FAM3C and CPED1 are also located at the 7q.31.31 locus containing WNT16, TNFRSF11B is also located at the 8q.24.12 locus containing COLEC10, LGR4 is also located at the 11p14.1 locus containing LIN7C and LRP5 is also located at the 11q13.2 locus containing PPP6R3. (TIF) [file pgen.1004423.s010.tif]

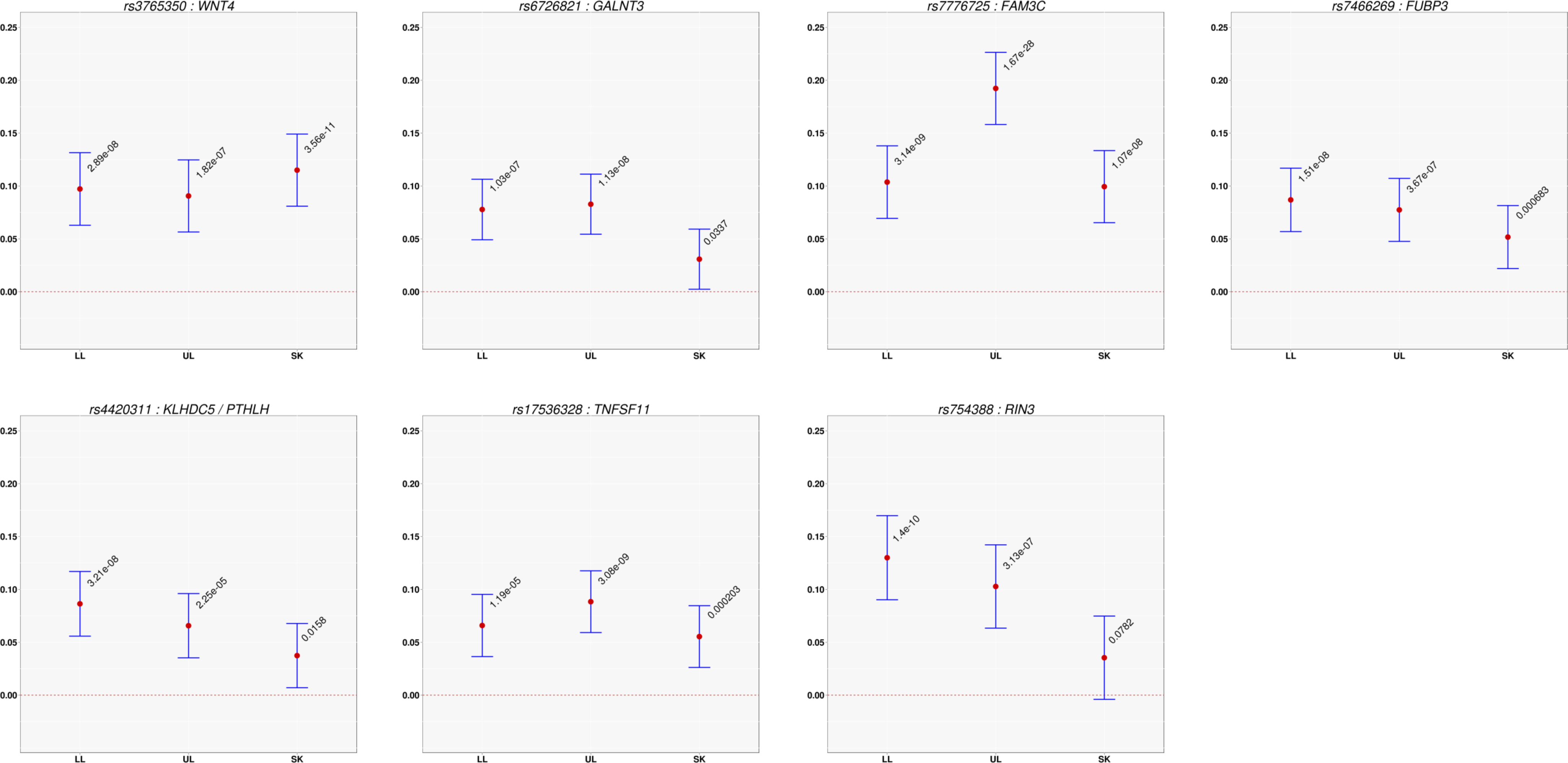

Supplement: Figure S11 — Comparison of effect sizes of the top TBLH-BMD associated variants across each skeletal site. The per allele effect in SD (red dot) and 95% confidence interval (error bar) of the top SNP associated with BMD measurements of the lower limb (LL), upper limb (UL) and skull (SK) are plotted with their specific strength of association. *Please note that PTHLH is also located at the 12p11.22 locus containing KLHDC5, RSPO3 is also located at the 6q.22.32 locus containing CENPW, FAM3C and CPED1 are also located at the 7q.31.31 locus containing WNT16, TNFRSF11B is also located at the 8q.24.12 locus containing COLEC10, LGR4 is also located at the 11p14.1 locus containing LIN7C and LRP5 is also located at the 11q13.2 locus containing PPP6R3. (TIF) [file pgen.1004423.s011.tif]

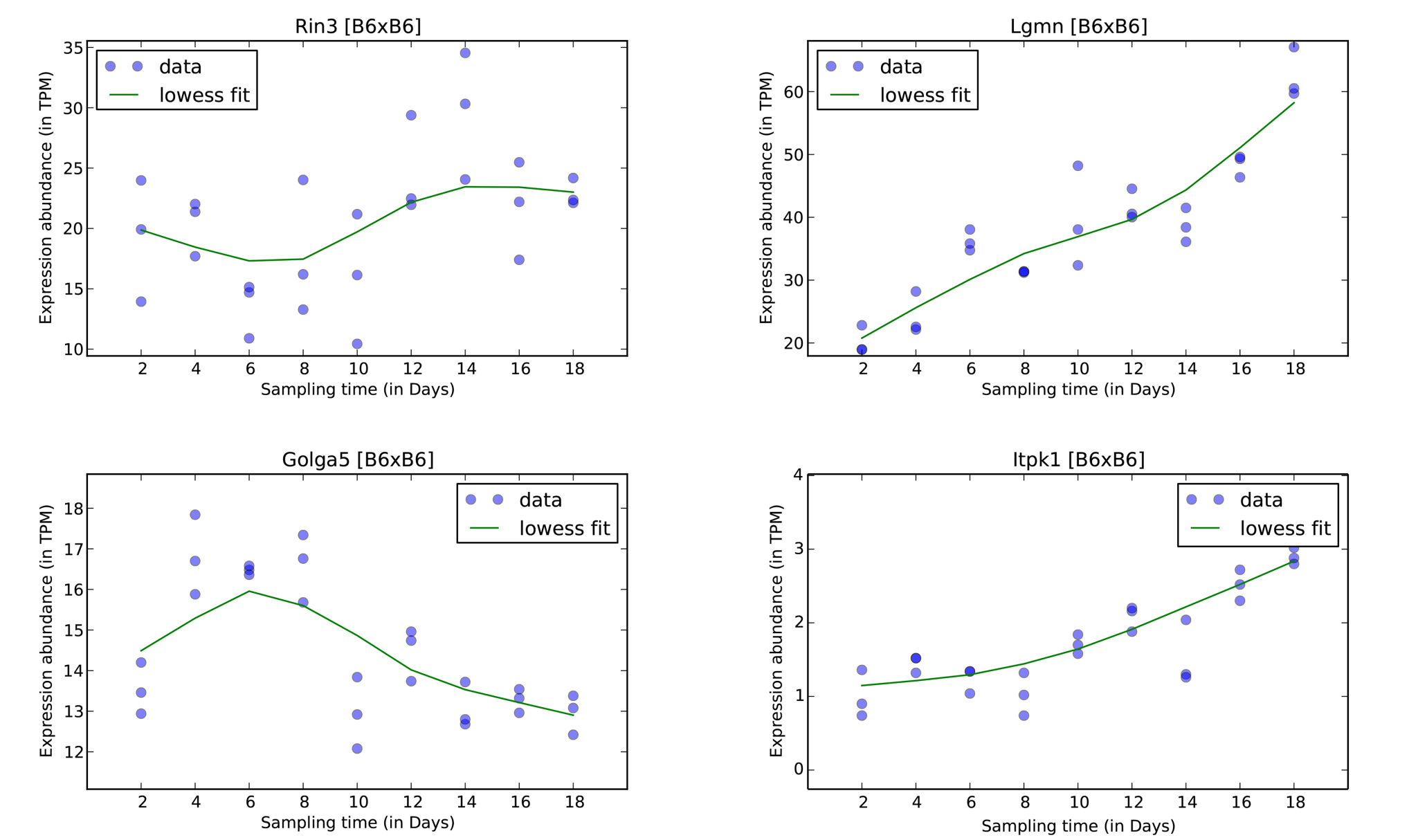

Supplement: Figure S12 — Gene expression profiles of Rin3, Golga5 Lgmn, and Itpk1 measured throughout the osteoblast maturation process in cells extracted from mouse calvariae, as measured by RNAseq. Samples for expression purposes were collected every other day for 18 days, starting 2 days after the cells were first exposed to an osteoblast differentiation cocktail. Relative transcript abundance is expressed as the number of query transcripts per million unique transcripts (transcripts per million), after normalizing to the upper quartile. A local weighted scatterplot smoothing curve was plotted to help with visualizing the expression pattern. Note: Slc24a4 and Chgm were not expressed in this cell type and have not been included in the figure. (TIF) [file pgen.1004423.s012.tif]

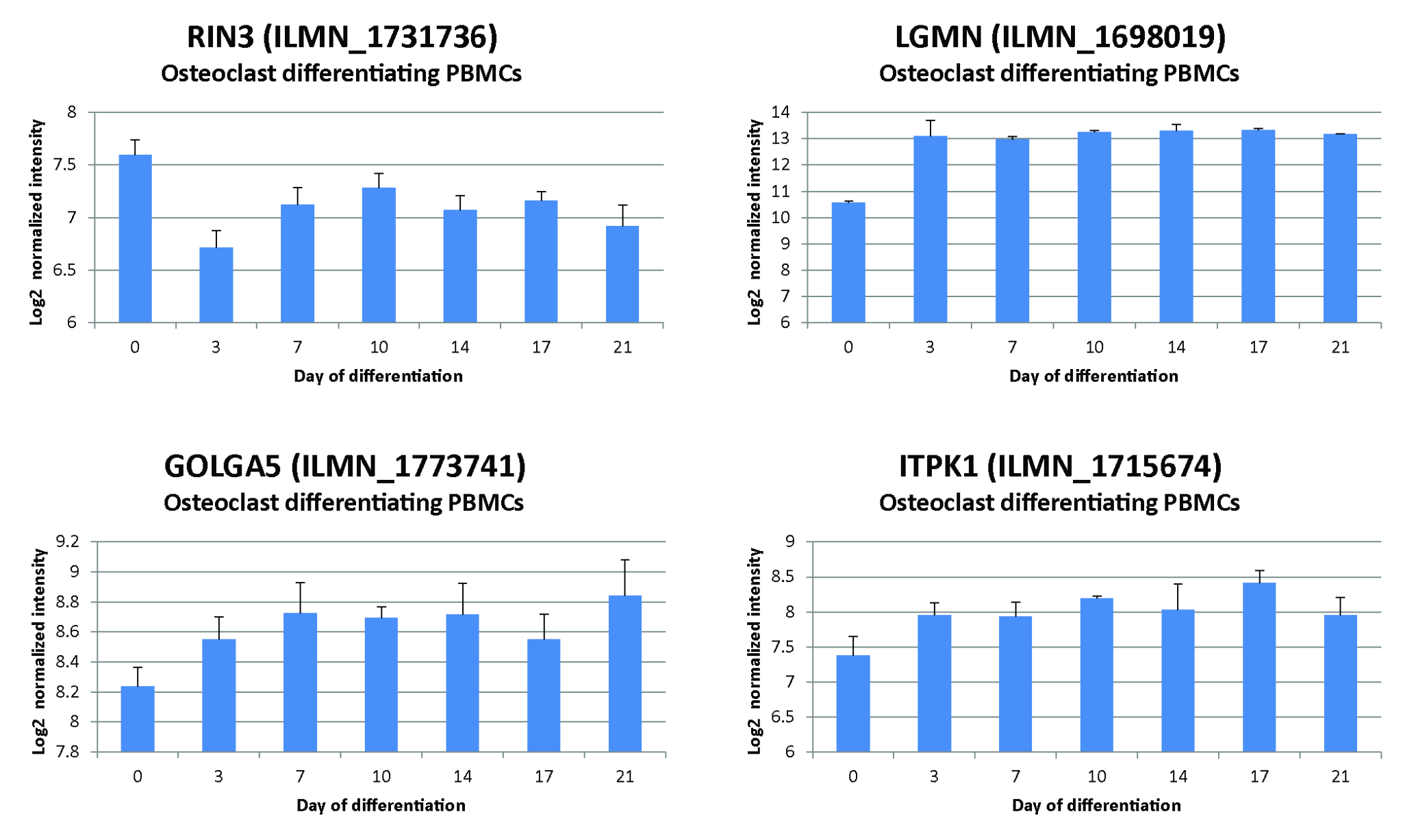

Supplement: Figure S13 — Gene expression profile of RIN3, LGMN, GOLGA5 and ITPK1 measured in osteoclast differentiating human PBMCs. Relative transcript abundance is expressed as Log2 normalized intensities. Each value is an average of 3 independent measurements and a standard deviation. (TIF) [file pgen.1004423.s013.tif]

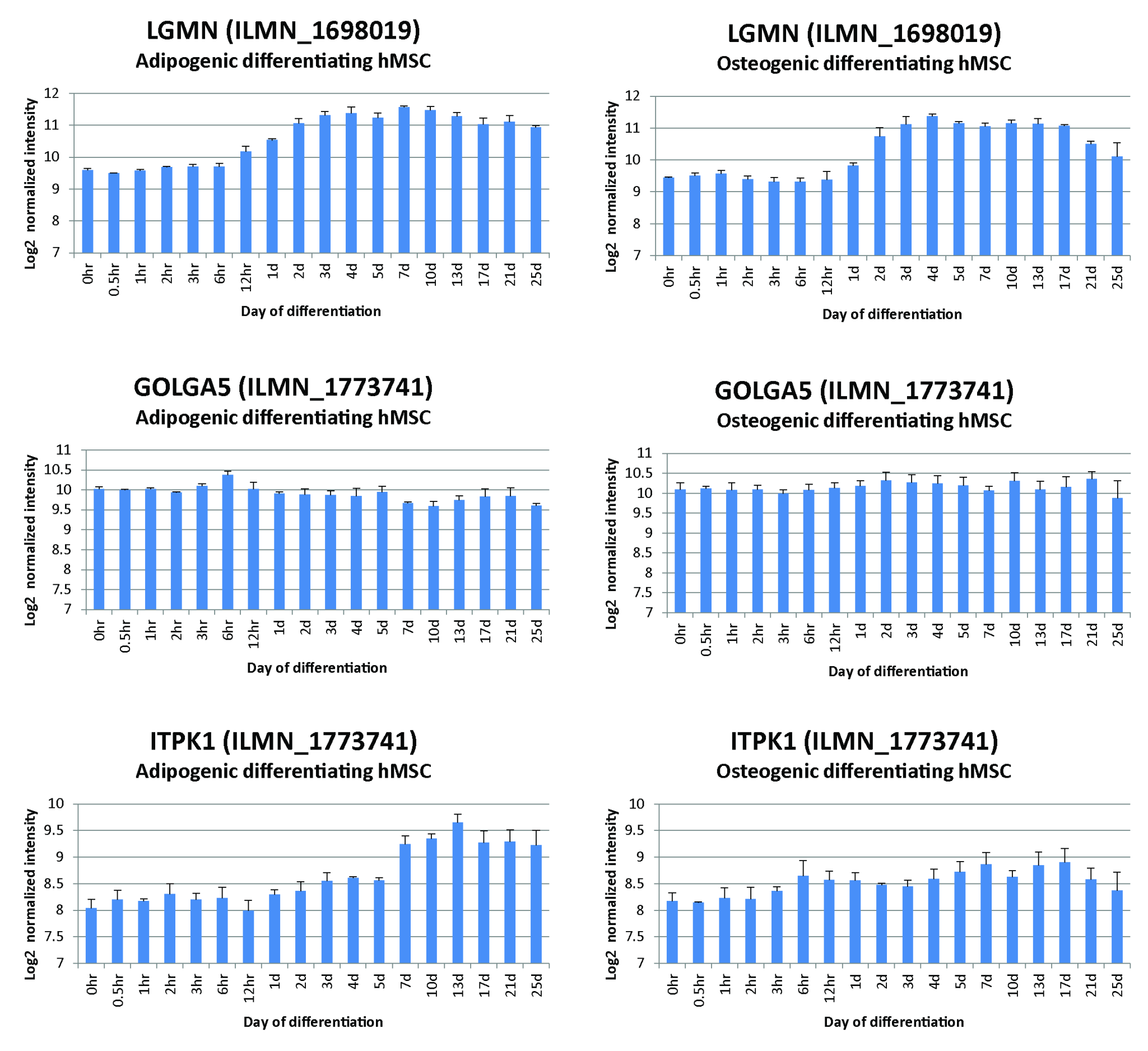

Supplement: Figure S14 — Gene expression profile of RIN3, LGMN, GOLGA5 and ITPK1 measured in adipogenic and osteogenic differentiating hMSCs. Relative transcript abundance is expressed as Log2 normalized intensities. RIN3 expression levels in differentiating hMSC are absent because the intensities were at background level. Each value is an average of 3 independent measurements and a standard deviation. (TIF) [file pgen.1004423.s014.tif]

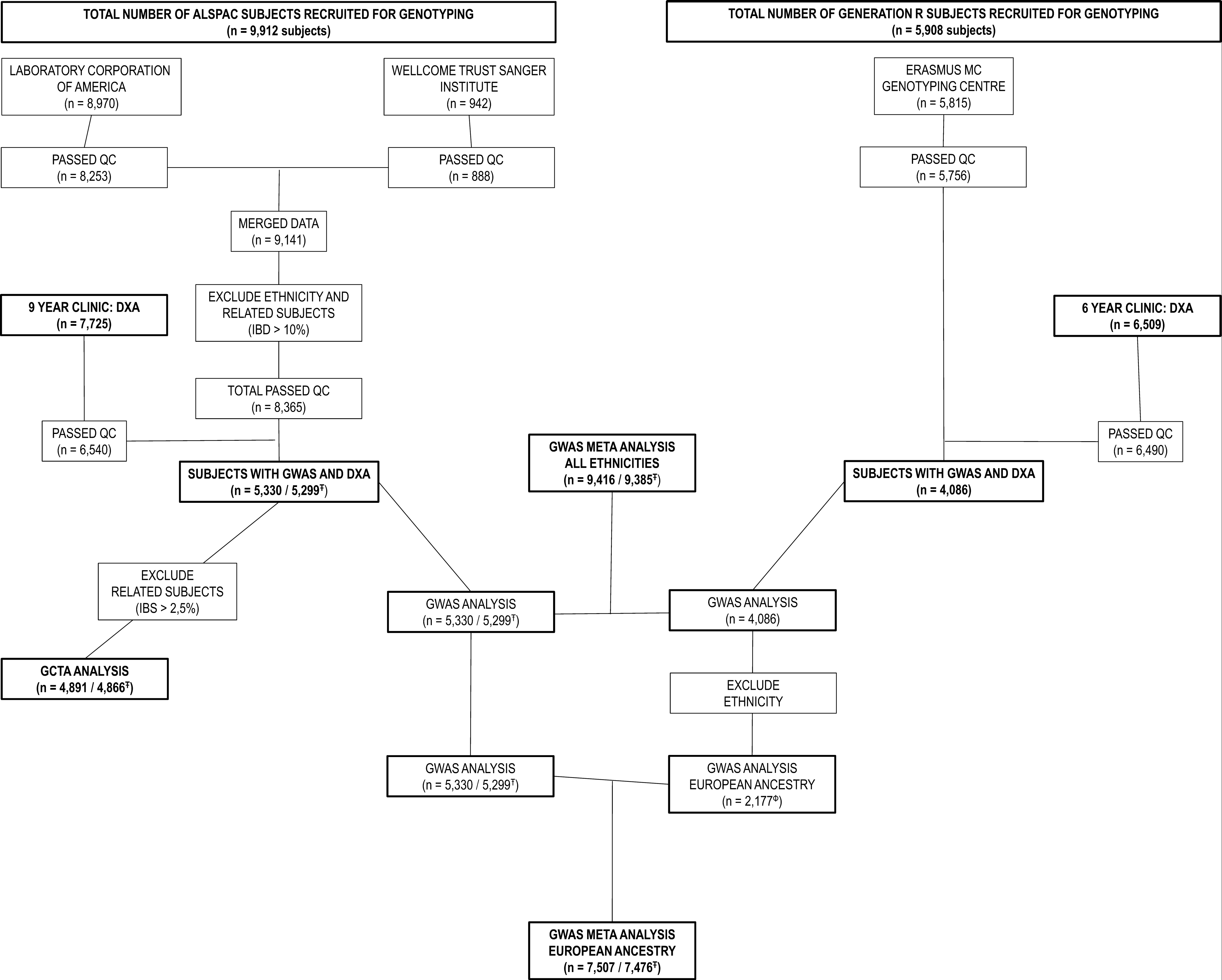

Supplement: Figure S15 — Flow diagram and overview of the analysis strategy used in this study. For ALSPAC, a total of 9,912 subjects were genotyped by Wellcome Trust Sanger Institute, Cambridge and the Laboratory Corporation of America. Individuals were excluded from further analysis using several quality control (QC) criteria (See methods). After merging and further QC, 8,365 unrelated subjects [identity by decent (IBD) <10% and of European ancestry] were available for GWAS analysis. Total-body DXA scans were performed on 7725 subjects that attended the Focus 9 clinic. Of these a total of 6540 passed DXA QC. For total body (TB), lower limb- (LL) and upper limb (UL) GWAS analysis 5,330 subjects had high quality bone mineral density (BMD) and genetic data, whereas 5,229 subjects were available for skull (S). For GCTA analysis, we employed a strict threshold of genome-wide identity by state >2.5% and resulting in the exclusion of additional individuals on the basis of cryptic relatedness. 4,891 (TB-, LL- and UL-BMD) and 4,866 (S-BMD) subjects were available for GCTA analysis. For Generation R study a total of 5,908 subjects were genotyped by the Erasmus Medical Centre. Following QC 5,756 individuals had high quality genotyping data. Total-body DXA scans were performed on 6,509 subjects, of these a total of 6,490 passed DXA QC. High quality BMD and genetic data was available for 4,086 subjects. Of these 2,177 subjects were of Dutch-European decent. Two GWAS meta-analysis strategies were performed for each site. The first strategy involved all the subjects in the ALSPAC and the Generation-R studies. The second approach involved all the ALSPAC subjects, but was restricted to Generation R subjects who were of European descent. The number of subjects (n) involved in each step of the analysis is indicated. Ŧ = Number of subjects that had S-BMD measurements that passed QC. Φ = Number of Generation R subjects that were of Dutch-European descent. (TIF) [file pgen.1004423.s015.tif]
