# Supplementary material for: Phenotypic Dissection of Bone Mineral Density Reveals Skeletal Site Specificity and Facilitates the Identification of Novel Loci in the Genetic Regulation of Bone Mass Attainment
Source: PLoS Genet. 2014 Jun 19;10(6):e1004423. doi: 10.1371/journal.pgen.1004423 (PMC4063697; doi:10.1371/journal.pgen.1004423)
Supplement: Table S1 — Bivariate GCTA estimates of the genetic and residual correlations of age-, gender-, height- and weight-corrected bone mineral density measurements of the total-body less head, lower limb, upper limb and skull. (TBLH) = total-body less head, (LL-BMD) = lower limb BMD, (UL-BMD) = upper limb BMD, (SK-BMD) = skull BMD, rg = genetic correlation between trait 1 and trait 2. re = residual correlation between trait 1 and trait 2. All traits were adjusted for age, gender and height and weight. P-refers to the P-value for the likelihood ratio test of whether rg = 0. Phenotypic correlations (rp) were as follows: SK-BMD/TBLH-BMD (rp = 0.44, SE = 0.012, P<0.001), SK-BMD/LL-BMD (rp = 0.34, SE = 0.013, P<0.001), SK-BMD/UL-BMD (rp = 0.41, SE = 0.013, P<0.001) and LL-BMD/UL-BMD (rp = 0.64, SE = 0.010, P<0.001). (DOCX) [file pgen.1004423.s016.docx]

**Table S1**. Bivariate GCTA estimates of the genetic and residual correlations of age-, gender-, height- and weight-corrected bone mineral density measurements of the total-body less head, lower limb, upper limb and skull.

| **TRAIT 1** | **TRAIT2** | **SAMPLE SIZE** | **r_g_** | **SE** | **r_e_** | **SE** | ***P*** |
| --- | --- | --- | --- | --- | --- | --- | --- |
| **SK-BMD** | **TBLH-BMD** | 9732 | 0.56 | 0.082 | 0.31 | 0.085 | 4.78E-07 |
|  | **LL-BMD** | 9732 | 0.46 | 0.094 | 0.22 | 0.089 | 3.74E-05 |
|  | **UL-BMD** | 9732 | 0.58 | 0.088 | 0.25 | 0.085 | 6.65E-07 |
| **LL-BMD** | **UL-BMD** | 9782 | 0.80 | 0.063 | 0.53 | 0.058 | 1.51E-08 |

(TBLH) = total-body less head, (LL-BMD) = lower limb BMD, (UL-BMD) = upper limb BMD, (SK-BMD) = skull BMD, r_g_ = genetic correlation between trait 1 and trait 2. r_e_ = residual correlation between trait 1 and trait 2. All traits were adjusted for age, gender and height and weight. *P*-refers to the *P*-value for the likelihood ratio test of whether r_g_ = 0. Phenotypic correlations (r_p)_ were as follows: SK-BMD/TBLH-BMD (r_p_ = 0.44, SE = 0.012, P < 0.001), SK-BMD/LL-BMD (r_p_ = 0.34, SE = 0.013, P < 0.001), SK-BMD/UL-BMD (r_p_ = 0.41, SE = 0.013, P < 0.001) and LL-BMD/UL-BMD (r_p_ = 0.64, SE = 0.010, P < 0.001).
