# Supplementary material for: Phenotypic Dissection of Bone Mineral Density Reveals Skeletal Site Specificity and Facilitates the Identification of Novel Loci in the Genetic Regulation of Bone Mass Attainment
Source: PLoS Genet. 2014 Jun 19;10(6):e1004423. doi: 10.1371/journal.pgen.1004423 (PMC4063697; doi:10.1371/journal.pgen.1004423)
Supplement: Table S2 — Characteristics of BMD measures and other anthropometrical traits for participants of the ALSPAC and GEN-R cohorts. (TBLH-BMD) = total-body less head BMD; (LL-BMD) = lower limb BMD; (UL-BMD) = upper limb BMD; (SK-BMD) = skull BMD; UNIT = unit of measurement; n = number of subjects; SD = standard deviation of the mean value; (C-MEAN) = mean of trait measured for males and females; (F-MEAN) = mean of trait measured in females; (M-MEAN) = mean of trait measured in males. *Only 5299 subjects had skull BMD measurements. (DOCX) [file pgen.1004423.s017.docx]

**Table S2**. Characteristics of BMD measures and other anthropometrical traits for participants of the ALSPAC and GEN-R cohorts

|  |  | **ALSPAC** | | | | | | **Generation R** | | | | | |
| --- | --- | --- | --- | --- | --- | --- | --- | --- | --- | --- | --- | --- | --- |
| **TRAIT** | **UNIT** | **C-MEAN (n=5330)** | **SD** | **F-MEAN (n=2734)** | **SD** | **M-MEAN (n=2596)** | **SD** | **C-MEAN (n=4086)** | **SD** | **F-MEAN (n=2043)** | **SD** | **M-MEAN (n=2043)** | **SD** |
| **TBLH-BMD** | (g/cm^2^) | 0.78 | 0.05 | 0.77 | 0.05 | 0.78 | 0.05 | 0.55 | 0.05 | 0.55 | 0.05 | 0.56 | 0.05 |
| **LL-BMD** | (g/cm^2^) | 0.90 | 0.08 | 0.90 | 0.08 | 0.90 | 0.08 | 0.62 | 0.06 | 0.62 | 0.06 | 0.62 | 0.06 |
| **UL-BMD** | (g/cm^2^) | 0.65 | 0.04 | 0.65 | 0.04 | 0.66 | 0.04 | 0.41 | 0.04 | 0.41 | 0.04 | 0.42 | 0.04 |
| **SK-BMD*** | (g/cm^2^) | 1.57 | 0.14 | 1.56 | 0.14 | 1.59 | 0.14 | 1.35 | 0.13 | 1.32 | 0.13 | 1.38 | 0.13 |
| **AGE** | (years) | 9.94 | 0.32 | 9.94 | 0.32 | 9.94 | 0.32 | 6.22 | 0.50 | 6.21 | 0.48 | 6.22 | 0.52 |
| **HEIGHT** | (cm) | 139.65 | 6.36 | 139.41 | 6.52 | 139.91 | 6.19 | 119.60 | 5.90 | 119.20 | 5.94 | 120.00 | 5.84 |
| **WEIGHT** | (kg) | 34.73 | 7.42 | 35.00 | 7.62 | 34.45 | 7.20 | 23.07 | 4.08 | 22.99 | 4.25 | 23.14 | 3.90 |

(TBLH-BMD) = total-body less head BMD; (LL-BMD) = lower limb BMD; (UL-BMD) = upper limb BMD; (SK-BMD) = skull BMD; UNIT = unit of measurement; n= number of subjects; SD = standard deviation of the mean value; (C-MEAN) = mean of trait measured for males and females; (F-MEAN) = mean of trait measured in females; (M-MEAN) = mean of trait measured in males. ^*^Only 5299 subjects had skull BMD measurements.
