# Supplementary material for: Phenotypic Dissection of Bone Mineral Density Reveals Skeletal Site Specificity and Facilitates the Identification of Novel Loci in the Genetic Regulation of Bone Mass Attainment
Source: PLoS Genet. 2014 Jun 19;10(6):e1004423. doi: 10.1371/journal.pgen.1004423 (PMC4063697; doi:10.1371/journal.pgen.1004423)
Supplement: Table S3 — Overall and population specific characteristics of BMD measures and other anthropometric traits in Generation R. (TBLH-BMD) = total-body less head BMD; (LL-BMD) = lower limb BMD; (UL-BMD) = upper limb BMD; (SK-BMD) = skull BMD; n = number of individuals; MEAN = mean value of each trait; SD = standard deviation of the mean each trait; UNIT = unit of measurement. Please note that these classifications are based on self-reported ethnicity. (DOCX) [file pgen.1004423.s018.docx]

**Table S3**. Overall and population specific characteristics of BMD measures and other anthropometric traits in Generation R.

|  |  | **DUTCH-OTHER EUROPEAN (n=2677)** | | **SURINAM (n=289)** | | **TURKISH (n=300)** | | **MORROCAN (n=232)** | | **OTHER (n=588)** | | **COMBINED (n=4086)** | |
| --- | --- | --- | --- | --- | --- | --- | --- | --- | --- | --- | --- | --- | --- |
| **TRAIT** | **UNIT** | **MEAN** | **SD** | **MEAN** | **SD** | **MEAN** | **SD** | **MEAN** | **SD** | **MEAN** | **SD** | **MEAN** | **SD** |
| **TBLH-BMD** | g/cm^2^ | 0.55 | 0.05 | 0.56 | 0.06 | 0.55 | 0.05 | 0.56 | 0.05 | 0.57 | 0.08 | 0.55 | 0.05 |
| **LL-BMD** | g/cm^2^ | 0.61 | 0.06 | 0.64 | 0.07 | 0.62 | 0.06 | 0.63 | 0.07 | 0.65 | 0.06 | 0.62 | 0.06 |
| **UL-BMD** | g/cm^2^ | 0.41 | 0.03 | 0.41 | 0.04 | 0.41 | 0.04 | 0.41 | 0.04 | 0.42 | 0.04 | 0.41 | 0.04 |
| **SK-BMD** | g/cm^2^ | 1.36 | 0.13 | 1.33 | 0.14 | 1.31 | 0.12 | 1.34 | 0.13 | 1.35 | 0.14 | 1.35 | 0.13 |
| **AGE** | (years) | 6.16 | 0.45 | 6.30 | 0.58 | 6.77 | 0.51 | 6.35 | 0.57 | 6.33 | 0.62 | 6.22 | 0.50 |
| **HEIGHT** | (cm) | 119.60 | 5.59 | 120.10 | 6.81 | 119.10 | 5.62 | 119.10 | 5.85 | 120.10 | 6.86 | 119.60 | 0.59 |
| **WEIGHT** | (kg) | 22.71 | 3.54 | 23.31 | 5.13 | 24.19 | 5.18 | 23.62 | 4.09 | 23.85 | 4.82 | 23.07 | 4.08 |

(TBLH-BMD) = total-body less head BMD; (LL-BMD) = lower limb BMD; (UL-BMD) = upper limb BMD; (SK-BMD) = skull BMD; n= number of individuals; MEAN = mean value of each trait; SD = standard deviation of the mean each trait; UNIT = unit of measurement. Please note that these classifications are based on self reported ethnicity.
