# Supplementary material for: Phenotypic Dissection of Bone Mineral Density Reveals Skeletal Site Specificity and Facilitates the Identification of Novel Loci in the Genetic Regulation of Bone Mass Attainment
Source: PLoS Genet. 2014 Jun 19;10(6):e1004423. doi: 10.1371/journal.pgen.1004423 (PMC4063697; doi:10.1371/journal.pgen.1004423)
Supplement: Table S4 — Genome-wide associated TBLH-BMD variants. (CHR) = chromosome number; (POS) = position in the genome based on hg18; (EAF) = effect allele frequency; (β) = estimates of effect size expressed as adjusted SD per copy of the effect allele (EA); (SE) = standard error of β; (P) = P-value; (I2) = Cochran's Q statistic evaluating heterogeneity and (P HET) = evidence of heterogeneity. The SNP that showed the strongest evidence of association at each locus is displayed in bold font. (DOCX) [file pgen.1004423.s019.docx]

**Table S4**. Genome-wide associated TBLH-BMD variants.

|  |  |  |  | **ALSPAC (n=5330)** | | | | **Generation R (n=4086)** | | | | **META-ANALYSIS (n=9416)** | | | | | |
| --- | --- | --- | --- | --- | --- | --- | --- | --- | --- | --- | --- | --- | --- | --- | --- | --- | --- |
| **RSID** | **CHR** | **POS** | **EA** | **EAF** | ***β*** | **SE** | ***P*** | **EAF** | ***β*** | **SE** | ***P*** | **EAF** | ***β*** | **SE** | ***P*** | **I^2^** | ***P*_HET_** |
| rs2268177 | 1 | 22287997 | A | 0.83 | 0.10 | 0.03 | 5.8E-05 | 0.84 | 0.12 | 0.03 | 3.7E-05 | 0.83 | 0.11 | 0.02 | 1.5E-08 | 0 | 6.7E-01 |
| rs7412010 | 1 | 22309033 | G | 0.84 | 0.11 | 0.03 | 2.8E-05 | 0.85 | 0.13 | 0.03 | 2.3E-05 | 0.84 | 0.12 | 0.02 | 2.6E-09 | 0 | 6.4E-01 |
| **rs3765350** | **1** | **22319903** | **A** | **0.78** | **0.11** | **0.02** | **5.7E-06** | **0.78** | **0.11** | **0.03** | **2.9E-05** | **0.78** | **0.11** | **0.02** | **7.0E-10** | **0** | **9.3E-01** |
| rs2235529 | 1 | 22323074 | C | 0.84 | 0.12 | 0.03 | 8.0E-06 | 0.85 | 0.13 | 0.03 | 1.6E-05 | 0.85 | 0.13 | 0.02 | 1.2E-09 | 0 | 7.4E-01 |
| rs3820282 | 1 | 22340802 | C | 0.84 | 0.11 | 0.03 | 3.0E-05 | 0.85 | 0.14 | 0.03 | 8.6E-06 | 0.85 | 0.12 | 0.02 | 2.0E-09 | 0 | 5.3E-01 |
| rs12042083 | 1 | 22345319 | G | 0.79 | 0.11 | 0.02 | 2.8E-06 | 0.77 | 0.09 | 0.03 | 7.0E-04 | 0.78 | 0.10 | 0.02 | 1.1E-08 | 0 | 5.5E-01 |
| rs7515106 | 1 | 22345997 | T | 0.79 | 0.11 | 0.02 | 2.8E-06 | 0.74 | 0.09 | 0.03 | 4.4E-04 | 0.77 | 0.10 | 0.02 | 7.8E-09 | 0 | 5.2E-01 |
| rs777346 | 2 | 166244164 | C | 0.52 | 0.09 | 0.02 | 2.3E-06 | 0.56 | 0.08 | 0.02 | 9.1E-04 | 0.54 | 0.09 | 0.02 | 2.4E-08 | 0 | 5.6E-01 |
| rs2194754 | 2 | 166247288 | A | 0.51 | 0.09 | 0.02 | 7.5E-06 | 0.55 | 0.08 | 0.02 | 7.2E-04 | 0.53 | 0.08 | 0.02 | 4.8E-08 | 0 | 6.7E-01 |
| rs777355 | 2 | 166262283 | G | 0.51 | 0.09 | 0.02 | 1.5E-06 | 0.54 | 0.08 | 0.02 | 3.4E-04 | 0.53 | 0.09 | 0.01 | 2.7E-09 | 0 | 6.1E-01 |
| rs1863196 | 2 | 166265756 | A | 0.51 | 0.09 | 0.02 | 3.5E-06 | 0.58 | 0.07 | 0.02 | 8.4E-04 | 0.54 | 0.08 | 0.02 | 2.7E-08 | 0 | 5.7E-01 |
| rs12185748 | 2 | 166285202 | C | 0.51 | 0.09 | 0.02 | 1.3E-06 | 0.58 | 0.09 | 0.02 | 8.9E-05 | 0.54 | 0.09 | 0.01 | 3.9E-10 | 0 | 8.1E-01 |
| rs7586085 | 2 | 166285735 | A | 0.51 | 0.09 | 0.02 | 1.3E-06 | 0.58 | 0.08 | 0.02 | 1.5E-04 | 0.54 | 0.09 | 0.01 | 7.0E-10 | 0 | 7.3E-01 |
| **rs6726821** | **2** | **166286360** | **T** | **0.51** | **0.09** | **0.02** | **1.3E-06** | **0.58** | **0.09** | **0.02** | **8.8E-05** | **0.54** | **0.09** | **0.01** | **3.9E-10** | **0** | **8.1E-01** |
| rs6710388 | 2 | 166291387 | C | 0.51 | 0.09 | 0.02 | 1.3E-06 | 0.58 | 0.08 | 0.02 | 1.1E-04 | 0.54 | 0.09 | 0.01 | 7.0E-10 | 0 | 7.3E-01 |
| rs6710518 | 2 | 166291490 | C | 0.51 | 0.09 | 0.02 | 1.3E-06 | 0.58 | 0.08 | 0.02 | 1.1E-04 | 0.54 | 0.09 | 0.01 | 7.0E-10 | 0 | 7.3E-01 |
| rs1346003 | 2 | 166305603 | C | 0.51 | 0.09 | 0.02 | 3.6E-06 | 0.58 | 0.09 | 0.02 | 1.1E-04 | 0.54 | 0.09 | 0.02 | 4.5E-09 | 0 | 8.7E-01 |
| rs1346004 | 2 | 166309292 | G | 0.51 | 0.09 | 0.02 | 3.7E-06 | 0.58 | 0.09 | 0.02 | 1.2E-04 | 0.54 | 0.09 | 0.02 | 4.5E-09 | 0 | 8.7E-01 |
| rs1895701 | 2 | 166311333 | C | 0.49 | 0.10 | 0.02 | 1.3E-06 | 0.53 | 0.07 | 0.02 | 2.6E-03 | 0.51 | 0.08 | 0.02 | 4.1E-08 | 0 | 3.5E-01 |
| rs13430211 | 2 | 166330844 | C | 0.50 | 0.09 | 0.02 | 1.5E-05 | 0.57 | 0.08 | 0.02 | 5.0E-04 | 0.53 | 0.08 | 0.02 | 4.4E-08 | 0 | 7.6E-01 |
| rs2110281 | 7 | 120513045 | G | 0.64 | 0.06 | 0.02 | 3.0E-03 | 0.65 | 0.11 | 0.02 | 1.2E-06 | 0.64 | 0.08 | 0.02 | 4.7E-08 | 66.4 | 8.5E-02 |
| rs798943 | 7 | 120546135 | G | 0.61 | 0.06 | 0.02 | 2.6E-03 | 0.62 | 0.12 | 0.02 | 9.2E-08 | 0.61 | 0.09 | 0.02 | 1.3E-08 | 74.6 | 4.7E-02 |
| rs7801723 | 7 | 120561396 | C | 0.61 | 0.06 | 0.02 | 2.6E-03 | 0.63 | 0.12 | 0.02 | 7.8E-08 | 0.62 | 0.09 | 0.02 | 1.1E-08 | 75.4 | 4.4E-02 |
| rs12706318 | 7 | 120562177 | A | 0.61 | 0.06 | 0.02 | 2.7E-03 | 0.62 | 0.12 | 0.02 | 1.6E-07 | 0.62 | 0.09 | 0.02 | 2.1E-08 | 71.9 | 5.9E-02 |
| rs13232048 | 7 | 120563517 | G | 0.61 | 0.06 | 0.02 | 2.7E-03 | 0.62 | 0.12 | 0.02 | 1.6E-07 | 0.62 | 0.09 | 0.02 | 2.1E-08 | 71.9 | 5.9E-02 |
| rs6952113 | 7 | 120564855 | G | 0.61 | 0.06 | 0.02 | 2.8E-03 | 0.62 | 0.12 | 0.02 | 2.0E-07 | 0.62 | 0.09 | 0.02 | 2.1E-08 | 71.9 | 5.9E-02 |
| rs872007 | 7 | 120567185 | C | 0.61 | 0.06 | 0.02 | 2.9E-03 | 0.62 | 0.12 | 0.02 | 1.9E-07 | 0.62 | 0.09 | 0.02 | 2.6E-08 | 72.9 | 5.5E-02 |
| rs10275439 | 7 | 120570661 | G | 0.62 | 0.06 | 0.02 | 3.6E-03 | 0.61 | 0.12 | 0.02 | 1.8E-07 | 0.62 | 0.08 | 0.02 | 3.2E-08 | 73.8 | 5.1E-02 |
| rs10261671 | 7 | 120570787 | C | 0.61 | 0.06 | 0.02 | 2.5E-03 | 0.61 | 0.12 | 0.02 | 2.7E-07 | 0.61 | 0.08 | 0.02 | 2.9E-08 | 69.9 | 6.8E-02 |
| rs13245690 | 7 | 120572300 | A | 0.61 | 0.06 | 0.02 | 2.4E-03 | 0.62 | 0.12 | 0.02 | 9.4E-08 | 0.62 | 0.09 | 0.02 | 1.0E-08 | 73.8 | 5.1E-02 |
| rs6950680 | 7 | 120577523 | A | 0.61 | 0.06 | 0.02 | 3.0E-03 | 0.63 | 0.12 | 0.02 | 6.3E-08 | 0.62 | 0.09 | 0.02 | 1.3E-08 | 76.2 | 4.0E-02 |
| rs3779381 | 7 | 120754026 | G | 0.26 | 0.12 | 0.02 | 9.9E-08 | 0.26 | 0.16 | 0.03 | 7.1E-10 | 0.26 | 0.14 | 0.02 | 4.6E-16 | 0 | 3.2E-01 |
| rs2908004 | 7 | 120757005 | A | 0.44 | 0.12 | 0.02 | 1.3E-09 | 0.50 | 0.14 | 0.02 | 2.5E-10 | 0.47 | 0.13 | 0.02 | 2.9E-18 | 0 | 5.5E-01 |
| rs2536189 | 7 | 120760857 | G | 0.44 | 0.12 | 0.02 | 1.3E-09 | 0.50 | 0.14 | 0.02 | 3.6E-10 | 0.47 | 0.13 | 0.02 | 3.8E-18 | 0 | 5.7E-01 |
| rs3801387 | 7 | 120762001 | G | 0.27 | 0.13 | 0.02 | 3.3E-09 | 0.27 | 0.17 | 0.03 | 3.3E-12 | 0.27 | 0.15 | 0.02 | 5.4E-19 | 41.7 | 1.9E-01 |
| rs2707466 | 7 | 120766325 | T | 0.42 | 0.12 | 0.02 | 1.1E-08 | 0.49 | 0.14 | 0.02 | 1.3E-09 | 0.45 | 0.13 | 0.02 | 1.9E-16 | 0 | 5.8E-01 |
| rs2536182 | 7 | 120778073 | G | 0.45 | 0.12 | 0.02 | 3.1E-09 | 0.47 | 0.14 | 0.02 | 9.0E-10 | 0.46 | 0.13 | 0.02 | 1.4E-16 | 0 | 4.5E-01 |
| rs2536180 | 7 | 120781909 | C | 0.46 | 0.11 | 0.02 | 5.7E-09 | 0.49 | 0.13 | 0.02 | 3.4E-09 | 0.48 | 0.12 | 0.01 | 9.3E-17 | 0 | 5.4E-01 |
| rs3801382 | 7 | 120785513 | G | 0.27 | 0.13 | 0.02 | 3.4E-09 | 0.27 | 0.18 | 0.03 | 2.1E-12 | 0.27 | 0.15 | 0.02 | 3.4E-19 | 46.7 | 1.7E-01 |
| rs2254595 | 7 | 120794485 | C | 0.46 | 0.11 | 0.02 | 5.9E-09 | 0.50 | 0.13 | 0.02 | 3.6E-09 | 0.48 | 0.12 | 0.01 | 1.5E-16 | 0 | 5.9E-01 |
| rs917727 | 7 | 120805815 | T | 0.27 | 0.13 | 0.02 | 3.5E-09 | 0.30 | 0.18 | 0.03 | 1.0E-12 | 0.28 | 0.16 | 0.02 | 4.4E-19 | 51 | 1.5E-01 |
| rs917726 | 7 | 120806093 | T | 0.27 | 0.13 | 0.02 | 3.5E-09 | 0.28 | 0.18 | 0.03 | 1.5E-12 | 0.28 | 0.16 | 0.02 | 5.3E-19 | 44.5 | 1.8E-01 |
| rs718766 | 7 | 120812738 | C | 0.27 | 0.13 | 0.02 | 3.6E-09 | 0.27 | 0.18 | 0.03 | 2.3E-12 | 0.27 | 0.16 | 0.02 | 5.3E-19 | 44.5 | 1.8E-01 |
| rs4727924 | 7 | 120819115 | T | 0.46 | 0.12 | 0.02 | 7.1E-09 | 0.47 | 0.16 | 0.02 | 5.5E-12 | 0.46 | 0.14 | 0.02 | 1.0E-18 | 53.1 | 1.4E-01 |
| **rs7776725** | **7** | **120820357** | **C** | **0.27** | **0.14** | **0.02** | **3.6E-09** | **0.26** | **0.19** | **0.03** | **7.7E-13** | **0.27** | **0.16** | **0.02** | **5.7E-20** | **54.7** | **1.4E-01** |
| **rs7466269** | **9** | **132453905** | **A** | **0.64** | **0.09** | **0.02** | **3.7E-06** | **0.66** | **0.07** | **0.02** | **2.0E-03** | **0.65** | **0.08** | **0.02** | **3.3E-08** | **0** | **4.7E-01** |
| **rs4420311** | **12** | **27875457** | **G** | **0.47** | **0.08** | **0.02** | **7.8E-05** | **0.44** | **0.09** | **0.02** | **1.0E-04** | **0.46** | **0.09** | **0.02** | **4.4E-08** | **0** | **7.0E-01** |
| rs17458078 | 13 | 42009354 | C | 0.45 | 0.07 | 0.02 | 1.4E-04 | 0.47 | 0.09 | 0.02 | 3.1E-05 | 0.46 | 0.08 | 0.02 | 3.1E-08 | 0 | 5.3E-01 |
| rs9533143 | 13 | 42009405 | C | 0.45 | 0.08 | 0.02 | 1.4E-04 | 0.47 | 0.09 | 0.02 | 3.1E-05 | 0.46 | 0.08 | 0.02 | 2.5E-08 | 0 | 5.5E-01 |
| rs1021189 | 13 | 42014211 | C | 0.45 | 0.08 | 0.02 | 1.4E-04 | 0.43 | 0.09 | 0.02 | 5.1E-05 | 0.44 | 0.08 | 0.02 | 4.3E-08 | 0 | 6.2E-01 |
| rs9525638 | 13 | 42026577 | C | 0.43 | 0.08 | 0.02 | 6.5E-05 | 0.41 | 0.09 | 0.02 | 2.6E-05 | 0.42 | 0.08 | 0.02 | 1.3E-08 | 0 | 6.2E-01 |
| rs1325798 | 13 | 42037049 | T | 0.43 | 0.08 | 0.02 | 6.5E-05 | 0.40 | 0.09 | 0.02 | 2.1E-05 | 0.42 | 0.09 | 0.02 | 1.1E-08 | 0 | 5.9E-01 |
| rs9533154 | 13 | 42038102 | T | 0.43 | 0.08 | 0.02 | 6.5E-05 | 0.41 | 0.09 | 0.02 | 3.2E-05 | 0.42 | 0.08 | 0.02 | 1.9E-08 | 0 | 6.6E-01 |
| **rs17536328** | **13** | **42041029** | **T** | **0.43** | **0.08** | **0.02** | **6.1E-05** | **0.40** | **0.10** | **0.02** | **1.5E-05** | **0.42** | **0.09** | **0.02** | **7.6E-09** | **0** | **5.9E-01** |
| rs7325635 | 13 | 42043319 | A | 0.43 | 0.08 | 0.02 | 7.5E-05 | 0.41 | 0.09 | 0.02 | 2.5E-05 | 0.42 | 0.08 | 0.02 | 1.3E-08 | 0 | 6.2E-01 |
| rs9525643 | 13 | 42057516 | C | 0.43 | 0.08 | 0.02 | 7.7E-05 | 0.40 | 0.09 | 0.02 | 2.2E-05 | 0.42 | 0.09 | 0.02 | 1.1E-08 | 0 | 5.9E-01 |
| rs9525644 | 13 | 42057804 | A | 0.45 | 0.06 | 0.02 | 1.4E-03 | 0.45 | 0.11 | 0.02 | 1.6E-06 | 0.45 | 0.08 | 0.02 | 3.0E-08 | 51.3 | 1.5E-01 |
| rs1054016 | 13 | 42080002 | T | 0.43 | 0.07 | 0.02 | 2.1E-04 | 0.40 | 0.09 | 0.02 | 4.5E-05 | 0.42 | 0.08 | 0.02 | 3.7E-08 | 0 | 5.5E-01 |
| rs8010344 | 14 | 92156671 | A | 0.82 | 0.09 | 0.03 | 3.8E-04 | 0.85 | 0.13 | 0.03 | 2.2E-05 | 0.83 | 0.11 | 0.02 | 5.0E-08 | 12.9 | 2.8E-01 |
| rs11623779 | 14 | 92166144 | T | 0.82 | 0.09 | 0.03 | 3.5E-04 | 0.85 | 0.14 | 0.03 | 1.6E-05 | 0.83 | 0.11 | 0.02 | 3.9E-08 | 20.5 | 2.6E-01 |
| rs11627441 | 14 | 92168987 | T | 0.82 | 0.09 | 0.03 | 3.4E-04 | 0.84 | 0.13 | 0.03 | 2.4E-05 | 0.83 | 0.11 | 0.02 | 4.9E-08 | 0 | 3.3E-01 |
| rs17184313 | 14 | 92172004 | C | 0.82 | 0.09 | 0.03 | 2.9E-04 | 0.85 | 0.13 | 0.03 | 1.7E-05 | 0.83 | 0.11 | 0.02 | 4.2E-08 | 8.7 | 3.0E-01 |
| rs10498635 | 14 | 92173062 | C | 0.82 | 0.09 | 0.03 | 2.8E-04 | 0.85 | 0.13 | 0.03 | 1.7E-05 | 0.83 | 0.11 | 0.02 | 4.2E-08 | 8.7 | 3.0E-01 |
| rs1075472 | 14 | 92177884 | A | 0.81 | 0.09 | 0.03 | 1.7E-04 | 0.85 | 0.14 | 0.03 | 2.0E-05 | 0.83 | 0.11 | 0.02 | 3.5E-08 | 0.5 | 3.2E-01 |
| **rs754388** | **14** | **92185163** | **C** | **0.81** | **0.10** | **0.03** | **1.3E-04** | **0.83** | **0.15** | **0.03** | **1.4E-06** | **0.82** | **0.12** | **0.02** | **3.0E-09** | **36** | **2.1E-01** |

(CHR) = chromosome number; (POS) = position in the genome based on hg18; (EAF) = effect allele frequency; (*β*) = estimates of effect size expressed as adjusted SD per copy of the effect allele (EA); (SE) = standard error of *β*; (*P*) = *P*-value; (I^2^) = Cochran’s Q statistic evaluating heterogeneity and (*P*_HET_) = evidence of heterogeneity. The SNP that showed the strongest evidence of association at each locus is displayed in bold font.
