# Supplementary material for: Phenotypic Dissection of Bone Mineral Density Reveals Skeletal Site Specificity and Facilitates the Identification of Novel Loci in the Genetic Regulation of Bone Mass Attainment
Source: PLoS Genet. 2014 Jun 19;10(6):e1004423. doi: 10.1371/journal.pgen.1004423 (PMC4063697; doi:10.1371/journal.pgen.1004423)
Supplement: Table S5 — Genome-wide associated LL-BMD variants. (CHR) = chromosome number; (POS) = position in the genome based on hg18; (EAF) = effect allele frequency; (β) = estimates of effect size expressed as adjusted SD per copy of the effect allele (EA); (SE) = standard error of β; (P) = P-value; (I2) = Cochran's Q statistic evaluating heterogeneity and (PHET) = evidence of heterogeneity. The SNP that showed the strongest evidence of association at each locus is displayed in bold font. (DOCX) [file pgen.1004423.s020.docx]

**Table S5**. Genome-wide associated LL-BMD variants.

|  |  |  |  | **ALSPAC (n=5330)** | | | | **Generation R (n=4086)** | | | | **META-ANALYSIS (n=9416)** | | | | | |
| --- | --- | --- | --- | --- | --- | --- | --- | --- | --- | --- | --- | --- | --- | --- | --- | --- | --- |
| **RSID** | **CHR** | **POS** | **EA** | **EAF** | ***β*** | **SE** | ***P*** | **EAF** | ***β*** | **SE** | ***P*** | **EAF** | ***β*** | **SE** | ***P*** | **I^2^** | ***P*_HET_** |
| **rs3765350** | **1** | **22319903** | **A** | **0.78** | **0.10** | **0.02** | **1.1E-05** | **0.78** | **0.09** | **0.03** | **5.7E-04** | **0.78** | **0.10** | **0.02** | **2.9E-08** | **0** | **7.1E-01** |
| **rs2908004** | **7** | **120757005** | **A** | **0.44** | **0.09** | **0.02** | **3.6E-06** | **0.50** | **0.11** | **0.02** | **1.3E-06** | **0.47** | **0.10** | **0.02** | **3.0E-11** | **0** | **6.2E-01** |
| rs2536189 | 7 | 120760857 | G | 0.44 | 0.09 | 0.02 | 3.6E-06 | 0.50 | 0.11 | 0.02 | 1.3E-06 | 0.47 | 0.10 | 0.02 | 3.7E-11 | 0 | 6.4E-01 |
| rs3801387 | 7 | 120762001 | G | 0.27 | 0.09 | 0.02 | 2.1E-05 | 0.27 | 0.10 | 0.03 | 4.4E-05 | 0.27 | 0.10 | 0.02 | 8.8E-09 | 0 | 8.1E-01 |
| rs2707466 | 7 | 120766325 | T | 0.42 | 0.09 | 0.02 | 9.8E-06 | 0.49 | 0.11 | 0.02 | 1.9E-06 | 0.45 | 0.10 | 0.02 | 1.9E-10 | 0 | 6.3E-01 |
| rs2536182 | 7 | 120778073 | G | 0.45 | 0.09 | 0.02 | 9.1E-06 | 0.47 | 0.10 | 0.02 | 1.2E-05 | 0.46 | 0.09 | 0.02 | 1.5E-09 | 0 | 6.7E-01 |
| rs2536180 | 7 | 120781909 | C | 0.46 | 0.09 | 0.02 | 1.1E-05 | 0.49 | 0.10 | 0.02 | 7.9E-06 | 0.48 | 0.09 | 0.01 | 4.6E-10 | 0 | 6.3E-01 |
| rs3801382 | 7 | 120785513 | G | 0.27 | 0.09 | 0.02 | 2.2E-05 | 0.27 | 0.10 | 0.03 | 3.9E-05 | 0.27 | 0.10 | 0.02 | 7.5E-09 | 0 | 7.9E-01 |
| rs2254595 | 7 | 120794485 | C | 0.46 | 0.09 | 0.02 | 1.1E-05 | 0.50 | 0.10 | 0.02 | 7.9E-06 | 0.48 | 0.09 | 0.01 | 6.7E-10 | 0 | 6.8E-01 |
| rs917727 | 7 | 120805815 | T | 0.27 | 0.10 | 0.02 | 2.4E-05 | 0.30 | 0.11 | 0.03 | 3.2E-05 | 0.28 | 0.10 | 0.02 | 9.9E-09 | 0 | 7.3E-01 |
| rs917726 | 7 | 120806093 | T | 0.27 | 0.10 | 0.02 | 2.4E-05 | 0.28 | 0.11 | 0.03 | 3.2E-05 | 0.28 | 0.10 | 0.02 | 8.2E-09 | 0 | 7.5E-01 |
| rs718766 | 7 | 120812738 | C | 0.27 | 0.10 | 0.02 | 2.4E-05 | 0.27 | 0.11 | 0.03 | 3.6E-05 | 0.27 | 0.10 | 0.02 | 8.2E-09 | 0 | 7.5E-01 |
| rs4727924 | 7 | 120819115 | T | 0.46 | 0.09 | 0.02 | 1.4E-05 | 0.47 | 0.12 | 0.02 | 5.7E-07 | 0.46 | 0.10 | 0.02 | 7.9E-11 | 0 | 3.4E-01 |
| rs7776725 | 7 | 120820357 | C | 0.27 | 0.10 | 0.02 | 2.4E-05 | 0.26 | 0.11 | 0.03 | 2.0E-05 | 0.27 | 0.10 | 0.02 | 3.1E-09 | 0 | 6.7E-01 |
| **rs7466269** | **9** | **132453905** | **A** | **0.64** | **0.10** | **0.02** | **1.8E-06** | **0.66** | **0.07** | **0.02** | **1.6E-03** | **0.65** | **0.09** | **0.02** | **1.5E-08** | **0** | **4.6E-01** |
| **rs4420311** | **12** | **27875457** | **G** | **0.47** | **0.09** | **0.02** | **2.1E-05** | **0.44** | **0.09** | **0.02** | **2.3E-04** | **0.46** | **0.09** | **0.02** | **3.2E-08** | **0** | **9.7E-01** |
| rs8010344 | 14 | 92156671 | A | 0.82 | 0.11 | 0.03 | 1.6E-05 | 0.85 | 0.14 | 0.03 | 9.2E-06 | 0.83 | 0.12 | 0.02 | 9.5E-10 | 0 | 4.6E-01 |
| rs2181378 | 14 | 92161250 | A | 0.64 | 0.08 | 0.02 | 1.2E-04 | 0.60 | 0.10 | 0.02 | 3.8E-05 | 0.62 | 0.09 | 0.02 | 2.5E-08 | 0 | 5.8E-01 |
| rs11623779 | 14 | 92166144 | T | 0.82 | 0.11 | 0.03 | 1.4E-05 | 0.85 | 0.14 | 0.03 | 6.8E-06 | 0.83 | 0.12 | 0.02 | 7.4E-10 | 0 | 4.3E-01 |
| rs11621587 | 14 | 92168092 | G | 0.82 | 0.11 | 0.03 | 1.4E-05 | 0.86 | 0.15 | 0.03 | 6.2E-06 | 0.83 | 0.12 | 0.02 | 8.5E-10 | 0 | 3.8E-01 |
| rs11627441 | 14 | 92168987 | T | 0.82 | 0.11 | 0.03 | 1.4E-05 | 0.84 | 0.14 | 0.03 | 5.0E-06 | 0.83 | 0.12 | 0.02 | 4.8E-10 | 0 | 4.5E-01 |
| rs7146689 | 14 | 92169777 | T | 0.82 | 0.11 | 0.03 | 1.3E-05 | 0.83 | 0.13 | 0.03 | 1.1E-05 | 0.82 | 0.12 | 0.02 | 1.1E-09 | 0 | 5.4E-01 |
| rs17184313 | 14 | 92172004 | C | 0.82 | 0.11 | 0.03 | 1.1E-05 | 0.85 | 0.14 | 0.03 | 6.7E-06 | 0.83 | 0.12 | 0.02 | 7.9E-10 | 0 | 4.7E-01 |
| rs10498635 | 14 | 92173062 | C | 0.82 | 0.11 | 0.03 | 1.1E-05 | 0.85 | 0.14 | 0.03 | 6.6E-06 | 0.83 | 0.12 | 0.02 | 7.9E-10 | 0 | 4.7E-01 |
| rs11627032 | 14 | 92173825 | T | 0.75 | 0.09 | 0.02 | 1.5E-04 | 0.76 | 0.10 | 0.03 | 6.8E-05 | 0.75 | 0.09 | 0.02 | 5.0E-08 | 0 | 5.8E-01 |
| rs1075472 | 14 | 92177884 | A | 0.81 | 0.11 | 0.03 | 6.4E-06 | 0.85 | 0.14 | 0.03 | 1.0E-05 | 0.83 | 0.12 | 0.02 | 7.3E-10 | 0 | 5.1E-01 |
| rs11624512 | 14 | 92180873 | C | 0.81 | 0.12 | 0.03 | 3.1E-06 | 0.85 | 0.13 | 0.03 | 9.7E-05 | 0.83 | 0.12 | 0.02 | 2.1E-09 | 0 | 9.3E-01 |
| **rs754388** | **14** | **92185163** | **C** | **0.81** | **0.12** | **0.03** | **3.5E-06** | **0.83** | **0.15** | **0.03** | **2.5E-06** | **0.82** | **0.13** | **0.02** | **1.4E-10** | **0** | **5.3E-01** |

(CHR) = chromosome number; (POS) = position in the genome based on hg18; (EAF) = effect allele frequency; (β) = estimates of effect size expressed as adjusted SD per copy of the effect allele (EA); (SE) = standard error of β; (P) = P-value; (I2) = Cochran’s Q statistic evaluating heterogeneity and (P_HET_) = evidence of heterogeneity. The SNP that showed the strongest evidence of association at each locus is displayed in bold font.
