# Supplementary material for: Phenotypic Dissection of Bone Mineral Density Reveals Skeletal Site Specificity and Facilitates the Identification of Novel Loci in the Genetic Regulation of Bone Mass Attainment
Source: PLoS Genet. 2014 Jun 19;10(6):e1004423. doi: 10.1371/journal.pgen.1004423 (PMC4063697; doi:10.1371/journal.pgen.1004423)
Supplement: Table S6 — Genome-wide associated UL-BMD variants. (CHR) = chromosome number; (POS) = position in the genome based on hg18; (EAF) = effect allele frequency; (β) = estimates of effect size expressed as adjusted SD per copy of the effect allele (EA); (SE) = standard error of β; (P) = P-value; (I2) = Cochran's Q statistic evaluating heterogeneity and (P HET) = evidence of heterogeneity. The SNP that showed the strongest evidence of association at each locus is displayed in bold font. (DOCX) [file pgen.1004423.s021.docx]

**Table S6**. Genome-wide associated UL-BMD variants.

|  |  |  |  | **ALSPAC (n=5330)** | | | | **Generation R (n=4086)** | | | | **META-ANALYSIS (n=9416)** | | | | | |
| --- | --- | --- | --- | --- | --- | --- | --- | --- | --- | --- | --- | --- | --- | --- | --- | --- | --- |
| **RSID** | **CHR** | **POS** | **EA** | **EAF** | ***β*** | **SE** | ***P*** | **EAF** | ***β*** | **SE** | ***P*** | **EAF** | ***β*** | **SE** | ***P*** | **I^2^** | ***P*_HET_** |
| rs7412010 | 1 | 22309033 | G | 0.84 | 0.09 | 0.03 | 4.8E-04 | 0.85 | 0.14 | 0.03 | 9.5E-06 | 0.84 | 0.11 | 0.02 | 2.3E-08 | 13.7 | 2.8E-01 |
| **rs2235529** | **1** | **22323074** | **C** | **0.84** | **0.10** | **0.03** | **2.0E-04** | **0.85** | **0.14** | **0.03** | **6.0E-06** | **0.85** | **0.12** | **0.02** | **1.2E-08** | **0** | **3.2E-01** |
| rs3820282 | 1 | 22340802 | C | 0.84 | 0.09 | 0.03 | 4.7E-04 | 0.85 | 0.14 | 0.03 | 5.0E-06 | 0.85 | 0.12 | 0.02 | 2.1E-08 | 25.7 | 2.5E-01 |
| rs777355 | 2 | 166262283 | G | 0.51 | 0.08 | 0.02 | 7.5E-05 | 0.54 | 0.08 | 0.02 | 1.4E-04 | 0.53 | 0.08 | 0.01 | 4.0E-08 | 0 | 8.4E-01 |
| rs12185748 | 2 | 166285202 | C | 0.51 | 0.08 | 0.02 | 6.3E-05 | 0.58 | 0.09 | 0.02 | 5.6E-05 | 0.54 | 0.08 | 0.01 | 1.1E-08 | 0 | 7.1E-01 |
| rs7586085 | 2 | 166285735 | A | 0.51 | 0.08 | 0.02 | 6.4E-05 | 0.58 | 0.09 | 0.02 | 7.0E-05 | 0.54 | 0.08 | 0.01 | 1.3E-08 | 0 | 7.3E-01 |
| **rs6726821** | **2** | **166286360** | **T** | **0.51** | **0.08** | **0.02** | **6.4E-05** | **0.58** | **0.09** | **0.02** | **5.6E-05** | **0.54** | **0.08** | **0.01** | **1.1E-08** | **0** | **7.1E-01** |
| rs6710388 | 2 | 166291387 | C | 0.51 | 0.08 | 0.02 | 6.5E-05 | 0.58 | 0.09 | 0.02 | 8.5E-05 | 0.54 | 0.08 | 0.01 | 1.9E-08 | 0 | 7.8E-01 |
| rs6710518 | 2 | 166291490 | C | 0.51 | 0.08 | 0.02 | 6.5E-05 | 0.58 | 0.09 | 0.02 | 8.5E-05 | 0.54 | 0.08 | 0.01 | 1.9E-08 | 0 | 7.8E-01 |
| **rs1262476** | **6** | **127028689** | **G** | **0.76** | **0.13** | **0.02** | **6.4E-09** | **0.79** | **0.06** | **0.03** | **2.4E-02** | **0.77** | **0.10** | **0.02** | **2.9E-09** | **72.3** | **5.8E-02** |
| rs13212044 | 6 | 127067354 | G | 0.76 | 0.13 | 0.02 | 6.2E-09 | 0.80 | 0.06 | 0.03 | 2.6E-02 | 0.77 | 0.10 | 0.02 | 3.4E-09 | 73.1 | 5.4E-02 |
| rs10484759 | 6 | 127078735 | C | 0.76 | 0.13 | 0.02 | 6.1E-09 | 0.81 | 0.05 | 0.03 | 6.1E-02 | 0.78 | 0.10 | 0.02 | 1.1E-08 | 78.9 | 2.9E-02 |
| rs13194508 | 6 | 127186376 | T | 0.76 | 0.14 | 0.02 | 1.3E-08 | 0.83 | 0.06 | 0.03 | 4.8E-02 | 0.79 | 0.11 | 0.02 | 2.4E-08 | 74.8 | 4.6E-02 |
| rs17563605 | 6 | 127206265 | T | 0.76 | 0.14 | 0.02 | 8.6E-09 | 0.82 | 0.06 | 0.03 | 5.2E-02 | 0.79 | 0.11 | 0.02 | 1.9E-08 | 76.7 | 3.8E-02 |
| rs13204965 | 6 | 127208765 | A | 0.76 | 0.14 | 0.02 | 7.9E-09 | 0.83 | 0.06 | 0.03 | 4.6E-02 | 0.79 | 0.11 | 0.02 | 1.2E-08 | 76.1 | 4.1E-02 |
| rs6949989 | 7 | 120428967 | C | 0.46 | 0.11 | 0.02 | 4.1E-08 | 0.46 | 0.07 | 0.02 | 2.4E-03 | 0.46 | 0.09 | 0.01 | 6.3E-10 | 46.5 | 1.7E-01 |
| rs7782552 | 7 | 120434133 | G | 0.45 | 0.11 | 0.02 | 4.3E-08 | 0.46 | 0.07 | 0.02 | 2.4E-03 | 0.46 | 0.09 | 0.01 | 6.3E-10 | 46.5 | 1.7E-01 |
| rs1118142 | 7 | 120435721 | C | 0.48 | 0.11 | 0.02 | 1.7E-08 | 0.47 | 0.07 | 0.02 | 4.0E-03 | 0.48 | 0.09 | 0.02 | 9.8E-10 | 59.2 | 1.2E-01 |
| rs6958635 | 7 | 120441384 | G | 0.45 | 0.11 | 0.02 | 4.5E-08 | 0.46 | 0.07 | 0.02 | 2.2E-03 | 0.46 | 0.09 | 0.01 | 5.2E-10 | 43.8 | 1.8E-01 |
| rs10277935 | 7 | 120441526 | A | 0.45 | 0.11 | 0.02 | 4.6E-08 | 0.46 | 0.07 | 0.02 | 1.8E-03 | 0.46 | 0.09 | 0.01 | 4.6E-10 | 34 | 2.2E-01 |
| rs10953923 | 7 | 120449087 | G | 0.45 | 0.11 | 0.02 | 4.9E-08 | 0.46 | 0.07 | 0.02 | 2.0E-03 | 0.46 | 0.09 | 0.01 | 5.5E-10 | 37.5 | 2.1E-01 |
| rs1981711 | 7 | 120451695 | A | 0.45 | 0.11 | 0.02 | 5.5E-08 | 0.48 | 0.08 | 0.02 | 8.5E-04 | 0.46 | 0.09 | 0.01 | 1.7E-10 | 11 | 2.9E-01 |
| rs12706305 | 7 | 120453899 | C | 0.44 | 0.12 | 0.02 | 4.6E-10 | 0.46 | 0.08 | 0.02 | 7.0E-04 | 0.45 | 0.10 | 0.01 | 1.7E-11 | 59.5 | 1.2E-01 |
| rs12706306 | 7 | 120454005 | G | 0.44 | 0.12 | 0.02 | 4.6E-10 | 0.46 | 0.08 | 0.02 | 6.9E-04 | 0.45 | 0.10 | 0.01 | 1.7E-11 | 59.5 | 1.2E-01 |
| rs6944789 | 7 | 120458270 | A | 0.44 | 0.12 | 0.02 | 4.7E-10 | 0.46 | 0.08 | 0.02 | 6.4E-04 | 0.45 | 0.10 | 0.01 | 1.4E-11 | 57.7 | 1.2E-01 |
| rs2058501 | 7 | 120467472 | C | 0.44 | 0.12 | 0.02 | 5.9E-10 | 0.46 | 0.08 | 0.02 | 5.6E-04 | 0.45 | 0.10 | 0.01 | 1.4E-11 | 53.7 | 1.4E-01 |
| rs10257314 | 7 | 120468077 | C | 0.44 | 0.12 | 0.02 | 5.7E-10 | 0.45 | 0.08 | 0.02 | 3.0E-04 | 0.45 | 0.10 | 0.02 | 1.0E-11 | 41.2 | 1.9E-01 |
| rs2721375 | 7 | 120491525 | C | 0.07 | 0.21 | 0.04 | 3.7E-08 | 0.07 | 0.21 | 0.05 | 3.0E-06 | 0.07 | 0.21 | 0.03 | 7.0E-13 | 0 | 9.7E-01 |
| rs2538491 | 7 | 120492412 | A | 0.07 | 0.21 | 0.04 | 3.7E-08 | 0.08 | 0.19 | 0.04 | 7.9E-06 | 0.07 | 0.20 | 0.03 | 1.7E-12 | 0 | 6.9E-01 |
| rs2538492 | 7 | 120492482 | A | 0.07 | 0.21 | 0.04 | 5.6E-08 | 0.09 | 0.18 | 0.04 | 3.1E-06 | 0.08 | 0.19 | 0.03 | 1.9E-12 | 0 | 6.6E-01 |
| rs6466766 | 7 | 120492536 | T | 0.49 | 0.11 | 0.02 | 7.6E-09 | 0.55 | 0.12 | 0.02 | 2.4E-08 | 0.52 | 0.12 | 0.01 | 1.8E-15 | 0 | 7.4E-01 |
| rs2192289 | 7 | 120494128 | G | 0.07 | 0.20 | 0.04 | 6.8E-08 | 0.09 | 0.17 | 0.04 | 1.9E-05 | 0.08 | 0.19 | 0.03 | 1.4E-11 | 0 | 5.4E-01 |
| rs2402554 | 7 | 120496295 | A | 0.07 | 0.20 | 0.04 | 7.6E-08 | 0.08 | 0.18 | 0.04 | 1.4E-05 | 0.08 | 0.19 | 0.03 | 1.1E-11 | 0 | 6.4E-01 |
| rs9641653 | 7 | 120496516 | C | 0.07 | 0.20 | 0.04 | 8.2E-08 | 0.09 | 0.18 | 0.04 | 8.0E-06 | 0.08 | 0.19 | 0.03 | 5.9E-12 | 0 | 6.2E-01 |
| rs10268570 | 7 | 120497025 | T | 0.07 | 0.20 | 0.04 | 8.4E-08 | 0.09 | 0.18 | 0.04 | 8.0E-06 | 0.08 | 0.19 | 0.03 | 5.9E-12 | 0 | 6.2E-01 |
| rs2721349 | 7 | 120497709 | C | 0.07 | 0.20 | 0.04 | 8.6E-08 | 0.08 | 0.19 | 0.04 | 8.4E-06 | 0.07 | 0.20 | 0.03 | 5.7E-12 | 0 | 8.1E-01 |
| rs9690143 | 7 | 120498209 | A | 0.07 | 0.20 | 0.04 | 8.7E-08 | 0.11 | 0.14 | 0.04 | 1.9E-04 | 0.09 | 0.17 | 0.03 | 3.0E-10 | 32.4 | 2.2E-01 |
| rs13246689 | 7 | 120500232 | T | 0.49 | 0.11 | 0.02 | 8.6E-09 | 0.54 | 0.12 | 0.02 | 6.5E-08 | 0.52 | 0.12 | 0.01 | 1.6E-15 | 0 | 7.8E-01 |
| rs6962260 | 7 | 120501748 | A | 0.07 | 0.20 | 0.04 | 9.1E-08 | 0.08 | 0.19 | 0.04 | 9.6E-06 | 0.07 | 0.19 | 0.03 | 8.1E-12 | 0 | 7.9E-01 |
| rs11766764 | 7 | 120502160 | G | 0.49 | 0.11 | 0.02 | 8.1E-09 | 0.55 | 0.12 | 0.02 | 4.3E-08 | 0.52 | 0.12 | 0.01 | 1.3E-15 | 0 | 7.6E-01 |
| rs10953924 | 7 | 120502365 | T | 0.49 | 0.11 | 0.02 | 8.0E-09 | 0.55 | 0.12 | 0.02 | 4.8E-08 | 0.52 | 0.12 | 0.01 | 1.3E-15 | 0 | 7.6E-01 |
| rs10953925 | 7 | 120502390 | A | 0.49 | 0.11 | 0.02 | 8.0E-09 | 0.54 | 0.12 | 0.02 | 1.4E-07 | 0.52 | 0.11 | 0.01 | 3.4E-15 | 0 | 8.6E-01 |
| rs2110280 | 7 | 120505655 | T | 0.49 | 0.11 | 0.02 | 2.2E-08 | 0.54 | 0.12 | 0.02 | 7.1E-08 | 0.51 | 0.11 | 0.01 | 5.4E-15 | 0 | 7.3E-01 |
| rs17143147 | 7 | 120507197 | T | 0.44 | 0.07 | 0.02 | 6.8E-04 | 0.40 | 0.10 | 0.02 | 9.1E-06 | 0.43 | 0.08 | 0.01 | 3.2E-08 | 37.6 | 2.1E-01 |
| rs6976513 | 7 | 120507674 | T | 0.09 | 0.18 | 0.03 | 1.4E-07 | 0.09 | 0.18 | 0.04 | 1.3E-05 | 0.09 | 0.18 | 0.03 | 8.7E-12 | 0 | 9.4E-01 |
| rs6956851 | 7 | 120507893 | C | 0.19 | 0.15 | 0.03 | 9.2E-09 | 0.18 | 0.09 | 0.03 | 2.3E-03 | 0.19 | 0.12 | 0.02 | 2.9E-10 | 54.3 | 1.4E-01 |
| rs7790569 | 7 | 120508055 | G | 0.09 | 0.18 | 0.03 | 1.4E-07 | 0.09 | 0.18 | 0.04 | 1.0E-05 | 0.09 | 0.18 | 0.03 | 7.8E-12 | 0 | 9.5E-01 |
| rs17536644 | 7 | 120508261 | G | 0.43 | 0.07 | 0.02 | 2.1E-04 | 0.42 | 0.12 | 0.02 | 2.6E-07 | 0.43 | 0.09 | 0.02 | 1.1E-09 | 53.5 | 1.4E-01 |
| rs759157 | 7 | 120509544 | G | 0.09 | 0.18 | 0.03 | 9.2E-08 | 0.08 | 0.18 | 0.04 | 1.2E-05 | 0.09 | 0.18 | 0.03 | 4.9E-12 | 0 | 9.1E-01 |
| rs4140913 | 7 | 120510179 | A | 0.19 | 0.14 | 0.03 | 8.9E-09 | 0.21 | 0.08 | 0.03 | 2.6E-03 | 0.20 | 0.12 | 0.02 | 4.3E-10 | 63.1 | 1.0E-01 |
| rs1024743 | 7 | 120512663 | A | 0.10 | 0.18 | 0.03 | 1.6E-08 | 0.10 | 0.19 | 0.04 | 4.6E-07 | 0.10 | 0.19 | 0.02 | 3.2E-14 | 0 | 9.2E-01 |
| rs2110281 | 7 | 120513045 | G | 0.64 | 0.18 | 0.02 | 7.1E-19 | 0.65 | 0.19 | 0.02 | 2.5E-16 | 0.64 | 0.18 | 0.02 | 2.1E-33 | 0 | 7.0E-01 |
| rs2160013 | 7 | 120513232 | C | 0.10 | 0.18 | 0.03 | 3.4E-08 | 0.10 | 0.17 | 0.04 | 1.1E-05 | 0.10 | 0.17 | 0.02 | 2.3E-12 | 0 | 7.8E-01 |
| rs2968349 | 7 | 120514162 | A | 0.53 | 0.14 | 0.02 | 9.3E-13 | 0.54 | 0.18 | 0.02 | 4.6E-15 | 0.54 | 0.15 | 0.01 | 3.4E-26 | 40.8 | 1.9E-01 |
| rs2968345 | 7 | 120516108 | A | 0.64 | 0.18 | 0.02 | 4.0E-19 | 0.64 | 0.19 | 0.02 | 1.1E-16 | 0.64 | 0.18 | 0.02 | 9.4E-34 | 0 | 7.0E-01 |
| rs6466767 | 7 | 120518813 | C | 0.64 | 0.18 | 0.02 | 3.6E-19 | 0.65 | 0.19 | 0.02 | 1.3E-15 | 0.64 | 0.18 | 0.02 | 4.8E-33 | 0 | 8.7E-01 |
| rs1917114 | 7 | 120519356 | G | 0.54 | 0.10 | 0.02 | 1.1E-07 | 0.53 | 0.11 | 0.02 | 5.2E-07 | 0.53 | 0.11 | 0.01 | 2.6E-13 | 0 | 8.1E-01 |
| rs1917113 | 7 | 120522298 | G | 0.64 | 0.18 | 0.02 | 3.1E-19 | 0.66 | 0.18 | 0.02 | 3.1E-15 | 0.65 | 0.18 | 0.02 | 1.4E-32 | 0 | 9.5E-01 |
| rs12673968 | 7 | 120524294 | G | 0.64 | 0.18 | 0.02 | 3.1E-19 | 0.65 | 0.18 | 0.02 | 2.1E-15 | 0.64 | 0.18 | 0.02 | 9.7E-33 | 0 | 9.2E-01 |
| rs1005400 | 7 | 120525505 | G | 0.54 | 0.10 | 0.02 | 1.1E-07 | 0.53 | 0.11 | 0.02 | 5.6E-07 | 0.54 | 0.11 | 0.01 | 2.6E-13 | 0 | 8.1E-01 |
| rs798937 | 7 | 120527339 | G | 0.20 | 0.15 | 0.02 | 2.2E-09 | 0.21 | 0.09 | 0.03 | 1.0E-03 | 0.20 | 0.12 | 0.02 | 2.3E-11 | 57.9 | 1.2E-01 |
| rs1917112 | 7 | 120527461 | G | 0.43 | 0.08 | 0.02 | 9.0E-05 | 0.43 | 0.11 | 0.02 | 3.8E-07 | 0.43 | 0.09 | 0.01 | 2.6E-10 | 34 | 2.2E-01 |
| rs798938 | 7 | 120528459 | T | 0.10 | 0.18 | 0.03 | 2.7E-08 | 0.10 | 0.14 | 0.04 | 1.0E-04 | 0.10 | 0.16 | 0.02 | 2.2E-11 | 0 | 4.6E-01 |
| rs6466769 | 7 | 120529339 | A | 0.64 | 0.18 | 0.02 | 3.0E-19 | 0.66 | 0.18 | 0.02 | 3.9E-15 | 0.65 | 0.18 | 0.02 | 1.9E-32 | 0 | 9.7E-01 |
| rs6954757 | 7 | 120530418 | G | 0.64 | 0.18 | 0.02 | 3.0E-19 | 0.66 | 0.18 | 0.02 | 4.2E-15 | 0.65 | 0.18 | 0.02 | 1.9E-32 | 0 | 9.7E-01 |
| rs17143161 | 7 | 120533445 | G | 0.43 | 0.08 | 0.02 | 8.8E-05 | 0.44 | 0.12 | 0.02 | 5.6E-08 | 0.44 | 0.10 | 0.01 | 5.4E-11 | 55.8 | 1.3E-01 |
| rs798939 | 7 | 120534464 | A | 0.10 | 0.18 | 0.03 | 3.8E-08 | 0.11 | 0.16 | 0.04 | 1.3E-05 | 0.10 | 0.17 | 0.02 | 2.4E-12 | 0 | 6.4E-01 |
| rs13223036 | 7 | 120534544 | T | 0.63 | 0.19 | 0.02 | 3.7E-20 | 0.65 | 0.19 | 0.02 | 3.1E-16 | 0.64 | 0.19 | 0.02 | 1.2E-34 | 0 | 9.0E-01 |
| rs13226812 | 7 | 120535493 | T | 0.96 | 0.31 | 0.06 | 3.5E-07 | 0.96 | 0.38 | 0.07 | 6.2E-08 | 0.96 | 0.34 | 0.05 | 1.7E-13 | 0 | 4.8E-01 |
| rs798940 | 7 | 120536011 | C | 0.10 | 0.18 | 0.03 | 2.1E-08 | 0.10 | 0.16 | 0.04 | 1.1E-05 | 0.10 | 0.17 | 0.02 | 1.5E-12 | 0 | 7.3E-01 |
| rs10251139 | 7 | 120542238 | C | 0.44 | 0.07 | 0.02 | 2.2E-04 | 0.44 | 0.12 | 0.02 | 2.0E-07 | 0.44 | 0.09 | 0.01 | 4.0E-10 | 53.7 | 1.4E-01 |
| **rs798943** | **7** | **120546135** | **G** | **0.61** | **0.19** | **0.02** | **8.8E-21** | **0.62** | **0.21** | **0.02** | **1.3E-19** | **0.61** | **0.19** | **0.02** | **1.5E-37** | **0** | **5.6E-01** |
| rs798947 | 7 | 120550448 | G | 0.10 | 0.18 | 0.03 | 2.1E-08 | 0.10 | 0.16 | 0.04 | 1.0E-05 | 0.10 | 0.17 | 0.02 | 1.5E-12 | 0 | 7.3E-01 |
| rs1524498 | 7 | 120551713 | C | 0.44 | 0.07 | 0.02 | 2.2E-04 | 0.43 | 0.12 | 0.02 | 1.5E-07 | 0.44 | 0.09 | 0.01 | 3.3E-10 | 55.8 | 1.3E-01 |
| rs1558541 | 7 | 120552011 | C | 0.44 | 0.07 | 0.02 | 2.3E-04 | 0.43 | 0.12 | 0.02 | 1.5E-07 | 0.44 | 0.09 | 0.01 | 3.3E-10 | 55.8 | 1.3E-01 |
| rs798949 | 7 | 120553190 | C | 0.44 | 0.07 | 0.02 | 2.3E-04 | 0.44 | 0.12 | 0.02 | 2.0E-07 | 0.44 | 0.09 | 0.01 | 4.0E-10 | 53.7 | 1.4E-01 |
| rs13221538 | 7 | 120555482 | T | 0.17 | 0.19 | 0.03 | 5.0E-13 | 0.17 | 0.14 | 0.03 | 2.8E-06 | 0.17 | 0.16 | 0.02 | 5.4E-17 | 43.2 | 1.8E-01 |
| rs12706314 | 7 | 120559722 | G | 0.44 | 0.07 | 0.02 | 2.3E-04 | 0.45 | 0.11 | 0.02 | 4.2E-07 | 0.45 | 0.09 | 0.01 | 7.1E-10 | 46.5 | 1.7E-01 |
| rs17283542 | 7 | 120560200 | G | 0.10 | 0.18 | 0.03 | 2.3E-08 | 0.10 | 0.16 | 0.04 | 1.0E-05 | 0.10 | 0.17 | 0.02 | 1.8E-12 | 0 | 7.5E-01 |
| rs7801723 | 7 | 120561396 | C | 0.61 | 0.19 | 0.02 | 8.6E-21 | 0.63 | 0.20 | 0.02 | 4.2E-19 | 0.62 | 0.19 | 0.02 | 4.5E-37 | 0 | 6.2E-01 |
| rs12706318 | 7 | 120562177 | A | 0.61 | 0.19 | 0.02 | 9.0E-21 | 0.62 | 0.20 | 0.02 | 4.9E-19 | 0.62 | 0.19 | 0.02 | 6.4E-37 | 0 | 6.5E-01 |
| rs13232048 | 7 | 120563517 | G | 0.61 | 0.19 | 0.02 | 9.2E-21 | 0.62 | 0.20 | 0.02 | 4.9E-19 | 0.62 | 0.19 | 0.02 | 6.4E-37 | 0 | 6.5E-01 |
| rs13235373 | 7 | 120563993 | A | 0.10 | 0.18 | 0.03 | 2.5E-08 | 0.10 | 0.16 | 0.04 | 1.0E-05 | 0.10 | 0.17 | 0.02 | 1.8E-12 | 0 | 7.5E-01 |
| rs6947453 | 7 | 120564419 | G | 0.61 | 0.18 | 0.02 | 1.1E-17 | 0.62 | 0.18 | 0.02 | 2.7E-15 | 0.62 | 0.18 | 0.02 | 9.1E-31 | 0 | 8.2E-01 |
| rs6952113 | 7 | 120564855 | G | 0.61 | 0.19 | 0.02 | 9.7E-21 | 0.62 | 0.20 | 0.02 | 5.6E-19 | 0.62 | 0.19 | 0.02 | 1.0E-36 | 0 | 6.2E-01 |
| rs10259383 | 7 | 120565247 | T | 0.44 | 0.07 | 0.02 | 2.2E-04 | 0.44 | 0.11 | 0.02 | 2.2E-07 | 0.44 | 0.09 | 0.01 | 4.8E-10 | 51.5 | 1.5E-01 |
| rs10248011 | 7 | 120566050 | A | 0.44 | 0.07 | 0.02 | 2.2E-04 | 0.43 | 0.12 | 0.02 | 1.6E-07 | 0.44 | 0.09 | 0.01 | 3.3E-10 | 55.8 | 1.3E-01 |
| rs10248019 | 7 | 120566067 | A | 0.17 | 0.19 | 0.03 | 6.6E-13 | 0.17 | 0.14 | 0.03 | 2.8E-06 | 0.17 | 0.16 | 0.02 | 6.9E-17 | 40.9 | 1.9E-01 |
| rs872007 | 7 | 120567185 | C | 0.61 | 0.19 | 0.02 | 1.1E-20 | 0.62 | 0.20 | 0.02 | 5.6E-19 | 0.62 | 0.19 | 0.02 | 1.0E-36 | 0 | 6.2E-01 |
| rs2177578 | 7 | 120568586 | A | 0.44 | 0.07 | 0.02 | 2.2E-04 | 0.43 | 0.12 | 0.02 | 1.7E-07 | 0.44 | 0.09 | 0.01 | 3.3E-10 | 55.8 | 1.3E-01 |
| rs10275439 | 7 | 120570661 | G | 0.62 | 0.19 | 0.02 | 1.5E-20 | 0.61 | 0.20 | 0.02 | 6.6E-19 | 0.62 | 0.19 | 0.02 | 1.5E-36 | 0 | 6.5E-01 |
| rs10261671 | 7 | 120570787 | C | 0.61 | 0.19 | 0.02 | 1.2E-20 | 0.61 | 0.20 | 0.02 | 1.7E-18 | 0.61 | 0.19 | 0.02 | 2.1E-36 | 0 | 6.7E-01 |
| rs13245690 | 7 | 120572300 | A | 0.61 | 0.19 | 0.02 | 1.3E-20 | 0.62 | 0.20 | 0.02 | 8.0E-19 | 0.62 | 0.19 | 0.02 | 1.0E-36 | 0 | 6.2E-01 |
| rs1112208 | 7 | 120575524 | G | 0.44 | 0.07 | 0.02 | 2.3E-04 | 0.43 | 0.12 | 0.02 | 1.7E-07 | 0.44 | 0.09 | 0.01 | 3.3E-10 | 55.8 | 1.3E-01 |
| rs6950680 | 7 | 120577523 | A | 0.61 | 0.19 | 0.02 | 1.2E-20 | 0.63 | 0.20 | 0.02 | 4.7E-19 | 0.62 | 0.19 | 0.02 | 7.2E-37 | 0 | 6.0E-01 |
| rs10246521 | 7 | 120578366 | T | 0.44 | 0.07 | 0.02 | 2.3E-04 | 0.44 | 0.11 | 0.02 | 4.1E-07 | 0.44 | 0.09 | 0.01 | 7.1E-10 | 46.5 | 1.7E-01 |
| rs2402560 | 7 | 120579853 | T | 0.44 | 0.07 | 0.02 | 1.7E-04 | 0.44 | 0.11 | 0.02 | 5.6E-07 | 0.44 | 0.09 | 0.01 | 6.7E-10 | 40.8 | 1.9E-01 |
| rs10235934 | 7 | 120580564 | G | 0.44 | 0.07 | 0.02 | 1.7E-04 | 0.44 | 0.11 | 0.02 | 5.3E-07 | 0.44 | 0.09 | 0.01 | 6.7E-10 | 40.8 | 1.9E-01 |
| rs2402561 | 7 | 120582803 | T | 0.10 | 0.17 | 0.03 | 5.5E-08 | 0.10 | 0.15 | 0.04 | 3.4E-05 | 0.10 | 0.16 | 0.02 | 1.1E-11 | 0 | 6.4E-01 |
| rs1125447 | 7 | 120584370 | A | 0.44 | 0.07 | 0.02 | 1.6E-04 | 0.44 | 0.11 | 0.02 | 5.6E-07 | 0.44 | 0.09 | 0.01 | 6.7E-10 | 40.8 | 1.9E-01 |
| rs10228519 | 7 | 120586320 | C | 0.51 | 0.11 | 0.02 | 8.2E-09 | 0.51 | 0.13 | 0.02 | 2.1E-09 | 0.51 | 0.12 | 0.01 | 1.2E-16 | 0 | 4.7E-01 |
| rs10215148 | 7 | 120586918 | A | 0.44 | 0.07 | 0.02 | 1.6E-04 | 0.44 | 0.11 | 0.02 | 5.7E-07 | 0.44 | 0.09 | 0.01 | 6.7E-10 | 40.8 | 1.9E-01 |
| rs10215475 | 7 | 120587028 | C | 0.44 | 0.07 | 0.02 | 1.6E-04 | 0.44 | 0.11 | 0.02 | 5.7E-07 | 0.44 | 0.09 | 0.01 | 6.7E-10 | 40.8 | 1.9E-01 |
| rs11531545 | 7 | 120587209 | G | 0.44 | 0.07 | 0.02 | 1.6E-04 | 0.43 | 0.11 | 0.02 | 3.5E-07 | 0.44 | 0.09 | 0.01 | 4.3E-10 | 40.8 | 1.9E-01 |
| rs1357755 | 7 | 120588073 | C | 0.44 | 0.07 | 0.02 | 1.6E-04 | 0.44 | 0.11 | 0.02 | 5.8E-07 | 0.44 | 0.09 | 0.01 | 6.3E-10 | 34 | 2.2E-01 |
| rs4144090 | 7 | 120598575 | T | 0.10 | 0.17 | 0.03 | 5.3E-08 | 0.10 | 0.17 | 0.04 | 1.1E-05 | 0.10 | 0.17 | 0.02 | 5.4E-12 | 0 | 8.6E-01 |
| rs11981613 | 7 | 120603299 | C | 0.10 | 0.17 | 0.03 | 5.0E-08 | 0.09 | 0.18 | 0.04 | 4.3E-06 | 0.09 | 0.18 | 0.03 | 1.9E-12 | 0 | 8.6E-01 |
| rs6971407 | 7 | 120603864 | T | 0.49 | 0.10 | 0.02 | 2.2E-07 | 0.49 | 0.11 | 0.02 | 5.6E-07 | 0.49 | 0.10 | 0.01 | 6.1E-13 | 0 | 7.3E-01 |
| rs6972481 | 7 | 120604355 | C | 0.49 | 0.10 | 0.02 | 2.3E-07 | 0.49 | 0.11 | 0.02 | 5.5E-07 | 0.49 | 0.10 | 0.01 | 6.1E-13 | 0 | 7.3E-01 |
| rs13233295 | 7 | 120605725 | T | 0.10 | 0.18 | 0.03 | 4.4E-08 | 0.09 | 0.17 | 0.04 | 8.7E-06 | 0.10 | 0.17 | 0.02 | 2.8E-12 | 0 | 9.0E-01 |
| rs1404267 | 7 | 120607720 | G | 0.10 | 0.17 | 0.03 | 4.9E-08 | 0.09 | 0.18 | 0.04 | 4.2E-06 | 0.10 | 0.18 | 0.02 | 2.2E-12 | 0 | 9.5E-01 |
| rs7798060 | 7 | 120609622 | C | 0.59 | 0.17 | 0.02 | 3.1E-17 | 0.60 | 0.17 | 0.02 | 2.7E-14 | 0.60 | 0.17 | 0.01 | 1.5E-29 | 0 | 9.2E-01 |
| rs12706321 | 7 | 120612402 | A | 0.96 | 0.31 | 0.06 | 3.4E-07 | 0.97 | 0.37 | 0.07 | 8.3E-08 | 0.96 | 0.33 | 0.05 | 2.9E-13 | 0 | 5.1E-01 |
| rs17143194 | 7 | 120613178 | A | 0.10 | 0.17 | 0.03 | 5.0E-08 | 0.09 | 0.18 | 0.04 | 3.9E-06 | 0.10 | 0.18 | 0.02 | 1.9E-12 | 0 | 9.4E-01 |
| rs1554634 | 7 | 120613474 | T | 0.59 | 0.17 | 0.02 | 5.2E-17 | 0.59 | 0.17 | 0.02 | 3.6E-14 | 0.59 | 0.17 | 0.01 | 4.8E-29 | 0 | 8.9E-01 |
| rs10085590 | 7 | 120619259 | A | 0.59 | 0.17 | 0.02 | 5.5E-17 | 0.58 | 0.17 | 0.02 | 4.4E-14 | 0.58 | 0.17 | 0.01 | 6.7E-29 | 0 | 9.2E-01 |
| rs12706322 | 7 | 120626403 | G | 0.10 | 0.17 | 0.03 | 5.2E-08 | 0.09 | 0.18 | 0.04 | 3.4E-06 | 0.10 | 0.18 | 0.02 | 1.7E-12 | 0 | 9.2E-01 |
| rs13238393 | 7 | 120630353 | G | 0.10 | 0.17 | 0.03 | 5.5E-08 | 0.09 | 0.18 | 0.04 | 3.4E-06 | 0.10 | 0.18 | 0.02 | 1.7E-12 | 0 | 9.2E-01 |
| rs7797976 | 7 | 120630752 | C | 0.59 | 0.17 | 0.02 | 6.1E-17 | 0.60 | 0.17 | 0.02 | 2.5E-14 | 0.59 | 0.17 | 0.02 | 2.3E-28 | 0 | 8.2E-01 |
| rs6947494 | 7 | 120630944 | C | 0.59 | 0.17 | 0.02 | 6.2E-17 | 0.58 | 0.17 | 0.02 | 5.2E-14 | 0.58 | 0.17 | 0.01 | 6.7E-29 | 0 | 9.2E-01 |
| rs1917118 | 7 | 120631560 | C | 0.59 | 0.17 | 0.02 | 6.4E-17 | 0.59 | 0.17 | 0.02 | 6.0E-14 | 0.59 | 0.17 | 0.02 | 6.0E-28 | 0 | 9.0E-01 |
| rs13229354 | 7 | 120632983 | A | 0.10 | 0.17 | 0.03 | 5.5E-08 | 0.09 | 0.18 | 0.04 | 3.3E-06 | 0.10 | 0.18 | 0.02 | 1.7E-12 | 0 | 9.2E-01 |
| rs6954210 | 7 | 120635621 | G | 0.59 | 0.17 | 0.02 | 6.8E-17 | 0.59 | 0.17 | 0.02 | 6.9E-14 | 0.59 | 0.17 | 0.02 | 6.0E-28 | 0 | 9.0E-01 |
| rs6970762 | 7 | 120639270 | A | 0.59 | 0.17 | 0.02 | 6.9E-17 | 0.60 | 0.17 | 0.02 | 2.0E-14 | 0.59 | 0.17 | 0.02 | 2.3E-28 | 0 | 8.2E-01 |
| rs1357756 | 7 | 120639429 | C | 0.59 | 0.17 | 0.02 | 7.0E-17 | 0.58 | 0.17 | 0.02 | 6.5E-14 | 0.58 | 0.17 | 0.01 | 6.7E-29 | 0 | 9.2E-01 |
| rs1534015 | 7 | 120640301 | G | 0.59 | 0.17 | 0.02 | 7.2E-17 | 0.58 | 0.17 | 0.02 | 6.7E-14 | 0.58 | 0.17 | 0.01 | 6.7E-29 | 0 | 9.2E-01 |
| rs7786203 | 7 | 120640843 | G | 0.59 | 0.16 | 0.02 | 8.1E-16 | 0.60 | 0.17 | 0.02 | 3.7E-14 | 0.59 | 0.16 | 0.02 | 3.6E-27 | 0 | 7.0E-01 |
| rs12706326 | 7 | 120643015 | C | 0.95 | 0.27 | 0.05 | 1.9E-07 | 0.96 | 0.34 | 0.06 | 1.5E-08 | 0.95 | 0.30 | 0.04 | 3.2E-14 | 0 | 4.1E-01 |
| rs1404268 | 7 | 120644983 | G | 0.59 | 0.16 | 0.02 | 9.6E-15 | 0.57 | 0.17 | 0.02 | 3.9E-13 | 0.59 | 0.16 | 0.02 | 1.5E-26 | 0 | 7.0E-01 |
| rs1524503 | 7 | 120655239 | A | 0.59 | 0.16 | 0.02 | 1.2E-14 | 0.61 | 0.18 | 0.02 | 3.9E-15 | 0.60 | 0.17 | 0.02 | 1.3E-28 | 0 | 3.8E-01 |
| rs1949803 | 7 | 120656861 | T | 0.54 | 0.08 | 0.02 | 2.2E-05 | 0.49 | 0.13 | 0.02 | 3.2E-08 | 0.52 | 0.10 | 0.02 | 1.7E-11 | 46.7 | 1.7E-01 |
| rs17284918 | 7 | 120657396 | A | 0.54 | 0.08 | 0.02 | 6.0E-05 | 0.49 | 0.12 | 0.02 | 1.9E-07 | 0.52 | 0.09 | 0.01 | 5.8E-11 | 43.8 | 1.8E-01 |
| rs6978080 | 7 | 120658191 | C | 0.54 | 0.08 | 0.02 | 6.2E-05 | 0.54 | 0.12 | 0.02 | 2.7E-08 | 0.54 | 0.10 | 0.01 | 1.4E-11 | 59.6 | 1.2E-01 |
| rs17357115 | 7 | 120659357 | A | 0.54 | 0.08 | 0.02 | 6.2E-05 | 0.49 | 0.12 | 0.02 | 1.5E-07 | 0.52 | 0.10 | 0.01 | 4.8E-11 | 46.5 | 1.7E-01 |
| rs12672898 | 7 | 120659805 | G | 0.54 | 0.08 | 0.02 | 6.1E-05 | 0.54 | 0.12 | 0.02 | 2.7E-08 | 0.54 | 0.10 | 0.01 | 1.4E-11 | 59.6 | 1.2E-01 |
| rs10953932 | 7 | 120659896 | C | 0.54 | 0.08 | 0.02 | 6.1E-05 | 0.54 | 0.12 | 0.02 | 2.7E-08 | 0.54 | 0.10 | 0.01 | 1.4E-11 | 59.6 | 1.2E-01 |
| rs1534016 | 7 | 120660314 | T | 0.63 | 0.13 | 0.02 | 3.3E-10 | 0.64 | 0.17 | 0.02 | 1.7E-13 | 0.63 | 0.14 | 0.02 | 1.8E-21 | 49.1 | 1.6E-01 |
| rs6965195 | 7 | 120660674 | G | 0.63 | 0.13 | 0.02 | 3.2E-10 | 0.64 | 0.17 | 0.02 | 1.7E-13 | 0.63 | 0.14 | 0.02 | 1.8E-21 | 49.1 | 1.6E-01 |
| rs1534017 | 7 | 120661778 | T | 0.54 | 0.08 | 0.02 | 6.1E-05 | 0.54 | 0.12 | 0.02 | 2.8E-08 | 0.54 | 0.10 | 0.01 | 1.4E-11 | 59.6 | 1.2E-01 |
| rs1524506 | 7 | 120664237 | A | 0.54 | 0.08 | 0.02 | 6.1E-05 | 0.54 | 0.12 | 0.02 | 6.3E-08 | 0.54 | 0.10 | 0.01 | 2.6E-11 | 53.7 | 1.4E-01 |
| rs11771945 | 7 | 120665597 | C | 0.65 | 0.13 | 0.02 | 1.5E-10 | 0.66 | 0.17 | 0.02 | 2.2E-12 | 0.65 | 0.15 | 0.02 | 7.3E-21 | 14.6 | 2.8E-01 |
| rs11765163 | 7 | 120665938 | A | 0.65 | 0.13 | 0.02 | 1.5E-10 | 0.66 | 0.17 | 0.02 | 2.3E-12 | 0.65 | 0.15 | 0.02 | 7.3E-21 | 14.6 | 2.8E-01 |
| rs1534019 | 7 | 120666554 | C | 0.56 | 0.08 | 0.02 | 5.3E-05 | 0.56 | 0.11 | 0.02 | 6.3E-07 | 0.56 | 0.09 | 0.02 | 6.8E-10 | 18.6 | 2.7E-01 |
| rs10480747 | 7 | 120667809 | A | 0.65 | 0.13 | 0.02 | 1.4E-10 | 0.66 | 0.17 | 0.02 | 2.0E-12 | 0.65 | 0.15 | 0.02 | 5.6E-21 | 19.4 | 2.7E-01 |
| rs7808120 | 7 | 120669958 | G | 0.56 | 0.08 | 0.02 | 5.3E-05 | 0.56 | 0.11 | 0.02 | 6.6E-07 | 0.56 | 0.09 | 0.02 | 6.8E-10 | 18.6 | 2.7E-01 |
| rs1534014 | 7 | 120670517 | A | 0.56 | 0.08 | 0.02 | 5.3E-05 | 0.56 | 0.11 | 0.02 | 6.7E-07 | 0.56 | 0.09 | 0.02 | 6.8E-10 | 18.6 | 2.7E-01 |
| rs10500083 | 7 | 120670838 | T | 0.65 | 0.13 | 0.02 | 1.4E-10 | 0.65 | 0.16 | 0.02 | 9.0E-12 | 0.65 | 0.14 | 0.02 | 2.6E-20 | 0 | 3.6E-01 |
| rs7805374 | 7 | 120671147 | T | 0.56 | 0.08 | 0.02 | 5.2E-05 | 0.56 | 0.11 | 0.02 | 7.0E-07 | 0.56 | 0.09 | 0.02 | 6.8E-10 | 18.6 | 2.7E-01 |
| rs2272196 | 7 | 120671689 | G | 0.65 | 0.13 | 0.02 | 1.4E-10 | 0.65 | 0.16 | 0.02 | 9.5E-12 | 0.65 | 0.14 | 0.02 | 1.8E-20 | 0 | 3.7E-01 |
| rs7792071 | 7 | 120672500 | C | 0.56 | 0.08 | 0.02 | 5.2E-05 | 0.55 | 0.12 | 0.02 | 3.4E-07 | 0.56 | 0.09 | 0.02 | 4.7E-10 | 27.4 | 2.4E-01 |
| rs7795660 | 7 | 120672559 | C | 0.65 | 0.13 | 0.02 | 1.3E-10 | 0.66 | 0.17 | 0.02 | 2.3E-12 | 0.65 | 0.15 | 0.02 | 5.1E-21 | 9.3 | 2.9E-01 |
| rs7795692 | 7 | 120672659 | A | 0.65 | 0.13 | 0.02 | 1.3E-10 | 0.65 | 0.16 | 0.02 | 9.8E-12 | 0.65 | 0.14 | 0.02 | 1.8E-20 | 0 | 3.7E-01 |
| rs7778938 | 7 | 120672741 | T | 0.65 | 0.13 | 0.02 | 1.3E-10 | 0.66 | 0.16 | 0.02 | 2.9E-12 | 0.65 | 0.15 | 0.02 | 6.6E-21 | 3.6 | 3.1E-01 |
| rs6466774 | 7 | 120674151 | T | 0.56 | 0.08 | 0.02 | 5.2E-05 | 0.56 | 0.11 | 0.02 | 7.4E-07 | 0.56 | 0.09 | 0.02 | 6.8E-10 | 18.6 | 2.7E-01 |
| rs7806875 | 7 | 120675379 | A | 0.56 | 0.08 | 0.02 | 5.1E-05 | 0.56 | 0.11 | 0.02 | 8.4E-07 | 0.56 | 0.09 | 0.02 | 8.2E-10 | 13.6 | 2.8E-01 |
| rs6979948 | 7 | 120676192 | C | 0.56 | 0.08 | 0.02 | 5.1E-05 | 0.57 | 0.11 | 0.02 | 6.1E-07 | 0.56 | 0.09 | 0.02 | 5.7E-10 | 23.2 | 2.5E-01 |
| rs6942652 | 7 | 120676508 | G | 0.56 | 0.08 | 0.02 | 4.8E-05 | 0.57 | 0.11 | 0.02 | 3.8E-06 | 0.57 | 0.09 | 0.02 | 2.8E-09 | 0 | 4.0E-01 |
| rs11509199 | 7 | 120681913 | C | 0.56 | 0.08 | 0.02 | 4.8E-05 | 0.57 | 0.11 | 0.02 | 3.4E-06 | 0.57 | 0.09 | 0.02 | 2.4E-09 | 0 | 3.8E-01 |
| rs10953933 | 7 | 120682497 | C | 0.57 | 0.08 | 0.02 | 4.8E-05 | 0.57 | 0.11 | 0.02 | 3.4E-06 | 0.57 | 0.09 | 0.02 | 2.4E-09 | 0 | 3.8E-01 |
| rs11770502 | 7 | 120682538 | A | 0.55 | 0.08 | 0.02 | 5.9E-05 | 0.55 | 0.10 | 0.02 | 4.1E-05 | 0.55 | 0.09 | 0.02 | 1.1E-08 | 0 | 6.0E-01 |
| rs12706333 | 7 | 120684886 | C | 0.57 | 0.08 | 0.02 | 4.8E-05 | 0.57 | 0.11 | 0.02 | 4.0E-06 | 0.57 | 0.09 | 0.02 | 2.8E-09 | 0 | 4.0E-01 |
| rs10266975 | 7 | 120685528 | T | 0.57 | 0.08 | 0.02 | 4.8E-05 | 0.57 | 0.11 | 0.02 | 4.0E-06 | 0.57 | 0.09 | 0.01 | 1.2E-09 | 0 | 3.9E-01 |
| rs10225276 | 7 | 120685796 | A | 0.57 | 0.08 | 0.02 | 4.9E-05 | 0.57 | 0.11 | 0.02 | 4.1E-06 | 0.57 | 0.09 | 0.01 | 1.2E-09 | 0 | 3.9E-01 |
| rs6948725 | 7 | 120685897 | T | 0.57 | 0.08 | 0.02 | 4.9E-05 | 0.56 | 0.11 | 0.02 | 1.9E-06 | 0.56 | 0.09 | 0.01 | 7.4E-10 | 0 | 3.3E-01 |
| rs6967129 | 7 | 120686306 | A | 0.65 | 0.13 | 0.02 | 1.9E-10 | 0.65 | 0.16 | 0.02 | 2.6E-11 | 0.65 | 0.14 | 0.02 | 7.7E-20 | 0 | 3.9E-01 |
| rs12539571 | 7 | 120687240 | T | 0.57 | 0.08 | 0.02 | 4.9E-05 | 0.57 | 0.10 | 0.02 | 4.9E-06 | 0.57 | 0.09 | 0.01 | 1.5E-09 | 0 | 4.0E-01 |
| rs12706334 | 7 | 120687250 | A | 0.65 | 0.13 | 0.02 | 1.9E-10 | 0.65 | 0.16 | 0.02 | 2.8E-11 | 0.65 | 0.14 | 0.02 | 7.7E-20 | 0 | 3.9E-01 |
| rs4731006 | 7 | 120689912 | G | 0.64 | 0.13 | 0.02 | 2.7E-10 | 0.65 | 0.16 | 0.02 | 2.8E-11 | 0.65 | 0.14 | 0.02 | 1.1E-19 | 0 | 3.7E-01 |
| rs6970383 | 7 | 120689976 | T | 0.56 | 0.08 | 0.02 | 6.3E-05 | 0.56 | 0.10 | 0.02 | 4.9E-06 | 0.56 | 0.09 | 0.01 | 1.9E-09 | 0 | 3.9E-01 |
| rs4609139 | 7 | 120691051 | A | 0.64 | 0.13 | 0.02 | 3.0E-10 | 0.64 | 0.15 | 0.02 | 1.1E-10 | 0.64 | 0.14 | 0.02 | 1.1E-19 | 0 | 4.5E-01 |
| rs6947934 | 7 | 120691371 | T | 0.56 | 0.08 | 0.02 | 6.6E-05 | 0.56 | 0.10 | 0.02 | 5.4E-06 | 0.56 | 0.09 | 0.01 | 2.3E-09 | 0 | 4.0E-01 |
| rs4731007 | 7 | 120691895 | A | 0.56 | 0.08 | 0.02 | 6.7E-05 | 0.56 | 0.10 | 0.02 | 5.6E-06 | 0.56 | 0.09 | 0.01 | 2.3E-09 | 0 | 4.0E-01 |
| rs2536150 | 7 | 120695318 | T | 0.82 | 0.20 | 0.03 | 3.6E-15 | 0.79 | 0.14 | 0.03 | 1.4E-07 | 0.81 | 0.17 | 0.02 | 7.0E-21 | 57.8 | 1.2E-01 |
| rs17508510 | 7 | 120695695 | T | 0.12 | 0.17 | 0.03 | 1.8E-07 | 0.09 | 0.12 | 0.04 | 2.1E-03 | 0.11 | 0.15 | 0.02 | 3.7E-09 | 0 | 3.6E-01 |
| rs2536149 | 7 | 120696058 | G | 0.89 | 0.20 | 0.03 | 1.5E-10 | 0.86 | 0.13 | 0.03 | 1.0E-04 | 0.87 | 0.16 | 0.02 | 7.5E-13 | 58.8 | 1.2E-01 |
| rs2691034 | 7 | 120696192 | A | 0.73 | 0.12 | 0.02 | 1.6E-07 | 0.67 | 0.07 | 0.03 | 8.9E-03 | 0.71 | 0.09 | 0.02 | 1.7E-08 | 58.5 | 1.2E-01 |
| rs2536148 | 7 | 120703223 | C | 0.89 | 0.20 | 0.03 | 4.2E-10 | 0.85 | 0.12 | 0.03 | 1.6E-04 | 0.87 | 0.16 | 0.02 | 1.3E-12 | 64.2 | 9.5E-02 |
| rs2952559 | 7 | 120707405 | G | 0.82 | 0.21 | 0.03 | 1.1E-16 | 0.74 | 0.13 | 0.03 | 2.0E-06 | 0.78 | 0.17 | 0.02 | 7.5E-20 | 81.9 | 1.9E-02 |
| rs798902 | 7 | 120707494 | A | 0.31 | 0.11 | 0.02 | 2.0E-07 | 0.32 | 0.08 | 0.03 | 2.5E-03 | 0.32 | 0.10 | 0.02 | 4.2E-09 | 11.9 | 2.9E-01 |
| rs13247600 | 7 | 120738146 | G | 0.92 | 0.24 | 0.04 | 4.7E-08 | 0.94 | 0.26 | 0.05 | 5.7E-07 | 0.93 | 0.25 | 0.03 | 2.5E-13 | 0 | 7.9E-01 |
| rs3757552 | 7 | 120751201 | A | 0.88 | 0.18 | 0.03 | 1.6E-09 | 0.89 | 0.08 | 0.04 | 1.7E-02 | 0.88 | 0.14 | 0.02 | 1.6E-09 | 76.7 | 3.8E-02 |
| rs3779381 | 7 | 120754026 | G | 0.26 | 0.17 | 0.02 | 6.6E-14 | 0.26 | 0.15 | 0.03 | 2.5E-09 | 0.26 | 0.16 | 0.02 | 1.1E-21 | 0 | 5.4E-01 |
| rs2908004 | 7 | 120757005 | A | 0.44 | 0.20 | 0.02 | 5.5E-24 | 0.50 | 0.15 | 0.02 | 3.5E-11 | 0.47 | 0.18 | 0.01 | 1.4E-32 | 71.4 | 6.1E-02 |
| rs2536189 | 7 | 120760857 | G | 0.44 | 0.20 | 0.02 | 5.4E-24 | 0.50 | 0.15 | 0.02 | 3.4E-11 | 0.47 | 0.18 | 0.01 | 2.0E-32 | 72.4 | 5.7E-02 |
| rs3801387 | 7 | 120762001 | G | 0.27 | 0.19 | 0.02 | 5.5E-18 | 0.27 | 0.17 | 0.03 | 1.7E-11 | 0.27 | 0.18 | 0.02 | 5.0E-27 | 0 | 5.1E-01 |
| rs2707466 | 7 | 120766325 | T | 0.42 | 0.20 | 0.02 | 7.8E-22 | 0.49 | 0.14 | 0.02 | 1.6E-10 | 0.45 | 0.17 | 0.02 | 4.1E-29 | 69 | 7.2E-02 |
| rs2536182 | 7 | 120778073 | G | 0.45 | 0.20 | 0.02 | 8.3E-24 | 0.47 | 0.15 | 0.02 | 5.4E-11 | 0.46 | 0.18 | 0.02 | 6.0E-31 | 59.2 | 1.2E-01 |
| rs2536180 | 7 | 120781909 | C | 0.46 | 0.19 | 0.02 | 3.7E-23 | 0.49 | 0.14 | 0.02 | 2.0E-10 | 0.48 | 0.17 | 0.01 | 1.1E-31 | 67.1 | 8.1E-02 |
| rs3801382 | 7 | 120785513 | G | 0.27 | 0.19 | 0.02 | 5.9E-18 | 0.27 | 0.17 | 0.03 | 3.4E-12 | 0.27 | 0.18 | 0.02 | 1.2E-27 | 0 | 6.5E-01 |
| rs2254595 | 7 | 120794485 | C | 0.46 | 0.19 | 0.02 | 4.0E-23 | 0.50 | 0.14 | 0.02 | 3.3E-10 | 0.48 | 0.17 | 0.01 | 4.3E-31 | 71.7 | 6.0E-02 |
| rs917727 | 7 | 120805815 | T | 0.27 | 0.20 | 0.02 | 5.1E-18 | 0.30 | 0.18 | 0.03 | 1.2E-12 | 0.28 | 0.19 | 0.02 | 9.1E-28 | 0 | 7.3E-01 |
| rs917726 | 7 | 120806093 | T | 0.27 | 0.20 | 0.02 | 5.1E-18 | 0.28 | 0.18 | 0.03 | 2.7E-12 | 0.28 | 0.19 | 0.02 | 2.0E-27 | 0 | 6.3E-01 |
| rs718766 | 7 | 120812738 | C | 0.27 | 0.20 | 0.02 | 5.0E-18 | 0.27 | 0.18 | 0.03 | 2.4E-12 | 0.27 | 0.19 | 0.02 | 8.0E-28 | 0 | 6.5E-01 |
| rs4727924 | 7 | 120819115 | T | 0.46 | 0.20 | 0.02 | 5.3E-23 | 0.47 | 0.17 | 0.02 | 2.8E-13 | 0.46 | 0.19 | 0.02 | 1.5E-34 | 0 | 3.6E-01 |
| rs7776725 | 7 | 120820357 | C | 0.27 | 0.20 | 0.02 | 4.8E-18 | 0.26 | 0.18 | 0.03 | 2.5E-12 | 0.27 | 0.19 | 0.02 | 1.7E-28 | 0 | 6.7E-01 |
| rs749934 | 13 | 42006245 | A | 0.46 | 0.09 | 0.02 | 1.0E-05 | 0.45 | 0.08 | 0.02 | 7.1E-04 | 0.45 | 0.08 | 0.01 | 3.2E-08 | 0 | 6.9E-01 |
| rs17458078 | 13 | 42009354 | C | 0.45 | 0.09 | 0.02 | 7.8E-06 | 0.47 | 0.08 | 0.02 | 5.4E-04 | 0.46 | 0.08 | 0.01 | 2.7E-08 | 0 | 7.1E-01 |
| rs9533143 | 13 | 42009405 | C | 0.45 | 0.09 | 0.02 | 7.6E-06 | 0.47 | 0.08 | 0.02 | 5.3E-04 | 0.46 | 0.08 | 0.01 | 2.7E-08 | 0 | 7.1E-01 |
| rs1021189 | 13 | 42014211 | C | 0.45 | 0.09 | 0.02 | 6.9E-06 | 0.43 | 0.08 | 0.02 | 3.5E-04 | 0.44 | 0.08 | 0.01 | 1.9E-08 | 0 | 7.6E-01 |
| **rs9525638** | **13** | **42026577** | **C** | **0.43** | **0.09** | **0.02** | **1.6E-06** | **0.41** | **0.08** | **0.02** | **1.5E-04** | **0.42** | **0.09** | **0.01** | **2.5E-09** | **0** | **7.1E-01** |
| rs1325798 | 13 | 42037049 | T | 0.43 | 0.09 | 0.02 | 1.6E-06 | 0.40 | 0.08 | 0.02 | 1.6E-04 | 0.42 | 0.09 | 0.01 | 2.5E-09 | 0 | 7.1E-01 |
| rs9533154 | 13 | 42038102 | T | 0.43 | 0.09 | 0.02 | 1.6E-06 | 0.41 | 0.08 | 0.02 | 2.6E-04 | 0.42 | 0.09 | 0.01 | 4.3E-09 | 0 | 6.4E-01 |
| rs17536328 | 13 | 42041029 | T | 0.43 | 0.09 | 0.02 | 2.0E-06 | 0.40 | 0.08 | 0.02 | 1.6E-04 | 0.42 | 0.09 | 0.01 | 3.1E-09 | 0 | 7.4E-01 |
| rs7325635 | 13 | 42043319 | A | 0.43 | 0.09 | 0.02 | 2.3E-06 | 0.41 | 0.08 | 0.02 | 2.8E-04 | 0.42 | 0.09 | 0.01 | 5.4E-09 | 0 | 6.6E-01 |
| rs9533156 | 13 | 42045671 | C | 0.45 | 0.09 | 0.02 | 1.4E-05 | 0.46 | 0.09 | 0.02 | 7.5E-05 | 0.46 | 0.09 | 0.01 | 8.4E-09 | 0 | 9.5E-01 |
| rs9525641 | 13 | 42046024 | C | 0.45 | 0.09 | 0.02 | 1.4E-05 | 0.46 | 0.09 | 0.02 | 5.3E-05 | 0.46 | 0.09 | 0.01 | 7.0E-09 | 0 | 9.2E-01 |
| rs9525643 | 13 | 42057516 | C | 0.43 | 0.09 | 0.02 | 2.6E-06 | 0.40 | 0.08 | 0.02 | 2.4E-04 | 0.42 | 0.09 | 0.01 | 4.6E-09 | 0 | 7.4E-01 |
| rs9525644 | 13 | 42057804 | A | 0.45 | 0.09 | 0.02 | 1.6E-05 | 0.45 | 0.09 | 0.02 | 8.3E-05 | 0.45 | 0.09 | 0.01 | 8.4E-09 | 0 | 9.5E-01 |
| rs1054016 | 13 | 42080002 | T | 0.43 | 0.09 | 0.02 | 1.0E-05 | 0.40 | 0.08 | 0.02 | 3.6E-04 | 0.42 | 0.08 | 0.01 | 1.6E-08 | 0 | 7.9E-01 |

(CHR) = chromosome number; (POS) = position in the genome based on hg18; (EAF) = effect allele frequency; (*β*) = estimates of effect size expressed as adjusted SD per copy of the effect allele (EA); (SE) = standard error of *β*; (*P*) = *P*-value; (I^2^) = Cochran’s Q statistic evaluating heterogeneity and (*P*_HET_) = evidence of heterogeneity. The SNP that showed the strongest evidence of association at each locus is displayed in bold font.
