# Supplementary material for: Phenotypic Dissection of Bone Mineral Density Reveals Skeletal Site Specificity and Facilitates the Identification of Novel Loci in the Genetic Regulation of Bone Mass Attainment
Source: PLoS Genet. 2014 Jun 19;10(6):e1004423. doi: 10.1371/journal.pgen.1004423 (PMC4063697; doi:10.1371/journal.pgen.1004423)
Supplement: Table S7 — Genome-wide associated SK-BMD variants. (CHR) = chromosome number; (POS) = position in the genome based on hg18; (EAF) = effect allele frequency; (β) = estimates of effect size expressed as adjusted SD per copy of the effect allele (EA); (SE) = standard error of β; (P) = P-value; (I2) = Cochran's Q statistic evaluating heterogeneity and (P HET) = evidence of heterogeneity. The SNP that showed the strongest evidence of association at each locus is displayed in bold font. (DOCX) [file pgen.1004423.s023.docx]

**Table S7**. Genome-wide associated SK-BMD variants.

|  |  |  |  | **ALSPAC (n=5299)** | | | | **Generation R (n=4086)** | | | | **META-ANALYSIS (n=9385)** | | | | | |
| --- | --- | --- | --- | --- | --- | --- | --- | --- | --- | --- | --- | --- | --- | --- | --- | --- | --- |
| **RSID** | **CHR** | **POS** | **EA** | **EAF** | ***β*** | **SE** | ***P*** | **EAF** | ***β*** | **SE** | ***P*** | **EAF** | ***β*** | **SE** | ***P*** | **I^2^** | ***P*_HET_** |
| rs2268177 | 1 | 22287997 | A | 0.83 | 0.15 | 0.03 | 2.7E-09 | 0.84 | 0.09 | 0.03 | 1.3E-03 | 0.83 | 0.13 | 0.02 | 9.7E-11 | 55.8 | 1.33E-01 |
| rs7412010 | 1 | 22309033 | G | 0.84 | 0.16 | 0.03 | 4.4E-10 | 0.85 | 0.11 | 0.03 | 4.8E-04 | 0.84 | 0.14 | 0.02 | 2.5E-12 | 52.5 | 1.47E-01 |
| rs3765350 | 1 | 22319903 | A | 0.78 | 0.15 | 0.02 | 2.0E-10 | 0.78 | 0.07 | 0.03 | 4.8E-03 | 0.78 | 0.12 | 0.02 | 3.6E-11 | 77.7 | 3.43E-02 |
| rs2235529 | 1 | 22323074 | C | 0.84 | 0.17 | 0.03 | 2.2E-10 | 0.85 | 0.11 | 0.03 | 4.4E-04 | 0.85 | 0.14 | 0.02 | 3.0E-12 | 54 | 1.41E-01 |
| rs3820282 | 1 | 22340802 | C | 0.84 | 0.17 | 0.03 | 4.5E-10 | 0.85 | 0.11 | 0.03 | 7.3E-04 | 0.85 | 0.14 | 0.02 | 9.7E-12 | 55.4 | 1.34E-01 |
| rs12042083 | 1 | 22345319 | G | 0.79 | 0.13 | 0.02 | 2.1E-08 | 0.77 | 0.08 | 0.03 | 1.6E-03 | 0.78 | 0.11 | 0.02 | 3.3E-10 | 49.1 | 1.61E-01 |
| rs7515106 | 1 | 22345997 | T | 0.79 | 0.13 | 0.02 | 2.0E-08 | 0.74 | 0.07 | 0.03 | 4.3E-03 | 0.77 | 0.10 | 0.02 | 1.5E-09 | 67.5 | 7.95E-02 |
| rs7521902 | 1 | 22363311 | C | 0.77 | 0.13 | 0.02 | 8.2E-09 | 0.78 | 0.11 | 0.03 | 4.7E-05 | 0.77 | 0.12 | 0.02 | 3.3E-12 | 0 | 4.40E-01 |
| **rs3920498** | **1** | **22365474** | **G** | **0.79** | **0.14** | **0.02** | **4.6E-09** | **0.82** | **0.12** | **0.03** | **8.4E-05** | **0.80** | **0.13** | **0.02** | **1.6E-12** | **0** | **5.01E-01** |
| rs7753563 | 6 | 126844291 | A | 0.24 | 0.12 | 0.02 | 1.9E-07 | 0.24 | 0.10 | 0.03 | 1.3E-04 | 0.24 | 0.11 | 0.02 | 4.2E-10 | 0 | 5.87E-01 |
| **rs2130604** | **6** | **126862254** | **T** | **0.24** | **0.12** | **0.02** | **1.9E-07** | **0.23** | **0.11** | **0.03** | **6.5E-05** | **0.24** | **0.11** | **0.02** | **3.3E-11** | **0** | **7.48E-01** |
| rs1262554 | 6 | 127121034 | T | 0.26 | 0.11 | 0.02 | 7.1E-07 | 0.26 | 0.10 | 0.03 | 8.1E-05 | 0.26 | 0.11 | 0.02 | 4.3E-10 | 0 | 7.71E-01 |
| rs271145 | 6 | 133323751 | A | 0.47 | 0.08 | 0.02 | 1.2E-04 | 0.51 | 0.09 | 0.02 | 4.8E-05 | 0.49 | 0.08 | 0.02 | 4.0E-08 | 0 | 6.25E-01 |
| rs271146 | 6 | 133323840 | G | 0.67 | 0.13 | 0.02 | 5.9E-08 | 0.73 | 0.15 | 0.03 | 4.9E-08 | 0.69 | 0.14 | 0.02 | 2.0E-14 | 0 | 4.93E-01 |
| rs872249 | 6 | 133336395 | G | 0.50 | 0.09 | 0.02 | 7.0E-06 | 0.57 | 0.08 | 0.02 | 3.1E-04 | 0.53 | 0.09 | 0.02 | 2.0E-08 | 0 | 8.45E-01 |
| rs271177 | 6 | 133340657 | T | 0.51 | 0.09 | 0.02 | 6.4E-06 | 0.60 | 0.07 | 0.02 | 9.9E-04 | 0.55 | 0.08 | 0.01 | 3.6E-08 | 0 | 5.70E-01 |
| rs271173 | 6 | 133353915 | T | 0.50 | 0.09 | 0.02 | 3.4E-06 | 0.58 | 0.08 | 0.02 | 2.1E-04 | 0.54 | 0.09 | 0.01 | 4.7E-09 | 0 | 7.38E-01 |
| rs271170 | 6 | 133357497 | C | 0.68 | 0.13 | 0.02 | 7.0E-10 | 0.71 | 0.13 | 0.02 | 3.5E-08 | 0.69 | 0.13 | 0.02 | 3.1E-16 | 0 | 8.76E-01 |
| rs3012463 | 6 | 133377186 | T | 0.68 | 0.13 | 0.02 | 4.6E-10 | 0.70 | 0.13 | 0.02 | 2.3E-08 | 0.69 | 0.13 | 0.02 | 2.3E-16 | 0 | 9.01E-01 |
| rs2926349 | 6 | 133381832 | A | 0.68 | 0.13 | 0.02 | 4.6E-10 | 0.70 | 0.13 | 0.02 | 2.3E-08 | 0.69 | 0.13 | 0.02 | 2.9E-16 | 0 | 9.26E-01 |
| **rs3012465** | **6** | **133392629** | **G** | **0.65** | **0.13** | **0.02** | **7.0E-10** | **0.69** | **0.13** | **0.02** | **3.1E-08** | **0.67** | **0.13** | **0.02** | **8.3E-17** | **0** | **8.96E-01** |
| rs3012471 | 6 | 133397928 | T | 0.68 | 0.13 | 0.02 | 4.6E-10 | 0.71 | 0.13 | 0.02 | 3.0E-08 | 0.69 | 0.13 | 0.02 | 2.9E-16 | 0 | 9.26E-01 |
| rs9389047 | 6 | 133478000 | T | 0.35 | 0.09 | 0.02 | 8.8E-06 | 0.42 | 0.08 | 0.02 | 2.4E-04 | 0.38 | 0.09 | 0.02 | 1.0E-08 | 0 | 6.71E-01 |
| rs7764524 | 6 | 133479819 | G | 0.35 | 0.09 | 0.02 | 8.6E-06 | 0.41 | 0.08 | 0.02 | 2.6E-04 | 0.38 | 0.09 | 0.02 | 2.2E-08 | 0 | 7.20E-01 |
| rs7751379 | 6 | 133489548 | G | 0.35 | 0.09 | 0.02 | 8.5E-06 | 0.40 | 0.09 | 0.02 | 1.2E-04 | 0.37 | 0.09 | 0.02 | 5.9E-09 | 0 | 8.45E-01 |
| rs6569867 | 6 | 133491991 | G | 0.36 | 0.10 | 0.02 | 1.6E-06 | 0.42 | 0.08 | 0.02 | 4.7E-04 | 0.39 | 0.09 | 0.02 | 7.9E-09 | 0 | 4.73E-01 |
| rs9389050 | 6 | 133493169 | G | 0.35 | 0.09 | 0.02 | 8.5E-06 | 0.42 | 0.08 | 0.02 | 1.7E-04 | 0.38 | 0.09 | 0.02 | 1.0E-08 | 0 | 7.69E-01 |
| rs546329 | 6 | 133537217 | T | 0.64 | 0.12 | 0.02 | 6.0E-09 | 0.66 | 0.06 | 0.02 | 1.8E-02 | 0.65 | 0.09 | 0.02 | 3.8E-09 | 76.3 | 3.98E-02 |
| rs509685 | 6 | 133558698 | G | 0.74 | 0.13 | 0.02 | 4.0E-09 | 0.77 | 0.08 | 0.03 | 3.2E-03 | 0.75 | 0.11 | 0.02 | 3.9E-10 | 57.9 | 1.24E-01 |
| rs509904 | 6 | 133558775 | T | 0.73 | 0.13 | 0.02 | 1.8E-08 | 0.73 | 0.08 | 0.03 | 3.4E-03 | 0.73 | 0.10 | 0.02 | 6.1E-10 | 53 | 1.45E-01 |
| rs569833 | 6 | 133569628 | C | 0.75 | 0.11 | 0.02 | 1.7E-06 | 0.78 | 0.09 | 0.03 | 9.8E-04 | 0.76 | 0.10 | 0.02 | 1.6E-08 | 0 | 5.29E-01 |
| rs10485232 | 6 | 133678504 | G | 0.65 | 0.09 | 0.02 | 1.7E-05 | 0.64 | 0.10 | 0.02 | 2.8E-05 | 0.65 | 0.09 | 0.02 | 1.5E-09 | 0 | 7.69E-01 |
| rs11759873 | 6 | 133683166 | A | 0.65 | 0.09 | 0.02 | 1.7E-05 | 0.64 | 0.10 | 0.02 | 3.0E-05 | 0.64 | 0.09 | 0.02 | 1.8E-09 | 0 | 7.94E-01 |
| rs1336521 | 6 | 133686710 | G | 0.65 | 0.09 | 0.02 | 1.6E-05 | 0.65 | 0.09 | 0.02 | 2.0E-04 | 0.65 | 0.09 | 0.02 | 1.0E-08 | 0 | 9.48E-01 |
| rs439123 | 6 | 133704714 | C | 0.65 | 0.09 | 0.02 | 1.5E-05 | 0.62 | 0.08 | 0.02 | 6.7E-04 | 0.64 | 0.08 | 0.02 | 4.6E-08 | 0 | 7.20E-01 |
| rs6466766 | 7 | 120492536 | T | 0.49 | 0.12 | 0.02 | 2.5E-09 | 0.55 | 0.10 | 0.02 | 9.8E-06 | 0.52 | 0.11 | 0.01 | 3.0E-13 | 0 | 5.04E-01 |
| rs13246689 | 7 | 120500232 | T | 0.49 | 0.12 | 0.02 | 2.4E-09 | 0.54 | 0.10 | 0.02 | 8.4E-06 | 0.52 | 0.11 | 0.01 | 6.9E-14 | 0 | 5.61E-01 |
| rs11766764 | 7 | 120502160 | G | 0.49 | 0.12 | 0.02 | 2.2E-09 | 0.55 | 0.10 | 0.02 | 1.1E-05 | 0.52 | 0.11 | 0.01 | 8.7E-14 | 0 | 5.39E-01 |
| rs10953924 | 7 | 120502365 | T | 0.49 | 0.12 | 0.02 | 2.2E-09 | 0.55 | 0.10 | 0.02 | 1.2E-05 | 0.52 | 0.11 | 0.01 | 1.1E-13 | 0 | 5.16E-01 |
| rs10953925 | 7 | 120502390 | A | 0.49 | 0.12 | 0.02 | 2.2E-09 | 0.54 | 0.10 | 0.02 | 7.9E-06 | 0.52 | 0.11 | 0.01 | 6.9E-14 | 0 | 5.61E-01 |
| rs2110280 | 7 | 120505655 | T | 0.49 | 0.12 | 0.02 | 3.3E-09 | 0.54 | 0.10 | 0.02 | 1.4E-05 | 0.51 | 0.11 | 0.01 | 1.8E-13 | 0 | 5.16E-01 |
| rs17143147 | 7 | 120507197 | T | 0.44 | 0.10 | 0.02 | 1.5E-07 | 0.40 | 0.07 | 0.02 | 3.6E-03 | 0.43 | 0.09 | 0.01 | 2.6E-09 | 22 | 2.58E-01 |
| rs17536644 | 7 | 120508261 | G | 0.43 | 0.11 | 0.02 | 1.8E-08 | 0.42 | 0.08 | 0.02 | 3.3E-04 | 0.43 | 0.10 | 0.02 | 1.1E-10 | 0 | 3.45E-01 |
| rs2110281 | 7 | 120513045 | G | 0.64 | 0.16 | 0.02 | 2.3E-15 | 0.65 | 0.16 | 0.02 | 8.8E-12 | 0.64 | 0.16 | 0.02 | 2.1E-25 | 0 | 9.74E-01 |
| rs2968349 | 7 | 120514162 | A | 0.53 | 0.13 | 0.02 | 2.7E-11 | 0.54 | 0.12 | 0.02 | 3.4E-08 | 0.54 | 0.13 | 0.01 | 2.9E-18 | 0 | 8.38E-01 |
| rs2968345 | 7 | 120516108 | A | 0.64 | 0.16 | 0.02 | 2.3E-15 | 0.64 | 0.16 | 0.02 | 4.8E-12 | 0.64 | 0.16 | 0.02 | 1.5E-25 | 0 | 1.00E+00 |
| rs6466767 | 7 | 120518813 | C | 0.64 | 0.16 | 0.02 | 2.3E-15 | 0.65 | 0.16 | 0.02 | 6.4E-12 | 0.64 | 0.16 | 0.02 | 1.5E-25 | 0 | 1.00E+00 |
| rs1917114 | 7 | 120519356 | G | 0.54 | 0.13 | 0.02 | 2.6E-11 | 0.53 | 0.12 | 0.02 | 5.9E-08 | 0.53 | 0.12 | 0.01 | 8.3E-18 | 0 | 7.33E-01 |
| rs1917113 | 7 | 120522298 | G | 0.64 | 0.16 | 0.02 | 2.4E-15 | 0.66 | 0.16 | 0.02 | 1.3E-12 | 0.65 | 0.16 | 0.02 | 3.3E-26 | 0 | 8.71E-01 |
| rs12673968 | 7 | 120524294 | G | 0.64 | 0.16 | 0.02 | 2.4E-15 | 0.65 | 0.16 | 0.02 | 8.8E-12 | 0.64 | 0.16 | 0.02 | 2.1E-25 | 0 | 9.74E-01 |
| rs1005400 | 7 | 120525505 | G | 0.54 | 0.13 | 0.02 | 2.4E-11 | 0.53 | 0.12 | 0.02 | 1.6E-07 | 0.54 | 0.12 | 0.01 | 1.7E-17 | 0 | 6.08E-01 |
| rs1917112 | 7 | 120527461 | G | 0.43 | 0.11 | 0.02 | 1.0E-08 | 0.43 | 0.09 | 0.02 | 9.8E-05 | 0.43 | 0.10 | 0.01 | 2.0E-11 | 0 | 3.85E-01 |
| rs6466769 | 7 | 120529339 | A | 0.64 | 0.16 | 0.02 | 1.5E-15 | 0.66 | 0.16 | 0.02 | 1.8E-12 | 0.65 | 0.16 | 0.02 | 3.1E-26 | 0 | 9.22E-01 |
| rs6954757 | 7 | 120530418 | G | 0.64 | 0.16 | 0.02 | 1.4E-15 | 0.66 | 0.16 | 0.02 | 1.6E-12 | 0.65 | 0.16 | 0.02 | 2.1E-26 | 0 | 9.48E-01 |
| rs17143161 | 7 | 120533445 | G | 0.43 | 0.11 | 0.02 | 1.2E-08 | 0.44 | 0.08 | 0.02 | 1.6E-04 | 0.44 | 0.10 | 0.01 | 4.8E-11 | 0 | 3.50E-01 |
| **rs13223036** | **7** | **120534544** | **T** | **0.63** | **0.17** | **0.02** | **3.1E-17** | **0.65** | **0.17** | **0.02** | **6.2E-13** | **0.64** | **0.17** | **0.02** | **1.5E-28** | **0** | **9.22E-01** |
| rs13226812 | 7 | 120535493 | T | 0.96 | 0.25 | 0.06 | 4.2E-05 | 0.96 | 0.28 | 0.07 | 4.4E-05 | 0.96 | 0.27 | 0.05 | 1.0E-08 | 0 | 7.24E-01 |
| rs10251139 | 7 | 120542238 | C | 0.44 | 0.12 | 0.02 | 2.8E-09 | 0.44 | 0.07 | 0.02 | 1.2E-03 | 0.44 | 0.10 | 0.01 | 1.4E-10 | 53.7 | 1.42E-01 |
| rs798943 | 7 | 120546135 | G | 0.61 | 0.18 | 0.02 | 6.2E-19 | 0.62 | 0.15 | 0.02 | 2.1E-11 | 0.61 | 0.17 | 0.02 | 9.4E-28 | 0 | 3.79E-01 |
| rs1524498 | 7 | 120551713 | C | 0.44 | 0.12 | 0.02 | 2.7E-09 | 0.43 | 0.07 | 0.02 | 1.2E-03 | 0.44 | 0.10 | 0.01 | 1.7E-10 | 55.7 | 1.33E-01 |
| rs1558541 | 7 | 120552011 | C | 0.44 | 0.12 | 0.02 | 2.6E-09 | 0.43 | 0.07 | 0.02 | 1.2E-03 | 0.44 | 0.10 | 0.01 | 1.7E-10 | 55.7 | 1.33E-01 |
| rs798949 | 7 | 120553190 | C | 0.44 | 0.12 | 0.02 | 2.5E-09 | 0.44 | 0.07 | 0.02 | 1.7E-03 | 0.44 | 0.10 | 0.01 | 4.3E-11 | 61.2 | 1.08E-01 |
| rs12706314 | 7 | 120559722 | G | 0.44 | 0.12 | 0.02 | 2.6E-09 | 0.45 | 0.07 | 0.02 | 9.2E-04 | 0.45 | 0.10 | 0.01 | 1.9E-11 | 53.7 | 1.42E-01 |
| rs7801723 | 7 | 120561396 | C | 0.61 | 0.18 | 0.02 | 6.0E-19 | 0.63 | 0.16 | 0.02 | 7.0E-12 | 0.62 | 0.17 | 0.02 | 2.6E-28 | 0 | 4.54E-01 |
| rs12706318 | 7 | 120562177 | A | 0.61 | 0.18 | 0.02 | 6.7E-19 | 0.62 | 0.15 | 0.02 | 2.3E-11 | 0.62 | 0.17 | 0.02 | 9.4E-28 | 0 | 3.79E-01 |
| rs13232048 | 7 | 120563517 | G | 0.61 | 0.18 | 0.02 | 7.0E-19 | 0.62 | 0.15 | 0.02 | 2.3E-11 | 0.62 | 0.17 | 0.02 | 9.4E-28 | 0 | 3.79E-01 |
| rs6947453 | 7 | 120564419 | G | 0.61 | 0.18 | 0.02 | 1.7E-17 | 0.62 | 0.14 | 0.02 | 1.6E-09 | 0.62 | 0.16 | 0.02 | 1.1E-24 | 24.1 | 2.51E-01 |
| rs6952113 | 7 | 120564855 | G | 0.61 | 0.18 | 0.02 | 7.9E-19 | 0.62 | 0.15 | 0.02 | 3.1E-11 | 0.62 | 0.17 | 0.02 | 1.9E-27 | 0 | 3.79E-01 |
| rs10259383 | 7 | 120565247 | T | 0.44 | 0.12 | 0.02 | 3.4E-09 | 0.44 | 0.07 | 0.02 | 1.7E-03 | 0.44 | 0.09 | 0.01 | 3.1E-10 | 57.6 | 1.25E-01 |
| rs10248011 | 7 | 120566050 | A | 0.44 | 0.12 | 0.02 | 3.5E-09 | 0.43 | 0.07 | 0.02 | 1.2E-03 | 0.44 | 0.09 | 0.01 | 2.1E-10 | 53.7 | 1.42E-01 |
| rs872007 | 7 | 120567185 | C | 0.61 | 0.18 | 0.02 | 9.5E-19 | 0.62 | 0.15 | 0.02 | 3.1E-11 | 0.62 | 0.17 | 0.02 | 1.9E-27 | 0 | 3.79E-01 |
| rs2177578 | 7 | 120568586 | A | 0.44 | 0.12 | 0.02 | 3.8E-09 | 0.43 | 0.07 | 0.02 | 1.2E-03 | 0.44 | 0.09 | 0.01 | 2.1E-10 | 53.7 | 1.42E-01 |
| rs10275439 | 7 | 120570661 | G | 0.62 | 0.18 | 0.02 | 1.5E-18 | 0.61 | 0.15 | 0.02 | 3.3E-11 | 0.62 | 0.16 | 0.02 | 2.9E-27 | 0 | 3.97E-01 |
| rs10261671 | 7 | 120570787 | C | 0.61 | 0.18 | 0.02 | 1.3E-18 | 0.61 | 0.15 | 0.02 | 4.3E-11 | 0.61 | 0.16 | 0.02 | 3.9E-27 | 0 | 3.79E-01 |
| rs13245690 | 7 | 120572300 | A | 0.61 | 0.18 | 0.02 | 1.1E-18 | 0.62 | 0.16 | 0.02 | 7.3E-12 | 0.62 | 0.17 | 0.02 | 2.9E-28 | 0 | 4.94E-01 |
| rs1112208 | 7 | 120575524 | G | 0.44 | 0.12 | 0.02 | 4.1E-09 | 0.43 | 0.07 | 0.02 | 1.2E-03 | 0.44 | 0.10 | 0.01 | 1.7E-10 | 51.5 | 1.51E-01 |
| rs6950680 | 7 | 120577523 | A | 0.61 | 0.18 | 0.02 | 1.2E-18 | 0.63 | 0.16 | 0.02 | 5.0E-12 | 0.62 | 0.17 | 0.02 | 4.3E-28 | 0 | 5.15E-01 |
| rs10246521 | 7 | 120578366 | T | 0.44 | 0.12 | 0.02 | 4.1E-09 | 0.44 | 0.07 | 0.02 | 9.9E-04 | 0.44 | 0.10 | 0.01 | 1.4E-10 | 49.2 | 1.61E-01 |
| rs2402560 | 7 | 120579853 | T | 0.44 | 0.12 | 0.02 | 2.3E-09 | 0.44 | 0.07 | 0.02 | 8.5E-04 | 0.44 | 0.10 | 0.01 | 7.1E-11 | 51.5 | 1.51E-01 |
| rs10235934 | 7 | 120580564 | G | 0.44 | 0.12 | 0.02 | 2.3E-09 | 0.44 | 0.07 | 0.02 | 8.4E-04 | 0.44 | 0.10 | 0.01 | 7.1E-11 | 51.5 | 1.51E-01 |
| rs1125447 | 7 | 120584370 | A | 0.44 | 0.12 | 0.02 | 2.3E-09 | 0.44 | 0.07 | 0.02 | 8.4E-04 | 0.44 | 0.10 | 0.01 | 7.1E-11 | 51.5 | 1.51E-01 |
| rs10228519 | 7 | 120586320 | C | 0.51 | 0.14 | 0.02 | 1.1E-13 | 0.51 | 0.11 | 0.02 | 6.9E-07 | 0.51 | 0.13 | 0.01 | 6.6E-19 | 30.1 | 2.32E-01 |
| rs10215148 | 7 | 120586918 | A | 0.44 | 0.12 | 0.02 | 2.2E-09 | 0.44 | 0.07 | 0.02 | 8.4E-04 | 0.44 | 0.10 | 0.01 | 7.1E-11 | 51.5 | 1.51E-01 |
| rs10215475 | 7 | 120587028 | C | 0.44 | 0.12 | 0.02 | 2.2E-09 | 0.44 | 0.07 | 0.02 | 8.4E-04 | 0.44 | 0.10 | 0.01 | 7.1E-11 | 51.5 | 1.51E-01 |
| rs11531545 | 7 | 120587209 | G | 0.44 | 0.12 | 0.02 | 2.2E-09 | 0.43 | 0.07 | 0.02 | 9.3E-04 | 0.44 | 0.10 | 0.01 | 8.7E-11 | 53.7 | 1.42E-01 |
| rs1357755 | 7 | 120588073 | C | 0.44 | 0.12 | 0.02 | 2.2E-09 | 0.44 | 0.07 | 0.02 | 7.8E-04 | 0.44 | 0.10 | 0.01 | 7.1E-11 | 51.5 | 1.51E-01 |
| rs10216123 | 7 | 120603625 | G | 0.41 | 0.12 | 0.02 | 3.6E-09 | 0.42 | 0.06 | 0.02 | 5.1E-03 | 0.42 | 0.09 | 0.01 | 8.1E-10 | 68.1 | 7.67E-02 |
| rs6971407 | 7 | 120603864 | T | 0.49 | 0.14 | 0.02 | 2.9E-13 | 0.49 | 0.10 | 0.02 | 1.0E-05 | 0.49 | 0.12 | 0.01 | 3.3E-17 | 53.7 | 1.42E-01 |
| rs6972481 | 7 | 120604355 | C | 0.49 | 0.14 | 0.02 | 2.9E-13 | 0.49 | 0.10 | 0.02 | 1.2E-05 | 0.49 | 0.12 | 0.01 | 4.2E-17 | 55.8 | 1.33E-01 |
| rs7798060 | 7 | 120609622 | C | 0.59 | 0.17 | 0.02 | 8.4E-17 | 0.60 | 0.13 | 0.02 | 2.8E-09 | 0.60 | 0.15 | 0.01 | 7.2E-24 | 12.4 | 2.85E-01 |
| rs12706321 | 7 | 120612402 | A | 0.96 | 0.25 | 0.06 | 3.5E-05 | 0.97 | 0.28 | 0.07 | 4.2E-05 | 0.96 | 0.26 | 0.05 | 9.7E-09 | 0 | 7.46E-01 |
| rs1554634 | 7 | 120613474 | T | 0.59 | 0.16 | 0.02 | 1.1E-16 | 0.59 | 0.13 | 0.02 | 2.1E-08 | 0.59 | 0.15 | 0.01 | 1.2E-22 | 41.1 | 1.93E-01 |
| rs10085590 | 7 | 120619259 | A | 0.59 | 0.16 | 0.02 | 1.2E-16 | 0.58 | 0.12 | 0.02 | 5.4E-08 | 0.58 | 0.14 | 0.01 | 4.1E-22 | 51.5 | 1.51E-01 |
| rs7797976 | 7 | 120630752 | C | 0.59 | 0.16 | 0.02 | 1.4E-16 | 0.60 | 0.14 | 0.02 | 1.9E-09 | 0.59 | 0.15 | 0.02 | 2.7E-23 | 0 | 3.45E-01 |
| rs6947494 | 7 | 120630944 | C | 0.59 | 0.16 | 0.02 | 1.4E-16 | 0.58 | 0.12 | 0.02 | 3.6E-08 | 0.58 | 0.15 | 0.01 | 2.3E-22 | 46.7 | 1.71E-01 |
| rs1917118 | 7 | 120631560 | C | 0.59 | 0.16 | 0.02 | 1.4E-16 | 0.59 | 0.13 | 0.02 | 1.9E-08 | 0.59 | 0.15 | 0.02 | 2.7E-22 | 31.2 | 2.28E-01 |
| rs10274486 | 7 | 120632479 | C | 0.42 | 0.11 | 0.02 | 3.8E-08 | 0.41 | 0.06 | 0.02 | 8.2E-03 | 0.41 | 0.09 | 0.01 | 1.0E-08 | 62.7 | 1.02E-01 |
| rs6954210 | 7 | 120635621 | G | 0.59 | 0.16 | 0.02 | 1.4E-16 | 0.59 | 0.13 | 0.02 | 1.7E-08 | 0.59 | 0.15 | 0.02 | 2.7E-22 | 31.2 | 2.28E-01 |
| rs6970762 | 7 | 120639270 | A | 0.59 | 0.16 | 0.02 | 1.4E-16 | 0.60 | 0.14 | 0.02 | 2.1E-09 | 0.59 | 0.15 | 0.02 | 2.7E-23 | 0 | 3.45E-01 |
| rs1357756 | 7 | 120639429 | C | 0.59 | 0.16 | 0.02 | 1.4E-16 | 0.58 | 0.12 | 0.02 | 2.9E-08 | 0.58 | 0.15 | 0.01 | 1.7E-22 | 44 | 1.82E-01 |
| rs1534015 | 7 | 120640301 | G | 0.59 | 0.16 | 0.02 | 1.4E-16 | 0.58 | 0.12 | 0.02 | 2.8E-08 | 0.58 | 0.15 | 0.01 | 1.7E-22 | 44 | 1.82E-01 |
| rs7786203 | 7 | 120640843 | G | 0.59 | 0.16 | 0.02 | 1.2E-16 | 0.60 | 0.13 | 0.02 | 3.8E-09 | 0.59 | 0.15 | 0.02 | 4.9E-23 | 2 | 3.12E-01 |
| rs10251901 | 7 | 120642564 | G | 0.41 | 0.11 | 0.02 | 4.3E-08 | 0.41 | 0.06 | 0.02 | 7.3E-03 | 0.41 | 0.09 | 0.01 | 1.0E-08 | 59.4 | 1.17E-01 |
| rs12706326 | 7 | 120643015 | C | 0.95 | 0.25 | 0.05 | 3.8E-06 | 0.96 | 0.26 | 0.06 | 1.8E-05 | 0.95 | 0.25 | 0.04 | 3.6E-10 | 0 | 8.62E-01 |
| rs1404268 | 7 | 120644983 | G | 0.59 | 0.17 | 0.02 | 3.8E-17 | 0.57 | 0.14 | 0.02 | 5.3E-09 | 0.59 | 0.16 | 0.02 | 1.5E-24 | 23.1 | 2.54E-01 |
| rs1524503 | 7 | 120655239 | A | 0.59 | 0.17 | 0.02 | 3.6E-17 | 0.61 | 0.15 | 0.02 | 2.5E-10 | 0.60 | 0.16 | 0.02 | 2.7E-26 | 0 | 4.34E-01 |
| rs1949803 | 7 | 120656861 | T | 0.54 | 0.11 | 0.02 | 2.9E-08 | 0.49 | 0.10 | 0.02 | 2.5E-05 | 0.52 | 0.10 | 0.02 | 8.8E-12 | 0 | 6.48E-01 |
| rs17284918 | 7 | 120657396 | A | 0.54 | 0.10 | 0.02 | 7.8E-08 | 0.49 | 0.09 | 0.02 | 3.3E-05 | 0.52 | 0.10 | 0.01 | 7.8E-12 | 0 | 7.07E-01 |
| rs6978080 | 7 | 120658191 | C | 0.54 | 0.10 | 0.02 | 8.0E-08 | 0.54 | 0.11 | 0.02 | 1.2E-06 | 0.54 | 0.11 | 0.01 | 2.5E-13 | 0 | 8.64E-01 |
| rs17357115 | 7 | 120659357 | A | 0.54 | 0.10 | 0.02 | 8.1E-08 | 0.49 | 0.09 | 0.02 | 3.6E-05 | 0.52 | 0.10 | 0.01 | 9.6E-12 | 0 | 6.82E-01 |
| rs12672898 | 7 | 120659805 | G | 0.54 | 0.10 | 0.02 | 8.1E-08 | 0.54 | 0.11 | 0.02 | 1.3E-06 | 0.54 | 0.11 | 0.01 | 3.1E-13 | 0 | 8.91E-01 |
| rs10953932 | 7 | 120659896 | C | 0.54 | 0.10 | 0.02 | 8.1E-08 | 0.54 | 0.11 | 0.02 | 1.3E-06 | 0.54 | 0.11 | 0.01 | 3.1E-13 | 0 | 8.91E-01 |
| rs1534016 | 7 | 120660314 | T | 0.63 | 0.15 | 0.02 | 2.3E-14 | 0.64 | 0.15 | 0.02 | 2.9E-11 | 0.63 | 0.15 | 0.02 | 6.2E-24 | 0 | 9.74E-01 |
| rs6965195 | 7 | 120660674 | G | 0.63 | 0.15 | 0.02 | 2.3E-14 | 0.64 | 0.15 | 0.02 | 3.0E-11 | 0.63 | 0.15 | 0.02 | 6.2E-24 | 0 | 9.74E-01 |
| rs1534017 | 7 | 120661778 | T | 0.54 | 0.10 | 0.02 | 8.2E-08 | 0.54 | 0.11 | 0.02 | 1.5E-06 | 0.54 | 0.11 | 0.01 | 3.1E-13 | 0 | 8.91E-01 |
| rs1524506 | 7 | 120664237 | A | 0.54 | 0.10 | 0.02 | 8.3E-08 | 0.54 | 0.11 | 0.02 | 7.7E-07 | 0.54 | 0.11 | 0.01 | 1.6E-13 | 0 | 8.11E-01 |
| rs11771945 | 7 | 120665597 | C | 0.65 | 0.16 | 0.02 | 3.9E-14 | 0.66 | 0.16 | 0.02 | 6.1E-12 | 0.65 | 0.16 | 0.02 | 3.5E-23 | 0 | 8.27E-01 |
| rs11765163 | 7 | 120665938 | A | 0.65 | 0.16 | 0.02 | 3.9E-14 | 0.66 | 0.16 | 0.02 | 6.2E-12 | 0.65 | 0.16 | 0.02 | 3.5E-23 | 0 | 8.27E-01 |
| rs1534019 | 7 | 120666554 | C | 0.56 | 0.10 | 0.02 | 2.1E-07 | 0.56 | 0.12 | 0.02 | 3.8E-07 | 0.56 | 0.11 | 0.02 | 1.2E-12 | 0 | 6.48E-01 |
| rs10480747 | 7 | 120667809 | A | 0.65 | 0.16 | 0.02 | 4.1E-14 | 0.66 | 0.16 | 0.02 | 4.4E-12 | 0.65 | 0.16 | 0.02 | 2.6E-23 | 0 | 8.03E-01 |
| rs7808120 | 7 | 120669958 | G | 0.56 | 0.10 | 0.02 | 2.2E-07 | 0.56 | 0.12 | 0.02 | 4.0E-07 | 0.56 | 0.11 | 0.02 | 2.0E-12 | 0 | 6.48E-01 |
| rs1534014 | 7 | 120670517 | A | 0.56 | 0.10 | 0.02 | 2.2E-07 | 0.56 | 0.12 | 0.02 | 4.1E-07 | 0.56 | 0.11 | 0.02 | 2.0E-12 | 0 | 6.48E-01 |
| rs10500083 | 7 | 120670838 | T | 0.65 | 0.16 | 0.02 | 4.3E-14 | 0.65 | 0.16 | 0.02 | 3.3E-12 | 0.65 | 0.16 | 0.02 | 2.0E-23 | 0 | 7.79E-01 |
| rs7805374 | 7 | 120671147 | T | 0.56 | 0.10 | 0.02 | 2.3E-07 | 0.56 | 0.12 | 0.02 | 4.3E-07 | 0.56 | 0.11 | 0.02 | 2.0E-12 | 0 | 6.48E-01 |
| rs2272196 | 7 | 120671689 | G | 0.65 | 0.16 | 0.02 | 4.4E-14 | 0.65 | 0.16 | 0.02 | 3.4E-12 | 0.65 | 0.16 | 0.02 | 2.0E-23 | 0 | 7.79E-01 |
| rs7792071 | 7 | 120672500 | C | 0.56 | 0.10 | 0.02 | 2.4E-07 | 0.55 | 0.11 | 0.02 | 8.6E-07 | 0.56 | 0.11 | 0.02 | 4.4E-12 | 0 | 7.45E-01 |
| rs7795660 | 7 | 120672559 | C | 0.65 | 0.16 | 0.02 | 4.6E-14 | 0.66 | 0.16 | 0.02 | 7.3E-12 | 0.65 | 0.16 | 0.02 | 4.6E-23 | 0 | 8.52E-01 |
| rs7795692 | 7 | 120672659 | A | 0.65 | 0.16 | 0.02 | 4.6E-14 | 0.65 | 0.16 | 0.02 | 3.5E-12 | 0.65 | 0.16 | 0.02 | 2.0E-23 | 0 | 7.79E-01 |
| rs7778938 | 7 | 120672741 | T | 0.65 | 0.16 | 0.02 | 4.6E-14 | 0.66 | 0.16 | 0.02 | 7.4E-12 | 0.65 | 0.16 | 0.02 | 4.6E-23 | 0 | 8.52E-01 |
| rs6466774 | 7 | 120674151 | T | 0.56 | 0.10 | 0.02 | 2.6E-07 | 0.56 | 0.12 | 0.02 | 4.6E-07 | 0.56 | 0.11 | 0.02 | 2.0E-12 | 0 | 6.48E-01 |
| rs7806875 | 7 | 120675379 | A | 0.56 | 0.10 | 0.02 | 2.8E-07 | 0.56 | 0.12 | 0.02 | 4.8E-07 | 0.56 | 0.11 | 0.02 | 2.0E-12 | 0 | 6.48E-01 |
| rs6979948 | 7 | 120676192 | C | 0.56 | 0.10 | 0.02 | 2.8E-07 | 0.57 | 0.12 | 0.02 | 1.7E-07 | 0.56 | 0.11 | 0.02 | 6.9E-13 | 0 | 5.36E-01 |
| rs6942652 | 7 | 120676508 | G | 0.56 | 0.10 | 0.02 | 4.3E-07 | 0.57 | 0.11 | 0.02 | 2.7E-06 | 0.57 | 0.10 | 0.02 | 1.7E-11 | 0 | 7.94E-01 |
| rs11509199 | 7 | 120681913 | C | 0.56 | 0.10 | 0.02 | 4.4E-07 | 0.57 | 0.11 | 0.02 | 2.4E-06 | 0.57 | 0.10 | 0.02 | 1.4E-11 | 0 | 7.69E-01 |
| rs10953933 | 7 | 120682497 | C | 0.57 | 0.10 | 0.02 | 4.4E-07 | 0.57 | 0.11 | 0.02 | 2.5E-06 | 0.57 | 0.10 | 0.02 | 1.4E-11 | 0 | 7.69E-01 |
| rs11770502 | 7 | 120682538 | A | 0.55 | 0.10 | 0.02 | 5.4E-07 | 0.55 | 0.11 | 0.02 | 3.4E-06 | 0.55 | 0.10 | 0.02 | 8.6E-12 | 0 | 7.69E-01 |
| rs12706333 | 7 | 120684886 | C | 0.57 | 0.10 | 0.02 | 4.6E-07 | 0.57 | 0.11 | 0.02 | 2.8E-06 | 0.57 | 0.10 | 0.02 | 1.7E-11 | 0 | 7.94E-01 |
| rs10266975 | 7 | 120685528 | T | 0.57 | 0.10 | 0.02 | 4.7E-07 | 0.57 | 0.11 | 0.02 | 2.8E-06 | 0.57 | 0.10 | 0.02 | 1.7E-11 | 0 | 7.94E-01 |
| rs10225276 | 7 | 120685796 | A | 0.57 | 0.10 | 0.02 | 4.9E-07 | 0.57 | 0.11 | 0.02 | 2.9E-06 | 0.57 | 0.10 | 0.02 | 2.1E-11 | 0 | 7.69E-01 |
| rs6948725 | 7 | 120685897 | T | 0.57 | 0.10 | 0.02 | 4.9E-07 | 0.56 | 0.10 | 0.02 | 6.6E-06 | 0.56 | 0.10 | 0.02 | 5.6E-11 | 0 | 8.96E-01 |
| rs6967129 | 7 | 120686306 | A | 0.65 | 0.16 | 0.02 | 2.1E-14 | 0.65 | 0.16 | 0.02 | 1.7E-11 | 0.65 | 0.16 | 0.02 | 3.5E-24 | 0 | 9.24E-01 |
| rs12539571 | 7 | 120687240 | T | 0.57 | 0.10 | 0.02 | 5.3E-07 | 0.57 | 0.11 | 0.02 | 3.2E-06 | 0.57 | 0.10 | 0.02 | 2.6E-11 | 0 | 7.94E-01 |
| rs12706334 | 7 | 120687250 | A | 0.65 | 0.16 | 0.02 | 2.3E-14 | 0.65 | 0.16 | 0.02 | 1.8E-11 | 0.65 | 0.16 | 0.02 | 4.6E-24 | 0 | 9.49E-01 |
| rs4731006 | 7 | 120689912 | G | 0.64 | 0.15 | 0.02 | 4.2E-14 | 0.65 | 0.16 | 0.02 | 1.8E-11 | 0.65 | 0.16 | 0.02 | 7.5E-24 | 0 | 8.74E-01 |
| rs6970383 | 7 | 120689976 | T | 0.56 | 0.10 | 0.02 | 8.0E-07 | 0.56 | 0.11 | 0.02 | 3.3E-06 | 0.56 | 0.10 | 0.02 | 4.3E-11 | 0 | 7.45E-01 |
| rs4609139 | 7 | 120691051 | A | 0.64 | 0.15 | 0.02 | 4.9E-14 | 0.64 | 0.16 | 0.02 | 1.1E-11 | 0.64 | 0.16 | 0.02 | 1.1E-24 | 0 | 8.71E-01 |
| rs6947934 | 7 | 120691371 | T | 0.56 | 0.10 | 0.02 | 8.6E-07 | 0.56 | 0.11 | 0.02 | 3.5E-06 | 0.56 | 0.10 | 0.02 | 5.2E-11 | 0 | 7.69E-01 |
| rs4731007 | 7 | 120691895 | A | 0.56 | 0.10 | 0.02 | 8.7E-07 | 0.56 | 0.11 | 0.02 | 3.6E-06 | 0.56 | 0.10 | 0.02 | 5.2E-11 | 0 | 7.69E-01 |
| rs2536150 | 7 | 120695318 | T | 0.82 | 0.17 | 0.03 | 1.3E-11 | 0.79 | 0.16 | 0.03 | 4.7E-09 | 0.80 | 0.17 | 0.02 | 1.3E-18 | 0 | 7.11E-01 |
| rs798913 | 7 | 120695434 | A | 0.08 | 0.16 | 0.04 | 1.7E-05 | 0.07 | 0.17 | 0.05 | 2.8E-04 | 0.08 | 0.16 | 0.03 | 2.1E-08 | 0 | 8.52E-01 |
| rs2536149 | 7 | 120696058 | G | 0.89 | 0.15 | 0.03 | 9.5E-07 | 0.86 | 0.18 | 0.03 | 4.9E-08 | 0.87 | 0.16 | 0.02 | 7.4E-13 | 0 | 5.69E-01 |
| rs2536148 | 7 | 120703223 | C | 0.89 | 0.15 | 0.03 | 1.0E-06 | 0.85 | 0.17 | 0.03 | 7.8E-08 | 0.87 | 0.16 | 0.02 | 8.4E-13 | 0 | 6.93E-01 |
| rs798903 | 7 | 120706731 | C | 0.51 | 0.12 | 0.02 | 2.7E-09 | 0.50 | 0.11 | 0.02 | 3.0E-06 | 0.50 | 0.11 | 0.02 | 6.7E-14 | 0 | 7.69E-01 |
| rs2952559 | 7 | 120707405 | G | 0.82 | 0.16 | 0.03 | 7.3E-10 | 0.74 | 0.13 | 0.03 | 1.0E-06 | 0.78 | 0.14 | 0.02 | 8.3E-15 | 0 | 4.50E-01 |
| rs2908004 | 7 | 120757005 | A | 0.44 | 0.07 | 0.02 | 3.1E-04 | 0.50 | 0.11 | 0.02 | 1.5E-06 | 0.47 | 0.09 | 0.01 | 3.6E-09 | 26.8 | 2.42E-01 |
| rs2536189 | 7 | 120760857 | G | 0.44 | 0.07 | 0.02 | 3.1E-04 | 0.50 | 0.11 | 0.02 | 1.6E-06 | 0.47 | 0.09 | 0.01 | 3.5E-09 | 17.7 | 2.70E-01 |
| rs3801387 | 7 | 120762001 | G | 0.27 | 0.08 | 0.02 | 1.5E-04 | 0.27 | 0.10 | 0.03 | 4.9E-05 | 0.27 | 0.09 | 0.02 | 4.7E-08 | 0 | 5.91E-01 |
| rs2536182 | 7 | 120778073 | G | 0.45 | 0.07 | 0.02 | 9.1E-04 | 0.47 | 0.11 | 0.02 | 2.4E-06 | 0.46 | 0.08 | 0.02 | 4.3E-08 | 46.6 | 1.71E-01 |
| rs2536180 | 7 | 120781909 | C | 0.46 | 0.06 | 0.02 | 1.1E-03 | 0.49 | 0.10 | 0.02 | 3.6E-06 | 0.48 | 0.08 | 0.01 | 3.0E-08 | 46.5 | 1.72E-01 |
| rs3801382 | 7 | 120785513 | G | 0.27 | 0.08 | 0.02 | 1.6E-04 | 0.27 | 0.11 | 0.03 | 9.7E-06 | 0.27 | 0.10 | 0.02 | 1.2E-08 | 0 | 4.21E-01 |
| rs917727 | 7 | 120805815 | T | 0.27 | 0.09 | 0.02 | 1.8E-04 | 0.30 | 0.13 | 0.03 | 8.6E-07 | 0.28 | 0.10 | 0.02 | 2.4E-09 | 30.7 | 2.30E-01 |
| rs917726 | 7 | 120806093 | T | 0.27 | 0.09 | 0.02 | 1.8E-04 | 0.28 | 0.12 | 0.03 | 3.4E-06 | 0.28 | 0.10 | 0.02 | 8.2E-09 | 0 | 3.31E-01 |
| rs718766 | 7 | 120812738 | C | 0.27 | 0.09 | 0.02 | 1.8E-04 | 0.27 | 0.12 | 0.03 | 9.3E-06 | 0.27 | 0.10 | 0.02 | 1.5E-08 | 0 | 3.91E-01 |
| rs4727924 | 7 | 120819115 | T | 0.46 | 0.07 | 0.02 | 1.1E-03 | 0.47 | 0.12 | 0.02 | 2.0E-07 | 0.46 | 0.09 | 0.02 | 4.8E-09 | 69.6 | 6.99E-02 |
| rs7776725 | 7 | 120820357 | C | 0.27 | 0.09 | 0.02 | 1.9E-04 | 0.26 | 0.12 | 0.03 | 1.0E-05 | 0.27 | 0.10 | 0.02 | 1.1E-08 | 0 | 3.91E-01 |
| rs3133585 | 8 | 119906332 | G | 0.57 | 0.09 | 0.02 | 1.3E-05 | 0.54 | 0.07 | 0.02 | 8.7E-04 | 0.55 | 0.08 | 0.01 | 3.8E-08 | 0 | 6.82E-01 |
| rs2035977 | 8 | 119951468 | G | 0.60 | 0.09 | 0.02 | 2.0E-05 | 0.63 | 0.09 | 0.02 | 3.8E-05 | 0.62 | 0.09 | 0.01 | 2.7E-09 | 0 | 8.41E-01 |
| rs2055101 | 8 | 119956104 | T | 0.52 | 0.09 | 0.02 | 1.6E-06 | 0.52 | 0.10 | 0.02 | 6.5E-06 | 0.52 | 0.10 | 0.01 | 4.3E-11 | 0 | 8.38E-01 |
| rs10955908 | 8 | 119973738 | A | 0.46 | 0.10 | 0.02 | 5.3E-07 | 0.40 | 0.08 | 0.02 | 1.9E-04 | 0.43 | 0.09 | 0.02 | 1.6E-09 | 0 | 6.48E-01 |
| rs13250753 | 8 | 119975873 | G | 0.46 | 0.10 | 0.02 | 5.3E-07 | 0.40 | 0.08 | 0.02 | 1.9E-04 | 0.43 | 0.09 | 0.02 | 1.6E-09 | 0 | 6.48E-01 |
| rs4407910 | 8 | 119986298 | G | 0.46 | 0.10 | 0.02 | 4.4E-07 | 0.39 | 0.09 | 0.02 | 1.6E-04 | 0.43 | 0.09 | 0.01 | 5.5E-10 | 0 | 6.40E-01 |
| rs13439134 | 8 | 119988049 | C | 0.46 | 0.10 | 0.02 | 4.3E-07 | 0.39 | 0.09 | 0.02 | 1.4E-04 | 0.43 | 0.09 | 0.01 | 4.5E-10 | 0 | 6.64E-01 |
| rs13277230 | 8 | 119992174 | T | 0.46 | 0.10 | 0.02 | 3.2E-07 | 0.40 | 0.08 | 0.02 | 2.0E-04 | 0.43 | 0.09 | 0.01 | 4.2E-10 | 0 | 5.70E-01 |
| rs10101385 | 8 | 119993031 | G | 0.46 | 0.10 | 0.02 | 3.2E-07 | 0.40 | 0.08 | 0.02 | 2.2E-04 | 0.43 | 0.09 | 0.01 | 5.1E-10 | 0 | 5.48E-01 |
| rs4355801 | 8 | 119993054 | G | 0.46 | 0.10 | 0.02 | 3.2E-07 | 0.40 | 0.08 | 0.02 | 2.5E-04 | 0.43 | 0.09 | 0.01 | 6.2E-10 | 0 | 5.26E-01 |
| rs4319131 | 8 | 120016832 | G | 0.45 | 0.10 | 0.02 | 6.6E-07 | 0.39 | 0.09 | 0.02 | 5.3E-05 | 0.43 | 0.10 | 0.02 | 3.7E-10 | 0 | 8.45E-01 |
| rs6469788 | 8 | 120021931 | A | 0.48 | 0.09 | 0.02 | 3.1E-06 | 0.41 | 0.09 | 0.02 | 1.9E-04 | 0.45 | 0.09 | 0.02 | 2.8E-09 | 0 | 7.94E-01 |
| rs6415470 | 8 | 120024292 | G | 0.45 | 0.10 | 0.02 | 6.1E-07 | 0.39 | 0.09 | 0.02 | 4.2E-05 | 0.43 | 0.10 | 0.02 | 3.1E-10 | 0 | 8.71E-01 |
| rs7463176 | 8 | 120026806 | A | 0.45 | 0.10 | 0.02 | 5.6E-07 | 0.39 | 0.09 | 0.02 | 4.2E-05 | 0.43 | 0.10 | 0.02 | 2.4E-10 | 0 | 8.45E-01 |
| rs11573829 | 8 | 120028804 | T | 0.45 | 0.10 | 0.02 | 5.4E-07 | 0.39 | 0.09 | 0.02 | 4.3E-05 | 0.43 | 0.10 | 0.02 | 2.4E-10 | 0 | 8.45E-01 |
| rs3134063 | 8 | 120028838 | C | 0.51 | 0.10 | 0.02 | 1.0E-06 | 0.47 | 0.07 | 0.02 | 1.7E-03 | 0.49 | 0.08 | 0.01 | 1.8E-08 | 0 | 3.85E-01 |
| rs6469789 | 8 | 120029842 | C | 0.45 | 0.10 | 0.02 | 5.0E-07 | 0.39 | 0.09 | 0.02 | 4.2E-05 | 0.43 | 0.10 | 0.02 | 2.4E-10 | 0 | 8.45E-01 |
| rs2073617 | 8 | 120033464 | G | 0.51 | 0.10 | 0.02 | 8.2E-07 | 0.47 | 0.07 | 0.02 | 1.8E-03 | 0.49 | 0.08 | 0.01 | 1.7E-08 | 0 | 3.50E-01 |
| rs4242592 | 8 | 120038156 | T | 0.45 | 0.10 | 0.02 | 5.2E-07 | 0.39 | 0.09 | 0.02 | 4.8E-05 | 0.42 | 0.10 | 0.02 | 2.9E-10 | 0 | 8.20E-01 |
| rs7006553 | 8 | 120039960 | C | 0.45 | 0.10 | 0.02 | 5.1E-07 | 0.39 | 0.09 | 0.02 | 4.8E-05 | 0.42 | 0.10 | 0.02 | 2.9E-10 | 0 | 8.20E-01 |
| rs10505348 | 8 | 120041877 | T | 0.45 | 0.10 | 0.02 | 5.0E-07 | 0.39 | 0.09 | 0.02 | 4.9E-05 | 0.42 | 0.10 | 0.02 | 2.9E-10 | 0 | 8.20E-01 |
| rs7014574 | 8 | 120046258 | C | 0.45 | 0.10 | 0.02 | 4.5E-07 | 0.39 | 0.09 | 0.02 | 6.5E-05 | 0.42 | 0.10 | 0.02 | 4.2E-10 | 0 | 7.69E-01 |
| rs2062375 | 8 | 120046973 | G | 0.45 | 0.10 | 0.02 | 6.6E-07 | 0.41 | 0.09 | 0.02 | 1.3E-04 | 0.43 | 0.09 | 0.02 | 1.1E-09 | 0 | 6.96E-01 |
| rs6992497 | 8 | 120049250 | A | 0.45 | 0.10 | 0.02 | 7.1E-07 | 0.40 | 0.09 | 0.02 | 1.6E-04 | 0.43 | 0.09 | 0.01 | 6.9E-10 | 0 | 6.64E-01 |
| rs2062377 | 8 | 120076601 | T | 0.43 | 0.11 | 0.02 | 2.3E-08 | 0.38 | 0.07 | 0.02 | 1.1E-03 | 0.41 | 0.09 | 0.02 | 4.7E-10 | 31.2 | 2.28E-01 |
| rs4567065 | 8 | 120077455 | C | 0.41 | 0.11 | 0.02 | 2.7E-08 | 0.38 | 0.07 | 0.02 | 1.5E-03 | 0.40 | 0.10 | 0.02 | 2.3E-10 | 41.1 | 1.92E-01 |
| rs6469792 | 8 | 120077552 | T | 0.47 | 0.11 | 0.02 | 2.3E-08 | 0.43 | 0.05 | 0.02 | 2.1E-02 | 0.45 | 0.08 | 0.01 | 3.4E-08 | 73.3 | 5.27E-02 |
| rs7842942 | 8 | 120077768 | T | 0.41 | 0.11 | 0.02 | 2.6E-08 | 0.37 | 0.08 | 0.02 | 1.2E-03 | 0.39 | 0.10 | 0.02 | 1.9E-10 | 38.1 | 2.04E-01 |
| rs2062376 | 8 | 120079190 | C | 0.47 | 0.11 | 0.02 | 2.3E-08 | 0.43 | 0.05 | 0.02 | 2.1E-02 | 0.45 | 0.08 | 0.01 | 3.4E-08 | 73.3 | 5.27E-02 |
| rs7010043 | 8 | 120080512 | G | 0.45 | 0.10 | 0.02 | 2.6E-07 | 0.41 | 0.06 | 0.02 | 4.1E-03 | 0.43 | 0.08 | 0.01 | 1.8E-08 | 34.5 | 2.17E-01 |
| rs11995824 | 8 | 120081881 | C | 0.45 | 0.12 | 0.02 | 1.4E-09 | 0.39 | 0.06 | 0.02 | 1.2E-02 | 0.42 | 0.09 | 0.02 | 1.2E-09 | 76.3 | 4.01E-02 |
| rs13264172 | 8 | 120082042 | T | 0.47 | 0.11 | 0.02 | 2.2E-08 | 0.42 | 0.05 | 0.02 | 2.7E-02 | 0.45 | 0.08 | 0.02 | 4.6E-08 | 72.9 | 5.45E-02 |
| rs13264791 | 8 | 120082271 | T | 0.46 | 0.11 | 0.02 | 1.8E-08 | 0.41 | 0.08 | 0.02 | 4.0E-04 | 0.44 | 0.10 | 0.02 | 9.9E-11 | 2 | 3.12E-01 |
| rs4424296 | 8 | 120082457 | C | 0.49 | 0.11 | 0.02 | 8.3E-09 | 0.44 | 0.06 | 0.02 | 1.4E-02 | 0.47 | 0.09 | 0.02 | 7.2E-09 | 71 | 6.32E-02 |
| rs4335155 | 8 | 120082650 | A | 0.49 | 0.11 | 0.02 | 8.2E-09 | 0.45 | 0.05 | 0.02 | 3.5E-02 | 0.47 | 0.08 | 0.02 | 2.7E-08 | 77.7 | 3.42E-02 |
| rs1905784 | 8 | 120083804 | G | 0.49 | 0.11 | 0.02 | 1.5E-08 | 0.45 | 0.06 | 0.02 | 9.3E-03 | 0.47 | 0.09 | 0.01 | 6.3E-09 | 68.1 | 7.67E-02 |
| rs1905783 | 8 | 120084017 | C | 0.49 | 0.11 | 0.02 | 1.5E-08 | 0.45 | 0.06 | 0.02 | 9.1E-03 | 0.47 | 0.09 | 0.01 | 5.2E-09 | 66.8 | 8.25E-02 |
| rs4354338 | 8 | 120085241 | A | 0.45 | 0.11 | 0.02 | 2.9E-08 | 0.40 | 0.08 | 0.02 | 6.8E-04 | 0.43 | 0.10 | 0.02 | 3.4E-10 | 13.5 | 2.82E-01 |
| rs6469795 | 8 | 120085650 | G | 0.49 | 0.11 | 0.02 | 1.6E-08 | 0.45 | 0.06 | 0.02 | 8.6E-03 | 0.47 | 0.09 | 0.01 | 5.2E-09 | 66.8 | 8.25E-02 |
| rs9650075 | 8 | 120086260 | A | 0.49 | 0.11 | 0.02 | 1.6E-08 | 0.45 | 0.06 | 0.02 | 8.5E-03 | 0.47 | 0.09 | 0.01 | 5.2E-09 | 66.8 | 8.25E-02 |
| rs6469797 | 8 | 120087340 | C | 0.49 | 0.11 | 0.02 | 1.5E-08 | 0.46 | 0.05 | 0.02 | 2.1E-02 | 0.48 | 0.08 | 0.01 | 1.9E-08 | 74.2 | 4.88E-02 |
| rs6469798 | 8 | 120087472 | C | 0.45 | 0.11 | 0.02 | 2.7E-08 | 0.40 | 0.08 | 0.02 | 6.8E-04 | 0.43 | 0.10 | 0.02 | 3.4E-10 | 13.5 | 2.82E-01 |
| rs4424291 | 8 | 120087916 | A | 0.49 | 0.11 | 0.02 | 1.5E-08 | 0.45 | 0.06 | 0.02 | 8.5E-03 | 0.47 | 0.09 | 0.01 | 5.2E-09 | 66.8 | 8.25E-02 |
| rs4495460 | 8 | 120088497 | A | 0.49 | 0.11 | 0.02 | 1.5E-08 | 0.45 | 0.06 | 0.02 | 9.2E-03 | 0.47 | 0.09 | 0.01 | 6.3E-09 | 68.1 | 7.67E-02 |
| rs7822098 | 8 | 120089310 | A | 0.45 | 0.11 | 0.02 | 2.8E-08 | 0.40 | 0.08 | 0.02 | 6.4E-04 | 0.43 | 0.10 | 0.02 | 3.4E-10 | 13.5 | 2.82E-01 |
| rs10955919 | 8 | 120090099 | C | 0.47 | 0.10 | 0.02 | 2.1E-07 | 0.43 | 0.07 | 0.02 | 1.5E-03 | 0.45 | 0.09 | 0.01 | 4.1E-09 | 6.7 | 3.01E-01 |
| rs1905777 | 8 | 120091021 | A | 0.45 | 0.11 | 0.02 | 3.2E-08 | 0.39 | 0.07 | 0.02 | 1.2E-03 | 0.42 | 0.09 | 0.02 | 7.1E-10 | 31.2 | 2.28E-01 |
| rs6469801 | 8 | 120094856 | T | 0.45 | 0.11 | 0.02 | 3.5E-08 | 0.40 | 0.08 | 0.02 | 6.7E-04 | 0.43 | 0.09 | 0.01 | 2.6E-10 | 12.4 | 2.85E-01 |
| rs1586274 | 8 | 120095743 | A | 0.54 | 0.12 | 0.02 | 2.3E-09 | 0.48 | 0.07 | 0.02 | 3.3E-03 | 0.51 | 0.10 | 0.02 | 3.1E-10 | 66.5 | 8.41E-02 |
| rs2326193 | 8 | 120098890 | A | 0.45 | 0.11 | 0.02 | 4.3E-08 | 0.40 | 0.08 | 0.02 | 6.8E-04 | 0.43 | 0.09 | 0.01 | 3.2E-10 | 17.7 | 2.70E-01 |
| rs7016585 | 8 | 120099279 | C | 0.47 | 0.10 | 0.02 | 2.9E-07 | 0.43 | 0.07 | 0.02 | 1.3E-03 | 0.45 | 0.09 | 0.01 | 4.2E-09 | 0 | 3.33E-01 |
| rs7004052 | 8 | 120100210 | T | 0.49 | 0.11 | 0.02 | 2.3E-08 | 0.45 | 0.06 | 0.02 | 7.4E-03 | 0.47 | 0.09 | 0.01 | 6.7E-09 | 62.7 | 1.02E-01 |
| rs6999476 | 8 | 120101440 | G | 0.45 | 0.11 | 0.02 | 4.3E-08 | 0.39 | 0.07 | 0.02 | 1.3E-03 | 0.42 | 0.09 | 0.02 | 8.9E-10 | 27.3 | 2.41E-01 |
| rs12682278 | 8 | 120101509 | A | 0.45 | 0.11 | 0.02 | 4.3E-08 | 0.39 | 0.07 | 0.02 | 1.2E-03 | 0.42 | 0.09 | 0.02 | 8.9E-10 | 27.3 | 2.41E-01 |
| rs7813486 | 8 | 120101706 | T | 0.49 | 0.11 | 0.02 | 2.3E-08 | 0.45 | 0.06 | 0.02 | 7.2E-03 | 0.47 | 0.09 | 0.01 | 6.7E-09 | 62.7 | 1.02E-01 |
| rs13262276 | 8 | 120103099 | T | 0.45 | 0.11 | 0.02 | 4.3E-08 | 0.40 | 0.08 | 0.02 | 5.9E-04 | 0.43 | 0.10 | 0.02 | 4.3E-10 | 8 | 2.97E-01 |
| rs10505351 | 8 | 120103179 | C | 0.49 | 0.11 | 0.02 | 2.3E-08 | 0.45 | 0.06 | 0.02 | 6.7E-03 | 0.47 | 0.09 | 0.01 | 5.6E-09 | 61.1 | 1.09E-01 |
| rs6996754 | 8 | 120104051 | T | 0.45 | 0.11 | 0.02 | 4.3E-08 | 0.40 | 0.08 | 0.02 | 7.1E-04 | 0.43 | 0.09 | 0.02 | 5.2E-10 | 13.5 | 2.82E-01 |
| rs4615609 | 8 | 120104674 | A | 0.49 | 0.11 | 0.02 | 2.3E-08 | 0.45 | 0.06 | 0.02 | 6.5E-03 | 0.47 | 0.09 | 0.01 | 5.6E-09 | 61.1 | 1.09E-01 |
| rs4307369 | 8 | 120106134 | A | 0.45 | 0.11 | 0.02 | 4.3E-08 | 0.40 | 0.08 | 0.02 | 5.1E-04 | 0.43 | 0.10 | 0.02 | 3.6E-10 | 2 | 3.12E-01 |
| rs7013731 | 8 | 120107546 | T | 0.49 | 0.11 | 0.02 | 2.3E-08 | 0.45 | 0.06 | 0.02 | 6.1E-03 | 0.47 | 0.09 | 0.01 | 4.7E-09 | 59.4 | 1.17E-01 |
| rs1485295 | 8 | 120108643 | A | 0.45 | 0.11 | 0.02 | 4.3E-08 | 0.40 | 0.08 | 0.02 | 6.5E-04 | 0.43 | 0.10 | 0.02 | 4.3E-10 | 8 | 2.97E-01 |
| rs10098408 | 8 | 120108902 | T | 0.49 | 0.11 | 0.02 | 2.3E-08 | 0.45 | 0.06 | 0.02 | 6.0E-03 | 0.47 | 0.09 | 0.02 | 6.2E-09 | 57.4 | 1.26E-01 |
| rs10086835 | 8 | 120109362 | C | 0.47 | 0.10 | 0.02 | 2.8E-07 | 0.43 | 0.07 | 0.02 | 1.0E-03 | 0.45 | 0.09 | 0.02 | 4.6E-09 | 0 | 3.79E-01 |
| rs1905779 | 8 | 120110346 | C | 0.49 | 0.11 | 0.02 | 2.3E-08 | 0.45 | 0.06 | 0.02 | 5.6E-03 | 0.47 | 0.09 | 0.02 | 6.2E-09 | 57.4 | 1.26E-01 |
| rs1905780 | 8 | 120110831 | A | 0.47 | 0.10 | 0.02 | 2.8E-07 | 0.43 | 0.08 | 0.02 | 9.7E-04 | 0.45 | 0.09 | 0.02 | 3.9E-09 | 0 | 3.97E-01 |
| rs1485312 | 8 | 120113116 | T | 0.49 | 0.11 | 0.02 | 2.3E-08 | 0.45 | 0.06 | 0.02 | 5.5E-03 | 0.47 | 0.09 | 0.02 | 5.2E-09 | 55.5 | 1.34E-01 |
| rs4401893 | 8 | 120113564 | C | 0.49 | 0.11 | 0.02 | 2.3E-08 | 0.45 | 0.06 | 0.02 | 5.4E-03 | 0.47 | 0.09 | 0.02 | 5.2E-09 | 55.5 | 1.34E-01 |
| rs6469804 | 8 | 120114010 | G | 0.45 | 0.11 | 0.02 | 4.3E-08 | 0.39 | 0.08 | 0.02 | 8.5E-04 | 0.42 | 0.09 | 0.02 | 6.2E-10 | 18.5 | 2.68E-01 |
| rs7013722 | 8 | 120116802 | A | 0.49 | 0.11 | 0.02 | 2.3E-08 | 0.45 | 0.06 | 0.02 | 5.5E-03 | 0.47 | 0.09 | 0.02 | 5.2E-09 | 55.5 | 1.34E-01 |
| rs7018198 | 8 | 120117214 | A | 0.49 | 0.11 | 0.02 | 2.3E-08 | 0.45 | 0.06 | 0.02 | 5.7E-03 | 0.47 | 0.09 | 0.02 | 6.2E-09 | 57.4 | 1.26E-01 |
| rs6469805 | 8 | 120117330 | C | 0.47 | 0.12 | 0.02 | 3.1E-09 | 0.43 | 0.07 | 0.02 | 3.8E-03 | 0.45 | 0.09 | 0.01 | 4.3E-10 | 66.8 | 8.25E-02 |
| rs6993813 | 8 | 120121419 | T | 0.47 | 0.12 | 0.02 | 3.2E-09 | 0.43 | 0.06 | 0.02 | 4.3E-03 | 0.45 | 0.09 | 0.01 | 5.3E-10 | 68.1 | 7.67E-02 |
| rs10955924 | 8 | 120122524 | C | 0.49 | 0.11 | 0.02 | 2.3E-08 | 0.45 | 0.06 | 0.02 | 6.2E-03 | 0.47 | 0.09 | 0.02 | 6.2E-09 | 57.4 | 1.26E-01 |
| rs4305930 | 8 | 120127202 | C | 0.45 | 0.11 | 0.02 | 4.3E-08 | 0.39 | 0.08 | 0.02 | 8.9E-04 | 0.42 | 0.09 | 0.02 | 6.2E-10 | 18.5 | 2.68E-01 |
| **rs2450083** | **8** | **120132723** | **T** | **0.48** | **0.11** | **0.02** | **1.7E-07** | **0.47** | **0.10** | **0.02** | **2.2E-05** | **0.47** | **0.10** | **0.02** | **2.1E-11** | **0** | **8.20E-01** |
| rs10835148 | 11 | 27222613 | A | 0.36 | 0.10 | 0.02 | 2.2E-06 | 0.34 | 0.08 | 0.02 | 1.0E-03 | 0.35 | 0.09 | 0.02 | 1.3E-08 | 0 | 5.46E-01 |
| rs7483297 | 11 | 27229309 | T | 0.36 | 0.10 | 0.02 | 2.1E-06 | 0.34 | 0.08 | 0.02 | 9.7E-04 | 0.35 | 0.09 | 0.02 | 8.3E-09 | 0 | 5.36E-01 |
| rs7948420 | 11 | 27233026 | C | 0.36 | 0.10 | 0.02 | 2.0E-06 | 0.34 | 0.08 | 0.02 | 1.0E-03 | 0.35 | 0.09 | 0.02 | 6.7E-09 | 0 | 5.15E-01 |
| rs1038099 | 11 | 27233428 | A | 0.36 | 0.10 | 0.02 | 1.8E-06 | 0.37 | 0.09 | 0.02 | 5.1E-05 | 0.37 | 0.10 | 0.02 | 3.2E-10 | 0 | 9.22E-01 |
| rs1351175 | 11 | 27236896 | C | 0.36 | 0.10 | 0.02 | 1.7E-06 | 0.37 | 0.10 | 0.02 | 3.4E-05 | 0.37 | 0.10 | 0.02 | 2.2E-10 | 0 | 9.74E-01 |
| rs7950903 | 11 | 27238844 | A | 0.36 | 0.10 | 0.02 | 1.7E-06 | 0.37 | 0.09 | 0.02 | 5.6E-05 | 0.37 | 0.10 | 0.02 | 3.9E-10 | 0 | 8.96E-01 |
| rs11029901 | 11 | 27243684 | A | 0.36 | 0.10 | 0.02 | 1.7E-06 | 0.37 | 0.09 | 0.02 | 5.2E-05 | 0.37 | 0.10 | 0.02 | 3.9E-10 | 0 | 8.96E-01 |
| rs10835149 | 11 | 27252254 | C | 0.30 | 0.12 | 0.02 | 1.4E-07 | 0.22 | 0.10 | 0.03 | 3.7E-04 | 0.27 | 0.11 | 0.02 | 4.6E-10 | 0 | 6.55E-01 |
| rs1478690 | 11 | 27252454 | G | 0.37 | 0.10 | 0.02 | 2.6E-06 | 0.37 | 0.09 | 0.02 | 5.4E-05 | 0.37 | 0.09 | 0.02 | 6.3E-10 | 0 | 9.48E-01 |
| rs7481109 | 11 | 27254638 | A | 0.37 | 0.10 | 0.02 | 2.6E-06 | 0.37 | 0.09 | 0.02 | 5.4E-05 | 0.37 | 0.09 | 0.02 | 6.3E-10 | 0 | 9.48E-01 |
| rs10835150 | 11 | 27257637 | G | 0.37 | 0.10 | 0.02 | 2.6E-06 | 0.37 | 0.09 | 0.02 | 5.3E-05 | 0.37 | 0.09 | 0.02 | 6.3E-10 | 0 | 9.48E-01 |
| rs7119628 | 11 | 27259292 | G | 0.37 | 0.10 | 0.02 | 2.6E-06 | 0.37 | 0.09 | 0.02 | 5.3E-05 | 0.37 | 0.09 | 0.02 | 6.3E-10 | 0 | 9.48E-01 |
| rs7126943 | 11 | 27260513 | C | 0.37 | 0.10 | 0.02 | 2.6E-06 | 0.35 | 0.09 | 0.02 | 5.8E-05 | 0.36 | 0.09 | 0.02 | 5.2E-10 | 0 | 9.74E-01 |
| rs899303 | 11 | 27266257 | A | 0.38 | 0.09 | 0.02 | 9.5E-06 | 0.38 | 0.09 | 0.02 | 4.6E-05 | 0.38 | 0.09 | 0.02 | 1.7E-09 | 0 | 8.96E-01 |
| rs10450561 | 11 | 27266532 | T | 0.38 | 0.09 | 0.02 | 9.5E-06 | 0.38 | 0.09 | 0.02 | 4.6E-05 | 0.38 | 0.09 | 0.02 | 1.7E-09 | 0 | 8.96E-01 |
| rs10767628 | 11 | 27267447 | G | 0.38 | 0.09 | 0.02 | 9.5E-06 | 0.38 | 0.09 | 0.02 | 4.6E-05 | 0.38 | 0.09 | 0.02 | 1.7E-09 | 0 | 8.96E-01 |
| rs10835154 | 11 | 27271932 | G | 0.38 | 0.09 | 0.02 | 9.6E-06 | 0.38 | 0.09 | 0.02 | 4.5E-05 | 0.38 | 0.09 | 0.02 | 1.7E-09 | 0 | 8.96E-01 |
| rs7483162 | 11 | 27272970 | A | 0.38 | 0.09 | 0.02 | 9.6E-06 | 0.38 | 0.10 | 0.02 | 4.2E-05 | 0.38 | 0.09 | 0.02 | 1.4E-09 | 0 | 8.71E-01 |
| rs7480595 | 11 | 27273199 | T | 0.38 | 0.09 | 0.02 | 9.6E-06 | 0.38 | 0.10 | 0.02 | 4.1E-05 | 0.38 | 0.09 | 0.02 | 1.4E-09 | 0 | 8.71E-01 |
| rs10835155 | 11 | 27274388 | C | 0.38 | 0.09 | 0.02 | 9.6E-06 | 0.38 | 0.10 | 0.02 | 4.0E-05 | 0.38 | 0.09 | 0.02 | 1.4E-09 | 0 | 8.71E-01 |
| rs10767632 | 11 | 27275041 | C | 0.38 | 0.09 | 0.02 | 9.7E-06 | 0.38 | 0.10 | 0.02 | 3.3E-05 | 0.38 | 0.09 | 0.02 | 1.2E-09 | 0 | 8.45E-01 |
| rs10082611 | 11 | 27276684 | C | 0.38 | 0.09 | 0.02 | 9.5E-06 | 0.38 | 0.10 | 0.02 | 3.0E-05 | 0.38 | 0.09 | 0.02 | 9.7E-10 | 0 | 8.20E-01 |
| rs10082678 | 11 | 27277024 | A | 0.38 | 0.09 | 0.02 | 9.3E-06 | 0.38 | 0.10 | 0.02 | 3.0E-05 | 0.38 | 0.09 | 0.02 | 9.7E-10 | 0 | 8.20E-01 |
| rs7950335 | 11 | 27279079 | C | 0.38 | 0.09 | 0.02 | 9.1E-06 | 0.38 | 0.10 | 0.02 | 2.4E-05 | 0.38 | 0.09 | 0.02 | 8.1E-10 | 0 | 7.94E-01 |
| rs10501084 | 11 | 27279715 | T | 0.38 | 0.09 | 0.02 | 9.0E-06 | 0.38 | 0.10 | 0.02 | 1.9E-05 | 0.38 | 0.09 | 0.02 | 5.4E-10 | 0 | 7.94E-01 |
| rs7107790 | 11 | 27281306 | G | 0.38 | 0.09 | 0.02 | 8.5E-06 | 0.38 | 0.10 | 0.02 | 2.2E-05 | 0.38 | 0.09 | 0.02 | 6.4E-10 | 0 | 8.20E-01 |
| rs10835156 | 11 | 27281794 | T | 0.38 | 0.09 | 0.02 | 8.4E-06 | 0.38 | 0.10 | 0.02 | 2.1E-05 | 0.38 | 0.09 | 0.02 | 5.4E-10 | 0 | 7.94E-01 |
| rs11029931 | 11 | 27282118 | T | 0.38 | 0.09 | 0.02 | 8.3E-06 | 0.38 | 0.10 | 0.02 | 1.1E-05 | 0.38 | 0.10 | 0.02 | 3.1E-10 | 0 | 7.20E-01 |
| rs11029932 | 11 | 27284830 | C | 0.38 | 0.09 | 0.02 | 8.2E-06 | 0.38 | 0.10 | 0.02 | 2.1E-05 | 0.38 | 0.09 | 0.02 | 5.4E-10 | 0 | 7.94E-01 |
| rs10767633 | 11 | 27286301 | T | 0.38 | 0.09 | 0.02 | 8.2E-06 | 0.38 | 0.10 | 0.02 | 2.0E-05 | 0.38 | 0.09 | 0.02 | 5.4E-10 | 0 | 7.94E-01 |
| rs7480168 | 11 | 27286621 | A | 0.38 | 0.09 | 0.02 | 8.1E-06 | 0.38 | 0.10 | 0.02 | 1.9E-05 | 0.38 | 0.09 | 0.02 | 5.4E-10 | 0 | 7.94E-01 |
| rs10835158 | 11 | 27288346 | A | 0.38 | 0.09 | 0.02 | 8.0E-06 | 0.38 | 0.10 | 0.02 | 1.9E-05 | 0.38 | 0.09 | 0.02 | 5.4E-10 | 0 | 7.94E-01 |
| rs10742174 | 11 | 27288867 | A | 0.38 | 0.09 | 0.02 | 7.9E-06 | 0.38 | 0.10 | 0.02 | 1.9E-05 | 0.38 | 0.09 | 0.02 | 5.4E-10 | 0 | 7.94E-01 |
| rs10767634 | 11 | 27289050 | C | 0.38 | 0.09 | 0.02 | 7.9E-06 | 0.38 | 0.10 | 0.02 | 1.9E-05 | 0.38 | 0.09 | 0.02 | 5.4E-10 | 0 | 7.94E-01 |
| rs10160456 | 11 | 27293280 | C | 0.38 | 0.09 | 0.02 | 7.5E-06 | 0.38 | 0.10 | 0.02 | 8.7E-06 | 0.38 | 0.10 | 0.02 | 2.0E-10 | 0 | 7.20E-01 |
| rs9299997 | 11 | 27293448 | C | 0.38 | 0.09 | 0.02 | 7.4E-06 | 0.38 | 0.10 | 0.02 | 1.8E-05 | 0.38 | 0.10 | 0.02 | 3.5E-10 | 0 | 7.94E-01 |
| rs10160687 | 11 | 27293759 | T | 0.38 | 0.09 | 0.02 | 7.4E-06 | 0.38 | 0.10 | 0.02 | 1.8E-05 | 0.38 | 0.10 | 0.02 | 3.5E-10 | 0 | 7.94E-01 |
| rs7110026 | 11 | 27294102 | A | 0.38 | 0.09 | 0.02 | 7.2E-06 | 0.38 | 0.10 | 0.02 | 1.8E-05 | 0.38 | 0.10 | 0.02 | 3.5E-10 | 0 | 7.94E-01 |
| rs9299998 | 11 | 27295157 | T | 0.38 | 0.10 | 0.02 | 3.8E-06 | 0.35 | 0.09 | 0.02 | 3.8E-04 | 0.37 | 0.09 | 0.02 | 6.0E-09 | 0 | 7.09E-01 |
| rs11029954 | 11 | 27311025 | C | 0.74 | 0.11 | 0.02 | 9.1E-07 | 0.71 | 0.08 | 0.02 | 9.6E-04 | 0.72 | 0.10 | 0.02 | 4.9E-09 | 0 | 3.45E-01 |
| rs3929244 | 11 | 27311607 | C | 0.74 | 0.11 | 0.02 | 1.1E-06 | 0.71 | 0.08 | 0.02 | 9.6E-04 | 0.72 | 0.10 | 0.02 | 6.0E-09 | 0 | 3.60E-01 |
| rs10835166 | 11 | 27312538 | C | 0.74 | 0.11 | 0.02 | 1.1E-06 | 0.71 | 0.08 | 0.02 | 9.2E-04 | 0.72 | 0.10 | 0.02 | 5.1E-09 | 0 | 3.77E-01 |
| rs7936621 | 11 | 27382967 | A | 0.64 | 0.09 | 0.02 | 1.8E-05 | 0.63 | 0.08 | 0.02 | 5.6E-04 | 0.63 | 0.08 | 0.01 | 3.3E-08 | 0 | 7.38E-01 |
| rs4074516 | 11 | 27422167 | T | 0.58 | 0.13 | 0.02 | 1.2E-10 | 0.61 | 0.09 | 0.02 | 3.1E-05 | 0.60 | 0.11 | 0.01 | 6.3E-14 | 26.8 | 2.42E-01 |
| rs4542364 | 11 | 27430557 | G | 0.59 | 0.13 | 0.02 | 2.1E-10 | 0.60 | 0.09 | 0.02 | 5.1E-05 | 0.60 | 0.11 | 0.01 | 1.0E-13 | 34.5 | 2.17E-01 |
| rs11030014 | 11 | 27437403 | C | 0.20 | 0.10 | 0.02 | 4.7E-05 | 0.23 | 0.13 | 0.03 | 9.4E-07 | 0.21 | 0.11 | 0.02 | 5.3E-10 | 0 | 3.79E-01 |
| rs11030016 | 11 | 27444568 | C | 0.26 | 0.10 | 0.02 | 3.7E-06 | 0.36 | 0.11 | 0.02 | 1.1E-06 | 0.31 | 0.11 | 0.02 | 4.0E-11 | 0 | 7.55E-01 |
| rs7938467 | 11 | 27459251 | C | 0.55 | 0.12 | 0.02 | 4.0E-10 | 0.59 | 0.09 | 0.02 | 1.0E-04 | 0.57 | 0.11 | 0.02 | 1.1E-12 | 27.3 | 2.41E-01 |
| **rs10835187** | **11** | **27462253** | **C** | **0.45** | **0.15** | **0.02** | **1.0E-13** | **0.50** | **0.11** | **0.02** | **1.6E-06** | **0.47** | **0.13** | **0.01** | **1.6E-17** | **41.1** | **1.93E-01** |
| rs11030024 | 11 | 27465257 | T | 0.21 | 0.09 | 0.02 | 1.5E-04 | 0.26 | 0.13 | 0.03 | 3.7E-07 | 0.23 | 0.11 | 0.02 | 9.8E-10 | 20.6 | 2.62E-01 |
| rs7104230 | 11 | 27471685 | T | 0.45 | 0.13 | 0.02 | 4.1E-11 | 0.51 | 0.10 | 0.02 | 6.0E-06 | 0.47 | 0.12 | 0.01 | 1.1E-14 | 0.4 | 3.16E-01 |
| rs3763965 | 11 | 27485563 | A | 0.45 | 0.13 | 0.02 | 6.0E-11 | 0.50 | 0.10 | 0.02 | 8.2E-06 | 0.47 | 0.11 | 0.01 | 1.9E-14 | 0.4 | 3.16E-01 |
| rs10835189 | 11 | 27498571 | G | 0.47 | 0.12 | 0.02 | 2.6E-10 | 0.52 | 0.09 | 0.02 | 2.0E-05 | 0.50 | 0.11 | 0.01 | 3.4E-14 | 4.8 | 3.05E-01 |
| rs1304101 | 11 | 27528170 | A | 0.48 | 0.12 | 0.02 | 5.4E-10 | 0.52 | 0.09 | 0.02 | 2.0E-05 | 0.50 | 0.11 | 0.01 | 4.9E-14 | 0 | 3.56E-01 |
| rs10835193 | 11 | 27528913 | C | 0.48 | 0.12 | 0.02 | 5.6E-10 | 0.52 | 0.10 | 0.02 | 1.4E-05 | 0.50 | 0.11 | 0.01 | 3.9E-14 | 0 | 3.74E-01 |
| rs10835196 | 11 | 27557911 | G | 0.48 | 0.12 | 0.02 | 9.5E-10 | 0.52 | 0.09 | 0.02 | 1.8E-05 | 0.50 | 0.11 | 0.01 | 8.8E-14 | 0 | 3.93E-01 |
| rs11030064 | 11 | 27574592 | C | 0.47 | 0.12 | 0.02 | 1.3E-09 | 0.52 | 0.09 | 0.02 | 3.1E-05 | 0.49 | 0.11 | 0.01 | 2.3E-13 | 0 | 3.56E-01 |
| rs7937150 | 11 | 27576936 | C | 0.47 | 0.12 | 0.02 | 1.4E-09 | 0.52 | 0.09 | 0.02 | 3.0E-05 | 0.49 | 0.11 | 0.01 | 2.3E-13 | 0 | 3.56E-01 |
| rs7937485 | 11 | 27577049 | G | 0.47 | 0.12 | 0.02 | 1.4E-09 | 0.52 | 0.09 | 0.02 | 3.0E-05 | 0.49 | 0.11 | 0.01 | 2.3E-13 | 0 | 3.56E-01 |
| rs12798439 | 11 | 27578536 | T | 0.47 | 0.12 | 0.02 | 1.4E-09 | 0.52 | 0.09 | 0.02 | 3.0E-05 | 0.49 | 0.11 | 0.01 | 2.3E-13 | 0 | 3.56E-01 |
| rs7949590 | 11 | 27580187 | C | 0.47 | 0.12 | 0.02 | 9.6E-10 | 0.51 | 0.09 | 0.02 | 3.2E-05 | 0.49 | 0.11 | 0.01 | 1.7E-13 | 0 | 3.39E-01 |
| rs11030073 | 11 | 27582518 | A | 0.47 | 0.12 | 0.02 | 9.6E-10 | 0.51 | 0.09 | 0.02 | 3.2E-05 | 0.49 | 0.11 | 0.01 | 1.7E-13 | 0 | 3.39E-01 |
| rs12223664 | 11 | 27584426 | C | 0.46 | 0.12 | 0.02 | 2.9E-09 | 0.50 | 0.09 | 0.02 | 2.4E-05 | 0.48 | 0.11 | 0.01 | 9.4E-13 | 0 | 4.04E-01 |
| rs10767651 | 11 | 27585183 | G | 0.51 | 0.11 | 0.02 | 1.3E-08 | 0.54 | 0.08 | 0.02 | 2.1E-04 | 0.52 | 0.10 | 0.01 | 3.6E-11 | 6.7 | 3.01E-01 |
| rs10767652 | 11 | 27585402 | G | 0.48 | 0.12 | 0.02 | 1.5E-09 | 0.52 | 0.09 | 0.02 | 2.0E-05 | 0.50 | 0.11 | 0.01 | 2.0E-13 | 0 | 4.12E-01 |
| rs11030094 | 11 | 27616351 | G | 0.56 | 0.11 | 0.02 | 5.6E-08 | 0.58 | 0.07 | 0.02 | 1.3E-03 | 0.57 | 0.09 | 0.01 | 4.0E-10 | 21.4 | 2.60E-01 |
| rs11030096 | 11 | 27622119 | T | 0.54 | 0.10 | 0.02 | 1.3E-07 | 0.57 | 0.08 | 0.02 | 6.3E-04 | 0.55 | 0.09 | 0.01 | 1.0E-09 | 0 | 3.85E-01 |
| rs1519479 | 11 | 27624107 | C | 0.53 | 0.10 | 0.02 | 9.6E-08 | 0.56 | 0.08 | 0.02 | 5.3E-04 | 0.54 | 0.09 | 0.01 | 3.1E-10 | 0 | 3.56E-01 |
| rs2203877 | 11 | 27627486 | T | 0.53 | 0.10 | 0.02 | 1.6E-07 | 0.56 | 0.08 | 0.02 | 5.1E-04 | 0.55 | 0.09 | 0.01 | 4.2E-10 | 0 | 4.12E-01 |
| rs11030101 | 11 | 27637320 | A | 0.54 | 0.10 | 0.02 | 9.5E-07 | 0.57 | 0.07 | 0.02 | 2.0E-03 | 0.56 | 0.08 | 0.01 | 7.8E-09 | 0 | 3.74E-01 |
| rs7103873 | 11 | 27656893 | G | 0.54 | 0.10 | 0.02 | 6.7E-07 | 0.52 | 0.06 | 0.02 | 5.4E-03 | 0.53 | 0.08 | 0.01 | 2.1E-08 | 25.9 | 2.45E-01 |
| rs2049046 | 11 | 27680351 | T | 0.54 | 0.10 | 0.02 | 6.1E-07 | 0.52 | 0.06 | 0.02 | 6.2E-03 | 0.53 | 0.08 | 0.01 | 2.5E-08 | 30.1 | 2.32E-01 |
| rs2306862 | 11 | 67934086 | C | 0.83 | 0.14 | 0.03 | 8.3E-08 | 0.88 | 0.08 | 0.03 | 1.7E-02 | 0.85 | 0.12 | 0.02 | 2.1E-08 | 46.9 | 1.70E-01 |
| rs3781586 | 11 | 67955969 | C | 0.83 | 0.14 | 0.03 | 8.2E-08 | 0.87 | 0.08 | 0.03 | 2.2E-02 | 0.85 | 0.11 | 0.02 | 3.3E-08 | 53.5 | 1.42E-01 |
| rs11228258 | 11 | 68010904 | C | 0.72 | 0.12 | 0.02 | 1.2E-08 | 0.75 | 0.06 | 0.03 | 1.8E-02 | 0.73 | 0.10 | 0.02 | 1.1E-08 | 69.4 | 7.05E-02 |
| rs7116994 | 11 | 68011203 | C | 0.72 | 0.12 | 0.02 | 1.2E-08 | 0.75 | 0.06 | 0.03 | 1.6E-02 | 0.73 | 0.10 | 0.02 | 9.8E-09 | 68.4 | 7.52E-02 |
| rs12294029 | 11 | 68013635 | A | 0.72 | 0.12 | 0.02 | 1.2E-08 | 0.75 | 0.06 | 0.03 | 1.6E-02 | 0.73 | 0.10 | 0.02 | 9.8E-09 | 68.4 | 7.52E-02 |
| rs7126340 | 11 | 68013869 | C | 0.72 | 0.12 | 0.02 | 1.8E-08 | 0.74 | 0.06 | 0.03 | 1.6E-02 | 0.73 | 0.10 | 0.02 | 1.2E-08 | 67.4 | 8.01E-02 |
| **rs12272917** | **11** | **68019946** | **T** | **0.74** | **0.13** | **0.02** | **4.0E-09** | **0.76** | **0.08** | **0.03** | **2.5E-03** | **0.75** | **0.11** | **0.02** | **1.3E-10** | **53** | **1.45E-01** |
| rs11228262 | 11 | 68031483 | G | 0.73 | 0.13 | 0.02 | 9.1E-09 | 0.76 | 0.07 | 0.03 | 4.9E-03 | 0.74 | 0.10 | 0.02 | 9.8E-10 | 58.2 | 1.22E-01 |
| rs10896334 | 11 | 68038070 | C | 0.72 | 0.12 | 0.02 | 1.3E-08 | 0.75 | 0.07 | 0.03 | 1.0E-02 | 0.73 | 0.10 | 0.02 | 5.4E-09 | 63.8 | 9.64E-02 |
| rs10896337 | 11 | 68040812 | T | 0.72 | 0.12 | 0.02 | 1.3E-08 | 0.75 | 0.06 | 0.03 | 1.6E-02 | 0.73 | 0.10 | 0.02 | 9.8E-09 | 68.4 | 7.52E-02 |
| rs11228269 | 11 | 68046372 | A | 0.72 | 0.12 | 0.02 | 1.4E-08 | 0.75 | 0.06 | 0.03 | 1.3E-02 | 0.73 | 0.10 | 0.02 | 8.4E-09 | 67.4 | 8.01E-02 |
| rs7925275 | 11 | 68047009 | T | 0.74 | 0.13 | 0.02 | 1.1E-08 | 0.76 | 0.07 | 0.03 | 5.0E-03 | 0.74 | 0.10 | 0.02 | 1.2E-09 | 56.5 | 1.29E-01 |
| rs6591340 | 11 | 68054212 | A | 0.74 | 0.13 | 0.02 | 1.1E-08 | 0.76 | 0.07 | 0.03 | 5.7E-03 | 0.75 | 0.10 | 0.02 | 1.4E-09 | 58.2 | 1.22E-01 |
| rs7106259 | 11 | 68055088 | T | 0.72 | 0.12 | 0.02 | 1.5E-08 | 0.75 | 0.06 | 0.03 | 1.5E-02 | 0.73 | 0.10 | 0.02 | 1.2E-08 | 67.4 | 8.01E-02 |
| rs4316515 | 11 | 68065416 | A | 0.74 | 0.13 | 0.02 | 9.4E-09 | 0.76 | 0.07 | 0.03 | 5.8E-03 | 0.74 | 0.10 | 0.02 | 1.2E-09 | 59.7 | 1.15E-01 |
| rs7944870 | 11 | 68065684 | C | 0.72 | 0.12 | 0.02 | 1.3E-08 | 0.75 | 0.07 | 0.03 | 9.5E-03 | 0.74 | 0.10 | 0.02 | 4.6E-09 | 62.5 | 1.02E-01 |
| rs948315 | 11 | 68065716 | T | 0.72 | 0.12 | 0.02 | 1.3E-08 | 0.74 | 0.06 | 0.03 | 2.8E-02 | 0.73 | 0.09 | 0.02 | 2.3E-08 | 73.8 | 5.07E-02 |
| rs948316 | 11 | 68066246 | G | 0.74 | 0.13 | 0.02 | 9.4E-09 | 0.76 | 0.07 | 0.03 | 5.7E-03 | 0.74 | 0.10 | 0.02 | 1.2E-09 | 59.7 | 1.15E-01 |
| rs10896341 | 11 | 68069756 | G | 0.72 | 0.12 | 0.02 | 1.3E-08 | 0.75 | 0.07 | 0.03 | 8.2E-03 | 0.74 | 0.10 | 0.02 | 4.0E-09 | 61.1 | 1.09E-01 |
| rs7104345 | 11 | 68073348 | G | 0.74 | 0.13 | 0.02 | 9.2E-09 | 0.76 | 0.07 | 0.03 | 5.7E-03 | 0.74 | 0.10 | 0.02 | 1.2E-09 | 59.7 | 1.15E-01 |
| rs12284933 | 11 | 68076065 | G | 0.75 | 0.13 | 0.02 | 1.4E-08 | 0.77 | 0.08 | 0.03 | 4.6E-03 | 0.76 | 0.11 | 0.02 | 8.9E-10 | 54.4 | 1.39E-01 |
| rs11228284 | 11 | 68079343 | A | 0.74 | 0.13 | 0.02 | 9.0E-09 | 0.76 | 0.07 | 0.03 | 5.7E-03 | 0.74 | 0.10 | 0.02 | 1.2E-09 | 59.7 | 1.15E-01 |
| rs12271290 | 11 | 68082812 | C | 0.72 | 0.12 | 0.02 | 1.2E-08 | 0.75 | 0.07 | 0.03 | 1.2E-02 | 0.73 | 0.10 | 0.02 | 6.3E-09 | 65.1 | 9.07E-02 |
| rs7102898 | 11 | 68085446 | G | 0.74 | 0.13 | 0.02 | 8.9E-09 | 0.76 | 0.07 | 0.03 | 5.7E-03 | 0.74 | 0.10 | 0.02 | 1.2E-09 | 59.7 | 1.15E-01 |
| rs2155730 | 11 | 68086050 | T | 0.72 | 0.12 | 0.02 | 1.2E-08 | 0.74 | 0.06 | 0.03 | 1.8E-02 | 0.73 | 0.10 | 0.02 | 1.1E-08 | 71.7 | 6.03E-02 |
| rs7109294 | 11 | 68088669 | T | 0.74 | 0.13 | 0.02 | 8.7E-09 | 0.76 | 0.07 | 0.03 | 5.7E-03 | 0.74 | 0.10 | 0.02 | 1.2E-09 | 59.7 | 1.15E-01 |
| rs2282563 | 11 | 68089776 | C | 0.74 | 0.13 | 0.02 | 8.7E-09 | 0.76 | 0.07 | 0.03 | 5.7E-03 | 0.74 | 0.10 | 0.02 | 1.2E-09 | 59.7 | 1.15E-01 |
| rs3740631 | 11 | 68098298 | T | 0.74 | 0.13 | 0.02 | 8.7E-09 | 0.76 | 0.07 | 0.03 | 5.6E-03 | 0.74 | 0.10 | 0.02 | 1.2E-09 | 59.7 | 1.15E-01 |
| rs11228287 | 11 | 68106330 | A | 0.74 | 0.13 | 0.02 | 8.6E-09 | 0.76 | 0.07 | 0.03 | 5.6E-03 | 0.75 | 0.10 | 0.02 | 1.2E-09 | 59.7 | 1.15E-01 |
| rs10896347 | 11 | 68109703 | G | 0.74 | 0.13 | 0.02 | 8.6E-09 | 0.76 | 0.07 | 0.03 | 5.6E-03 | 0.74 | 0.10 | 0.02 | 1.2E-09 | 59.7 | 1.15E-01 |
| rs7118897 | 11 | 68117256 | G | 0.74 | 0.13 | 0.02 | 8.6E-09 | 0.75 | 0.06 | 0.03 | 1.5E-02 | 0.74 | 0.10 | 0.02 | 4.6E-09 | 70.4 | 6.61E-02 |
| rs6591344 | 11 | 68117449 | G | 0.74 | 0.13 | 0.02 | 8.6E-09 | 0.76 | 0.07 | 0.03 | 5.3E-03 | 0.75 | 0.10 | 0.02 | 9.8E-10 | 58.2 | 1.22E-01 |
| rs7123564 | 11 | 68118740 | C | 0.72 | 0.12 | 0.03 | 5.7E-07 | 0.76 | 0.07 | 0.03 | 4.7E-03 | 0.74 | 0.10 | 0.02 | 4.1E-08 | 47.2 | 1.69E-01 |
| rs7127948 | 11 | 68119638 | G | 0.74 | 0.13 | 0.02 | 8.6E-09 | 0.76 | 0.07 | 0.03 | 5.3E-03 | 0.75 | 0.10 | 0.02 | 9.8E-10 | 58.2 | 1.22E-01 |
| rs3758643 | 11 | 68123769 | C | 0.74 | 0.13 | 0.02 | 8.7E-09 | 0.76 | 0.07 | 0.03 | 5.7E-03 | 0.75 | 0.10 | 0.02 | 1.2E-09 | 59.7 | 1.15E-01 |
| rs12283755 | 11 | 68128234 | A | 0.74 | 0.13 | 0.02 | 8.8E-09 | 0.76 | 0.07 | 0.03 | 5.0E-03 | 0.75 | 0.10 | 0.02 | 9.8E-10 | 58.2 | 1.22E-01 |
| rs7104877 | 11 | 68134178 | A | 0.72 | 0.12 | 0.02 | 1.4E-08 | 0.74 | 0.07 | 0.03 | 9.7E-03 | 0.73 | 0.10 | 0.02 | 3.8E-09 | 63.8 | 9.64E-02 |
| rs2236708 | 11 | 68134621 | G | 0.74 | 0.13 | 0.02 | 1.5E-08 | 0.76 | 0.07 | 0.03 | 4.9E-03 | 0.75 | 0.10 | 0.02 | 2.2E-09 | 58.1 | 1.22E-01 |
| rs11228292 | 11 | 68136348 | G | 0.74 | 0.13 | 0.02 | 1.5E-08 | 0.76 | 0.07 | 0.03 | 4.9E-03 | 0.75 | 0.10 | 0.02 | 2.2E-09 | 58.1 | 1.22E-01 |
| rs4988291 | 11 | 68138183 | G | 0.74 | 0.13 | 0.02 | 1.5E-08 | 0.76 | 0.07 | 0.03 | 4.8E-03 | 0.75 | 0.10 | 0.02 | 2.2E-09 | 58.1 | 1.22E-01 |
| rs11228293 | 11 | 68141222 | T | 0.74 | 0.13 | 0.02 | 1.6E-08 | 0.76 | 0.08 | 0.03 | 4.7E-03 | 0.75 | 0.10 | 0.02 | 1.9E-09 | 56.5 | 1.29E-01 |
| rs7102273 | 11 | 68142155 | T | 0.73 | 0.13 | 0.02 | 2.5E-08 | 0.75 | 0.07 | 0.03 | 1.2E-02 | 0.74 | 0.10 | 0.02 | 1.3E-08 | 67.2 | 8.10E-02 |
| **rs884205** | **18** | **58205837** | **C** | **0.72** | **0.09** | **0.02** | **5.4E-05** | **0.80** | **0.12** | **0.03** | **3.9E-05** | **0.75** | **0.10** | **0.02** | **1.8E-08** | **0** | **4.15E-01** |

(CHR) = chromosome number; (POS) = position in the genome based on hg18; (EAF) = effect allele frequency; (*β*) = estimates of effect size expressed as adjusted SD per copy of the effect allele (EA); (SE) = standard error of *β*; (*P*) = *P*-value; (I^2^) = Cochran’s Q statistic evaluating heterogeneity and (*P*_HET_) = evidence of heterogeneity. The SNP that showed the strongest evidence of association at each locus is displayed in bold font.
