# Supplementary material for: Phenotypic Dissection of Bone Mineral Density Reveals Skeletal Site Specificity and Facilitates the Identification of Novel Loci in the Genetic Regulation of Bone Mass Attainment
Source: PLoS Genet. 2014 Jun 19;10(6):e1004423. doi: 10.1371/journal.pgen.1004423 (PMC4063697; doi:10.1371/journal.pgen.1004423)
Supplement: Table S8 — Comparison of published BMD SNPs with results from the total-body less head, lower limb, upper limb and skull BMD GWAS. (LS-BMD) = lumbar spine BMD; (FN-BMD) = femoral neck BMD; (F-BMD) = forearm BMD; (TBLH-BMD) = total-body less head BMD; (LL-BMD) = lower limb BMD; (UL-BMD) = upper limb BMD; (SK-BMD) = skull BMD; (POSITION) = location in the genome based on hg18; (GENE) = closest gene; (PMID) = accession number of the publication in Pubmed from which the summary statistics were obtained; (EA) = effect allele; (EAF) = effect allele frequency; (β) = estimates of effect size expressed as adjusted SD per copy of the effect allele (EA); (SE) = standard error of β and (P) = pvalue; We failed to obtain estimates for: rs9287237 (1q43, FMN2); rs7017914 (8q13.3, XKR9); rs7851693 (9q34.11, FUBP3) and rs5934507 (Xp22.31, FAM9B) as they were not imputed in the GEN-R dataset. Note for rs9287237* (FMN2) and rs271170* (LOC285735/EYA4) the summary statistics were obtained from a study performed by Patenoster et. al. 2013 and represent the effect sizes and evidence of association for these SNPs with volumetric trabecular (LS-BMD column) and cortical BMD (FN-BMD column). (DOCX) [file pgen.1004423.s024.docx]

**Table S8**. Comparison of published BMD SNPs with results from the total-body less head, lower limb, upper limb and skull BMD GWAS.

|  | | | | | | | **LS-BMD** | | **FN-BMD** | | **TBLH-BMD** | | **LL-BMD** | | **UL-BMD** | | **SK-BMD** | |
| --- | --- | --- | --- | --- | --- | --- | --- | --- | --- | --- | --- | --- | --- | --- | --- | --- | --- | --- |
| **RSID** | **POSITION** | **GENE** | **LOCUS** | **EA** | **EAF** | **PMID** | ***β*** | ***P*** | ***β*** | ***P*** | ***β*** | ***P*** | ***β*** | ***P*** | ***β*** | ***P*** | ***β*** | ***P*** |
| rs17482952 | 68411973 | *WLS* | 1p31.3 | A | 0.07 | 22504420 | 0.07 | 1.69E-08 | 0.08 | 1.31E-11 | 0.10 | 3.66E-04 | 0.08 | 4.16E-03 | 0.05 | 6.13E-02 | 0.10 | 3.92E-04 |
| rs12407028 | 68420304 | *WLS* | 1p31.3 | T | 0.6 | 22504420 | 0.08 | 3.00E-45 | 0.06 | 3.40E-23 | 0.04 | 4.10E-03 | 0.03 | 2.75E-02 | 0.02 | 1.15E-01 | 0.01 | 7.42E-01 |
| rs7521902 | 22363311 | *WNT4* | 1p36.12 | A | 0.31 | 22504420 | -0.05 | 1.00E-10 | -0.04 | 2.80E-09 | **-0.08** | **1.58E-05** | -0.05 | 2.04E-03 | **-0.09** | **3.81E-07** | **-0.12** | **3.32E-12** |
| rs6426749 | 22584060 | *ZBTB40* | 1p36.12 | C | 0.17 | 22504420 | 0.1 | 1.80E-44 | 0.11 | 7.00E-57 | 0.07 | 2.27E-04 | 0.06 | 1.19E-03 | 0.04 | 2.35E-02 | 0.06 | 2.43E-03 |
| rs479336 | 170466196 | *DNM3* | 1q24.3 | T | 0.74 | 22504420 | -0.03 | 2.14E-05 | -0.04 | 9.00E-15 | -0.02 | 1.88E-01 | -0.02 | 2.80E-01 | -0.03 | 1.14E-01 | -0.02 | 2.03E-01 |
| **rs9287237*** | **238663837** | ***FMN2*** | **1q43** | **T** | **0.15** | **23437003** | 0.19^*^ | 1.90E-09 | 0.2 | 3.20E-01 | *n/a* | *n/a* | *n/a* | *n/a* | *n/a* | *n/a* | *n/a* | *n/a* |
| rs4233949 | 54513211 | *SPTBN1* | 2p16.2 | C | 0.38 | 22504420 | 0.05 | 2.00E-18 | 0.02 | 5.91E-06 | 0.02 | 2.34E-01 | *0.00* | *9.89E-01* | 0.03 | 3.14E-02 | 0.03 | 6.16E-02 |
| rs7584262 | 42104053 | *PKDCC* | 2p21 | T | 0.23 | 22504420 | 0.01 | 7.00E-02 | 0.04 | 1.00E-09 | 0.06 | 1.22E-03 | 0.06 | 1.21E-03 | 0.05 | 4.94E-03 | 0.03 | 6.95E-02 |
| rs17040773 | 112216506 | *ANAPC1* | 2q13 | A | 0.76 | 22504420 | 0.01 | 1.90E-01 | 0.04 | 2.00E-09 | 0.00 | 8.28E-01 | 0.01 | 5.17E-01 | 0.01 | 5.21E-01 | *-0.01* | *5.62E-01* |
| rs1878526 | 118755068 | *INSIG2* | 2q14.2 | A | 0.22 | 22504420 | 0.04 | 1.00E-10 | 0 | 7.90E-01 | 0.00 | 8.23E-01 | *-0.01* | *6.93E-01* | *-0.01* | *5.58E-01* | 0.02 | 2.89E-01 |
| rs1346004 | 166309292 | *GALNT3* | 2q24.3 | A | 0.5 | 22504420 | -0.06 | 4.00E-30 | -0.05 | 1.00E-25 | **-0.09** | **4.51E-09** | **-0.08** | **5.01E-07** | **-0.08** | **1.09E-07** | -0.03 | 7.16E-02 |
| rs430727 | 41103568 | *CTNNB1* | 3p22.1 | T | 0.48 | 22504420 | -0.05 | 1.50E-18 | -0.06 | 4.00E-25 | -0.05 | 6.53E-04 | -0.04 | 1.82E-02 | -0.03 | 3.01E-02 | -0.03 | 3.38E-02 |
| rs1026364 | 114852700 | *KIAA2018* | 3q13.2 | T | 0.37 | 22504420 | 0.02 | 7.57E-04 | 0.03 | 4.00E-10 | 0.03 | 4.59E-02 | 0.02 | 2.57E-01 | 0.01 | 3.44E-01 | 0.01 | 5.79E-01 |
| rs344081 | 158038678 | *LEKR1* | 3q25.31 | T | 0.87 | 22504420 | 0.06 | 4.00E-12 | 0.04 | 2.22E-06 | 0.02 | 2.65E-01 | 0.01 | 5.69E-01 | 0.02 | 3.68E-01 | 0.07 | 4.71E-04 |
| rs3755955 | 984414 | *IDUA* | 4p16.3 | A | 0.16 | 22504420 | -0.06 | 5.00E-15 | -0.06 | 1.40E-15 | -0.03 | 2.09E-01 | -0.03 | 1.96E-01 | -0.03 | 1.29E-01 | -0.03 | 1.45E-01 |
| rs6532023 | 88992873 | *MEPE* | 4q22.1 | T | 0.34 | 22504420 | 0.06 | 1.00E-27 | 0.06 | 5.00E-26 | **0.07** | **2.66E-06** | 0.06 | 1.09E-04 | 0.04 | 3.31E-03 | 0.06 | 2.25E-04 |
| rs1366594 | 88411817 | *MEF2C* | 5q14.3 | A | 0.54 | 22504420 | 0.01 | 1.00E-02 | 0.08 | 4.00E-61 | 0.03 | 2.34E-02 | 0.05 | 2.49E-03 | 0.03 | 6.62E-02 | 0.03 | 5.41E-02 |
| rs11755164 | 44747162 | *SUPT3H* | 6p21.1 | T | 0.4 | 22504420 | -0.04 | 6.00E-11 | -0.01 | 5.00E-02 | -0.02 | 3.39E-01 | -0.02 | 2.69E-01 | -0.02 | 2.50E-01 | -0.02 | 2.37E-01 |
| rs9466056 | 21492592 | *CDKAL1* | 6p22.3 | A | 0.38 | 22504420 | -0.04 | 3.00E-13 | -0.03 | 3.00E-08 | -0.03 | 6.16E-02 | -0.02 | 2.88E-01 | -0.03 | 2.92E-02 | -0.02 | 1.77E-01 |
| rs13204965 | 127208765 | *RSPO3* | 6q22.33 | A | 0.76 | 22504420 | 0.04 | 3.60E-10 | 0.04 | 8.00E-12 | **0.09** | **1.75E-06** | 0.08 | 5.20E-05 | **0.11** | **1.25E-08** | 0.05 | 9.85E-03 |
| **rs271170*** | **133357497** | ***EYA4*** | **6q23.2** | **T** | **0.33** | **23437003** | -0.02 | 5.40E-01 | -0.11 | 2.70E-12^**^ | -0.02 | 2.16E-01 | -0.02 | 2.29E-01 | -0.04 | 5.28E-03 | -0.13 | 3.12E-16 |
| rs4869742 | 151949441 | *CCDC170* | 6q25.1 | T | 0.31 | 22504420 | -0.08 | 4.00E-35 | -0.05 | 4.00E-18 | -0.01 | 6.83E-01 | -0.01 | 6.26E-01 | -0.01 | 6.94E-01 | -0.01 | 7.19E-01 |
| rs7751941 | 151988351 | *CCDC170* | 6q25.1 | A | 0.23 | 22504420 | -0.08 | 1.99E-24 | -0.04 | 1.59E-09 | *0.02* | *3.16E-01* | *0.02* | *2.15E-01* | *0.01* | *5.42E-01* | -0.02 | 2.75E-01 |
| rs10226308 | 37904947 | *NME8* | 7p14.1 | A | 0.84 | 22504420 | -0.06 | 6.00E-13 | -0.02 | 2.00E-02 | *0.00* | *8.00E-01* | *0.02* | *3.69E-01* | -0.01 | 7.73E-01 | -0.03 | 1.26E-01 |
| rs6959212 | 38094851 | *STARD3NL* | 7p14.1 | T | 0.32 | 22504420 | -0.07 | 4.00E-38 | -0.04 | 1.20E-13 | -0.02 | 2.18E-01 | *0.01* | *4.07E-01* | *0.02* | *2.22E-01* | -0.02 | 3.30E-01 |
| rs4727338 | 95958611 | *SHFM1* | 7q21.3 | C | 0.67 | 22504420 | 0.07 | 2.00E-35 | 0.08 | 8.00E-48 | **0.07** | **1.53E-05** | **0.07** | **4.93E-05** | 0.05 | 2.41E-03 | 0.03 | 1.16E-01 |
| rs13245690 | 120572300 | *CPED1* | 7q31.31 | A | 0.65 | 22504420 | 0.05 | 2.00E-11 | 0.02 | 8.20E-04 | **0.09** | **1.01E-08** | 0.03 | 4.06E-02 | **0.19** | **1.03E-36** | **0.17** | **2.88E-28** |
| rs3801387 | 120762001 | *WNT16* | 7q31.31 | A | 0.74 | 22504420 | -0.09 | 3.00E-51 | -0.08 | 5.00E-40 | **-0.15** | **5.44E-19** | **-0.10** | **8.82E-09** | **-0.18** | **4.97E-27** | **-0.09** | **4.71E-08** |
| rs7812088 | 150550762 | *ABCF2* | 7q36.1 | A | 0.13 | 22504420 | 0.04 | 2.20E-07 | 0.05 | 7.00E-09 | 0.07 | 3.21E-03 | 0.05 | 2.41E-02 | 0.08 | 4.60E-04 | 0.06 | 1.03E-02 |
| rs7017914 | 71753757 | *XKR9* | 8q13.3 | A | 0.49 | 22504420 | 0.01 | 2.60E-01 | 0.03 | 2.00E-07 | n/a | n/a | n/a | n/a | n/a | n/a | n/a | n/a |
| rs2062377 | 120076601 | *TNFRSF11B* | 8q24.12 | A | 0.57 | 22504420 | -0.08 | 3.00E-39 | -0.06 | 9.00E-25 | -0.02 | 1.39E-01 | -0.01 | 5.49E-01 | -0.03 | 3.81E-02 | **-0.09** | **4.67E-10** |
| rs7851693 | 132468648 | *FUBP3* | 9q34.11 | C | 0.64 | 22504420 | 0.03 | 6.00E-08 | 0.05 | 3.00E-22 | *n/a* | *n/a* | *n/a* | *n/a* | *n/a* | *n/a* | *n/a* | *n/a* |
| rs3905706 | 28519948 | *MPP7* | 10p12.1 | T | 0.22 | 22504420 | 0.05 | 2.00E-16 | 0.01 | 3.00E-02 | 0.04 | 2.45E-02 | 0.02 | 1.89E-01 | 0.04 | 1.38E-02 | **0.08** | **2.93E-06** |
| rs1373004 | 54097831 | *MBL2* | 10q21.1 | T | 0.13 | 22504420 | -0.06 | 2.00E-12 | -0.04 | 1.45E-08 | -0.04 | 9.91E-02 | -0.03 | 1.32E-01 | *0.01* | *7.26E-01* | -0.06 | 7.22E-03 |
| rs7071206 | 79071322 | *KCNMA1* | 10q22.3 | T | 0.78 | 22504420 | -0.06 | 5.00E-19 | *0* | *8.10E-01* | -0.02 | 1.88E-01 | *0.02* | *2.12E-01* | -0.03 | 9.15E-02 | -0.02 | 3.27E-01 |
| rs7084921 | 101803792 | *CPN1* | 10q24.2 | T | 0.39 | 22504420 | 0.03 | 9.00E-07 | 0.03 | 9.00E-10 | 0.04 | 1.67E-02 | 0.04 | 1.16E-02 | 0.03 | 9.45E-02 | 0.04 | 1.43E-02 |
| rs7932354 | 46678797 | *ARHGAP1* | 11p11.2 | T | 0.31 | 22504420 | 0.04 | 5.40E-12 | 0.05 | 5.00E-18 | 0.02 | 1.51E-01 | 0.03 | 5.72E-02 | *-0.01* | *4.99E-01* | 0.01 | 4.83E-01 |
| rs10835187 | 27462253 | *LIN7C* | 11p14.1 | T | 0.55 | 22504420 | -0.03 | 5.00E-08 | -0.01 | 3.00E-02 | -0.05 | 7.11E-04 | -0.04 | 3.10E-03 | -0.04 | 5.53E-03 | **-0.13** | **1.63E-17** |
| rs163879 | 30908250 | *DCDC5* | 11p14.1 | T | 0.68 | 22504420 | -0.04 | 2.00E-11 | -0.03 | 2.00E-08 | 0.00 | 8.19E-01 | 0.00 | 8.09E-01 | -0.01 | 4.62E-01 | -0.01 | 4.58E-01 |
| rs7108738 | 15666660 | *SOX6* | 11p15.2 | T | 0.83 | 22504420 | -0.03 | 2.14E-06 | -0.08 | 1.00E-32 | -0.05 | 9.54E-03 | -0.05 | 9.95E-03 | -0.06 | 2.08E-03 | -0.05 | 1.64E-02 |
| rs3736228 | 67957871 | *LRP5* | 11q13.2 | T | 0.16 | 22504420 | -0.08 | 2.00E-26 | -0.05 | 4.80E-11 | -0.08 | 2.24E-04 | -0.06 | 1.01E-02 | -0.06 | 4.45E-03 | **-0.11** | **4.63E-07** |
| rs7953528 | 27908426 | *KLHDC5* | 12p11.22 | A | 0.18 | 22504420 | *-0.01* | *1.30E-01* | 0.05 | 1.87E-12 | 0.04 | 6.41E-02 | 0.05 | 7.29E-03 | 0.05 | 2.09E-02 | *-0.03* | *9.55E-02* |
| rs2887571 | 1508432 | *ERC1* | 12p13.33 | A | 0.76 | 22504420 | -0.04 | 6.00E-12 | -0.03 | 6.50E-09 | -0.04 | 1.63E-02 | -0.05 | 6.45E-03 | -0.01 | 5.76E-01 | -0.01 | 5.36E-01 |
| rs12821008 | 47760872 | *DHH* | 12q13.12 | T | 0.39 | 22504420 | 0.05 | 1.00E-15 | 0.03 | 3.30E-07 | 0.00 | 7.95E-01 | 0.00 | 9.36E-01 | *-0.02* | *2.34E-01* | 0.02 | 2.18E-01 |
| rs2016266 | 52014222 | *SP7* | 12q13.13 | A | 0.68 | 22504420 | -0.05 | 3.00E-20 | -0.03 | 3.70E-10 | -0.06 | 3.70E-04 | -0.04 | 1.78E-02 | **-0.06** | **4.23E-05** | -0.05 | 3.52E-03 |
| rs736825 | 52703843 | *HOXC6* | 12q13.13 | C | 0.56 | 22504420 | 0.05 | 8.00E-16 | 0.04 | 1.00E-09 | 0.04 | 2.84E-02 | 0.02 | 2.74E-01 | 0.03 | 6.61E-02 | 0.02 | 2.99E-01 |
| rs1053051 | 105891355 | *C12orf23* | 12q23.3 | T | 0.52 | 22504420 | -0.03 | 8.00E-08 | -0.03 | 1.00E-09 | -0.04 | 1.58E-02 | -0.04 | 1.65E-02 | -0.02 | 8.68E-02 | -0.04 | 4.09E-03 |
| rs9533090 | 41849449 | *AKAP11* | 13q14.11 | T | 0.49 | 22504420 | -0.1 | 5.00E-68 | -0.05 | 5.00E-23 | -0.02 | 2.48E-01 | -0.01 | 3.71E-01 | 0.00 | 8.39E-01 | 0.04 | 5.85E-03 |
| rs1286083 | 90512532 | *RPS6KA5* | 14q32.11 | T | 0.81 | 22504420 | -0.05 | 1.80E-14 | -0.05 | 2.00E-15 | -0.07 | 6.00E-05 | -0.08 | 5.20E-05 | -0.05 | 3.19E-03 | -0.01 | 4.53E-01 |
| rs11623869 | 102953386 | *MARK3* | 14q32.32 | T | 0.35 | 22504420 | -0.04 | 5.00E-11 | -0.04 | 5.00E-16 | *0.01* | *7.00E-01* | *0.00* | *9.82E-01* | *0.01* | *5.03E-01* | *0.01* | *5.03E-01* |
| rs4985155 | 15036960 | *NTAN1* | 16p13.11 | A | 0.67 | 22504420 | -0.03 | 2.00E-09 | -0.03 | 2.00E-10 | -0.01 | 5.68E-01 | 0.00 | 7.76E-01 | -0.01 | 7.23E-01 | -0.03 | 6.09E-02 |
| rs9921222 | 315783 | *AXIN1* | 16p13.3 | T | 0.48 | 22504420 | -0.04 | 1.00E-16 | -0.04 | 5.00E-12 | -0.06 | 1.13E-04 | -0.05 | 1.85E-03 | -0.04 | 7.64E-03 | -0.06 | 7.95E-05 |
| rs13336428 | 1472464 | *PTX4* | 16p13.3 | A | 0.43 | 22504420 | -0.04 | 1.60E-13 | -0.04 | 1.00E-16 | -0.06 | 2.34E-04 | -0.06 | 5.66E-05 | -0.04 | 1.18E-02 | -0.03 | 2.24E-02 |
| rs1564981 | 49543809 | *CYLD* | 16q12.1 | A | 0.5 | 22504420 | -0.04 | 2.00E-10 | -0.02 | 4.38E-05 | -0.03 | 3.08E-02 | -0.02 | 9.39E-02 | -0.01 | 4.41E-01 | *0.01* | *3.87E-01* |
| rs1566045 | 49579304 | *SALL1* | 16q12.1 | T | 0.8 | 22504420 | -0.01 | 4.00E-02 | -0.06 | 2.00E-22 | -0.03 | 7.21E-02 | -0.03 | 8.20E-02 | -0.01 | 7.37E-01 | -0.01 | 6.27E-01 |
| rs10048146 | 85268161 | *FOXL1* | 16q24.1 | A | 0.8 | 22504420 | 0.05 | 3.00E-11 | 0.05 | 1.00E-14 | 0.02 | 2.59E-01 | 0.05 | 1.75E-02 | 0.00 | 9.62E-01 | 0.01 | 4.62E-01 |
| rs4790881 | 2015682 | *SMG6* | 17p13.3 | A | 0.69 | 22504420 | 0.03 | 3.40E-09 | 0.05 | 1.00E-18 | 0.03 | 3.65E-02 | 0.02 | 2.29E-01 | 0.05 | 9.78E-04 | 0.04 | 2.47E-02 |
| rs4792909 | 39154350 | *SOST* | 17q21.31 | T | 0.37 | 22504420 | 0.04 | 9.40E-10 | 0.04 | 2.00E-11 | 0.03 | 4.67E-02 | 0.01 | 5.21E-01 | 0.02 | 1.97E-01 | 0.04 | 1.62E-02 |
| rs227584 | 39581073 | *C17orf53* | 17q21.31 | A | 0.7 | 22504420 | -0.04 | 9.90E-10 | -0.06 | 3.00E-24 | -0.03 | 1.08E-01 | -0.02 | 3.11E-01 | -0.02 | 2.66E-01 | -0.02 | 2.09E-01 |
| rs1864325 | 41333623 | *MAPT* | 17q21.31 | T | 0.22 | 22504420 | -0.04 | 5.00E-11 | -0.03 | 7.47E-05 | -0.06 | 1.48E-03 | -0.06 | 9.03E-04 | -0.04 | 2.07E-02 | *0.03* | *7.54E-02* |
| rs7217932 | 67460611 | *SOX9* | 17q24.3 | A | 0.46 | 22504420 | 0.01 | 8.00E-02 | 0.03 | 2.00E-11 | 0.02 | 1.96E-01 | 0.03 | 2.89E-02 | 0.02 | 1.35E-01 | *-0.01* | *3.39E-01* |
| rs4796995 | 13698574 | *FAM210A* | 18p11.21 | A | 0.63 | 22504420 | 0.02 | 6.65E-04 | 0.03 | 5.00E-08 | 0.01 | 4.30E-01 | 0.02 | 2.44E-01 | 0.00 | 8.37E-01 | 0.01 | 5.33E-01 |
| rs884205 | 58205837 | *TNFRSF11A* | 18q21.33 | A | 0.27 | 22504420 | -0.05 | 2.00E-17 | -0.04 | 3.00E-10 | -0.03 | 1.10E-01 | 0.00 | 8.58E-01 | -0.02 | 2.11E-01 | **-0.10** | **1.84E-08** |
| rs10416218 | 38290967 | *GPATCH1* | 19q13.11 | T | 0.73 | 22504420 | -0.04 | 7.00E-11 | -0.03 | 5.50E-08 | -0.02 | 3.29E-01 | -0.02 | 3.44E-01 | 0.00 | 7.65E-01 | -0.01 | 6.60E-01 |
| rs3790160 | 10587988 | *JAG1* | 20p12.2 | T | 0.5 | 22504420 | 0.05 | 3.00E-19 | 0.04 | 3.60E-12 | 0.01 | 7.24E-01 | *0.00* | *7.78E-01* | *0.00* | *8.57E-01* | 0.02 | 1.54E-01 |
| rs5934507 | 8877206 | *FAM9B* | Xp22.31 | A | 0.73 | 22504420 | -0.06 | 1.18E-08 | -0.04 | 1.64E-04 | *n/a* | *n/a* | *n/a* | *n/a* | *n/a* | *n/a* | *n/a* | *n/a* |

(LS-BMD) = lumbar spine BMD; (FN-BMD) = femoral neck BMD; (F-BMD) = forearm BMD; (TBLH-BMD) = total-body less head BMD; (LL-BMD) = lower limb BMD; (UL-BMD) = upper limb BMD; (SK-BMD) = skull BMD; (POSITION) = location in the genome based on hg18; (GENE) = closest gene; (PMID) = accession number of the publication in Pubmed from which the summary statistics were obtained; (EA) = effect allele; (EAF) = effect allele frequency; (β) = estimates of effect size expressed as adjusted SD per copy of the effect allele (EA); (SE) = standard error of *β* and (*P*) = pvalue; We failed to obtain estimates for: rs9287237 (1q43, *FMN2*); rs7017914 (8q13.3, *XKR9*); rs7851693 (9q34.11, *FUBP3*) and rs5934507 (Xp22.31, *FAM9B*) as they were not imputed in the GEN-R dataset. Note for rs9287237^*^ (*FMN2*) and rs271170^*^ (*LOC285735 / EYA4*) the summary statistics were obtained from a study performed by Patenoster *et. al.* 2013 and represent the effect sizes and evidence of association for these SNPs with volumetric trabecular (LS-BMD column) and cortical BMD (FN-BMD column).
