# Supplementary material for: Phenotypic Dissection of Bone Mineral Density Reveals Skeletal Site Specificity and Facilitates the Identification of Novel Loci in the Genetic Regulation of Bone Mass Attainment
Source: PLoS Genet. 2014 Jun 19;10(6):e1004423. doi: 10.1371/journal.pgen.1004423 (PMC4063697; doi:10.1371/journal.pgen.1004423)
Supplement: Table S9 — Sensitivity analysis comparing genome-wide significant SNPs associated with bone mineral density measured at four skeletal sites. (TBLH-BMD) = total-body less head BMD, (LL-BMD) = lower limb BMD, (UL-BMD) = upper limb BMD, (SK-BMD) = skull BMD. (MODEL 0) = GWAS meta-analysis performed on age-, gender-, weight- or height-adjusted BMD, (MODEL 1a) = GWAS meta-analysis performed on age-, gender-, weight- and height-adjusted BMD, (MODEL 1b) = GWAS meta-analysis performed on age-, gender-, weight- and height-adjusted BMD measurements in individuals of European ancestry. (GENE) = closest gene, (POS) = position in the genome based on hg18, (EAF) = effect allele frequency, (β) = estimates of effect size expressed as adjusted SD per copy of the effect allele (EA), (SE) = standard error of β, (P) = pvalue, (I2) = Cochran's Q statistic evaluating heterogeneity, (P HET) = evidence of heterogeneity and *Sample sizes used for SK-BMD genome-wide meta-analysis. **Please note that PTHLH is also located at the 12p11.22 locus containing KLHDC5, RSPO3 is also located at the 6q.22.32 locus containing CENPW, FAM3C and CPED1 are also located at the 7q.31.31 locus containing WNT16, TNFRSF11B is also located at the 8q.24.12 locus containing COLEC10, LGR4 is also located at the 11p14.1 locus containing LIN7C and LRP5 is also located at the 11q13.2 locus containing PPP6R3. (DOCX) [file pgen.1004423.s025.docx]

**Table S9**.Sensitivity analysis comparing genome-wide significant SNPs associated with bone mineral density measured at four skeletal sites.

|  |  |  |  |  |  | **MODEL0 (n = 9416 \ 9385*)** | | | | | **MODEL 1a (n = 9416 \ 9385*)** | | | | | **MODEL1b CEU (n = 7508 \ 7476*)** | | | | |
| --- | --- | --- | --- | --- | --- | --- | --- | --- | --- | --- | --- | --- | --- | --- | --- | --- | --- | --- | --- | --- |
| **TRAIT** | **RSID** | **LOCUS** | **POS** | **GENE** | **EA** | ***β*** | **SE** | ***P*** | **I^2^** | **P_HET_** | ***β*** | **SE** | ***P*** | **I^2^** | **P_HET_** | ***β*** | **SE** | ***P*** | **I^2^** | **P_HET_** |
| **TBLH-BMD** | rs3765350 | 1p36.12 | 22319903 | *WNT4* | A | 0.1073 | 0.0174 | **7.04E-10** | 0 | 9.32E-01 | 0.11 | 0.0174 | **2.85E-10** | 0 | 1.00E+00 | 0.1159 | 0.0197 | **4.07E-09** | 0 | 6.43E-01 |
|  | rs6726821 | 2q24.3 | 166286360 | *GALNT3* | T | 0.0909 | 0.0145 | **3.95E-10** | 0 | 8.11E-01 | 0.0916 | 0.0146 | **3.22E-10** | 0 | 9.73E-01 | 0.0889 | 0.0165 | 6.86E-08 | 0 | 7.64E-01 |
|  | rs7776725 | 7q31.31 | 120820357 | *FAM3C*** | C | 0.1593 | 0.0174 | **5.67E-20** | 54.7 | 1.38E-01 | 0.1613 | 0.0174 | **2.28E-20** | 57.9 | 1.24E-01 | 0.1582 | 0.0199 | **1.73E-15** | 64.7 | 9.24E-02 |
|  | rs7466269 | 9q34.11 | 132453905 | *FUBP3* | A | 0.0843 | 0.0153 | **3.26E-08** | 0 | 4.74E-01 | 0.0812 | 0.0153 | 1.09E-07 | 0 | 5.16E-01 | 0.0756 | 0.0173 | 1.18E-05 | 42 | 1.89E-01 |
|  | rs4420311 | 12p11.22 | 27875457 | *KLHDC5*** | G | 0.085 | 0.0155 | **4.44E-08** | 0 | 7.03E-01 | 0.0843 | 0.0156 | 6.08E-08 | 0 | 6.34E-01 | 0.0872 | 0.0173 | 4.30E-07 | 0 | 4.01E-01 |
|  | rs17536328 | 13q14.11 | 42041029 | *TNFSF11* | T | 0.0864 | 0.015 | **7.58E-09** | 0 | 5.94E-01 | 0.0874 | 0.015 | **5.34E-09** | 0 | 5.94E-01 | 0.0866 | 0.0169 | 3.07E-07 | 0 | 5.64E-01 |
|  | rs754388 | 14q32.12 | 92185163 | *RIN3* | C | 0.1195 | 0.0201 | **2.96E-09** | 36 | 2.11E-01 | 0.1255 | 0.0202 | **5.02E-10** | 21.2 | 2.60E-01 | 0.1219 | 0.0223 | **4.83E-08** | 17.6 | 2.71E-01 |
| **LL-BMD** | rs3765350 | 1p36.12 | 22319903 | *WNT4* | A | 0.0972 | 0.0175 | **2.89E-08** | 0 | 7.12E-01 | 0.0991 | 0.0175 | **1.59E-08** | 0 | 7.12E-01 | 0.1071 | 0.0198 | **6.43E-08** | 0 | 8.71E-01 |
|  | rs2908004 | 7q31.31 | 120757005 | *WNT16*** | A | 0.0999 | 0.015 | **3.01E-11** | 0 | 6.19E-01 | 0.0992 | 0.0151 | **4.49E-11** | 0 | 4.66E-01 | 0.1039 | 0.0172 | **1.45E-09** | 42 | 1.89E-01 |
|  | rs7466269 | 9q34.11 | 132453905 | *FUBP3* | A | 0.0869 | 0.0153 | **1.51E-08** | 0 | 4.57E-01 | 0.0833 | 0.0154 | 6.08E-08 | 0 | 4.77E-01 | 0.0793 | 0.0173 | 4.76E-06 | 34 | 2.18E-01 |
|  | rs4420311 | 12p11.22 | 27875457 | *KLHDC5*** | G | 0.0864 | 0.0156 | **3.21E-08** | 0 | 9.75E-01 | 0.0861 | 0.0157 | **3.80E-08** | 0 | 8.75E-01 | 0.0921 | 0.0173 | 1.07E-07 | 0 | 4.63E-01 |
|  | rs754388 | 14q32.12 | 92185163 | *RIN3* | C | 0.13 | 0.0203 | **1.40E-10** | 0 | 5.26E-01 | 0.1348 | 0.0203 | **3.14E-11** | 0 | 5.75E-01 | 0.1351 | 0.0224 | **1.75E-09** | 0 | 4.89E-01 |
| **UL-BMD** | rs2235529 | 1p36.12 | 22323074 | *WNT4* | C | 0.117 | 0.0205 | **1.21E-08** | 0 | 3.22E-01 | 0.1181 | 0.0205 | **8.73E-09** | 0 | 3.46E-01 | 0.1173 | 0.0229 | 2.99E-07 | 14.7 | 2.79E-01 |
|  | rs6726821 | 2q24.3 | 166286360 | *GALNT3* | T | 0.0828 | 0.0145 | **1.13E-08** | 0 | 7.07E-01 | 0.0818 | 0.0145 | **1.69E-08** | 0 | 7.07E-01 | 0.0789 | 0.0164 | 1.60E-06 | 0 | 8.50E-01 |
|  | rs1262476 | 6q22.32 | 127028689 | *CENPW*** | G | 0.1036 | 0.0175 | **2.93E-09** | 72.3 | 5.76E-02 | 0.1022 | 0.0175 | **4.67E-09** | 59.1 | 1.18E-01 | 0.1045 | 0.0191 | **4.17E-08** | 64.6 | 9.30E-02 |
|  | rs798943 | 7q31.31 | 120546135 | *CPED1*** | G | 0.1949 | 0.0152 | **1.47E-37** | 0 | 5.57E-01 | 0.1931 | 0.0152 | **6.44E-37** | 0 | 6.48E-01 | 0.2016 | 0.0171 | **3.05E-32** | 43.8 | 1.82E-01 |
|  | rs9525638 | 13q14.11 | 42026577 | *TNFSF11* | C | 0.0889 | 0.0149 | **2.47E-09** | 0 | 7.13E-01 | 0.0894 | 0.0149 | **2.04E-09** | 0 | 7.38E-01 | 0.0949 | 0.0169 | **1.92E-08** | 0 | 9.35E-01 |
| **SK-BMD** | rs3920498 | 1p36.12 | 22365474 | *WNT4* | G | 0.1337 | 0.0189 | **1.56E-12** | 0 | 5.01E-01 | 0.1371 | 0.0194 | **1.57E-12** | 0 | 4.31E-01 | 0.139 | 0.0215 | **1.01E-10** | 0 | 4.15E-01 |
|  | rs2130604 | 6q22.32 | 126862254 | *CENPW* | T | 0.1123 | 0.0169 | **3.33E-11** | 0 | 7.48E-01 | 0.1109 | 0.0174 | **1.85E-10** | 0 | 8.42E-01 | 0.1291 | 0.0195 | **3.59E-11** | 27.8 | 2.39E-01 |
|  | rs3012465 | 6q23.2 | 133392629 | *EYA4* | G | 0.1267 | 0.0152 | **8.29E-17** | 0 | 8.96E-01 | 0.1284 | 0.0152 | **3.59E-17** | 0 | 9.74E-01 | 0.1215 | 0.0172 | **1.61E-12** | 0 | 5.48E-01 |
|  | rs13223036 | 7q31.31 | 120534544 | *CPED1*** | T | 0.1687 | 0.0152 | **1.53E-28** | 0 | 9.22E-01 | 0.169 | 0.0152 | **1.51E-28** | 0 | 8.20E-01 | 0.1652 | 0.017 | **3.24E-22** | 0 | 5.39E-01 |
|  | rs2450083 | 8q24.12 | 120132723 | *COLEC10*** | T | 0.1019 | 0.0152 | **2.13E-11** | 0 | 8.20E-01 | 0.1025 | 0.0152 | **1.75E-11** | 0 | 7.95E-01 | 0.0959 | 0.0172 | **2.50E-08** | 0 | 3.47E-01 |
|  | rs10835187 | 11p14.1 | 27462253 | *LIN7C*** | C | 0.1271 | 0.0149 | **1.63E-17** | 41.1 | 1.93E-01 | 0.1272 | 0.0149 | **1.67E-17** | 46.5 | 1.72E-01 | 0.1245 | 0.017 | **2.76E-13** | 73.7 | 5.11E-02 |
|  | rs12272917 | 11q13.2 | 68019946 | *PPP6R3*** | T | 0.1088 | 0.0169 | **1.34E-10** | 53 | 1.45E-01 | 0.1094 | 0.017 | **1.15E-10** | 62.4 | 1.03E-01 | 0.1213 | 0.019 | **1.89E-10** | 0.9 | 3.15E-01 |
|  | rs884205 | 18q21.33 | 58205837 | *TNFRSF11A* | C | 0.1037 | 0.0184 | **1.84E-08** | 0 | 4.15E-01 | 0.1007 | 0.0184 | **4.73E-08** | 0 | 3.72E-01 | 0.1033 | 0.02 | 2.29E-07 | 37.5 | 2.06E-01 |

(TBLH-BMD) = total-body less head BMD, (LL-BMD) = lower limb BMD, (UL-BMD) = upper limb BMD, (SK-BMD) = skull BMD. (MODEL 0) = GWAS meta-analysis performed on age-, gender-, weight- or height-adjusted BMD, (MODEL 1a) = GWAS meta-analysis performed on age-, gender-, weight- and height-adjusted BMD, (MODEL 1b) = GWAS meta-analysis performed on age-, gender-, weight- and height-adjusted BMD measurements in individuals of European ancestry. (GENE) = closest gene, (POS) = position in the genome based on hg18, (EAF) = effect allele frequency, (*β*) = estimates of effect size expressed as adjusted SD per copy of the effect allele (EA), (SE) = standard error of *β*, (*P*) = pvalue, (I^2^) = Cochran’s Q statistic evaluating heterogeneity, (*P*_HET_) = evidence of heterogeneity and ^*^Sample sizes used for SK-BMD genome-wide meta-analysis. **Please note that *PTHLH* is also located at the 12p11.22 locus containing *KLHDC5, RSPO3* is also located at the 6q.22.32 locus containing *CENPW, FAM3C and CPED1* are also located at the 7q.31.31 locus containing *WNT16*, *TNFRSF11B* is also located at the 8q.24.12 locus containing *COLEC10, LGR4* is also located at the 11p14.1 locus containing *LIN7C* and *LRP5* is also located at the 11q13.2 locus containing *PPP6R3.*
