# Supplementary material for: Phenotypic Dissection of Bone Mineral Density Reveals Skeletal Site Specificity and Facilitates the Identification of Novel Loci in the Genetic Regulation of Bone Mass Attainment
Source: PLoS Genet. 2014 Jun 19;10(6):e1004423. doi: 10.1371/journal.pgen.1004423 (PMC4063697; doi:10.1371/journal.pgen.1004423)
Supplement: Table S10 — Top SNPs associated with bone mineral density of the total-body less head, lower limb, upper limb and skull after conditional meta-analysis. (TBLH-BMD) = total-body less head BMD, (LL-BMD) = lower limb BMD, (UL-BMD) = upper limb BMD, (SK-BMD) = skull BMD, (GENE) = closest gene, (POS) = position in the genome based on hg18, (EAF) = effect allele frequency, (β) = estimates of effect size expressed as adjusted SD per copy of the effect allele (EA), (SE) = standard error of β, (P) = P-value, (I2) = Cochran's Q statistic evaluating heterogeneity and (P HET) = evidence of heterogeneity. *Sample sizes used for SK-BMD genome-wide meta-analysis. Locus specific multiple testing correction thresholds as calculated by SNPSpD for SNPs in LD are as follows: 1p36.12 (P≤7.5×10^−5^), 2q24.3 (P≤4.7×10^−5^), 6q22.32 (P≤7.2×10^−5^), 6q23.2 (P≤8.9×10^−5^), 7q31.31 (P≤1.2×10^−4^), 8q24.12 (P≤4.7×10^−5^), 9q34.11 (P≤9.4×10^−5^), 11p14.1 (P≤1.1×10^−4^), 11q13.2 (P≤6.3×10^−5^), 12p11.22 (P≤4.1×10^−5^), 13q14.11 (P≤3.7×10^−5^), 14q32.12 (P≤4.5×10^−5^) and 18q21.33 (P≤4.1×10^−5^). **Please note that PTHLH is also located at the 12p11.22 locus containing KLHDC5 and RSPO3 is also located at the 6q.22.32 locus containing CENPW. FAM3C and CPED1 are also located at the 7q.31.31 locus containing WNT16. (DOCX) [file pgen.1004423.s026.docx]

**Table S10**. Top SNPs associated with bone mineral density of the total-body less head, lower limb, upper limb and skull after conditional meta-analysis.

|  |  |  |  |  |  | **ALSPAC (n=5330, 5299**)** | | | | **GEN-R (n=4086)** | | | | **META-ANALYSIS (n=9416, 9385**)** | | | | | |
| --- | --- | --- | --- | --- | --- | --- | --- | --- | --- | --- | --- | --- | --- | --- | --- | --- | --- | --- | --- |
| **TRAIT** | **RSID** | **LOCUS** | **POS** | **GENE** | **EA** | **EAF** | **β*** | **SE** | **P** | **EAF** | **β*** | **SE** | **P** | **EAF** | **β*** | **SE** | **P** | **I^2^** | **P_HET_** |
| **TBLH-BMD** | rs3765350 | 1p36.12 | 22319903 | *WNT4* | A | 0.78 | 0.062 | 0.023 | 8.17E-03 | 0.78 | 0.076 | 0.026 | 3.53E-03 | 0.78 | 0.0683 | 0.0174 | 8.80E-05 | 0 | 6.89E-01 |
|  | rs692995 | 2q24.3 | 166594195 | *SCN1A* | A | 0.98 | 0.125 | 0.068 | 6.72E-02 | 0.95 | 0.087 | 0.051 | 8.58E-02 | 0.96 | 0.1003 | 0.0411 | 1.46E-02 | 0 | 6.59E-01 |
|  | rs717226 | 7q31.31 | 120186120 | *KCND2* | A | 0.07 | 0.087 | 0.04 | 2.81E-02 | 0.04 | 0.136 | 0.06 | 2.28E-02 | 0.06 | 0.1025 | 0.0337 | 2.38E-03 | 0 | 4.99E-01 |
|  | rs11793535 | 9q34.11 | 132915634 | *LAMC3* | C | 0.08 | 0.103 | 0.041 | 1.13E-02 | 0.11 | 0.086 | 0.045 | 5.45E-02 | 0.10 | 0.0951 | 0.0306 | 1.89E-03 | 0 | 7.82E-01 |
|  | rs4420311 | 12p11.22 | 27875457 | *KLHDC5*** | G | 0.47 | 0.063 | 0.02 | 1.96E-03 | 0.44 | 0.096 | 0.024 | 4.94E-05 | 0.46 | 0.0768 | 0.0155 | **7.62E-07** | 8.9 | 2.95E-01 |
|  | rs17536328 | 13q14.11 | 42041029 | *TNFSF11* | T | 0.43 | 0.067 | 0.02 | 5.76E-04 | 0.40 | 0.084 | 0.022 | 1.44E-04 | 0.42 | 0.0749 | 0.015 | **5.56E-07** | 0 | 5.71E-01 |
|  | rs8019291 | 14q32.12 | 92000917 | *SLC24A4* | G | 0.87 | 0.09 | 0.029 | 1.85E-03 | 0.84 | 0.061 | 0.03 | 4.10E-02 | 0.86 | 0.0757 | 0.0211 | 3.23E-04 | 0 | 4.91E-01 |
| **LL-BMD** | rs3765350 | 1p36.12 | 22319903 | *WNT4* | A | 0.78 | 0.069 | 0.023 | 3.15E-03 | 0.78 | 0.069 | 0.026 | 8.65E-03 | 0.78 | 0.069 | 0.0175 | 8.17E-05 | 0 | 1.00E+00 |
|  | rs717226 | 7q31.31 | 120186120 | *KCND2* | A | 0.07 | 0.108 | 0.04 | 6.68E-03 | 0.04 | 0.115 | 0.06 | 5.45E-02 | 0.06 | 0.1102 | 0.0339 | 1.16E-03 | 0 | 9.24E-01 |
|  | rs11793535 | 9q34.11 | 132915634 | *LAMC3* | C | 0.08 | 0.124 | 0.041 | 2.30E-03 | 0.11 | 0.079 | 0.045 | 7.66E-02 | 0.10 | 0.1031 | 0.0308 | 8.17E-04 | 0 | 4.66E-01 |
|  | rs4420311 | 12p11.22 | 27875457 | *KLHDC5*** | G | 0.47 | 0.064 | 0.02 | 1.69E-03 | 0.44 | 0.089 | 0.024 | 1.63E-04 | 0.46 | 0.0745 | 0.0156 | **1.87E-06** | 0 | 4.30E-01 |
|  | rs8019291 | 14q32.12 | 92000917 | *SLC24A4* | G | 0.87 | 0.082 | 0.029 | 4.79E-03 | 0.84 | 0.074 | 0.03 | 1.28E-02 | 0.86 | 0.078 | 0.0212 | 2.29E-04 | 0 | 8.50E-01 |
| **UL-BMD** | rs6690148 | 1p36.12 | 22520998 | *ZBTB40* | C | 0.74 | 0.049 | 0.022 | 2.58E-02 | 0.73 | 0.07 | 0.025 | 4.62E-03 | 0.73 | 0.0583 | 0.0166 | 4.61E-04 | 0 | 5.31E-01 |
|  | rs627868 | 2q24.3 | 166007759 | *CSRNP3* | C | 0.23 | 0.025 | 0.022 | 2.75E-01 | 0.26 | 0.081 | 0.025 | 1.17E-03 | 0.25 | 0.0498 | 0.0166 | 2.76E-03 | 64.2 | 9.47E-02 |
|  | rs12201564 | 6q22.32 | 126143165 | *NCOA7* | G | 0.10 | 0.136 | 0.047 | 3.82E-03 | 0.04 | 0.095 | 0.103 | 3.57E-01 | 0.09 | 0.1288 | 0.0433 | 2.92E-03 | 0 | 7.18E-01 |
|  | rs17533951 | 7q31.31 | 120395687 | *ING3* | T | 0.89 | 0.179 | 0.05 | 3.07E-04 | 0.92 | 0.087 | 0.06 | 1.47E-01 | 0.90 | 0.1407 | 0.0387 | 2.82E-04 | 27.1 | 2.42E-01 |
|  | rs2148072 | 13q14.11 | 42060063 | *TNFSF11* | G | 0.63 | 0.06 | 0.02 | 2.89E-03 | 0.64 | 0.088 | 0.023 | 1.19E-04 | 0.63 | 0.0723 | 0.0152 | **2.05E-06** | 0 | 3.61E-01 |
| **SK-BMD** | rs3765350 | 1p36.12 | 22319903 | *WNT4* | A | 0.78 | 0.075 | 0.023 | 1.35E-03 | 0.78 | 0.023 | 0.026 | 3.72E-01 | 0.78 | 0.0518 | 0.0174 | 2.85E-03 | 54.8 | 1.37E-01 |
|  | rs4418209 | 6q22.32 | 126861392 | *CENPW*** | T | 0.54 | 0.1 | 0.019 | 2.56E-07 | 0.52 | 0.075 | 0.022 | 6.26E-04 | 0.53 | 0.0892 | 0.0145 | 7.88E-10 | 0 | 3.93E-01 |
|  | rs9483586 | 6q23.2 | 133893070 | *EYA4* | A | 0.29 | 0.063 | 0.021 | 3.35E-03 | 0.25 | 0.056 | 0.025 | 2.67E-02 | 0.27 | 0.0601 | 0.0162 | 2.14E-04 | 0 | 8.31E-01 |
|  | rs10245811 | 7q31.31 | 120459809 | *CPED1* | T | 0.22 | 0.08 | 0.024 | 6.70E-04 | 0.26 | 0.052 | 0.026 | 4.22E-02 | 0.23 | 0.0669 | 0.0178 | 1.67E-04 | 0 | 4.32E-01 |
|  | rs11573816 | 8q24.12 | 120031223 | *TNFRSF11B* | G | 0.05 | 0.109 | 0.047 | 2.15E-02 | 0.03 | 0.06 | 0.068 | 3.78E-01 | 0.04 | 0.0929 | 0.0391 | 1.74E-02 | 0 | 5.55E-01 |
|  | rs10160456 | 11p14.1 | 27293280 | *CCDC34* | C | 0.38 | 0.058 | 0.02 | 4.29E-03 | 0.38 | 0.081 | 0.023 | 5.13E-04 | 0.38 | 0.0681 | 0.0152 | **7.79E-06** | 0 | 4.54E-01 |
|  | rs3781579 | 11q13.2 | 67966294 | *LRP5*** | T | 0.89 | 0.071 | 0.032 | 2.38E-02 | 0.89 | 0.084 | 0.037 | 2.19E-02 | 0.89 | 0.0766 | 0.0244 | 1.69E-03 | 0 | 7.92E-01 |
|  | rs12960049 | 18q21.33 | 57811663 | *PIGN* | G | 0.95 | 0.106 | 0.046 | 2.08E-02 | 0.96 | 0.123 | 0.053 | 2.12E-02 | 0.95 | 0.1134 | 0.035 | 1.21E-03 | 0 | 8.10E-01 |

(TBLH-BMD) = total-body less head BMD, (LL-BMD) = lower limb BMD, (UL-BMD) = upper limb BMD, (SK-BMD) = skull BMD, (GENE) = closest gene, (POS) = position in the genome based on hg18, (EAF) = effect allele frequency, (*β*) = estimates of effect size expressed as adjusted SD per copy of the effect allele (EA), (SE) = standard error of *β*, (*P*) = *P-*value, (I^2^) = Cochran’s Q statistic evaluating heterogeneity and (*P*_HET_) = evidence of heterogeneity. ^*^Sample sizes used for SK-BMD genome-wide meta-analysis. Locus specific multiple testing correction thresholds as calculated by SNPSpD for SNPs in LD are as follows: 1p36.12 (*P* ≤ 7.5×10^-5^), 2q24.3 (*P* ≤ 4.7×10^-5^), 6q22.32 (*P* ≤ 7.2×10^-5^), 6q23.2 (*P* ≤ 8.9×10^-5^), 7q31.31 (*P* ≤ 1.2×10^-4^), 8q24.12 (*P* ≤ 4.7×10^-5^), 9q34.11 (*P* ≤ 9.4×10^-5^), 11p14.1 (*P* ≤ 1.1×10^-4^), 11q13.2 (*P* ≤ 6.3×10^-5^), 12p11.22 (*P* ≤ 4.1×10^-5^), 13q14.11 (*P* ≤ 3.7×10^-5^), 14q32.12 (*P* ≤ 4.5×10^-5^) and 18q21.33 (*P* ≤ 4.1×10^-5^). ^**^Please note that *PTHLH* is also located at the 12p11.22 locus containing KLHDC5 and *RSPO3* is also located at the 6q.22.32 locus containing *CENPW. FAM3C and CPED1* are also located at the 7q.31.31 locus containing *WNT16.*
