# Supplementary material for: Phenotypic Dissection of Bone Mineral Density Reveals Skeletal Site Specificity and Facilitates the Identification of Novel Loci in the Genetic Regulation of Bone Mass Attainment
Source: PLoS Genet. 2014 Jun 19;10(6):e1004423. doi: 10.1371/journal.pgen.1004423 (PMC4063697; doi:10.1371/journal.pgen.1004423)
Supplement: Table S11 — Lookup of selected primary and secondary BMD SNPs in the publically released GEFOS GWAS of hip and spine BMD, in addition to a comparison of the summary statistics across each skeletal site before conditional analysis. (TBLH-BMD) = total-body less head BMD; (LL-BMD) = lower limb BMD; (UL-BMD) = upper limb BMD; (SK-BMD) = skull BMD; (LS-BMD) = lumbar spine BMD; (FN-BMD) = femoral neck BMD; (GENE) = closest gene; (EA) = effect allele; (β) = estimates of effect size expressed as adjusted SD per copy of the effect allele (EA); (SE) = standard error of β and (P) = pvalue. Note – all the summary statistics refer to those obtained prior to conditional analysis and in the case of femoral neck or lumbar spine, the results were obtained from the publically available data release from the GEFOS consortium. (DOCX) [file pgen.1004423.s027.docx]

**Table S11**. Lookup of selected primary and secondary BMD SNPs in the publically released GEFOS GWAS of hip and spine BMD, in addition to a comparison of the summary statistics across each skeletal site before conditional analysis.

|  |  |  |  | **TBLH-BMD** | | | **LL-BMD** | | | **UL-BMD** | | | **SK-BMD** | | | **FN-BMD** | | | | **LS-BMD** | |
| --- | --- | --- | --- | --- | --- | --- | --- | --- | --- | --- | --- | --- | --- | --- | --- | --- | --- | --- | --- | --- | --- |
| **RSID** | **GENE** | **LOCUS** | **EA** | ***β*** | **SE** | ***P*** | ***β*** | **SE** | ***P*** | ***β*** | **SE** | ***P*** | ***β*** | **SE** | ***P*** | ***β*** | ***P*** | ***β*** | ***P*** | |  |
| rs754388 | *RIN3* | 14q32.12 | C | 0.12 | 0.020 | **2.96E-09** | 0.13 | 0.020 | **1.40E-10** | 0.10 | 0.020 | **3.13E-07** | 0.04 | 0.020 | 7.82E-02 | +/- | 8.69E-01 | - | 4.22E-01 | |  |
| rs2130604 | *CENPW* | 6q22.32 | T | 0.03 | 0.017 | 6.16E-02 | 0.02 | 0.017 | 2.82E-01 | 0.04 | 0.017 | **2.42E-02** | 0.11 | 0.017 | **3.33E-11** | +/- | 9.75E-01 | + | 3.98E-01 | |  |
| rs4418209 | *CENPW* | 6q22.32 | T | -0.03 | 0.015 | **1.74E-02** | -0.04 | 0.015 | **6.52E-03** | -0.04 | 0.015 | **8.18E-03** | 0.07 | 0.015 | **1.07E-06** | - | **4.98E-03** | - | 3.35E-01 | |  |
| rs3012465 | *EYA4* | 6q23.2 | G | 0.02 | 0.015 | 1.30E-01 | 0.02 | 0.015 | 2.09E-01 | 0.05 | 0.015 | **7.45E-04** | 0.13 | 0.015 | **8.29E-17** | - | **7.05E-03** | - | **3.86E-02** | |  |
| rs10160456 | *LIN7C* | 11p14.1 | C | 0.07 | 0.015 | **1.92E-05** | 0.07 | 0.015 | **1.81E-05** | 0.05 | 0.015 | **3.06E-03** | 0.10 | 0.015 | **2.02E-10** | + | 5.79E-02 | + | **3.13E-02** | |  |
| rs4420311 | *KLHDC5* | 12p11.22 | G | 0.09 | 0.016 | **4.44E-08** | 0.09 | 0.016 | **3.21E-08** | 0.07 | 0.016 | **2.25E-05** | 0.04 | 0.016 | **1.58E-02** | + | 3.32E-01 | - | 4.47E-01 | |  |
| rs2148072 | *TNFSF11* | 13q14.11 | G | 0.06 | 0.015 | **1.61E-04** | 0.05 | 0.015 | **4.58E-04** | 0.05 | 0.015 | **1.19E-03** | 0.01 | 0.015 | 7.36E-01 | +/- | 8.70E-01 | - | **4.98E-02** | |  |
| rs17536328 | *TNFSF11* | 13q14.11 | T | 0.09 | 0.015 | **7.58E-09** | 0.07 | 0.015 | **1.19E-05** | 0.09 | 0.015 | **3.08E-09** | 0.06 | 0.015 | **2.03E-04** | + | **3.76E-02** | + | 3.35E-01 | |  |

(TBLH-BMD) = total-body less head BMD; (LL-BMD) = lower limb BMD; (UL-BMD) = upper limb BMD; (SK-BMD) = skull BMD; (LS-BMD) = lumbar spine BMD; (FN-BMD) = femoral neck BMD; (GENE) = closest gene; (EA) = effect allele; (*β*) = estimates of effect size expressed as adjusted SD per copy of the effect allele (EA); (SE) = standard error of *β* and (*P*) = pvalue. Note – all the summary statistics refer to those obtained prior to conditional analysis and in the case of femoral neck or lumbar spine, the results were obtained from the publically available data release from the GEFOS consortium.
