# Supplementary material for: Phenotypic Dissection of Bone Mineral Density Reveals Skeletal Site Specificity and Facilitates the Identification of Novel Loci in the Genetic Regulation of Bone Mass Attainment
Source: PLoS Genet. 2014 Jun 19;10(6):e1004423. doi: 10.1371/journal.pgen.1004423 (PMC4063697; doi:10.1371/journal.pgen.1004423)
Supplement: Table S12 — Published SNPs used for conditional meta-analyses. (LS-BMD) = lumbar spine BMD; (FN-BMD) = femoral neck BMD; (F-BMD) = forearm BMD; (TBLH-BMD) = total-body less head BMD; (SK-BMD) = skull BMD; BMD; (TRABEC-BMD) = volumetric trabecular BMD of the tibia; (CORT-BMD) = volumetric cortical BMD of the tibia. (POSITION) = location in the genome based on hg18; (GENE) = closest gene; (PMID) = accession number of the publication in Pubmed from which the summary statistics were obtained; (β) = estimates of effect size expressed as adjusted SD per copy of the effect allele; (SE) = standard error of β and (P) = P-value. *Please note that PTHLH is also located at the 12p11.22 locus containing KLHDC5, RSPO3 is also located at the 6q.22.32 locus containing CENPW, FAM3C and CPED1 are also located at the 7q.31.31 locus containing WNT16, TNFRSF11B is also located at the 8q.24.12 locus containing COLEC10, LGR4 is also located at the 11p14.1 locus containing LIN7C and LRP5 is also located at the 11q13.2 locus containing PPP6R3. **The Generation R cohort did not impute the published FUBP3 SNP (rs7851693) and therefore we chose to condition on rs7030440, a SNP which was in high LD (HapMap phase 2 release 22, CEU: r2 = 0.96) with the published FUBP3 associated BMD variant. ***No previous BMD SNPs found in 14q32.12 have been published. (DOCX) [file pgen.1004423.s028.docx]

**Table S12**. Published SNPs used for conditional meta-analyses

|  |  |  |  |  | **LS-BMD** | | **FN-BMD** | | **F-BMD** | | **TBLH-BMD** | | **SK-BMD** | | **TRABEC-BMD** | | **CORT-BMD** | | |
| --- | --- | --- | --- | --- | --- | --- | --- | --- | --- | --- | --- | --- | --- | --- | --- | --- | --- | --- | --- |
| **LOCUS** | **SNPID** | **POSITION** | **GENE** | **PMID** | ***β*** | ***P*** | ***β*** | ***P*** | ***β*** | ***P*** | ***β*** | ***P*** | ***β*** | ***P*** | ***β*** | ***P*** | ***β*** | ***P*** |  |
| **1p36.12** | rs7521902 | 22363311 | *WNT4* | 22504420 | -0.05 | 1.0E-10 | -0.04 | 2.8E-09 | - | - | - | - | - | - | - | - | - | - |  |
|  | rs7524102 | 22571034 | *ZBTB40* | 19079262 | -0.11 | 7.0E-09 | -0.15 | 1.0E-16 | - | - | - | - | - | - | - | - | - | - |  |
|  | rs6696981 | 22575445 | *ZBTB40* | 19079262 | -0.12 | 2.0E-08 | -0.14 | 2.5E-12 | - | - | - | - | - | - | - | - | - | - |  |
|  | rs6426749 | 22584060 | *ZBTB40* | 22504420 | 0.10 | 1.8E-44 | 0.11 | 7.0E-57 | - | - | - | - | - | - | - | - | - | - |  |
|  | rs7543680 | 22603856 | *ZBTB40* | 19079262 | -0.07 | 9.0E-06 | -0.10 | 1.0E-10 | - | - | - | - | - | - | - | - | - | - |  |
| **2q24.3** | rs6710518 | 166291490 | *GALNT3* | 21533022 | - | - | -0.06 | 5.0E-10 | - | - | - | - | - | - | - | - | - | - |  |
|  | rs1346004 | 166309292 | *GALNT3* | 22504420 | -0.06 | 4.0E-30 | -0.05 | 1.0E-25 | - | - | - | - | - | - | - | - | - | - |  |
| **6q22.33** | rs13204965 | 127208765 | *RSPO3* | 22504420 | 0.04 | 3.6E-10 | 0.04 | 8.0E-12 | - | - | - | - | - | - | - | - | - | - |  |
| **6q23.2** | rs271170 | 133357497 | *EYA4* | 23437003 | - | - | - | - | - | - | - | - | - | - | -0.02 | 5.4E-01 | -0.11 | 2.7E-12 |  |
| **7q31.31** | rs7801723 | 120561396 | *CPED1* | 22792070 | - | - | - | - | - | - | 0.08 | 2.0E-10 | 0.14 | 9.0E-28 | - | - | - | - |  |
|  | rs13245690 | 120572300 | *CPED1* | 22504420 | 0.05 | 2.0E-11 | 0.02 | 8.2E-04 | - | - | - | - | - | - | - | - | - | - |  |
|  | rs10274324 | 120686577 | *CEPD1* | 22792071 | - | - | - | - | -0.21 | 3.8E-08 | - | - | - | - | - | - | - | - |  |
|  | rs4609139 | 120691051 | *CPED1* | 22792070 | - | - | - | - | - | - | -0.08 | 1.0E-10 | -0.12 | 1.0E-21 | - | - | - | - |  |
|  | rs2908004 | 120757005 | *WNT16* | 22792071 | - | - | - | - | -0.16 | 1.0E-15 | - | - | - | - | - | - | - | - |  |
|  | rs2536189 | 120760857 | *WNT16* | 22792071 | - | - | - | - | -0.15 | 8.5E-16 | - | - | - | - | - | - | - | - |  |
|  | rs3801387 | 120762001 | *WNT16* | 22504420 | -0.09 | 3.0E-51 | -0.08 | 5.0E-40 | - | - | - | - | - | - | - | - | - | - |  |
|  | rs2707466 | 120766325 | *WNT16* | 22792071 | - | - | - | - | -0.14 | 2.3E-12 | - | - | - | - | - | - | - | - |  |
|  | rs917727 | 120805815 | *FAM3C* | 22792070 | - | - | - | - | - | - | 0.15 | 1.2E-27 | 0.12 | 1.9E-16 | - | - | - | - |  |
|  | rs7776725 | 120820357 | *FAM3C* | 22792071 | - | - | - | - | -0.17 | 8.5E-15 | - | - | - | - | - | - | - | - |  |
| **8q24.12** | rs4355801 | 119993054 | *TNFRSF11B* | 18455228 | -0.09 | 8.0E-10 | -0.09 | 3.3E-10 | - | - | - | - | - | - | - | - | - | - |  |
|  | rs7839059 | 120045723 | *TNFRSF11B* | 23437003 | - | - | - | - | - | - | - | - | - | - | -0.06 | 4.7E-02 | -0.10 | 1.2E-09 |  |
|  | rs2062375 | 120046973 | *TNFRSF11B* | 20548944 | 0.14 | 3.0E-11 | - | - | - | - | - | - | - | - | - | - | - | - |  |
|  | rs2062377 | 120076601 | *TNFRSF11B* | 22504420 | -0.08 | 3.0E-39 | -0.06 | 9.0E-25 | - | - | - | - | - | - | - | - | - | - |  |
|  | rs6469792 | 120077552 | *TNFRSF11B* | 19079262 | -0.11 | 1.1E-15 | -0.10 | 3.1E-13 | - | - | - | - | - | - | - | - | - | - |  |
|  | rs11995824 | 120081881 | *TNFRSF11B* | 19801982 | -0.09 | 1.0E-15 | -0.07 | 7.0E-09 | - | - | - | - | - | - | - | - | - | - |  |
|  | rs6469804 | 120114010 | *TNFRSF11B* | 19079262 | -0.11 | 1.4E-15 | -0.08 | 7.0E-10 | - | - | - | - | - | - | - | - | - | - |  |
|  | rs6993813 | 120121419 | *TNFRSF11B* | 19079262 | -0.11 | 2.1E-15 | -0.09 | 1.5E-11 | - | - | - | - | - | - | - | - | - | - |  |
| **9q34.11**** | rs7851693 | 132468648 | *FUBP3* | 22504420 | 0.03 | 6.0E-08 | 0.05 | 3.0E-22 | - | - | - | - | - | - | - | - | - | - |  |
| **11p14.1** | rs10835187 | 27462253 | *LIN7C* | 22504420 | -0.03 | 5.0E-08 | -0.01 | 3.0E-02 | - | - | - | - | - | - | - | - | - | - |  |
| **11q13.2** | rs599083 | 67948922 | *LRP5* | 19801982 | -0.07 | 5.0E-08 | -0.05 | 9.7E-05 | - | - | - | - | - | - | - | - | - | - |  |
|  | rs3736228 | 67957871 | *LRP5* | 22504420 | -0.08 | 2.0E-26 | -0.05 | 4.8E-11 | - | - | - | - | - | - | - | - | - | - |  |
| **12p11.22** | rs7953528 | 27908426 | *KLHDC5* | 22504420 | -0.01 | 1.3E-01 | 0.05 | 1.87E‐12 | - | - | - | - | - | - | - | - | - | - |  |
| **13q14.11** | rs9533090 | 41849449 | *AKAP11* | 22504420* | -0.10 | 5.0E-68 | -0.05 | 5.0E-23 | - | - | - | - | - | - | - | - | - | - |  |
|  | rs9594738 | 41850145 | *TNFSF11* | 19079262 | -0.16 | 4.0E-23 | -0.01 | 1.4E-09 | - | - | - | - | - | - | - | - | - | - |  |
|  | rs9533093 | 41859597 | *TNFSF11* | 19079262 | -0.11 | 5.4E-11 | -0.04 | 3.8E-02 | - | - | - | - | - | - | - | - | - | - |  |
|  | rs9594759 | 41930593 | *TNFSF11* | 19079262 | -0.12 | 2.0E-17 | -0.07 | 1.2E-06 | - | - | - | - | - | - | - | - | - | - |  |
|  | rs1021188 | 42014133 | *TNFSF11* | 23437003 | - | - | - | - | - | - | - | - | - | - | -0.01 | 8.5E-01 | -0.15 | 3.6E-14 |  |
| **14q32.12***** | rs754388 | 92185163 | *RIN3* | n/a | - | - | - | - | - | - | - | - | - | - | - | - | - | - |  |
| **18q21.33** | rs884205 | 58205837 | *TNFRSF11A* | 22504420 | -0.05 | 2.0E-17 | -0.04 | 3.0E-10 | - | - | - | - | - | - | - | - | - | - |  |
|  | rs3018362 | 58233073 | *TNFRSF11A* | 19079262 | -0.06 | 8.0E-06 | -0.08 | 5.4E-08 | - | - | - | - | - | - | - | - | - | - |  |

(LS-BMD) = lumbar spine BMD; (FN-BMD) = femoral neck BMD; (F-BMD) = forearm BMD; (TBLH-BMD) = total-body less head BMD; (SK-BMD) = skull BMD; BMD; (TRABEC-BMD) = volumetric trabecular BMD of the tibia; (CORT-BMD) = volumetric cortical BMD of the tibia. (POSITION) = location in the genome based on hg18; (GENE) = closest gene; (PMID) = accession number of the publication in Pubmed from which the summary statistics were obtained; (*β*) = estimates of effect size expressed as adjusted SD per copy of the effect allele; (SE) = standard error of *β* and (*P*) = *P*-value. *Please note that *PTHLH* is also located at the 12p11.22 locus containing *KLHDC5, RSPO3* is also located at the 6q.22.32 locus containing *CENPW, FAM3C and CPED1* are also located at the 7q.31.31 locus containing *WNT16*, *TNFRSF11B* is also located at the 8q.24.12 locus containing *COLEC10, LGR4* is also located at the 11p14.1 locus containing *LIN7C* and *LRP5* is also located at the 11q13.2 locus containing *PPP6R3.*

**The Generation R cohort did not impute the published *FUBP3* SNP (rs7851693) and therefore we chose to condition on rs7030440, a SNP which was in high LD (HapMap phase 2 release 22, CEU: r^2^=0.96) with the published *FUBP3* associated BMD variant.

***No previous BMD SNPs found in *14q32.12* have been published.
