# Supplementary material for: Phenotypic Dissection of Bone Mineral Density Reveals Skeletal Site Specificity and Facilitates the Identification of Novel Loci in the Genetic Regulation of Bone Mass Attainment
Source: PLoS Genet. 2014 Jun 19;10(6):e1004423. doi: 10.1371/journal.pgen.1004423 (PMC4063697; doi:10.1371/journal.pgen.1004423)
Supplement: Table S13 — Comparison of transcript levels between healthy and osteoporotic women. Transcript log2 signal levels expressed from genes ±250 Kb of rs754388 were compared between postmenopausal osteoporotic women with fracture and healthy controls using students T-test. Transcripts with maximal log2 signal values below 4 were excluded. (SD) = Standard deviation and (P) = P-value. (DOCX) [file pgen.1004423.s029.docx]

**Table S13**. Comparison of transcript levels between healthy and osteoporotic women

|  |  | **HEALTHY (n =39)** | | **OSTEOPOROTIC (n=27)** | |  |
| --- | --- | --- | --- | --- | --- | --- |
| **AFFYMETRIX ID** | **GENE SYMBOL** | **SIGNAL LEVEL** | **SD** | **SIGNAL LEVEL** | **SD** | **P** |
| 215771_x_at | *RET* | 5.23 | 0.22 | 5.16 | 0.15 | 0.1779 |
| 201212_at | *LGMN* | 10.45 | 0.32 | 10.58 | 0.33 | 0.1151 |
| 206790_s_at | *NDUFB1* | 11.25 | 0.09 | 11.28 | 0.11 | 0.2438 |
| 222069_s_at | *LOC100287697* | 4.37 | 0.16 | 4.41 | 0.17 | 0.3408 |
| 1568922_at | *SLC24A4* | 4.69 | 0.16 | 4.67 | 0.12 | 0.502 |
| 243969_at | *SLC24A4* | 5.47 | 0.38 | 5.41 | 0.27 | 0.4959 |
| 1568870_at | *SLC24A4* | 6.55 | 0.4 | 6.48 | 0.31 | 0.449 |
| **220439_at** | ***RIN3*** | **5.4** | **0.19** | **5.26** | **0.16** | **0.0027** |
| 1562005_at | *RIN3* | 5.07 | 0.24 | 5.09 | 0.19 | 0.6858 |
| 219456_s_at | *RIN3* | 6.04 | 0.17 | 6.11 | 0.14 | 0.0961 |
| 219457_s_at | *RIN3* | 7.53 | 0.17 | 7.54 | 0.15 | 0.769 |
| 60471_at | *RIN3* | 8.19 | 0.16 | 8.19 | 0.15 | 0.9363 |
| 218241_at | *GOLGA5* | 8.21 | 0.11 | 8.16 | 0.1 | 0.0869 |

Transcript log2 signal levels expressed from genes ± 250 Kb of rs754388 were compared between postmenopausal osteoporotic women with fracture and healthy controls using students T-test. Transcripts with maximal log2 signal values below 4 were excluded. (SD) = Standard deviation and (*P*) = *P*-value.
